# Tailored phenyl ureas eradicate drug-resistant *Mycobacterium tuberculosis* by targeting mycolic acid cell wall assembly†

**DOI:** 10.1039/d5sc02565f

**Published:** 2025-04-30

**Authors:** Dietrich Mostert, Josef Braun, Matthew D. Zimmerman, Curtis A. Engelhart, Sara Berndl, Patrick K. Quoika, Andreas M. Kany, Julianna Proietto, Suyapa Penalva-Lopez, Joshua B. Wallach, Anna K. H. Hirsch, Martin Zacharias, Dirk Schnappinger, Véronique Dartois, Stephan A. Sieber

**Affiliations:** a Center for Functional Protein Assemblies, Department of Bioscience, TUM School of Natural Sciences, Technical University of Munich Ernst-Otto-Fischer-Straße 8 85748 Garching Germany stephan.sieber@tum.de; b Center for Discovery and Innovation, Hackensack Meridian Health Nutley New Jersey USA; c Department of Microbiology and Immunology, Weill Cornell Medical College New York NY USA; d Helmholtz Institute for Pharmaceutical Research Saarland (HIPS) – Helmholtz Centre for Infection Research (HZI) Campus E8.1 66123 Saarbrücken Germany; e Deutsches Zentrum für Infektionsforschung (DZIF) e.V. 38124 Braunschweig Germany; f Saarland University, Department of Pharmacy 66123 Saarbrücken Germany

## Abstract

Treatment of *Mycobacterium tuberculosis* infections is a challenging task due to long treatment regiments and a growing number of resistant clinical isolates. To identify new antibiotic hits, we screened a focused library of 400 synthetic compounds derived from a recently discovered molecule with promising anti-mycobacterial activity. A suite of more potent hit molecules was deciphered with sub-micromolar activity. Utilising tailored affinity-based probes for chemical proteomic investigations, we successfully pinpointed the mycolic acid transporter MmpL3 and two epoxide hydrolases, EphD and EphF, also linked to mycolic acid biosynthesis, as specific targets of the compounds. These targets were thoroughly and independently validated by activity assays, under- and overexpression, resistance generation, and proteomic studies. Structural refinement of the most potent hit molecules led to the development of a new lead compound that demonstrates enhanced biological activity in *M. tuberculosis*, low human cytotoxicity, and improved solubility and oral bioavailability – traits that are often challenging to achieve with anti-mycobacterial drugs. Overall, drug-likeness, as well as the dual mode of action, addressing the mycolic acid cell wall assembly at two distinct steps, holds significant potential for further *in vivo* applications.

## Abstract 2

Treatment of *Mycobacterium tuberculosis* infections is a challenging task due to long treatment regiments and a growing number of resistant clinical isolates.

## Graphical abstract

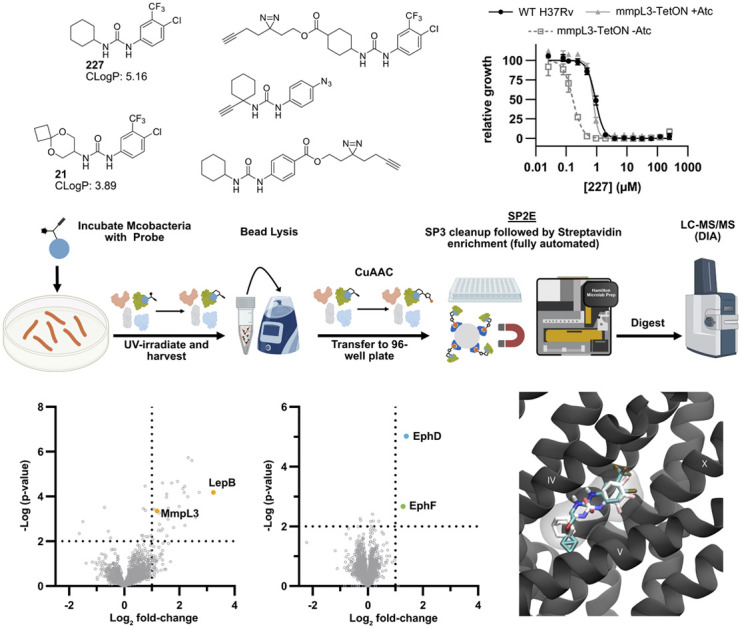

## Introduction

**Fig. 1:**
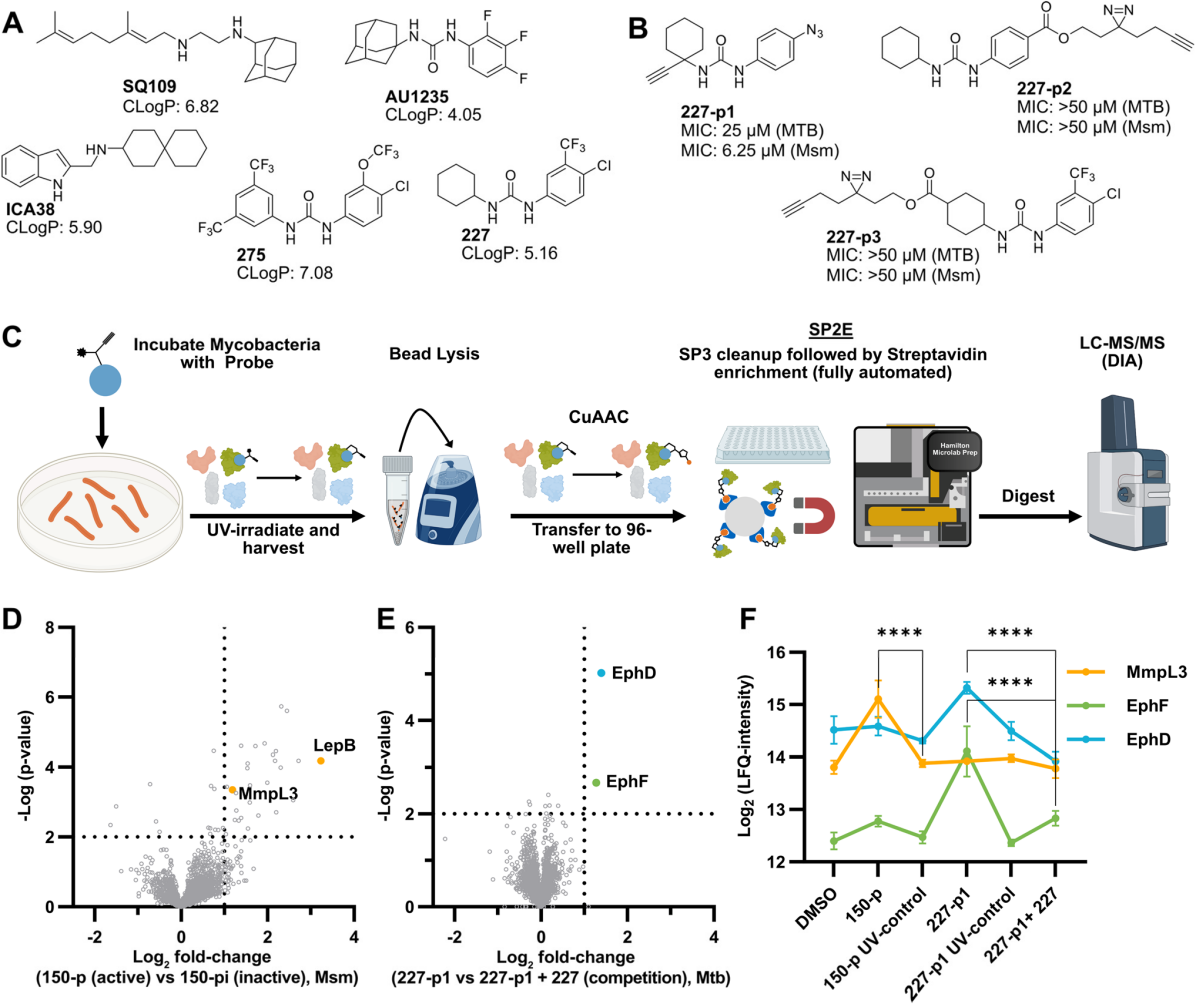
AfBPP to decipher protein targets. (A) Structures of known MmpL3 inhibitors SQ109, AU1235, and ICA38 and the best two hits, 275 and 227, from a screen against *M. smegmatis* and their respective *C* log *P*-values. (B) Structures of affinity-based probes based on the structure of 227 and their respective MIC against MTB H37Ra and *M. smegmatis* DSM43756. (C) Schematic overview of the AfBPP workflow used in this study. An affinity-based probe is incubated with a mycobacterial culture followed by UV-irradiation for covalent attachment of the probe to its bound protein. After cell lysis, the protein-bound probe is clicked to a biotin-azide, the proteins are captured on magnetic beads, and the probe-bound proteins are enriched using streptavidin beads. After tryptic digest, the bound proteins can be identified by LC-MS/MS analysis. (D) Volcano-plot of *M. smegmatis* cells treated with 12.5 μM 150-p compared to 150-pi. Both MmpL3 and LepB (both essential) are significantly enriched. Dotted lines indicate significance cut-off at *p* < 0.01 (*n* = 7) and a log_2_(fold change) >1. (E) Volcano-plot of *M. tuberculosis* cells treated with 5 μM 227-p1 compared to 5 μM 227-p1 in competition with 25 μM 227. Both EphD and EphF are significantly enriched. Dotted lines indicate significance cut-off at *p* < 0.01 (*n* = 4) and a log_2_(fold change) >1. (F) Profile plot of the mean LFQ-values of MmpL3, EphF and EphD across different proteomic samples. 150-p significantly enriches MmpL3 compared to its UV-control (*p* < 0.0001, *n* = 7), while 227-p1 significantly enriches EphF and EphD and is out-competed by 227 (*p* < 0.0001, *n* = 4), adjusted *p*-values, two-way ANOVA.

With rising numbers of multi-resistant superbugs, healthcare professionals desperately call for novel strategies to fight pathogenic bacteria, with particular emphasis on those with already limited treatment options.^1–3^ Especially *Mycobacterium tuberculosis* (Mtb) poses a significant challenge due to its elaborate and unique cell wall structure, effectively preventing the penetration of small molecules.^4,5^ Thus, the treatment of Mtb infections has so far relied on several drugs that must be applied in combination for several months, often with severe side effects.^6^ Although the TB drug discovery pipeline has improved over the past 20 years, highlighted by the approvals of bedaquiline, delamanid, and pretomanid,^7–9^ the continued rise of multidrug-resistant (MDR-TB) and extensively drug-resistant Mtb (XDR-TB) has significantly impaired the effectiveness of many compounds.^3,10^ Since 2022, the WHO recommends an all-oral regimen of bedaquiline, pretomanide, linezolid, and moxifloxacin (BPaLM) for the treatment of MDR-TB and rifampicin-resistant TB (RR-TB).^11^ While these new drug regimens are helping in the ongoing fight of treating TB, the rising number of bedaquiline-resistant strains poses a risk to these new regimens,^12,13^ highlighting the continued need for new drugs in the pipeline. Current front-line antibiotics comprise compounds such as isoniazid (INH), ethambutol, rifamycin, and pyrazinamide, of which INH and ethambutol address the cell wall biosynthesis as a hot spot target. For example, INH is a prodrug that forms a radical intermediate readily reacting with NADH upon activation by catalase peroxidase (KatG). The resulting NADH-INH conjugate effectively blocks enoyl-acyl carrier protein reductase (InhA), which is crucial for the biosynthesis of essential cell wall mycolic acids. Mutations in the prodrug activating KatG are one of the major INH resistance mechanisms limiting its application.^14^ Several other targets in the cell wall biosynthesis pathway have been identified as sweet spots to kill the pathogen, including d-alanyl-d-alanine ligase,^15^ polyketide synthase Pks13 (ref. 16) and mycobacterial membrane protein large (MmpL3).^17–25^ MmpL3 is a membrane transporter required for the translocation of trehalose monomycolates (TMM) across the Mtb inner membrane, where the mycolic acid chain is transferred to arabinogalactan or TMM to yield trehalose dimycolate (TDM) *via* the Ag85 complex.^26–30^ Due its essentiality, MmpL3 is regarded as a promising drug target.^20,31^ Several inhibitors of MmpL3 have been identified *via* high-throughput screens and rational design campaigns.^21,32^ Among those, SQ109, an ethylene diamine derivative, was the most advanced and reached clinical phase 2 (ref. 33) (Fig. 1A). In addition, carboxamides, benzothiazole amides, pyrroles, benzimidazoles, spiropiperidines, phenylureas (HC2169, HC2138),^34^ and adamantly ureas (AU1235) have been reported as MmpL3 inhibitors.^20,35–38^ The co-crystal structures of several MmpL3 inhibitors, including SQ109, AU1235, and ICA38, have been obtained, demonstrating a conserved binding pocket in the proton translocating channel^39^ (Fig. 1A). For most compounds, MmpL3 was confirmed as a target *via* sequencing of resistant strains with corresponding mutations in the bespoke binding site.^37,40^ Some of these molecules also address additional targets, such as menaquinone biosynthesis enzymes, dissipation of the proton motive force, and epoxide hydrolases (Eph).^41,42^ However, limitations of the current MmpL3 compound generations include the decoration by large lipophilic, non-aromatic groups associated with high *C* log *P* values low solubility and limited pharmacokinetics (PK).^17^

Here, we follow-up on a recently discovered diphenyl urea antibiotic, PK150,^43^ designed against methicillin-resistant *Staphylococcus aureus* (MRSA) but also displaying notable activity against Mtb with an unknown MoA. An in-house screen of 450 diverse PK150 analogues and subsequent chemical refinement of an initial hit revealed a nanomolar Mtb antibiotic active against drug-resistant isolates. Target identification with tailored probes deciphered MmpL3 and two epoxide hydrolases as cellular targets, which were subsequently validated by activity assays, target over- and under-expression, and sequencing of resistant isolates. The novel antibiotic exhibited suitable solubility, stability, PK properties, and low human toxicity.

## Results and discussion

### Screening of an in-house urea library reveals a potent Mtb antibiotic hit molecule

The diphenyl urea compound PK150 (Fig. S1†) was previously shown to rapidly kill *S. aureus* by a dual mode of action targeting menaquinone methyltransferase MenG and the signal peptidase SpsB.^43^ The compound was also tested against other bacteria and displayed high activity against Mtb with a minimal inhibitory concentration (MIC) of 6.25 μM. However, the underlying MoA in Mtb remains unknown. Prior to an in-depth target deconvolution, we screened our urea in-house library comprising 400 compounds against *M. smegmatis* (Msm), an easier-to-handle surrogate of Mtb, to search for hits with even better activity. 26 compounds displayed the same or better MIC values than PK150 (3.1 μM in Msm) with 275 as the most potent derivative (MIC = 0.8 μM) (Fig. 1A). We selected the top 3 hits plus PK150 as a reference for a counter screen against Mtb H37Rv, which largely confirmed their potency. 227, a cyclohexyl substituted phenyl urea, stood out with the best MIC of 1.8 μM, which is in the same range as front-line antibiotics such as ethambutol.

### Chemical proteomics reveal essential targets in the mycolic acid pathway

**Scheme 1:**
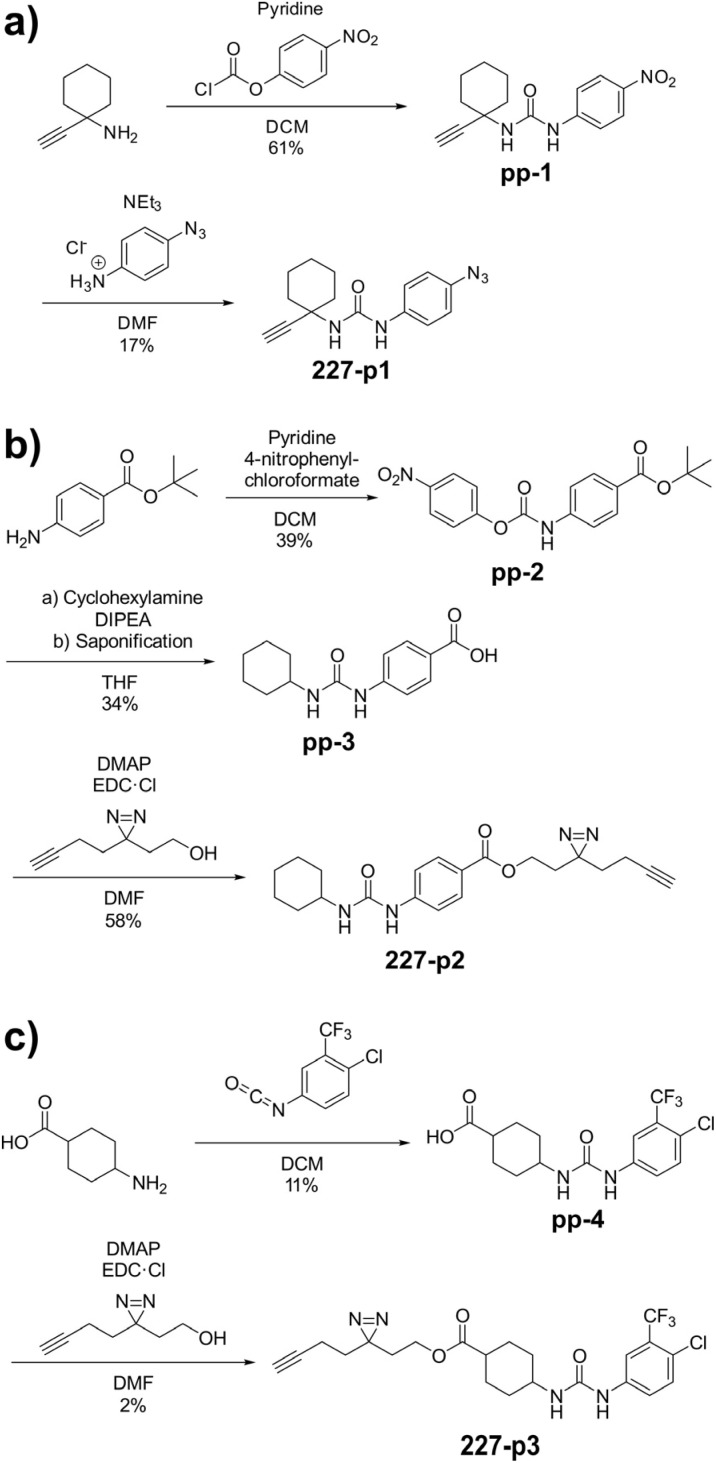
Synthesis of affinity based probes 227-p1 (a), 227-p2 (b), and 227-p3 (c) based on the structure of 227.

Prior to chemical proteomic studies, we excluded unspecific effects on the membrane integrity. No pronounced membrane disruption was observed for the most active compounds (Fig. S2†). To decipher the cellular targets responsible for the antibiotic effect, we first designed and synthesized three probes closely mimicking 227 (Scheme 1). In the first case, the aryl ring was substituted with an azide moiety to install a photocrosslinker. The cyclohexyl ring was equipped with an alkyne handle to enrich bound proteins *via* click chemistry to affinity handles (227-p1). In the second probe, we appended a minimal alkyne photocrosslinker to the aryl ring (227-p2) and in the third probe on the cyclohexyl ring (227-p3) (Fig. 1B). In brief, the synthesis of 227-p1 was started by coupling 1-Ethinylcyclohexylamine to -nitrophenyl chloroformate to yield carbamate probe-precursor 1 (pp-1). We then converted the carbamate with 4-azido aniline to probe 227-p1 (Scheme 1a). The second probe 227-p2 was synthesized from *tert*-butyl 4-aminobenzoate, converting it with 4-nitrophenyl chloroformate to the respective carbamate pp-2. Replacement of nitrophenol by cyclohexylamine and simultaneous saponification yielded the acid pp-3. The photoprobe 227-p2 was obtained by esterification of the free acid with the minimal photocrosslinker (Scheme 1b). The final probe 227-p3 was synthesized from 4-aminocyclohexane-carboxylic acid, which was first converted with the respective isocyanate to urea pp-4. The urea then was esterified with the minimal photocrosslinker to yield the probe 227-p3 (Scheme 1c). All probes were tested for their anti-mycobacterial activities, and although probes 227-p2 and 227-p3 were inactive, 227-p1 retained antibiotic activity albeit with a higher MIC of 25 μM in MTB H37Ra. In addition, we used the existing PK150-like probe (150-p), which exhibited a MIC of 25 μM and an inactive analogue (150-ip) as control (Fig. S1†).

We initiated affinity-based protein profiling (AfBPP) studies by 150-p labelling in living Msm cells (Fig. 1C). Incubation of live bacteria with various probe concentrations was followed by lysis, click to rhodamine azide, and SDS-gel analysis *via* fluorescence scanning.^44–47^ Several fluorescent bands were visible on the gel with a concentration for a good signal to noise ratio of 12.5 μM (Fig. S3A†). We commenced the study by treatment of Msm cells with 150-p and 150-ip, followed by lysis, click to biotin azide, enrichment on avidin beads, and tryptic digest to release peptides for LC-MS/MS analysis *via* label free-quantification (LFQ).^48^ Identified proteins were visualized in a volcano plot with significantly enriched targets (*p*-value < 0.01), fold-change >2 (log_2_(1)) displayed on the upper right side (Fig. 1D and Table S1†). Among the proteins solely enriched by the active probe, MmpL3 and the signal peptidase LepB were the only proteins assigned to be essential for mycobacterial growth. Of note, MmpL3 was also significantly enriched when Mtb H37Ra cells were labelled with 150-p (Fig. S3E†).

To gain further insight into the target scope of our hit-compound 227, we applied the optimised 227-p1 probe in the labelling of intact Mtb H37Ra, with a pre-treatment of the bacteria with a 5-fold excess of parent 227 prior to probe addition, to verify high-confident targets by competition (Fig. 1E). Interestingly, two epoxide hydrolases, EphD and EphF, involved in mycolic acid metabolism, were the only competed hits and thus pertained as high confident targets. MmpL3 could not be enriched in the case of 227-p1 (Fig. 1F), which might be due to the different location of the photocrosslinker compared to 150-p and the introduction of the zwitterionic azide which might hinder the probe from getting into the MmpL3 binding pocket. Overall, chemical proteomic studies with two different affinity-based probes suggest that LepB, MmpL3, and two epoxide hydrolases are putative targets which are selected for in-depth validation.

### MmpL3 and two epoxide hydrolases are targets of 227 and derivatives

The signal peptidase LepB is essential for the cleavage of protein signal tags prior to secretion and is needed for survival. Although the corresponding signal peptidase of *S. aureus*, SpsB, was a significant target of PK150, the corresponding LepB assay with Mycobacterial membranes did not show any inhibition compared to MD3, a known mycobacterial LepB inhibitor (Fig. S4†). We thus regard this enzyme as to not being involved in the mechanism of action of the new compounds.

**Fig. 2:**
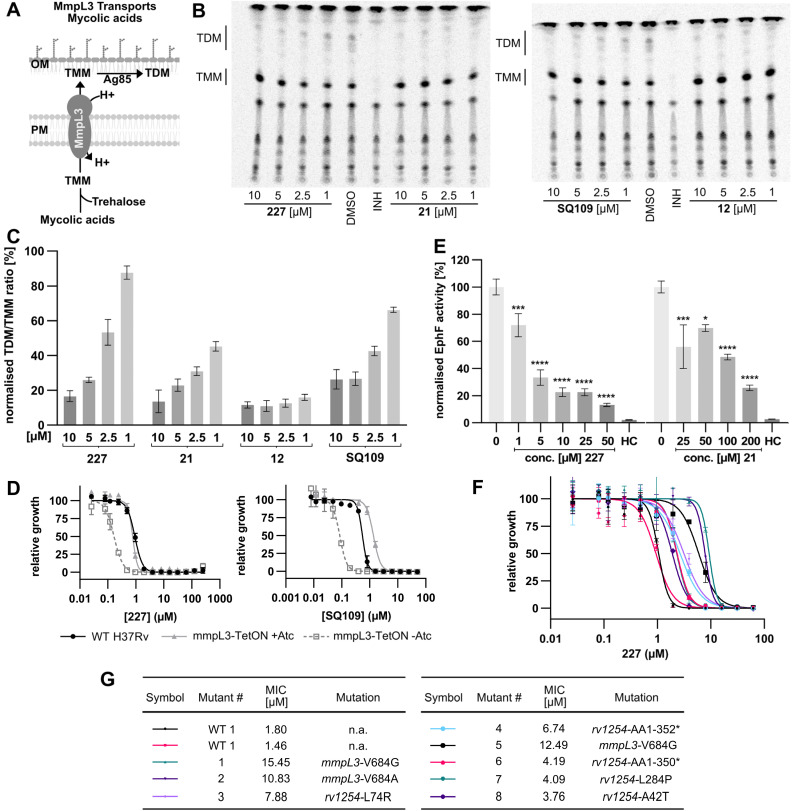
227 and its derivatives inhibit MmpL3 and EphF. (A) MmpL3 is an essential mycolic acid transporter that exports trehalose monomycolate (TMM) across the mycomembrane, where it is then converted to trehalose dimycolate (TDM) by Ag85 before being incorporated into the cell wall of Mycobacteria. (B) Representative TLC plates (pooled replicates) used to determine the TDM to TMM ratio. The spots corresponding to TDM and TMM were quantified, the background was subtracted, and the ratio of TDM to TMM was calculated. (C) Normalised TDM/TMM ratios in Mtb H37Ra cells treated with different compound concentrations and SQ109, a known MmpL3 inhibitor. The Assay was performed in biological replicates (*n* = 3), the ratio of TDM to TMM was normalised to a DMSO control. (D) Dose–response curves of Mtb H37Rv WT, MmpL3 over- (+ATc), and underexpressing (−ATc) strains dosed with 227 or SQ109. Figures each represent one of two biological replicates, each consisting of three technical replicates. (E) EphF activity of purified EphF. Protein was pre-treated with compound or DMSO before adding 9-10-*cis* epoxystearic acid. After 15 minutes, the reaction was quenched with chloroform, the stearic acids were extracted, and taurocholic acid was added as an internal standard. The resulting 9,10-dihydroxystearic acid was relatively quantified by LC-MS/MS. A heat control (HC) of heat-denatured EphF was included to monitor the background hydrolysis of the epoxide. Statistical significance of inhibition (compared to DMSO) was calculated using ordinary one-way ANOVA (* = *p* < 0.05; *** = *p* < 0.001; **** = *p* < 0.0001, *n* = 5). (F) Dose–response curves of Mtb H37Rv WT and 8 227-resistant mutants. The MICs and the mutations are illustrated in the table (G). The mutants carrying a mutation of V684 in *mmpL3* result in the biggest shift in MIC.

MmpL3 is a transporter essential for Mycobacterial cell wall biosynthesis. The activity of MmpL3 can be probed by feeding mycobacterial cultures with ^14^C-acetic acid and monitoring its incorporation into TMM and TDM *via* autoradiography (Fig. 2A and S5A†). If MmpL3 is blocked, TMM levels increase while TDM levels decline. The known MmpL3 inhibitor SQ109 was included as a positive control, reducing the ratio of TDM to TMM to 26% compared to the DMSO control at a concentration of 10 μM (Fig. 2B and C). Importantly, our hit-compound 227 reduced the ratio to 16% at 10 μM, validating this transporter as an antibiotic target. Newer 227 derivatives with improved MICs against Mtb H37Ra, 21 and 12 exhibited even lower TDM/TMM ratios (Fig. 2C). Moreover, we used the previously reported MmpL3 under and over-expressing Mtb H37Rv strains^22^ to determine MIC shifts with the compounds. SQ109 was used as a positive control and showed the expected higher susceptibility of MmpL3 under-expressing and lower susceptibility in over-expressing strains (Fig. 2D, S5C and D†). Ethambutol was included as a negative control and showed no significant shifts. Interestingly, our urea analogues, including 227, exhibited higher susceptibility only in under-expressing strains but no change in susceptibility when MmpL3 is over-expressed, suggesting a diverging MoA compared to SQ109, which may involve additional targets. SQ109 itself has been shown to have other targets apart from MmpL3,^41^ however, the drop in susceptibility in MmpL3-overexpressing strains indicates that MmpL3 inhibition seems to play a more important role for SQ109 than it does for 227.

Epoxide hydrolases, the third target class, could be linked to the MoA due to their role in mycolic acid metabolism. Although not essential to promote Mtb growth *in vitro*, EphD was shown to be essential for survival in macrophages and the biosynthesis of oxygenated mycolic acid species, important for maintaining the integrity of the cell envelope.^49,50^ To validate the target engagement of our urea compounds on EphD and EphF, we cloned, expressed, and purified the enzymes. Despite unsuccessful attempts to yield functional EphD, we successfully expressed functional EphF, allowing the establishment of an *in vitro* activity assay (Fig. 2E). We devised a new assay for mycobacterial Ephs that employs epoxystearic acid as a substrate and quantifies the enzymatic conversion from the epoxide to the diol using LC-MS. Heat controls with inactivated enzyme were included to assess background hydrolysis. 227 showed strong inhibition even at a mere 10-fold excess of compound compared to the enzyme (Fig. 2E). Compound 21 also inhibits EphF, albeit to a lesser extent.

**Fig. 3:**
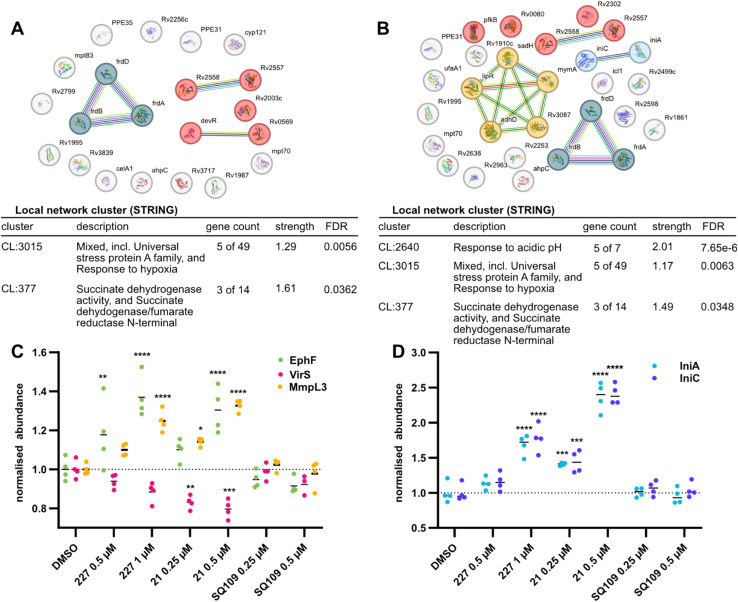
Comparative full-proteome analysis of 227, 21, and SQ109. (A and B) STRING GO-term analysis of Significantly dysregulated proteins (log_2_(fold change)) >1 or <−1, *p* < 0.01 of SQ109 treated cells (A) and 21 treated cells (B). For the analysis, the default STRING DB v.12.0 settings were used and the required interaction score was set to high confidence (>0.7). Both compounds induce a down-regulation of the clusters CL:3015 (red) and CL:377 (dark blue), while 21 additionally induces down-regulation of the entire *mymA* operon (CL:2640, yellow) and an up-regulation of isoniazid inducible genes *iniA* and *iniC* (light blue). (C) Profile plot of the normalised abundance of EphF, VirS, and MmpL3 across the different full proteome samples. ephF and MmpL3 are slightly upregulated in 227 and 21 treated cells, but not in SQ109 treated cells. VirS is slightly down-regulated in 21 treated cells, explaining the down-regulation of the whole mymA operon. (* = *p* < 0.05, ** = *p* < 0.01, **** = *p* < 0.0001 adjusted *p*-values, two-way ANOVA) (D) profile plot of the mean LFQ-values of, IniA and IniC across the different full proteome samples. Both proteins are concentration-dependently upregulated when cells are treated with 227 or 21, but not induced by SQ109 (**** = *p* < 0.0001, adjusted *p*-values, two-way ANOVA).

To further investigate additional targets of our novel urea compounds compared to SQ109, we performed comparative whole-cell proteomic MS studies with sub-lethal doses of SQ109, 227, and 21 in Mtb H37Ra (Fig. 3 and S6†). SQ109 is the most advanced MmpL3 inhibitor, having reached clinical phase 2b, and was therefore chosen as a positive control for our comparative analysis. There is a significant overlap of dysregulated proteins between the two urea compounds and SQ109, highlighting the fact that all three molecules target the same protein, MmpL3 (Fig. S6A–D†). All three compounds lead to a down-regulation of 3 subunits of the fumarate reductase complex (FrdA, FrdB, FrdD) (Fig. 3A and B). Previous studies have shown that impaired succinate oxidation attenuates the activity of cell wall inhibitors, including SQ109.^51^ Another cluster dysregulated by all three molecules is the two carbon starvation-inducible Proteins, Rv2557 and Rv2558, whose function is still unknown but has been linked to persistence.^52^ Interestingly, some notable differences between the urea compounds and SQ109 were observed. First, the proteins belonging to the *mymA* operon and its transcription factor VirS are down-regulated (Fig. 3B and C). The proteins of the *mymA* operon are required for appropriate mycolic acid composition of the cell wall and survival under acidic stress.^53^ MymA is required for the activation of the prodrug ethionamide, and its loss of function has been shown to result in ethionamide-resistant MTB.^54^ Therefore, we investigated whether compounds 21 or 227 have an antagonistic effect on the activity of ethionamide by conducting checkerboard assays. No significant antagonistic or synergistic effect could be observed, with fractional inhibitory concentration (FIC) index values^55,56^ of 0.83 and 1.33, respectively. Secondly, both 227 and 21 trigger the upregulation of the isoniazid inducible proteins IniA and IniC (Fig. 3D) which provide tolerance to various cell wall biosynthesis inhibitors.^57^ Previous studies did show that SQ109 induces the *iniBAC* operon, however these were conducted at much higher, lethal concentrations.^58^ Additionally, both 227 and 21 lead to upregulation of EphF (Fig. 3C), another indication that its inhibition plays a vital role in the mechanism of action of these novel compounds. Interestingly, both urea compounds induce a slight upregulation of the validated target MmpL3, while SQ109 does not (Fig. 3C).

To finally validate the protein targets of 227 on a genetic level, we generated resistant mutants of Mtb H37Rv at an estimated frequency of resistance of 1.5 × 10^−7^ at 4-fold the MIC. In total, eight resistant strains were isolated and sequenced (Fig. 2F and G). The most substantial shift was observed for strains carrying a mutation in *mmpl3* (9.5-fold MIC), followed by strains carrying mutations in *rv1254* (4.8-fold MIC), an uncharacterised acyl transferase essential for growth. Three strains had a distinct single point mutation in *rv1254* (L74R, L284P, A42T), and another two strains had a frame-shift in *rv1254*, which resulted in a C-terminal truncation, losing the last 31 or 33 amino acids. Given the lack of direct interaction with our tailored probes, or dysregulation in the full proteome analysis, and the unknown function of *rv1254*, in-depth studies into its role in the mode of action will be subject to future work. Importantly, three strains with the most pronounced resistance each carried a single point mutation of V684 in *mmpL3*, confirming MmpL3 as the main target of our new compound class. To confirm that our improved 227-derivatives exhibit the same mechanism of action, we measured the MICs of compounds 12 and 21 against mutants 1, 3, 5, and 7. The shifts of the MICs relative to WT MTB H37Rv are very similar, confirming the same mechanism of action (Table S3†).

Previously reported MmpL3 inhibitors such as SQ109, AU1235, and ICA-38 all share the same conserved binding pocket.^59^ Binding to this pocket blocks the proton relay pathway, rendering MmpL3 inactive.^59,60^ To test whether our novel compounds bind to the same pocket, we performed *in silico* docking experiments (using Autodock-Vina^61^) using compounds 227, 21, and the affinity-based probe 150-p and the co-crystal structure of MmpL3 with AU1235 (Fig. S7†). All three compounds achieved good docking scores, below −10 kcal mol^−1^. These results support the hypothesis that our novel compounds bind to the same pocket as other MmpL3 inhibitors. This conclusion is further supported by the resistance mutation V684G in MmpL3, which also confers resistance to indolcarboxamide compounds,^35^ with co-crystal structures confirming the same binding pocket.^59,60^ The exact mechanism by which this mutation generates resistance remains unclear but is hypothesised to indirectly influence the formation of the pocket.^62^

### Optimised 227-analogues are more potent, less hydrophobic and exhibit improved bioavailability

Many MmpL3 inhibitors suffer from high hydrophobicity (expressed in calculated log *P* values (*C* log *P*)) and, therefore, limited solubility. In fact, a *C* log *P* of 5.2 for 227 is insufficient, and a snapshot PK experiment in mice confirmed poor oral bioavailability probably due to limited solubility (Fig. S8A†). Intravenous (i.v.) administration also showed a fast clearance of 227, likely due to hepatic metabolsim. Thus, 227 needed a severe structural revision to become suitable for *in vivo* studies. We devised and synthesised over 50 compounds to obtain closer insights into the structure–activity relationship (SAR). We focused on introducing structural moieties that reduce the *C* log *P* and enhance solubility (Tables 1 and S2†). In the first series of 227 analogues, we varied substituents on the aromatic ring (1–8, 49–51); however, all compounds deviating from the 227-based trifluoromethyl and chlorine substitution showed no significantly improved or, in most cases, even strongly reduced antibiotic activity. We, therefore, focused on the aliphatic side for further derivatisation. We reduced the ring size from cyclohexyl over cyclopentyl, cyclobutyl to cyclopropyl (9–11). While for 3- and 4-membered rings, the activity decreased (MIC >25 μM), cyclopentyl derivative 9 remained active with a MIC of 3.2 μM. We systematically varied the aliphatic side by introducing methyl substituents, which could break planarity and influence the compound conformation. Interestingly, the incorporation of a methyl group either at 1, 2, 3, or 4 position (12–15) of the cyclohexyl ring significantly enhanced the antibiotic potency with the best MIC of 0.1 μM for compound 12 bearing the methyl group at the 1-position. We combined the two learnings to minimize hydrophobicity while improving the MIC and fused the methyl group in 1-position to the cyclopentyl ring (3). Compound 3 indeed showed a favorable MIC of 0.4 μM compared to the non-methylated derivative 9 (3.2 μM).

**Table 1:** Overview of novel 227-derived compounds. The remaining structures are listed in Table S2

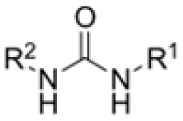				
Compound	*R* ^1^	*R* ^2^	*C* log *P*	MIC MTB H37Ra [μM]
227	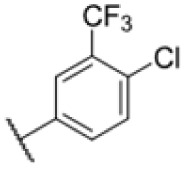	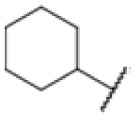	5.16	1.56–3.13
1	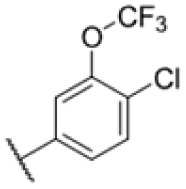	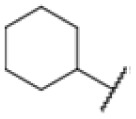	5.05	3.13
2	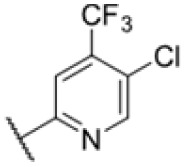	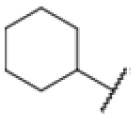	4.28	>50
3	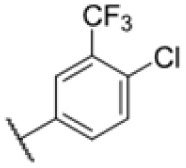	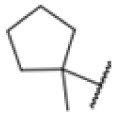	5.12	0.39
4	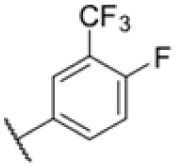	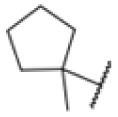	4.75	3.13
5	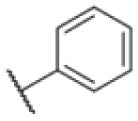	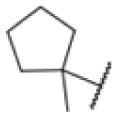	3.38	25
6	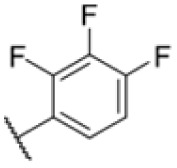	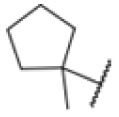	3.09	>50
7	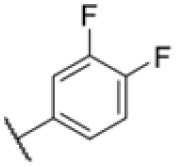	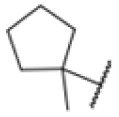	3.71	25
8	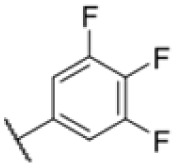	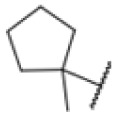	3.82	25
9	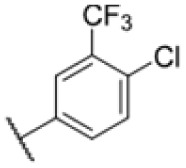	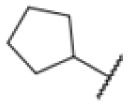	4.60	3.13
10	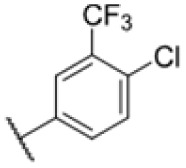	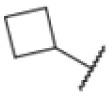	4.05	25
11	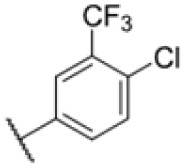	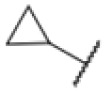	3.71	>50
12	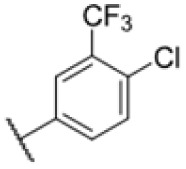	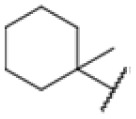	5.68	0.10
13	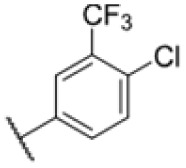	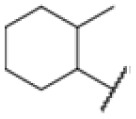	5.68	0.39
14	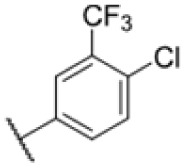	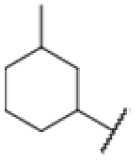	5.68	0.39
15	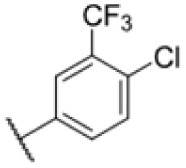	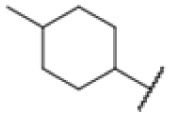	5.68	0.39
16	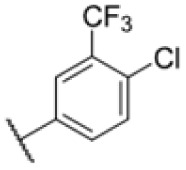	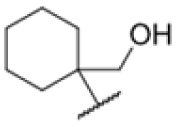	4.53	3.13
17	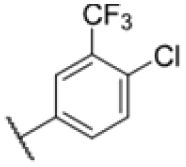	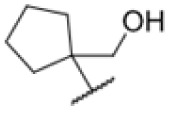	3.97	25
18	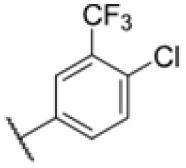	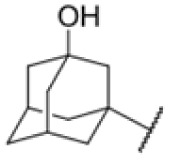	4.35	6.25
19	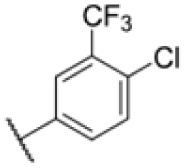	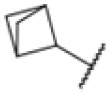	3.80	12.5
20	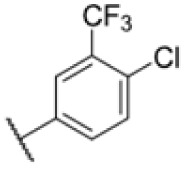	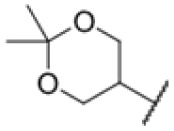	4.05	3.13
21	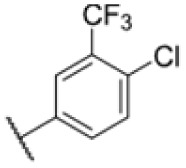	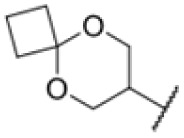	3.89	0.78
22	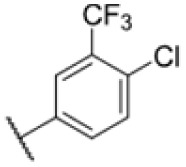	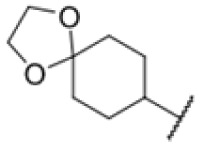	3.70	1.56–3.13
23	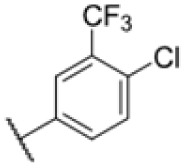	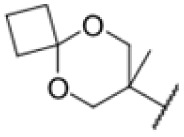	4.40	6.25–12.5

To further lower the hydrophobicity, we hydroxylated the methyl group at the 1-position (16, 17) and introduced different ligands bearing acetal groups and other hetero-atoms (18–22, 24–46). Here, compound 21, bearing a cyclobutyl acetal moiety, stood out with a MIC of 0.8 μM and an improved *C* log *P* of 3.9. Finally, we combined 21, the best compound with a low *C* log *P*, and 17, the C1-methylated cyclohexyl derivative with the best MIC 0.1 μM, to synthesize 23. However, this did not lead to an improved derivative as the MIC dropped to 6.25 μM, highlighting 21 as the most promising compound for *in vivo* studies. To confirm that the improved *C* log *P* values translate to improved solubility, we measured the kinetic solubility of the compounds in PBS (Table S4†). As expected, there is a strong correlation between the *C* log *P* values and the solubility of the compounds, with compound 21 exhibiting the highest solubility among the measured compounds with over 200 μM, compared to compound 227 at 55 μM. The mode of action of 21, namely MmpL3- and Eph-inhibition, was confirmed to be the same as its parent 227 (Fig. 2A, D, 3 and S6†).

Importantly, compounds 227, 21, and the most active compound, 12, showed no shift in MIC when testing against a bedaquiline-resistant clinical isolate (Fig. S8C†), displaying the potential use of these compounds to treat drug-resistant TB. Furthermore, compound 21 does not kill three important bacteria from the Oligo-Mouse-Microbiota (OMM^12^), an important characteristic for the typically long treatment times of TB infections (Fig. S8C†).

**Fig. 4:**
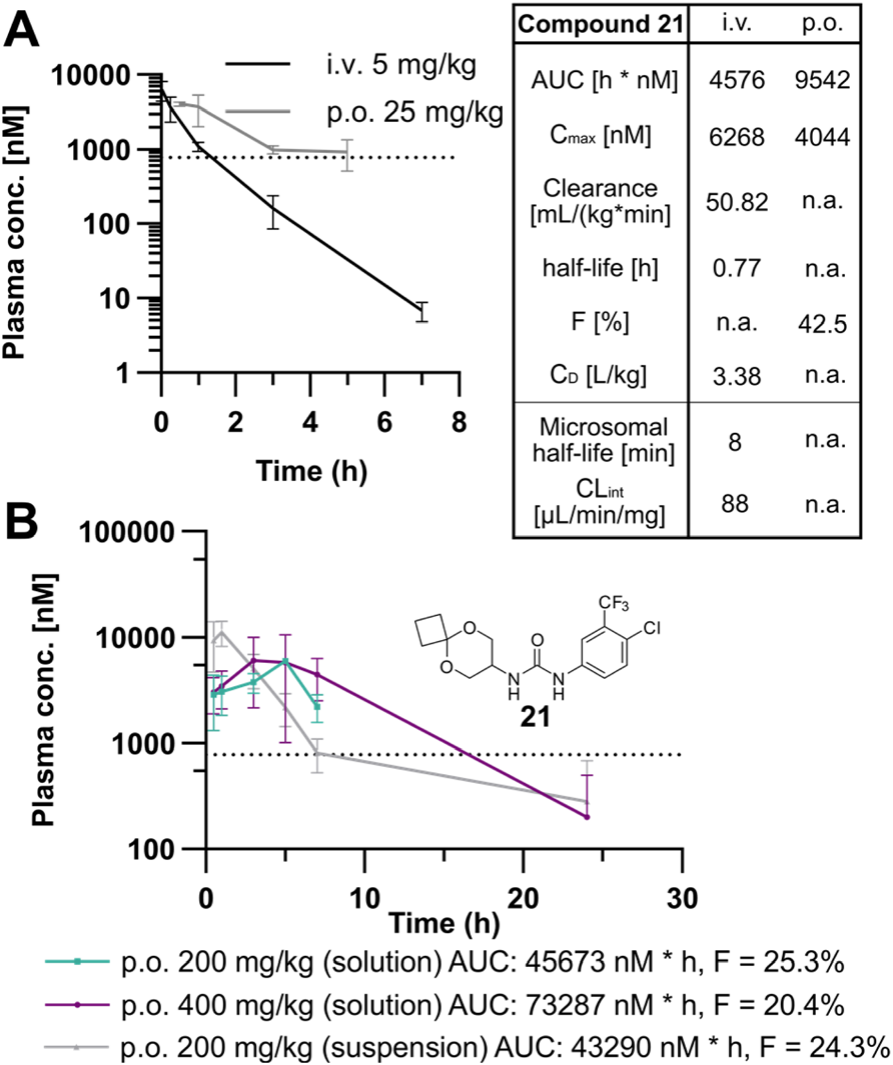
Pharmacokinetic profiling of 21. (A) Snapshot PK studies of compound 21. The compound was dosed orally at 25 mg kg^−1^ in 95% (20%) Solutol HS15, 5% DMA, or i.v. at 5 mg kg^−1^ in 4% Cremophor EL. The plasma concentration was measured over time (p.o. *n* = 2, i.v. *n* = 3). The dotted line indicates the MIC against MTB H37Ra. PK properties and microsomal stability data are listed in the table on the right of the graph. (B) In-depth oral PK study of compound 21. Compounds were dosed orally in 100% PEG400 (solution) or 20% Solutol HS15 (suspension) three days in a row, and blood was taken after the third dose (*n* = 3). The compound was well tolerated, and there was no weight loss, sickness, or necrosis.

As a result of the structural optimization, we identified compounds 21 and 22 to have promising activity profiles to advance for *in vivo* studies. Of note, 21 has 2 to 4-fold better MIC than the parent 227 (0.78 μM compared to 1.56–3.13 μM). To select the best derivative for *in vivo* applications, we performed snapshot PK studies with both compounds and compare them to 227. All snapshot PK studies were conducted at 5 mg per kg i.v. and 25 mg per kg p.o. We obtained significantly improved oral exposure with both molecules exhibiting plasma concentrations above the MIC (Fig. 4A and S8B†). Intravenous dosing revealed that the clearance rate could not be improved compared to 227, and a microsomal stability assay indicates that metabolisation seems to be the driver of clearance (Fig. 4B). However, the bioavailability of 21 (*F* = 42.5%) was vastly improved compared to 227 (*F* = 8.6%) (Fig. 4A and S8A†). As 21 has a sub-micromolar MIC, achieved prolonged plasma levels above MIC, and displayed no pronounced cytotoxicity against human cells in a biologically relevant range (IC_50_ = 71 μM) in MTT assays (Fig. S8C†), it was selected for more in-depth oral dosing studies (Fig. 4B). For this, the drug was dosed at concentrations more relevant for future efficacy studies (200 mg kg^−1^, 400 mg kg^−1^) for three consecutive days using both a polyethylene glycol (PEG400) based solution and a 20% solutol HS15 based suspension. The PK analysis was performed at steady-state on day three. The compound was well tolerated throughout the three days of dosing. The solution formulation led to similar oral exposure compared to a suspension formulation, demonstrating that solubility is not contributing to higher bioavailability. Dosing in the PEG400-based formulations demonstrated an estimated time above MIC of approximately 16 hours for both the 200 and 400 mg kg^−1^ dosing indicating potentially therapeutic levels. Overall, the significantly improved bioavailability and reduced toxicity of 21 compared to the initial hit 227 make it a promising starting point for further structural optimisation to address metabolic stability focused on examining the metabolisation of the acetal moiety.

## Conclusions

Finding drugs with novel molecular targets is crucial for the continued fight against a rise in drug-resistant M*tb*. In this study, we applied tailored affinity-based probes to identify the molecular targets of a novel hit compound (227) and validated these using cellular assays and resistance generation. Of note, we provide strong evidence for MmpL3 as the primary target *via* two independent methods: chemical proteomics and sequencing of resistant strains. The strains with the highest resistance to 227 all carried a single-point mutation of V684 in *mmpL3*. This mutation has been reported for strains resistant to indolecarboxamides,^35,63^ which indicates a similar binding mode or a conserved resistance mechanism. While the activity assay and sequencing of the resistant mutants validated MmpL3 as a target, overexpression of MmpL3 in Mtb did not result in reduced susceptibility. Although some reports also indicate no change in susceptibility to MmpL3 inhibitors in MmpL3-overexpressing *M. smegmatis* and *M. abscessus* strains,^20,64^ this nonetheless could suggest a polypharmacological mode of action. In this context, the unbiased nature of chemical proteomics is ideally suited to identify multiple protein targets of new drugs. Affinity-based protein profiling with a refined probe identified two epoxide hydrolases as additional high-confidence targets. Although both proteins are not essential for growth *in vitro*, they are essential for survival in macrophages, which could translate to improved *in vivo* efficacy. EphD plays a role in the biosynthesis of oxygenated mycolic acid species and the integrity of the cell envelope. The exact role of EphF is still elusive, but due to the similar *in vitro* substrate specificity, a role in mycolic acid biosynthesis is also likely. While we could not express active EphD, we could show concentration-dependent inhibition of EphF. These results indicate a dual mode of action on the mycolic acid biosynthesis in which the synthesis of some mycolic acid species is impaired through the inhibition of epoxide hydrolases and by blocking the essential export of TMM across the membrane by inhibiting MmpL3. The essential role of the epoxide hydrolases for growth during infection could prove advantageous in future efficacy studies and may limit resistance generation compared to *in vitro* experiments.

Based on the initial hit 227, an extensive SAR study was conducted. The aim was to improve the activity while lowering the hydrophobicity. We achieved both goals by introducing acetals on the non-aromatic side of the urea compounds. Compound 21 has a significantly lower *C* log *P* (3.89) while exhibiting a sub-micromolar MIC. The lowered hydrophobicity resulted in significantly improved oral bioavailability, even with non-optimised formulations. In contrast, known MmpL3 inhibitors are significantly more hydrophobic and thus require extensive formulation studies and optimisation to achieve satisfactory bioavailability.^35^

## Data availability

The mass spectrometry proteomics data have been deposited to the ProteomeXchange Consortium *via* the PRIDE partner repository^65^ with the identifier PXD050993.

## Author contributions

DM and SAS planned the project and all experiments. JB synthesised all previously unpublished affinity-based probes and all derivatives in the SAR study. DM performed all proteomic and validation experiments included in this work. Susceptibility testing in *mmpL3* TetON strains and generation of 227-resistant mutants was performed by CAE, JBW and DS. PK studies were performed by MDZ, JC and SPL and VD. The ethionamide checkerboard assay was performed by SB. Molecular Docking studies were performed by PQ and MZ. Kinetic solubility studies were conducted by MAK and AKH. DM and SAS prepared the manuscript for publication and DM created all figures.

## Conflicts of interest

S .A. S. is cofounder of smartbax limited.

## Supplementary Material

SC-016-D5SC02565F-s001

SC-016-D5SC02565F-s002

## References

[cit1] Cassini A., Högberg L. D., Plachouras D., Quattrocchi A., Hoxha A., Simonsen G. S., Colomb-Cotinat M., Kretzschmar M. E., Devleesschauwer B., Cecchini M., Ouakrim D. A., Oliveira T. C., Struelens M. J., Suetens C., Monnet D. L., Strauss R., Mertens K., Struyf T., Catry B., Latour K., Ivanov I. N., Dobreva E. G., Tambic Andraševic A., Soprek S., Budimir A., Paphitou N., Žemlicková H., Schytte Olsen S., Wolff Sönksen U., Märtin P., Ivanova M., Lyytikäinen O., Jalava J., Coignard B., Eckmanns T., Abu Sin M., Haller S., Daikos G. L., Gikas A., Tsiodras S., Kontopidou F., Tóth Á., Hajdu Á., Guólaugsson Ó., Kristinsson K. G., Murchan S., Burns K., Pezzotti P., Gagliotti C., Dumpis U., Liuimiene A., Perrin M., Borg M. A., de Greeff S. C., Monen J. C., Koek M. B., Elstrøm P., Zabicka D., Deptula A., Hryniewicz W., Caniça M., Nogueira P. J., Fernandes P. A., Manageiro V., Popescu G. A., Serban R. I., Schréterová E., Litvová S., Štefkovicová M., Kolman J., Klavs I., Korošec A., Aracil B., Asensio A., Pérez-Vázquez M., Billström H., Larsson S., Reilly J. S., Johnson A., Hopkins S. (2019). Lancet Infect. Dis..

[cit2] Zhen X., Stålsby Lundborg C., Sun X., Zhu N., Gu S., Dong H. (2021). Antimicrob. Resist. Infect. Control.

[cit3] Bagcchi S. (2023). Lancet Microbe.

[cit4] Craggs P. D., de Carvalho L. P. S. (2022). Curr. Opin. Microbiol..

[cit5] Fernandes G. F. S., Thompson A. M., Castagnolo D., Denny W. A., Dos Santos J. L. (2022). J. Med. Chem..

[cit6] World Health Organisation , What’s new in the TB section of the 2023 WHO Model Lists of Essential Medicines, 2023, https://www.who.int/news/item/09-08-2023-what-s-new-in-the-tb-section-of-the-2023-who-model-lists-of-essential-medicines

[cit7] Ryan N. J., Lo J. H. (2014). Drugs.

[cit8] Keam S. J. (2019). Drugs.

[cit9] Dartois V., Dick T. (2024). Nat. Rev. Drug Discovery.

[cit10] Nguyen T. V. A., Anthony R. M., Bañuls A. L., Vu D. H., Alffenaar J. W. C. (2018). Clin. Infect. Dis..

[cit11] World Health Organisation , Rapid Communication: Key changes to the treatment of drug-resistant tuberculosis, 2022, https://www.who.int/publications/i/item/WHO-UCN-TB-2022-2

[cit12] Van Rie A., Walker T., de Jong B., Rupasinghe P., Rivière E., Dartois V., Sonnenkalb L., Machado D., Gagneux S., Supply P., Dreyer V., Niemann S., Goig G., Meehan C., Tagliani E., Cirillo D. M. (2022). Lancet Infect. Dis..

[cit13] Sonnenkalb L., Carter J. J., Spitaleri A., Iqbal Z., Hunt M., Malone K. M., Utpatel C., Cirillo D. M., Rodrigues C., Nilgiriwala K. S., Fowler P. W., Merker M., Niemann S., Barilar I., Battaglia S., Borroni E., Brandao A. P., Brankin A., Cabibbe A. M., Carter J., Claxton P., Clifton D. A., Cohen T., Coronel J., Crook D. W., Dreyer V., Earle S. G., Escuyer V., Ferrazoli L., Fowler P. W., Fu Gao G., Gardy J., Gharbia S., Ghisi K. T., Ghodousi A., Gibertoni Cruz A. L., Grandjean L., Grazian C., Groenheit R., Guthrie J. L., He W., Hoffmann H., Hoosdally S. J., Ismail N. A., Jarrett L., Joseph L., Jou R., Kambli P., Khot R., Knaggs J., Koch A., Kohlerschmidt D., Kouchaki S., Lachapelle A. S., Lalvani A., Grandjean Lapierre S., Laurenson I. F., Letcher B., Lin W. H., Liu C., Liu D., Malone K. M., Mandal A., Mansjö M., Matias D., Meintjes G., de Freitas Mendes F., Mihalic M., Millard J., Miotto P., Mistry N., Moore D., Musser K. A., Ngcamu D., Hoang N. N., Nimmo C., Okozi N., Oliveira R. S., Omar S. V., Paton N., Peto T. E., Watanabe Pinhata J. M., Plesnik S., Puyen Z. M., Rabodoarivelo M. S., Rakotosamimanana N., Rancoita P. M., Rathod P., Rodger G., Rodwell T. C., Roohi E., Santos-Lazaro D., Shah S., Kohl T. A., Smith G., Solano W., Supply P., Surve U., Tahseen S., Thuong N. T. T., Thwaites G., Todt K., Trovato A., Van Rie A., Vijay S., Walker T. M., Walker S. A., Warren R., Werngren J., Wijkander M., Wilkinson R. J., Wilson D. J., Wintringer P., Yu X. X., Yang Y., Zhao Y., Yao S. Y., Zhu B. (2023). Lancet Microbe.

[cit14] Timmins G. S., Deretic V. (2006). Mol. Microbiol..

[cit15] Shetye G. S., Franzblau S. G., Cho S. (2020). Transl. Res..

[cit16] Aggarwal A., Parai M. K., Shetty N., Wallis D., Woolhiser L., Hastings C., Dutta N. K., Galaviz S., Dhakal R. C., Shrestha R., Wakabayashi S., Walpole C., Matthews D., Floyd D., Scullion P., Riley J., Epemolu O., Norval S., Snavely T., Robertson G. T., Rubin E. J., Ioerger T. R., Sirgel F. A., van der Merwe R., van Helden P. D., Keller P., Böttger E. C., Karakousis P. C., Lenaerts A. J., Sacchettini J. C. (2017). Cell.

[cit17] Shao M., McNeil M., Cook G. M., Lu X. (2020). Eur. J. Med. Chem..

[cit18] Umare M. D., Khedekar P. B., Chikhale R. V. (2021). ChemMedChem.

[cit19] Williams J. T., Abramovitch R. B. (2023). Microb. Drug Resist..

[cit20] Grzegorzewicz A. E., Pham H., Gundi V. A. K. B., Scherman M. S., North E. J., Hess T., Jones V., Gruppo V., Born S. E. M., Korduláková J., Chavadi S. S., Morisseau C., Lenaerts A. J., Lee R. E., McNeil M. R., Jackson M. (2012). Nat. Chem. Biol..

[cit21] Tahlan K., Wilson R., Kastrinsky D. B., Arora K., Nair V., Fischer E., Barnes S. W., Walker J. R., Alland D., Barry C. E., Boshoff H. I. (2012). Antimicrob. Agents Chemother..

[cit22] Grover S., Engelhart C. A., Pérez-Herrán E., Li W., Abrahams K. A., Papavinasasundaram K., Bean J. M., Sassetti C. M., Mendoza-Losana A., Besra G. S., Jackson M., Schnappinger D. (2021). ACS Infect. Dis..

[cit23] Pandya A. N., Prathipati P. K., Hegde P., Li W., Graham K. F., Mandal S., Drescher K. M., Destache C. J., Ordway D., Jackson M., Northa E. J. (2019). Antimicrob. Agents Chemother..

[cit24] Kozikowski A. P., Onajole O. K., Stec J., Dupont C., Viljoen A., Richard M., Chaira T., Lun S., Bishai W., Raj V. S., Ordway D., Kremer L. (2017). J. Med. Chem..

[cit25] Hu T., Yang X., Liu F., Sun S., Xiong Z., Liang J., Yang X., Wang H., Yang X., Guddat L. W., Yang H., Rao Z., Zhang B. (2022). Structure.

[cit26] Harth G., Lee B. Y., Wang J., Clemens D. L., Horwitz M. A. (1996). Infect. Immun..

[cit27] Sanki A. K., Boucau J., Ronning D. R., Sucheck S. J. (2009). Glycoconj. J..

[cit28] Belisle J. T., Vissa V. D., Sievert T., Takayama K., Brennan P. J., Besra G. S. (1997). Science.

[cit29] Ronning D. M., Vissa V., Besra G. S., Belisle J. T., Sacchettini J. C. (2004). J. Biol. Chem..

[cit30] Favrot L., Grzegorzewicz A. E., Lajiness D. H., Marvin R. K., Boucau J., Isailovic D., Jackson M., Ronning D. R. (2013). Nat. Commun..

[cit31] Xu Z., Meshcheryakov V. A., Poce G., Chng S. S. (2017). Proc. Natl. Acad. Sci. U. S. A..

[cit32] Li W., Upadhyay A., Fontes F. L., North E. J., Wang Y., Crans D. C., Grzegorzewicz A. E., Jones V., Franzblau S. G., Lee R. E., Crick D. C., Jackson M. (2014). Antimicrob. Agents Chemother..

[cit33] Imran M., Arora M. K., Chaudhary A., Khan S. A., Kamal M., Alshammari M. M., Alharbi R. M., Althomali N. A., Alzimam I. M., Alshammari A. A., Alharbi B. H., Alshengeti A., Alsaleh A. A., Alqahtani S. A., Rabaan A. A. (2022). Biomedicines.

[cit34] Williams J. T., Haiderer E. R., Coulson G. B., Conner K. N., Ellsworth E., Chen C., Alvarez-Cabrera N., Li W., Jackson M., Dick T., Abramovitch R. B. (2019). Antimicrob. Agents Chemother..

[cit35] Rao S. P. S., Lakshminarayana S. B., Kondreddi R. R., Herve M., Camacho L. R., Bifani P., Kalapala S. K., Jiricek J., Ma N. L., Tan B. H., Ng S. H., Nanjundappa M., Ravindran S., Seah P. G., Thayalan P., Lim S. H., Lee B. H., Goh A., Barnes W. S., Chen Z., Gagaring K., Chatterjee A. K., Pethe K., Kuhen K., Walker J., Feng G., Babu S., Zhang L., Blasco F., Beer D., Weaver M., Dartois V., Glynne R., Dick T., Smith P. W., Diagana T. T., Manjunatha U. H. (2013). Sci. Transl. Med..

[cit36] Graham J., Wong C. E., Day J., McFaddin E., Ochsner U., Hoang T., Young C. L., Ribble W., DeGroote M. A., Jarvis T., Sun X. (2018). Bioorg. Med. Chem. Lett..

[cit37] Raynaud C., Daher W., Johansen M. D., Roquet-Banères F., Blaise M., Onajole O. K., Kozikowski A. P., Herrmann J. L., Dziadek J., Gobis K., Kremer L. (220). ACS Infect. Dis..

[cit38] La Rosa V., Poce G., Canseco J. O., Buroni S., Pasca M. R., Biava M., Raju R. M., Porretta G. C., Alfonso S., Battilocchio C., Javid B., Sorrentino F., Ioerger T. R., Sacchettini J. C., Manetti F., Botta M., De Logu A., Rubin E. J., De Rossi E. (2012). Antimicrob. Agents Chemother..

[cit39] Zhang B., Li J., Yang X., Wu L., Zhang J., Yang Y., Zhao Y., Zhang L., Yang X., Yang X., Cheng X., Liu Z., Jiang B., Jiang H., Guddat L. W., Yang H., Rao Z. (2019). Cell.

[cit40] Li W., Stevens C. M., Pandya A. N., Darzynkiewicz Z., Bhattarai P., Tong W., Gonzalez-Juarrero M., North E. J., Zgurskaya H. I., Jackson M. (2019). ACS Infect. Dis..

[cit41] Li K., Schurig-Briccio L. A., Feng X., Upadhyay A., Pujari V., Lechartier B., Fontes F. L., Yang H., Rao G., Zhu W., Gulati A., No J. H., Cintra G., Bogue S., Liu Y. L., Molohon K., Orlean P., Mitchell D. A., Freitas-Junior L., Ren F., Sun H., Jiang T., Li Y., Guo R. T., Cole S. T., Gennis R. B., Crick D. C., Oldfield E. (2014). J. Med. Chem..

[cit42] Madacki J., Kopál M., Jackson M., Korduláková J. (2021). Int. J. Mol. Sci..

[cit43] Le P., Kunold E., Macsics R., Rox K., Jennings M. C., Ugur I., Reinecke M., Chaves-Moreno D., Hackl M. W., Fetzer C., Mandl F. A. M., Lehmann J., Korotkov V. S., Hacker S. M., Kuster B., Antes I., Pieper D. H., Rohde M., Wuest W. M., Medina E., Sieber S. A. (2020). Nat. Chem..

[cit44] Fonović M., Bogyo M. (2008). Expert Rev. Proteomics.

[cit45] Evans M. J., Cravatt B. F. (2006). Chem. Rev..

[cit46] Rostovtsev V. V., Green L. G., Fokin V. V., Sharpless K. B. (2002). Angew. Chem. Int. Ed..

[cit47] Tornøe C. W., Christensen C., Meldal M. (2002). J. Org. Chem..

[cit48] Cox J., Hein M. Y., Luber C. A., Paron I., Nagaraj N., Mann M. (2014). Mol. Cell. Proteomics.

[cit49] Madacki J., Laval F., Grzegorzewicz A., Lemassu A., Záhorszká M., Arand M., McNeil M., Daffé M., Jackson M., Lanéelle M., Korduláková J. (2018). J. Biol. Chem..

[cit50] Rengarajan J., Bloom B. R., Rubin E. J. (2005). Proc. Natl. Acad. Sci. U. S. A..

[cit51] Adolph C., McNeil M. B., Cook G. M. (2022). mBio.

[cit52] Gordhan B. G., Smith D. A., Kana B. D., Bancroft G., Mizrahi V. (2006). Tuberculosis.

[cit53] Singh A., Gupta R., Vishwakarma R. A., Narayanan P. R., Paramasivan C. N., Ramanathan V. D., Tyagi A. K. (2022). J. Bacteriol..

[cit54] Grant S. S., Wellington S., Kawate T., Earl A. M., Fitzgerald M., Hung D. T., Grant S. S., Wellington S., Kawate T., Desjardins C. A., Haseley N., Iwase N., Earl A. M., Fitzgerald M., Hung D. T. (2016). Cell Chem. Biol..

[cit55] Meletiadis J. , Pournaras S., Roilides E. and Walsh T. J., 2010, 54, 602 609 10.1128/AAC.00999-09 PMC2812160 19995928

[cit56] Emorypharma , Antimicrobial Synergy Study – Checkerboard Testing, https://emerypharma.com/solutions/cell-microbiology-services/antimicrobial-synergy-study-checkerboard-testing/

[cit57] Alland D., Steyn A. J., Weisbrod T., Aldrich K., Jacobs W. R. (2000). J. Bacteriol..

[cit58] Lee R. E., Protopopova M., Crooks E., Slayden R. A., Terrot M., Barry C. E. (2003). J. Comb. Chem..

[cit59] Zhang B., Li J., Yang X., Wu L., Zhang J., Yang Y., Zhao Y., Zhang L., Yang X., Yang X., Cheng X., Liu Z., Jiang B., Jiang H., Guddat L. W., Yang H., Rao Z. (2019). Cell.

[cit60] Yang X., Hu T., Yang X., Xu W., Yang H., Guddat L. W., Zhang B., Rao Z. (2020). J. Mol. Biol..

[cit61] Eberhardt J., Santos-Martins D., Tillack A. F., Forli S. (2021). J. Chem. Inf. Model..

[cit62] Yang X., Hu T., Yang X., Xu W., Yang H., Guddat L. W., Zhang B., Rao Z. (2020). J. Mol. Biol..

[cit63] Stanley S. A., Grant S. S., Kawate T., Iwase N., Shimizu M., Wivagg C., Silvis M., Kazyanskaya E., Aquadro J., Golas A., Fitzgerald M., Dai H., Zhang L., Hung D. T. (2012). ACS Chem. Biol..

[cit64] Dupont C., Viljoen A., Dubar F., Blaise M., Bernut A., Pawlik A., Bouchier C., Brosch R., Guérardel Y., Lelièvre J., Ballell L., Herrmann J. L., Biot C., Kremer L. (2016). Mol. Microbiol..

[cit65] Perez-Riverol Y., Bai J., Bandla C., García-Seisdedos D., Hewapathirana S., Kamatchinathan S., Kundu D. J., Prakash A., Frericks-Zipper A., Eisenacher M., Walzer M., Wang S., Brazma A., Vizcaíno J. A. (2022). Nucleic Acids Res..

