# Supplementary material for: Tailored phenyl ureas eradicate drug-resistant Mycobacterium tuberculosis by targeting mycolic acid cell wall assembly
Source: Chem Sci. 2025 Apr 30;16(21):9472–83. doi: 10.1039/d5sc02565f (PMC12041881; doi:10.1039/d5sc02565f)
Supplement: SC-016-D5SC02565F-s002 [file SC-016-D5SC02565F-s002.pdf]

Supplementary Information (SI) for Chemical Science.
This journal is © The Royal Society of Chemistry 2025

Supplementary Information

Tailored phenyl ureas eradicate drug-resistant Mycobacterium tuberculosis by targeting mycolic acid cell wall assembly

Dietrich Mostert, [†a] Josef Braun, [†a] Matthew D. Zimmerman, [b] Curtis A. Engelhart, [c] Sara Berndl, [a] Patrick K. Quoika, [a] Andreas M. Kany, [de] Julianna Proietto, [b] Suyapa Penalva-Lopez, [b] Joshua B. Wallach, [c] Anna K. H. Hirsch, [def] Martin Zacharias, [a] Dirk Schnappinger, [c] Véronique Dartois, [b] and Stephan A. Sieber *[a]

[a] Center for Functional Protein Assemblies, Department of Bioscience, TUM School of Natural Sciences, Technical University of Munich, Ernst-Otto-Fischer-Straße 8, 85748 Garching, Germany. E-mail: stephan.sieber@tum.de
[b] Center for Discovery and Innovation, Hackensack Meridian Health, Nutley, New Jersey, USA
[c] Department of Microbiology and Immunology, Weill Cornell Medical College, New York, NY, USA
[d] Helmholtz Institute for Pharmaceutical Research Saarland (HIPS) – Helmholtz Centre for Infection Research (HZI), Campus E8.1, 66123 Saarbrücken, Germany
[e] Deutsches Zentrum für Infektionsforschung (DZIF) e.V., 38124 Braunschweig, Germany
[f] Saarland University, Department of Pharmacy, 66123 Saarbrücken, Germany

[†] These authors contributed equally.

Table of Content

Supplementary Figures.......... 3

Methods 16

Biochemical Methods.......... 16

Mycobacterial culture 16

Minimal inhibitory concentration measurements in *M. tuberculosis*.......... 16

Minimal inhibitory concentration measurements in strains from the Oligo-Mouse-Microbiota. 16

Kinetic Turbidimetric Solubility 17

Gel-based fluorescent labelling in Mycobacteria.......... 17

Preparative labelling in *M. smegmatis* using probe 150-p for Mass spectrometry.......... 18

Preparative labelling in *M. tuberculosis* for Mass spectrometry 20

Full Proteome analysis in M*tb* H37Ra 21

LC-MS measurements and data analysis timsTOF Pro samples.......... 22

Membrane integrity assay in *M. tuberculosis* H37Ra.......... 24

Measuring membrane bound LepB activity 25

TDM/TMM autoradiography assay (MmpL3).......... 26

Expression and purification of Mtb epoxide hydrolases EphF and EphD.......... 26

EphF activity assay.......... 27

MTT Cytotoxicity Assay 28

Drug susceptibility testing in MmpL3 under/over-expressing *M. tuberculosis* 29

Isolation of 227-resistant mutants 29

Pharmacokinetics studies.......... 29

LC-MS/MS analytical methods for PK studies 30

Metabolic stability in mouse liver microsomes (MLM).......... 30

Molecular Docking MmpL3 31

Chemical Synthesis.......... 32

General synthesis methods and materials.......... 32

Synthesis of compounds in order of appearance.......... 33

NMR Spectra.......... 67

References.......... 128

Supplementary Figures

Table S1. Significant hits from volcano plot in figure 1D. *M. smegmatis* cells treated with 12.5 µM **150-p** compared to **150-pi**.

Uniprot ID	Gene name	Protein Description	Log_2 fold-change	Essential ortholog in MTB? (Mycobrowser)
A0QV43	lepB	Signal peptidase I	3.23	**yes**[1–4]
A0QTI3	MSMEG_1855	Tetratricopeptide repeat protein, putative	2.72	no
A0R5I8	MSMEG_6207	Polyketide cyclase / dehydrase and lipid transport	2.59	no
A0R2U1	MSMEG_5233	RmlD substrate binding domain superfamily protein	2.45	no
A0R292	MSMEG_5030	Deazaflavin-dependent nitroreductase family protein	2.32	no
A0R5R8	MSMEG_6288	Transmembrane protein	2.19	no
A0QSW8	MSMEG_1629	Uncharacterized protein	2.17	no
A0R1J8	MSMEG_4779	Probable regulatory protein	2.13	no
A0QWJ1	yajC	Preprotein translocase, YajC subunit	1.99	no
Q3I5Q7	MSMEG_0919	HBHA-like protein, Heparin-binding hemagglutinin	1.93	no
A0R0Z5	MSMEG_4563	Puromycin N-acetyltransferase	1.72	no
A0QTV7	MSMEG_1981	Deazaflavin-dependent nitroreductase family protein	1.60	no
A0R2T3	MSMEG_5225	Lipid droplet-associated protein	1.59	no
A0R2B0	MSMEG_5048	EcsC protein family protein	1.54	no
A0QRA8	MSMEG_1049	Methyltransferase	1.53	no
A0QQB0	MSMEG_0690	Iron-sulfur cluster-binding protein	1.39	no
A0QNZ7	MSMEG_0220	Monoacylglycerol lipase	1.27	no
A0QP27	mmpL3	Trehalose monomycolate exporter MmpL3	1.18	**yes**[1–4]
A0R5G6	MSMEG_6183	Serine protease	1.06	unclear[1,2,4,5]

Table S2. Overview of 227-derivatives, not shown in Table 1

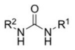

Compound	R[1]	R[2]	CLogP	MIC MTB H37Ra [μM]
24	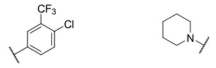		3.93	50
25	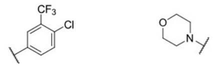		2.68	> 50
26	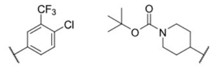		4.68	25
27	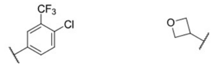		3.17	> 50
28	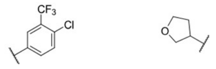		3.10	> 50
29	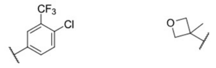		3.69	> 50
30	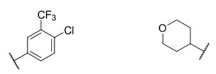		2.76	> 50
31	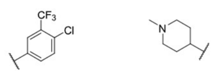		3.19	> 50
32	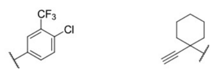		5.57	0.39
33	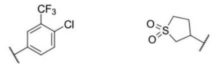		2.61	> 50
34	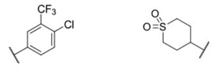		2.62	> 50
35	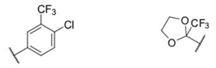		5.27	25
36	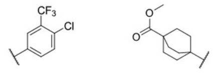		4.34	> 50
37	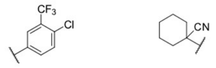		4.39	> 50
38	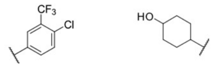		3.07	> 50
39	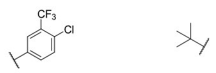		4.37	12.5 - 25
40	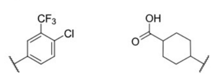		3.65	>50
41	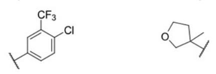		3.62	25
42	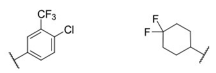		4.55	6.25
43	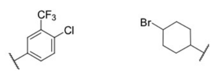		5.14	0.78
44	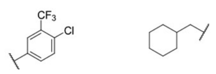		5.78	1.56 - 3.125

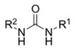

Compound	R[1]	R[2]	CLogP	MIC MTB H37Ra [μM]
45	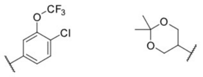		3.94	50
46	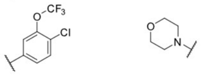		2.938	> 50
47	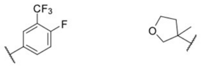		3.25	25
48	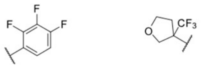		2.40	> 50
49	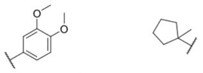		2.88	> 50
50	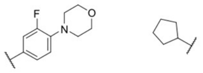		2.53	> 50
51	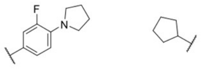		3.35	> 50

Compound	Structure	CLogP	MIC MTB H37Ra [μM]
52	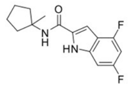	3.87	3.125

Table S3. MICs of compounds **227**, **12**, and **21** against **227**-resistant mutants of *M. tuberculosis* H37rv

Strain	Mutation	Compound 227		Compound 12		Compound 21	
		IC50 (µM)	MIC90 (µM)	IC50 (µM)	MIC90 (µM)	IC50 (µM)	MIC90 (µM)
**WT H37Rv**		1.3	2.5	0.11	0.2	0.85	1.8
**Mutant #1**	***mmpl3*-V684G**	9.7	18	0.83	1.5	7.1	12
**Mutant #3**	***rv1254*-L74R**	3.3	6.7	0.12	0.28	1.3	3.1
**Mutant #5**	***mmpl3*-V684G**	6.9	12	0.66	1.3	8.5	18
**Mutant #7**	***rv1254*-L284P**	3.3	5.7	0.14	0.29	1.5	3.3

Table S4. Kinetic solubility of selected compounds in PBS (n=3)

Kinetic solubility			
**Compound**	**[µM]**	**SD**	**CLogP**
**227**	**55**	13	5.16
**12**	**12**	5	5.68
**21**	**>200**	0	3.89
**22**	**112**	19	3.70
**23**	**58**	4	4.40

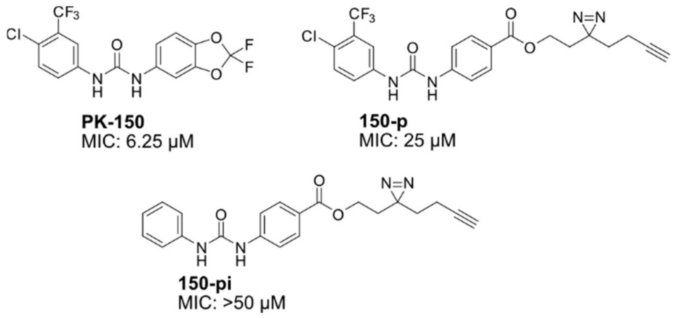

Figure S1. Structures of PK150 and the probes derived from its structure. Minimal inhibitory concentrations (MICs) in *M. tuberculosis* H37Ra show that **150-p** is active, while **150-pi** is inactive.

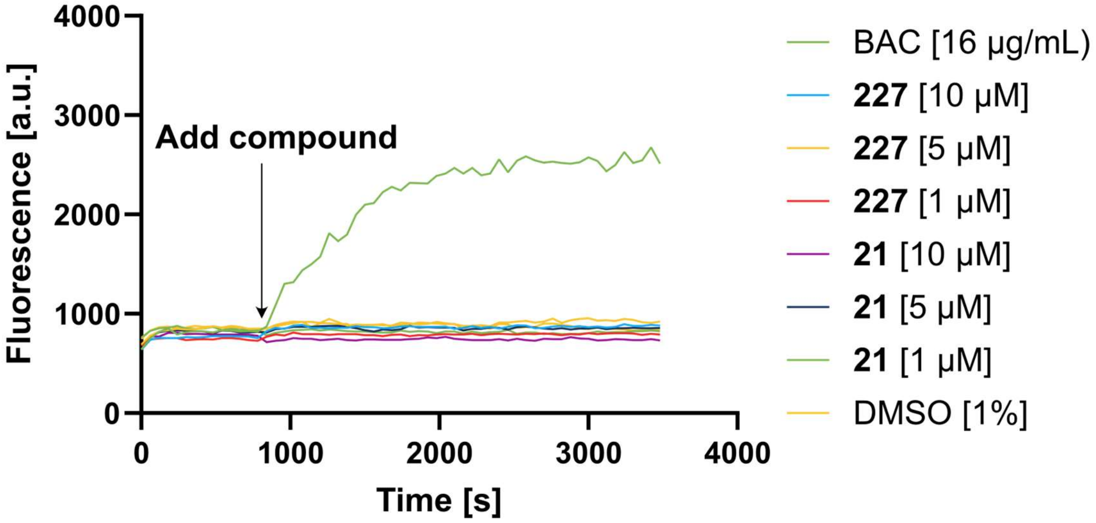

Figure S2. Membrane integrity assay. *M. tuberculosis* H37Ra cells were incubated with propidium iodide for 12 minutes before adding the indicated compounds. The Detergent benzalkonium chloride (BAC) was used as a positive control. Neither **227** nor **21** disrupt the membrane to allow propidium iodide to enter the cells. The experiment was conducted in triplicates, and the mean (n=3) was plotted.

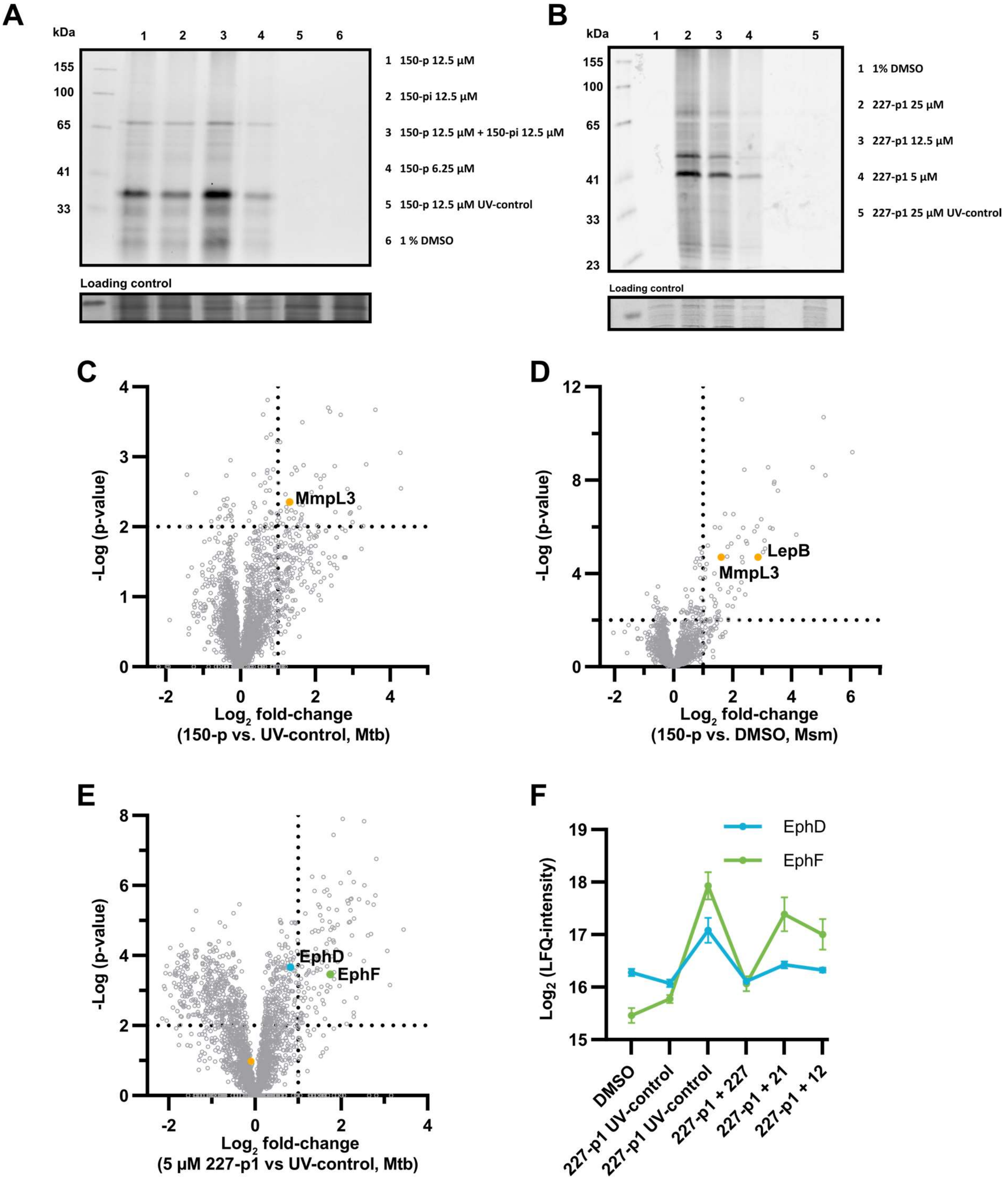

Figure S3. AfBPP to decipher protein targets. (A) Analytical labelling studies using **150-p** and **150-pi** in *M. smegmatis*. (B) Analytical labelling studies using **227-p1** in Mtb H37Ra. (C) Volcano-plot of Mtb cells treated with 12.5 µM **150-p** compared to its UV-control. MmpL3 is also enriched in Mtb. Dotted lines indicate significance cut-off at $p < 0.01$ (n=4) and a Log_2(fold change) > 1. (D) Volcano-plot of Msm cells treated with 12.5 µM **150-p** compared to DMSO. Both MmpL3 and LepB are significantly enriched. Dotted lines indicate significance cut-off at $p < 0.01$ (n=7) and a Log_2(fold change) > 1. (E) Volcano-plot of Mtb cells treated with 5 µM **227-p1** compared to DMSO. Both EphD and EphF are significantly enriched. (F) Profile plot of the mean LFQ-values (n=4) of EphF and EphD across different proteomic samples. **227-p1** significantly enriches EphF and EphD and is strongly out-competed by **227**. **21** and **12** show weaker competition in EphF binding.

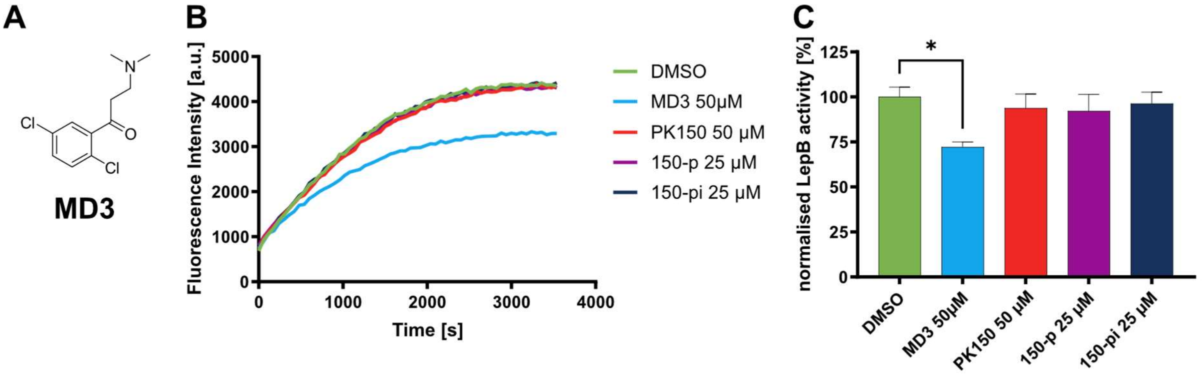

Figure S4. LepB is not a target of PK150 in Mycobacteria. (A) Structure of the known mycobacterial LepB inhibitor **MD3**. MD3 is known to strongly inhibit LepB in Mtb and to weakly inhibit LepB in *M. smegmatis*. (B) Cleavage of a fluorogenic LepB substrate by isolated *M. smegmatis* membranes treated with different compounds. (C) Calculated initial velocities in linear range. Neither **PK150** nor the probes derived from it modulate LepB activity. Statistical significance was determined using ordinary one-way ANOVA (n=3), * = $p < 0.05$.

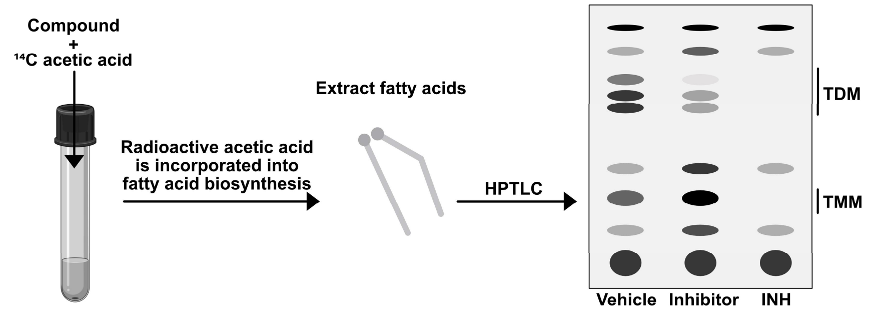

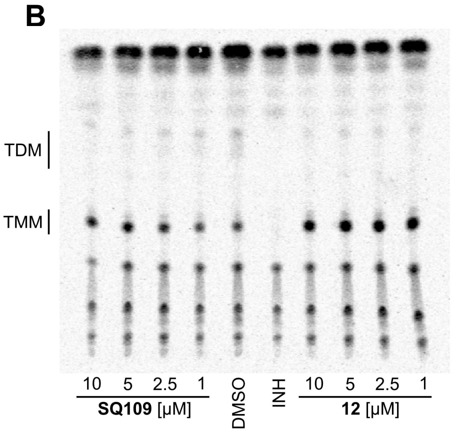

D

Compound	WT H37Rv MIC [µM]	*mmpL3*-TetON +ATc MIC [µM]	*mmpL3*-TetON -ATc MIC [µM]
Ethambutol	4.1<br>5.2	2.3<br>3.4	2.1<br>3.7
Rifampicin	0.05<br>0.07	0.04<br>0.06	0.004<br>0.014
SQ109	0.9<br>1.1	2.4<br>1.8	0.14<br>0.21
227	1.8<br>2.1	1.1<br>1.1	0.36<br>0.42
275	7.8<br>9.8	4.2<br>7.7	4.2<br>9.9
PK150	7.7<br>8.3	4.4<br>5.1	2.1<br>3.7

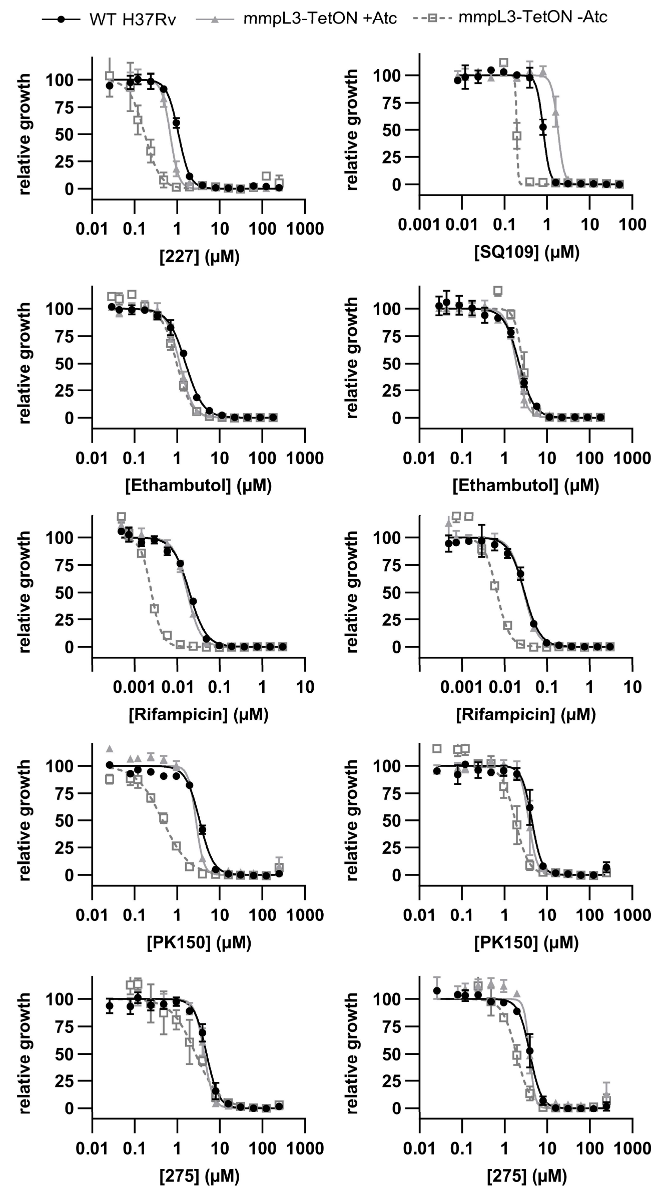

Figure S5. MmpL3 is the main target of the new compounds. (A) MmpL3 activity can be measured in living cells by feeding them ^{14}C acetic acid, which is incorporated into mycolic acids. After the extraction of fatty acids and thin layer chromatography (TLC), their relative abundance can be measured by autoradiography. As TMM is only converted to TDM after export by MmpL3, the ratio of TDM to TMM is an indirect measure of MmpL3 activity. Mycolic acids can be identified by comparison to INH-treated cells, in which mycolic acid biosynthesis is completely inhibited. (B) Example of a TLC plate quantified for the MmpL3 assay. The spots corresponding to TDM and TMM were quantified, the background was subtracted, and the ratio of TDM to TMM was calculated. (C) Dose-response curves of Mtb H37Rv WT,

MmpL3 over- (+ATc), and underexpressing (-ATc) strains dosed with various compounds. Each Compound was performed in three technical triplicates and two biological replicates. Ethambutol activity is known not to be affected by MmpL3 expression levels. Rifampicin activity is known to be affected when MmpL3 is under-expressed. This is not due to direct binding. **SQ109**, a known MmpL3 inhibitor, exhibits distinct shifts in both directions, depending on whether MmpL3 is under- or over-expressed. (D) Table of the MICs from all under- and over-expressing experiments in Figure S5B and Figure 2D. MICs were calculated using the Gompertz model.

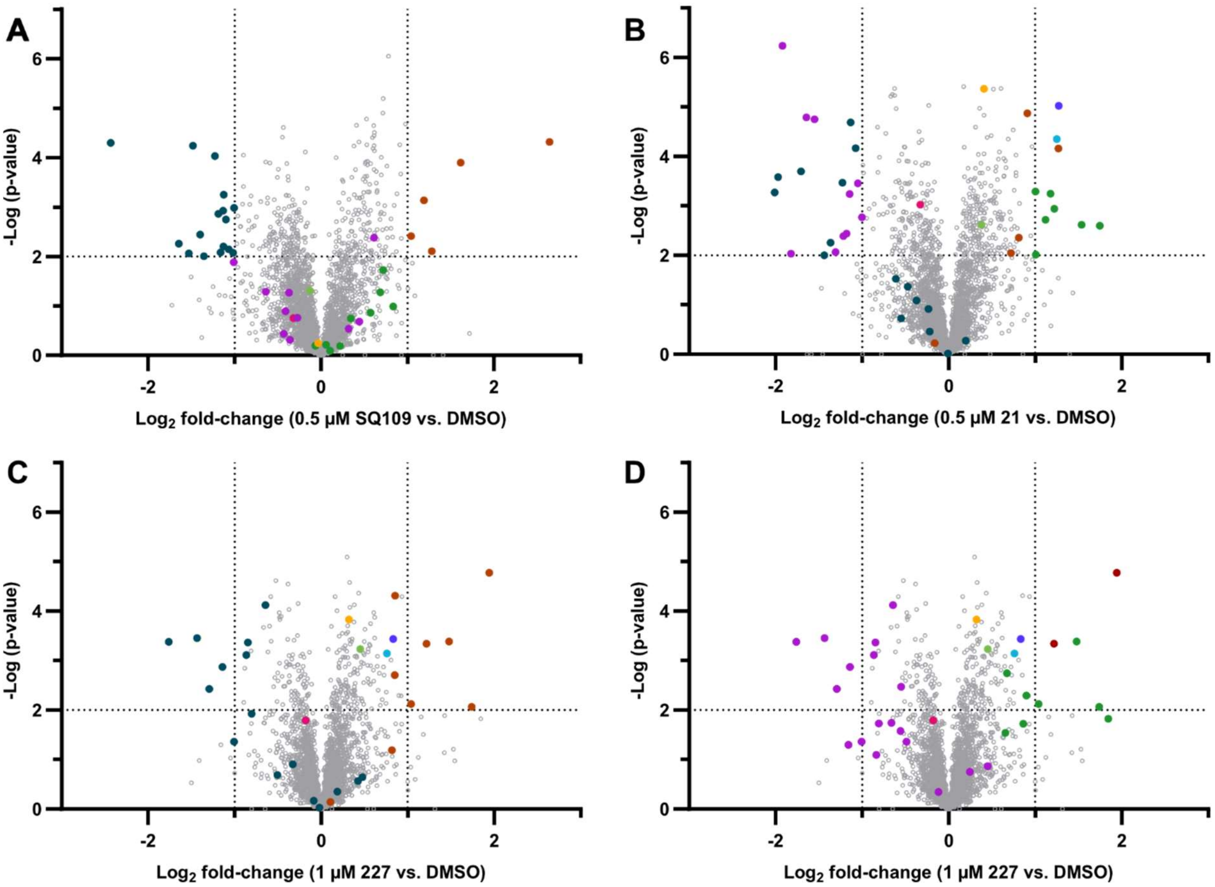

Figure S6. Comparative whole cell analysis. (A - D) Volcano-plot of Mtb cells treated with 0.5 µM **SQ109** (A), 0.5 µM **21** (B) or 1 µM **227** (C, D) compared to DMSO. C and D are the same data, with differently labelled proteins. Coloured Dark blue dots indicate proteins that are significantly down-regulated by a fold-change of more than 2 (Log_2(fold change) > 1) in **SQ109** treated cells. Maroon dots indicate proteins that are significantly up-regulated by a fold-change of more than 2 (Log_2(fold change) > 1) in **SQ109** treated cells. Green dots indicate proteins that are significantly up-regulated by a fold-change of more than 2 (Log_2(fold change) > 1) in **21** treated cells. Violett dots indicate proteins that are significantly down-regulated by a fold-change of more than 2 (Log_2(fold change) > 1) in **21** treated cells. Light blue dot represents IniA, purple dot IniC. Light green dot is the epoxide hydrolase EphF, and orange dot is MmpL3. Pink dot is the transcriptional regulator VirS, responsible for activation of the *mymA* operon. Dotted lines indicate significance cut-off at $p < 0.01$ (n=4) and a Log_2(fold change) > 1.

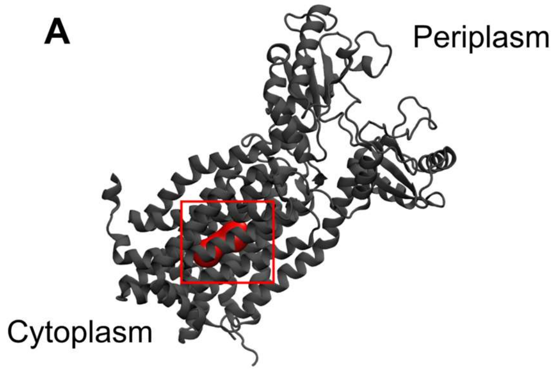

B

Docking scores [kcal / mol]

Compound	Poses 01	02	03
**150-p**	-10.4	-10.3	-10.3
**227**	-10.4	-10.0	-9.9
**21**	-10.9	-10.8	-10.6

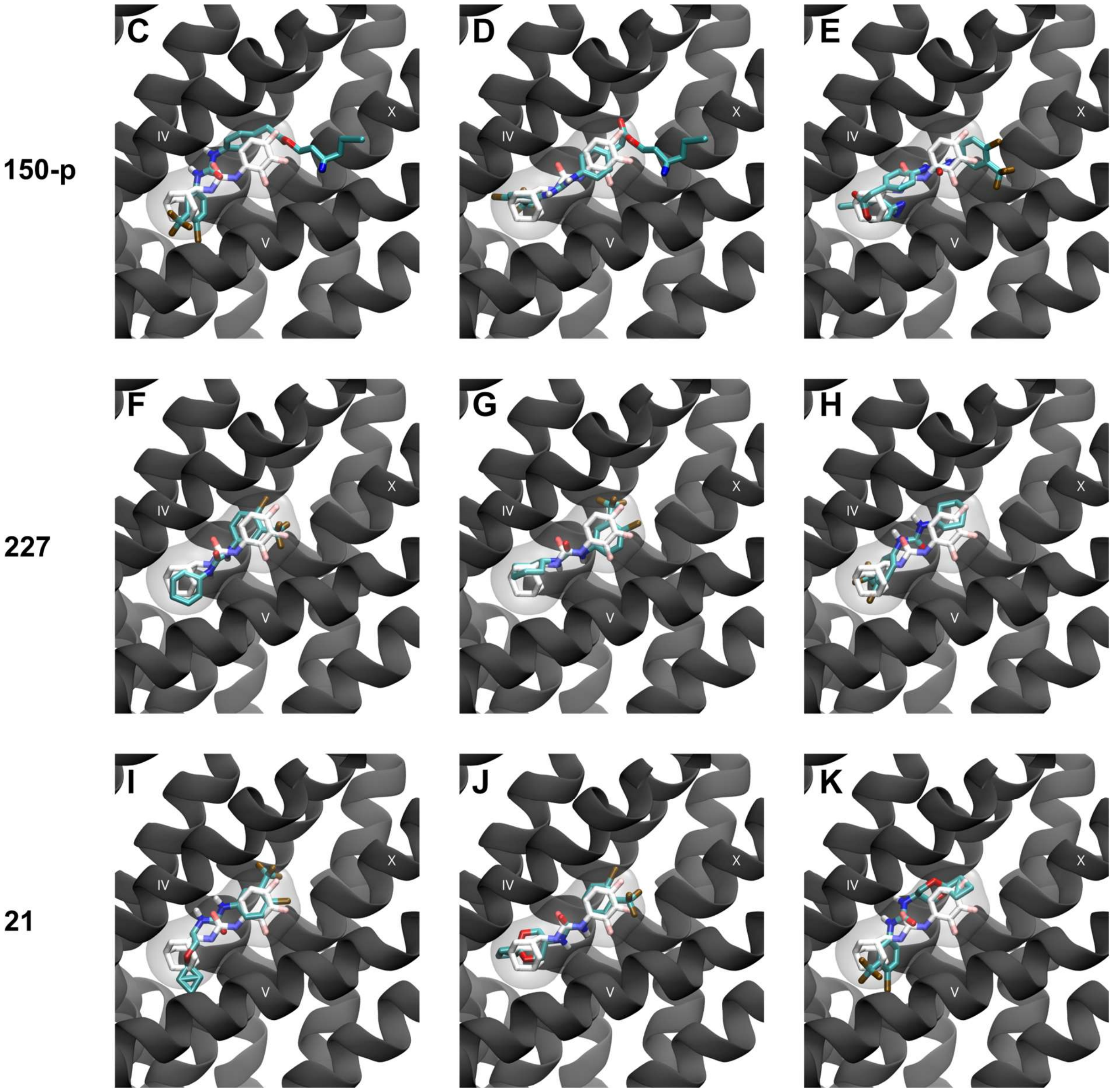

Figure S7. The results of *in silico* docking for compounds of interest with mmpl3. For docking, we used the protein crystal structure of mmpl3 with AU1235 (pdb-id 6ajh), as depicted in (A). The binding pocket within the protein is highlighted as a red volume, and the corresponding protein section, illustrated in the subfigures below, is outlined by a red rectangle. For visualization, we truncated the C-terminal domain of the protein. In (B), docking scores for the top three poses of each compound, obtained from Autodock-Vina, are provided. Subsequent subfigures (C-K) display the top three docking poses of each compound: (C-E) probe **150-p**, (F-H) compound **227**, and (J-K) compound **21**. Throughout,

the protein is represented as a gray cartoon model, with helices IV, V, and X labelled in white. Additionally, the original ligand of the crystal structure (AU1235) is depicted alongside white translucent molecules with a bubble surface. The compounds of interest are shown as coloured liquorice structures, with atoms indicated as follows: O in red, N in blue, F and Cl in gold, and C in green (or white for the original ligand). Generally, we do not show any H atoms

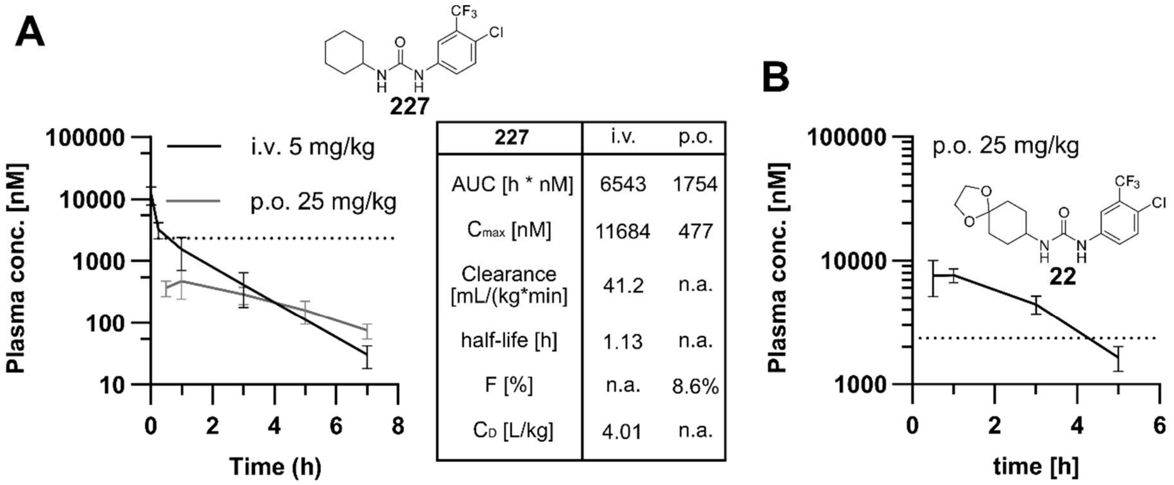

C

Compound	IC_{50} HeLa [µM]	MIC *E. faecalis* KB1 [µM]	MIC *B. animalis* YL2 [µM]	MIC *M. intestinale* YL27 [µM]	MIC Bdq/CFZ resistant *Mtb* [µM]
**227**	24	> 50	> 50	12.5	3.125
**12**	n.d.	> 50	> 50	> 50	0.1
**21**	71	> 50	> 50	> 50	0.78

Figure S8. Initial hit **227** is less bioavailable and more toxic than the novel compound **21** (Figure 4A). (A) Snapshot PK study of compound **227**. The compound was dosed orally at 25 mg/Kg in 95% (20 %) Solutol HS15, 5% DMA, or i.v. at 5mg/kg in 4% Cremophor EL. The plasma concentration was measured over time (p.o. n=2, i.v. n=4). The dotted line indicates the MIC against MTB H37Ra. PK properties are listed in the table on the right of the graph. (B) Snapshot PK study of compound **22**. The compound was dosed orally at 25 mg/Kg in 95% (20 %) Solutol HS15, 5% DMA, and the plasma concentration was measured over time (n=2). The dotted line indicates the MIC against MTB H37Ra. (C) Table with IC_{50} of indicated compounds against HeLa cells in an MTT assay and MICs in three bacterial strains from the mouse microbiota and a Bedaquiline and Clofazimine-resistant clinical isolate of Mtb. Compound **21** displays toxicity at only very high concentrations. The bacteria from the gut microbiota are not affected by **12** and **21** while retaining their activity against a drug-resistant clinical isolate of Mtb. MTT assay: 95% confidence interval (CI) of **21** (52-97 µM) and **227** (15- 37 µM).

Methods

Biochemical Methods

Mycobacterial culture

Unless otherwise stated, mycobacteria were cultured in 7H9+OADC+0.05% Tween-80. OADC (0.5 g/L oleic acid, 50 g/L BSA Frac V, 20 g/L Dextrose, 30 mg/L catalase) was sterile filtered and added 1:10 to autoclaved 7H9 broth (4.7 g/L 7H9 powder). The broth was stored in the fridge for no more than 4 weeks. For each culture, sterile filtered 20% Tween-80 was added freshly to a final concentration of 0.05%. *Mycobacterium tuberculosis* H37Ra was inoculated 1:100 from a glycerol Stock and grown for 10-21 days in T-175 cell culture flasks (37 °C, humidified) prior to any experiment. *M. smegmatis* DSM43756 was inoculated 1:1000 from a glycerol stock and grown for 3 days prior to experiments. *M. tuberculosis* was grown at 37 °C in a humidified plate incubator, while *M. smegmatis* was grown at 37 °C and 200 rpm shaking in Erlenmeyer flasks. All sample preparations for LC-MS based analysis were performed in at least four biological replicates (n=4) from four separately cultured Mycobacterial cultures.

Minimal inhibitory concentration measurements in *M. tuberculosis*

To prepare the inoculum of Mycobacteria, a 10 to 14-day culture of *M. tuberculosis* H37Ra was harvested in a 50 mL falcon tube by centrifugation (7000 xg) and reconstituted in fresh 7H9+OADC+0.05% Tween-80. Five sterile glass beads were added, and the cells were vigorously vortexed to homogenise the culture. The culture was left standing for 10-20 minutes to allow the larger cell clumps to settle at the bottom. The homogenous supernatant was carefully transferred to a new sterile falcon tube, and the optical density (OD_{600}) was measured. The cultures were adjusted to an OD_{600} = 0.0063 (equivalent to 0.05 McFarland) using the respective medium and used as such as an inoculum for MIC measurements. For this, a serial dilution (1:1) was performed in the wells with the respective medium. The compounds were added from a DMSO stock to 100 µL of medium (1:50), and the serial dilution was prepared on the same plate (50 µL + 50 µL). Lastly, 50 µL of the inoculum was added to each well (accept sterile control). Plates with *M. tuberculosis* were sealed with gas-permeable seals, and incubated for 18 days at 37 °C (humidified plate incubator). The absorption at 600 nm was measured in a *TECAN* Infinite M200 Pro microplate reader.

Minimal inhibitory concentration measurements in strains from the Oligo-Mouse-Microbiota

To prepare the inoculum of the microbiome strains, strains were inoculated from glycerol stocks into AAM broth (see table below) under anaerobic conditions (Whitley DG250 Anaerobic Workstation) and were grown overnight at 37 °C. The following day, the optical density (OD_{600}) was measured, and the cultures were adjusted to an OD_{600} = 0.0063 (equivalent to 0.05 McFarland) using the respective medium and used as such as an inoculum for MIC measurements. For this, a serial dilution (1:1) was performed in the wells with the respective medium. The compounds were added from a DMSO stock to 100 µL of medium (1:50), and the serial dilution was prepared on the same plate (50 µL + 50 µL).

Lastly, 50 µL of the inoculum was added to each well (accept sterile control). Plates were incubated for 24 h at 37 °C in the anaerobic chamber. The absorption at 600 nm was measured in a *TECAN* Infinite M200 Pro microplate reader.

**per litre AAM broth**	**components**
18.5 g	Brain Heart Infusion (BHI)
5 g	Yeast Extract
15 g	Trypticase Soy Broth
2.5 g	K_2HPO_4
0.5 g	(D-)Glucose
pH was adjusted to 7 before autoclaving using 1 M NaOH	
**Supplements**	
0.4 g	Na_2CO_3 (sodium carbonate)
0.5 g	Cysteine Hydrochloride
5 mg	Menadione (in 96 % Ethanol)
1 mg	Haemine
3% (v/v)	Complement-inactivated Fetal Calf Serum (FCS)
For the preparation of the supplement solutions, supplements were dissolved in ddH_2O and sterile filtered (0.2 micron), except Menadione, which was dissolved in Ethanol (96 %). *Haemine: dissolve 10 mM Haemine in 50 mM NaOH sterile filter (0.2 micron)	

Kinetic Turbidimetric Solubility

The desired compounds were sequentially diluted in DMSO in a 96-well plate. 1.5 µL of each well were transferred into another 96-well plate and mixed with 148.5 µL of PBS. Plates were shaken for 5 min at 600 rpm at room temperature (r.t.), and the absorbance at 620 nm was measured. Absorbance values were normalized by blank subtraction and plotted using GraphPad Prism 10 (GraphPad Software, San Diego, CA, USA). Solubility (S) was determined based on the First X value of AUC function using a threshold of 0.005.

Gel-based fluorescent labelling in Mycobacteria

The optical density of a late exponential to early stationary Mycobacterial culture (*M. smegmatis*: 3 days, *M. tuberculosis*: 21 days) was measured, and the cells were harvested in a 50 mL falcon tube by centrifugation (7000 xg, 10 min, 4 °C). The cell pellet was washed with 20 mL cold PBS (7000 xg, 10 min, 4 °C) and reconstituted in PBS to a theoretical OD_{600}- value of 10. For each labelling condition, 220 µL of the cell suspension was transferred into an Eppendorf tube. 2.2 µL of the respective photo-probe was added (1 % final DMSO concentration) to the cell suspension. The cells were incubated with the photo-probes for 1 hour (37 °C, 200 rpm). After incubation, the cells were transferred to a transparent 48-well plate. While cooling, the cells were irradiated with UV light using an 18 W *Philips* TL-D BLB UV lamp (λ= 310- 400 nm) to allow photo-crosslinking of the photo-reactive group. After photo-crosslinking, the cells were transferred into an Eppendorf tube and washed with 1 mL cold PBS (7000 xg, 4°C, 5 min). To lyse the cells, the pellets were reconstituted in 150 µL PBS supplemented with 0.5 % SDS and 1 % Triton X-100. The cells were sonicated (10 s, 30 % intensity, Sonopuls HD 2070 ultrasonic rod, *Bandelin electronic GmbH*) and transferred into bead-mill lysis tubes filled with 0.1 mm zirconium

beads. The cells were lysed at 6500 rpm for 3x 30 s using a precellys 24 bead beater (*peqlab*). The Lysate was centrifuged at 10.000 xg for 10 minutes, and the supernatant (120 µL) was transferred into a microcentrifuge tube and centrifuged for 10 minutes at 21.000 xg. The supernatant (100 µL) was transferred into a new centrifuge tube. The lysate was clicked to rhodamine azide by the addition of 7 µL freshly prepared click-mix (1 µL 10 mM Rhodamine-azide in DMSO, 3 µL 1.67 mM tris(benzyltriazoylmethyl)amine (TBTA) in 80% *t*BuOH and 20% DMSO, 1 µL 50 mM $CuSO_4$ in water, and 2 µL 52 mM TCEP in water) and incubation for 1 h at RT. The reaction was quenched by adding 500 µL ice-cold acetone and incubating for 1 h at -20°C to precipitate all proteins. The proteins were harvested by centrifugation (20 min, 21.000 xg, 4 °C) and reconstituted in Laemmli buffer by sonication (10 s, 10% intensity). The samples were then analysed by SDS-PAGE and the fluorescence was recorded in a Fujifilm Las-4000 Luminescent Image Analyser with a Fujinon VRF43LMD3 and a 575DF20 filter.

Preparative labelling in *M. smegmatis* using probe 150-p for Mass spectrometry

Labelling, Lysis and Click. The *M. smegmatis* DSM43756 cells were inoculated into 5 mL 7H9 + ADC broth and incubated for 40 hours (37 °C, 200 rpm). 4 mL of the culture was then used to inoculate 60 mL 7H9 + ADC broth in 250 mL Erlenmeyer flasks with deep chicanes. The cells were grown (37 °C, 200 rpm) until they reached the stationary phase (20 – 23 hours). The OD_{600}- value was measured, and the cells were harvested in 50 mL falcon tubes by centrifugation (6000 xg, 5 min, 4 °C). The cell pellet was washed in 20 mL cold PBS (7000 xg, 5 min, 4 °C) and resuspended in PBS to a theoretical OD_{600}- value of 40. For each labelling condition, 1 mL of the cell suspension was transferred into an Eppendorf tube. 10 µL of the respective photo-probe was added (1 % final DMSO concentration) to the cell suspension. The cells were incubated with the photo-probes for 1 hour (37 °C, 200 rpm). After incubation, the cells were transferred to transparent 6-well plates. While cooling, the cells were irradiated with UV-light using a *Hitachi* FL8BL-B UV-lamp (λ= 310- 400 nm) to allow photo-crosslinking of the diazirine group. After photo-crosslinking the cells were transferred into an Eppendorf tube and washed with 1 mL cold PBS (7000 xg, 4°C, 5 min). The cell pellet was frozen and stored at -80 °C until cell lysis. To lyse the cells, the pellets were resuspended in 1 mL PBS supplemented with 0.5 % SDS and 1 % Triton X-100. The resuspended cells were lysed by sonication (3x 15 s, 80 % intensity), cooling the samples on ice in between each sonication step. The cell debris was now removed by centrifugation (6000 xg, 5 min, 4 °C) and the supernatant was transferred into a new Eppendorf tube. The protein concentrations of the samples were determined by BCA assay (Roti Quant, Roth), and all samples were adjusted to the same protein concentration using the lysis buffer. 1 mL of each sample was transferred into a 15 mL falcon tube and clicked to biotin azide by the addition of 80 µL freshly prepared click-mix (20 µL 10 mM biotin-azide in DMSO, 30 µL 1.67 mM tris(benzyltriazoylmethyl)amine (TBTA) in 80% *t*BuOH and 20% DMSO, 10 µL 50 mM $CuSO_4$ in water, and 20 µL 52 mM TCEP in water) and incubation for 1 h at RT. The click-reaction was quenched, and the proteins precipitated by the addition of 5 mL cold (-80 °C) Acetone (5x excess volume). The samples were incubated overnight at -20 °C.

Enrichment and Digestion. The proteins were harvested (21,000 xg, 4°C, 20 min) and washed twice with methanol. Therefore, the pellet was reconstituted in 500 µL methanol, sonicated (10 % intensity, 10 s, Sonopuls HD 2070 ultrasonic rod, *Bandelin electronic GmbH*) and harvested again via centrifugation as before. Next, the proteins were reconstituted in 500 µL 0.2% SDS in PBS by sonication (10 % intensity, 10 s) and the insoluble part was removed by centrifugation. The soluble fraction was added to 50 µL of washed (3x in 0.2% SDS in PBS) avidin-agarose beads and incubated for 1 h with continuous mixing. Afterwards, the samples were washed three times with 0.2% SDS in PBS, two times

with 6 M urea and 3 times with PBS. For this the samples were centrifuged for 3 minutes at 400 xg and the supernatant war discarded each time. The beads were now resuspended in 200 µL digestion buffer 1 (3.6 M urea, 1.1 M thiourea, 5 mM TCEP in 20 mM Hepes, pH 7.5) and incubated for 30 minutes at 25 °C, 1000 rpm. The reduced bead-bound proteins were now alkylated with with 5.5 mM iodoacetamide (30 min, 1000 rpm, 25 °C) and then the reaction was quenched with 10 mM DTT (30 min, 1000 rpm, 25 °C). Samples were first digested with 0.5 µg LysC (Wako) for 2 h at 25 °C before adding 600 µL 50 mM TEAB with 1.5 µg Trypsin (Promega) and a further incubation of 16 hours at 37 °C, 1000 rpm. The digest was stopped by adding 1 % FA and the peptides were desalted using 50 mg Sep-Pak C18 cartridges (Waters Corp.). Therefore, the cartridges were equilibrated with 1 mL acetonitrile, 1 mL (elution biffer (80% acetonitrile, 0.5 % FA in H_2O) and 3 ml of wash buffer 1 (0.1 % TFA in H_2O). The samples were loaded, washed with 3 ml wash buffer 1 and 0.5 mL of 0.5% FA in H_2O. The peptides were eluted with 2x 250 µL elution buffer and dried in a centrifugal evaporator. The peptides were reconstituted in 30 µL 1% FA and measured on an Q Exactive Plus instrument (*Thermo Fischer*).

LC-MS measurements on QExacative Plus. Peptide Samples were analyzed on an UltiMate 3000 nano HPLC system (Dionex) equipped d with an Acclaim C18 PepMap100 (75 µm ID × 2 cm) trap column and a 25 cm Aurora Series emitter column (25 cm × 75 µm ID, 1.6 µm FSC C18) (Ionoptics) separation column (column oven heated to 40 °C) coupled to an Q Exactive Plus Instrument (Thermo Fisher). For peptide separation, samples were loaded on the trap column and washed for 10 min with 0.1% TFA in ddH2O at a flow rate of 5 µL/min. Subsequently, peptides were transferred to the analytical column for peptide separation and separated using the following 132 min gradient (Buffer A: H2O + 0.1% FA; B: MeCN + 0.1% FA) with a flow rate of 300 nL/min.: in 7 min to 5% B, in 105 min from 5% to 22%, in 10 min from 22 to 35% and in another 10 min to 90% B. Separation gradient was followed by a column washing step using 90% B for 10 min and subsequent column re-equilibration with 5% B for 5 min. Peptides were ionized at a capillary temperature of 275 °C and the instrument was operated in a Top12 data dependent mode. For full scan acquisition, the orbitrap mass analyzer was set to a resolution of R = 140000, an automatic gain control (AGC) target of 3e6, and a maximal injection time of 80 ms in a scan range of 300-1500 m/z. Precursors having a charge state of >1, a minimum AGC target of 1e3 and intensities higher than 1e4 were selected for fragmentation. Peptide fragments were generated by HCD (higher-energy collisional dissociation) with a normalized collision energy of 27 % and recorded in the orbitrap at a resolution of R = 17500. Moreover, the AGC target was set to 1e5 with a maximum injection time of 100 ms scan range. Dynamic exclusion duration was set to 60 s and isolation was performed in the quadrupole using a window of 1.6 m/z.

Data analysis of QExactive Plus samples. MS raw data was analysed using MaxQuant[6] software (version 2.0.3.0) and peptides were searched against the UniProt reference proteome for *M. smegmatis* mc^{2}155 (taxon identifier: 246196, downloaded on 02.05.2022). Carbamidomethylation of cysteines was set as fixed modification, and oxidation of methionines and acetylation of N-termini were set as variable modifications. Trypsin was set as the proteolytic enzyme with a maximum of 2 missed cleavages. For the main search, precursor mass tolerance was set to 4.5 ppm and fragment mass tolerance to 0.5 Da. Label-free quantification (LFQ) mode was activated with a LFQ minimum ratio count of 2. Second peptide identification was enabled, and false discovery rate (FDR) determination carried out by applying a decoy database and thresholds were set to 1% FDR at peptide-spectrum match and at protein levels and “match between runs” (0.7 min match and 20 min alignment time windows) option was enabled. Normalized LFQ intensities extracted from the MaxQuant result

table proteinGroups.txt were further analysed with Perseus software[7] (version 2.03.1). Prior to analysis, putative contaminants, reverse hits and only identified by site hits were removed. Normalized LFQ intensities were log2 transformed and proteins with at least four valid values in at least one group were used for missing value imputation from normal distribution (width 0.3, downshift 1.8, total matrix). Two-sample Students' t-test including permutation-based multiple testing correction (FDR = 0.05) was performed.

Preparative labelling in *M. tuberculosis* for Mass spectrometry

Labelling, Lysis and Click. The optical density of each biological replicate culture of the Mycobacterial culture of *M. tuberculosis* H37Ra was measured and the cells were harvested in a 50 mL falcon tube by centrifugation (7000 xg, 10 min, 4 °C). The cell pellet was washed with 20 mL cold PBS (7000 xg, 10 min, 4 °C) and reconstituted in PBS to a theoretical OD_{600}- value of 15. For each labelling condition, 500 µL of the cell suspension was transferred into an Eppendorf tube. 5 µL of the respective photo-probe was added (1 % final DMSO concentration) to the cell suspension. The cells were incubated with the photo-probes for 1 hour (37 °C, 200 rpm). After incubation, the cells were transferred to a transparent 48-well plate. While cooling, the cells were irradiated with UV light using an 18 W *Philips* TL-D BLB UV lamp (λ= 310- 400 nm) to allow photo-crosslinking of the photo-reactive group. After photo-crosslinking, the cells were transferred into an Eppendorf tube and washed with 1 mL cold PBS (7000 xg, 4°C, 5 min). To lyse the cells, the pellets were reconstituted in 150 µL PBS supplemented with 0.5 % SDS and 1 % Triton X-100. The cells were sonicated (10 s, 30 % intensity, Sonopuls HD 2070 ultrasonic rod, *Bandelin electronic GmbH*) and transferred into bead-mill lysis tubes filled with 0.1 mm zirconium beads. The cells were lysed at 6500 rpm for 3x 30 s using a precellys 24 bead beater (*peqlab*). The Lysate was centrifuged at 10.000 xg for 10 minutes, and the supernatant (120 µL) was transferred into a micro-centrifuge tube and centrifuged for 10 minutes at 21.000 xg. The supernatant (100 µL) was transferred into a new centrifuge tube. The protein concentrations of the samples were determined by BCA assay (Roti Quant, Roth), and all samples were adjusted to a protein concentration of 2.23 mg/mL using the lysis buffer. 45 µL of Lysate from each sample was transferred to a 96-well plate (Polypropylene, V-bottom, *Greiner* cat. 651201), and the samples were clicked to biotin-azide. For this, 4.9 µL of click-mix (0.6 µL 20 mM biotin-azide in DMSO, 2.5 µL 1.67 mM tris(benzyltriazoylmethyl)amine (TBTA) in 80% *t*BuOH and 20% DMSO, 1.2 µL 50 mM $CuSO_4$ in water, and 0.6 µL 100 mM TCEP in water) were added to each well, the plate was sealed and incubated for 90 minutes at 25 °C, 950 rpm. The reaction was quenched by the addition of 65 µL 8 M urea with 10 mM TCEP and 20 mM iodoacetamide (IAA). After 15 minutes at 25 °C, 950 rpm to allow alkylation of cysteines, the reaction was quenched by adding 2 µL 500 mM DTT per sample.

Enrichment and Digestion. All reagents used were LC-MS grade. The sample processing is based on the publication from Becker et al.[8] with some alterations. 10 µL of a 2-fold concentrated 1:1 mix of washed (3x H_2O) hydrophobic and hydrophilic carboxylate-coated magnetic beads (*Cytiva*) was added to each sample. To precipitate the proteins onto the beads, 175 µL ethanol were added to each sample. The plate was now placed into an automated liquid handling system (*Hamilton* Microlab Prep) for further processing. The plate was incubated for 5 minutes, 500 rpm. For each washing step, the plate was placed onto a 96-well ring magnet (*Alpaqua*, Magnum FLX) for 90 s and the supernatant was removed with a low draw speed (20 µL/s) in order to not remove any beads. The plate was then

removed from the magnet and the next washing solution was added, followed by 1 minutes shaking at 800 rpm. In this way, the samples were washed 3x with 180 µL 80% ethanol and once with 180 µL acetonitrile. To elute the proteins from the carboxyl-coated beads, 75 µL 0.2% SDS in PBS was added to the samples and the plate was incubated for 5 minutes at 40°C, while shaking at 800 rpm. The plate was then placed on the magnet and the supernatant was transferred into new wells. This step was repeated and the additional 75 µL of eluted proteins was added to yield 150 µL of eluted proteins in new wells. Meanwhile, streptavidin magnet beads (*New England Biolabs*, cat# S1420S) were washed 3x with 0.2 % SDS in PBS. 50 µL of the washed streptavidin beads were added to each well containing eluted protein sample. The plate was taken out of the liquid handling system, sealed, and incubated for 1 h at 25 °C, 800 rpm in a plate shaker, with a heated lid to prevent condensation. This allowed the binding of labelled proteins to the streptavidin beads. Afterwards, the plate-seal was removed and the plate was placed back into the liquid handling system. The beads were washed 3x with 180 µL 0.1% NP-40 in PBS, 2x with 180 µL 6 M urea and 3x with 200 µL H_2O. The bead-bound proteins were digested in 100 µL 50 mM TEAB with 0.5 µg Trypsin (sequencing grade, *Promega*) overnight at 37 °C in a tightly sealed plate and with a heated lid at 800 rpm. After the tryptic digest, the peptides were eluted from the beads in the liquid handling system and subsequently desalted. For this, the samples were loaded on pre-equilibrated stage tips with two layers of SDP-RPS (Empore, *3M*). The desalting was performed as previously described Coscia et al.[9] with some minor alterations. In short, the stage-tips were equilibrated with 150 µL wash buffer 1 (1% (vol/vol) TFA in isopropanol) before loading the samples. The samples were loaded at 500 xg for 10 minutes, followed by a wash step with 170 µL wash buffer 1 (800 xg, 10 minutes) and another washing step with 170 µL wash buffer 2 (0.2% (vol/vol) TFA in H_2O). The peptides were eluted with 50 µL elution buffer (1% ammonia, 80% acetonitrile) by centrifugation at 300 xg for 5 minutes, followed by 800 xg for 5 minutes. Samples were dried in a centrifugal evaporator and reconstituted in 35 µL 1% FA for LC-MS measurements on a timsTOF Pro instrument in data-independent acquisition.

Full Proteome analysis in M*tb* H37Ra

To prepare the inoculum, four 14-day cultures of *M. tuberculosis* H37Ra were harvested in 50 mL falcon tubes by centrifugation (7000 xg) and reconstituted in fresh 7H9+OADC+0.05% Tween-80. Five sterile glass beads were added, and the cells were vigorously vortexed to homogenise the culture. The culture was left standing for 10-20 minutes to allow the larger cell clumps to settle at the bottom. The homogenous supernatants were carefully transferred to a new sterile falcon tube, and the optical densities (OD_{600}) were measured. The cultures were adjusted to the lowest OD_{600} value and used to inoculate 20 mL fresh medium (1:20) in T-25 cell culture flasks with the respective compound already added (1:1000, 0.1% DMSO). After 10 days at 37 °C (humidified), 10 mL of each culture was harvested by centrifugation (7000 xg, 4 °C), washed with cold PBS and transferred into a micro centrifuge tube. The cell pellets were reconstituted in 150 µL PBS supplemented with 0.5 % SDS and 1 % Triton X-100. The cells were sonicated (10 s, 30 % intensity, Sonopuls HD 2070 ultrasonic rod, *Bandelin electronic GmbH*) and transferred into bead-mill lysis tubes filled with 0.1 mm zirconium beads. The cells were lysed at 6500 rpm for 3x 30 s using a precellys 24 bead beater (*peqlab*). The Lysate was centrifuged at 10.000 xg for 10 minutes, and the supernatant (120 µL) was transferred into a micro-centrifuge tube and centrifuged for 10 minutes at 21.000 xg. The supernatant (100 µL) was transferred into a new centrifuge tube. The protein concentrations of the samples were determined by BCA assay (Roti Quant, Roth), and all samples were adjusted to a protein concentration of 0.6 mg/mL using the lysis buffer. 25

µL of each sample was transferred into a 96-well plate and the samples were alkylated with 10 mM TCEP and 20 mM iodoacetamide (IAA). 10 µL of a 1:1 mix of washed (3x H_2O) hydrophobic and hydrophilic carboxylate-coated magnetic beads (*Cytiva*) was added to each sample. To precipitate the proteins onto the beads, 175 µL ethanol were added to each sample. The plate was now placed into an automated liquid handling system (*Hamilton* Microlab Prep) for further processing. The plate was incubated for 5 minutes, 500 rpm. For each washing step, the plate was placed onto a 96-well ring magnet (*Alpaqua*, Magnum FLX) for 90 s and the supernatant was removed with a low draw speed (20 µL/s) in order to not remove any beads. The plate was then removed from the magnet and the next washing solution was added, followed by 1 minutes shaking at 800 rpm. In this way, the samples were washed 3x with 180 µL 80% ethanol and once with 180 µL acetonitrile. The bead-bound proteins were digested in 100 µL 50 mM TEAB with 0.5 µg Trypsin (sequencing grade, *Promega*) overnight at 37 °C in a tightly sealed plate and with a heated lid at 800 rpm. After the tryptic digest, the peptides were eluted from the beads in the liquid handling system, the beads were washed with 40 µL of 3.5% formic acid and the resulting 140 µL were directly transferred into MS-vials without any desalting and 2.5 µL of each sample were measured on a timsTOF Pro in data-independent acquisition mode.

LC-MS measurements and data analysis timsTOF Pro samples

LC-MS measurements on timsTOF Pro. Peptides were measured and online-separated using an UltiMate 3000 nano HPLC system (*Dionex*) coupled to a *Bruker* timsTOF Pro mass spectrometer via a CaptiveSpray nano-electrospray ion source and *Sonation* column oven. Peptides were first loaded on the trap column (Acclaim PepMap 100 C18, 75 µm ID x 2 cm, 3 µm particle size, *Thermo Scientific*), washed with 0.1% formic acid in water for 7 min at 5 µL/min and subsequently transferred to the separation column (*IonOpticks* Aurora C18 column, 25 cm × 75 µm, 1.7 µm) and separated over a 36 min gradient from 5% to 17% B, then to 25 % B over 18 min, then to 37% B over 6 min, followed by 10 min at 95% before re-equilibration and at a flow rate of 400 nL/min. The mobile phases A and B were 0.1 % (v/v) formic acid in water and 0.1% (v/v) formic acid in acetonitrile, respectively. The timsTOF Pro was operated in data-independent dia-PASEF mode with the dual TIMS analyser operating at equal accumulation and ramp times of 100 ms each with a set $1/K_0$ ion mobility range from 0.60 to 1.60 V × s/cm^2 for MS1 scans. The dia-PASEF settings for fragmentation were set to a mass range of 400 to 1201 m/z and an ion mobility range of 0.60 to 1.43 V × s × cm^{-2}. Two ion mobility isolation windows were performed per dia-PASEF scan with 26 m/z window widths. A total of 32 isolation windows with 1 m/z overlaps to cover the mass range were used resulting in 16 dia-PASEF scans per MS1 scan and an estimated total cycle time of 1.80 s (see Table). The collision energy was ramped linearly as a function of the mobility from 59 eV at $1/K_0$ = 1.3 V × s × cm^{-2} to 20 eV at $1/K_0$ = 0.85 V × s × cm^{-2}. TIMS elution voltages were calibrated linearly to obtain the reduced ion mobility coefficients ($1/K_0$) using three Agilent ESI-L Tuning Mix ions (*m/z* 622, 922 and 1,222) spiked on the CaptiveSpray Source inlet filter. The timsTOF Pro was operated in data-independent dia-PASEF mode with the dual TIMS analyser operating at equal accumulation and ramp times of 100 ms each with a set $1/K_0$ ion mobility range

from 0.60 to 1.60 V × s/cm^2 for MS1 scans. The dia-PASEF settings for fragmentation were set to a mass range of 400 to 1201 m/z and an ion mobility range of 0.60 to 1.43 V × s × cm^{-2}. Two ion mobility isolation windows were performed per dia-PASEF scan with 26 m/z window widths. A total of 32 isolation windows with 1 m/z overlaps to cover the mass range were used resulting in 16 dia-PASEF scans per MS1 scan and an estimated total cycle time of 1.80 s (see Table below). The collision energy was ramped linearly as a function of the mobility from 59 eV at $1/K_0$ = 1.3 V × s × cm^{-2} to 20 eV at $1/K_0$ = 0.85 V × s × cm^{-2}. TIMS elution voltages were calibrated linearly to obtain the reduced ion mobility coefficients ($1/K_0$) using three Agilent ESI-L Tuning Mix ions (*m/z* 622, 922 and 1,222) spiked on the CaptiveSpray Source inlet filter.

MS Type	Scan	Start IM [1/K0]	End IM [1/K0]	Start Mass [m/z]	End Mass [m/z]
MS1	0	0.6	1.6	100	1700
dia-PASEF	1	0.9	1.2	800	826
dia-PASEF	1	0.6	0.9	400	426
dia-PASEF	2	0.92	1.22	825	851
dia-PASEF	2	0.62	0.92	425	451
dia-PASEF	3	0.93	1.23	850	876
dia-PASEF	3	0.63	0.93	450	476
dia-PASEF	4	0.95	1.25	875	901
dia-PASEF	4	0.65	0.95	475	501
dia-PASEF	5	0.96	1.26	900	926
dia-PASEF	5	0.66	0.96	500	526
dia-PASEF	6	0.98	1.28	925	951
dia-PASEF	6	0.68	0.98	525	551
dia-PASEF	7	0.99	1.29	950	976
dia-PASEF	7	0.69	0.99	550	576
dia-PASEF	8	1.01	1.31	975	1001
dia-PASEF	8	0.71	1.01	575	601
dia-PASEF	9	1.02	1.32	1000	1026
dia-PASEF	9	0.72	1.02	600	626
dia-PASEF	10	1.04	1.34	1025	1051
dia-PASEF	10	0.74	1.04	625	651
dia-PASEF	11	1.06	1.36	1050	1076
dia-PASEF	11	0.76	1.06	650	676

dia-PASEF	12	1.07	1.37	1075	1101
dia-PASEF	12	0.77	1.07	675	701
dia-PASEF	13	1.09	1.39	1100	1126
dia-PASEF	13	0.79	1.09	700	726
dia-PASEF	14	1.1	1.4	1125	1151
dia-PASEF	14	0.8	1.1	725	751
dia-PASEF	15	1.12	1.42	1150	1176
dia-PASEF	15	0.82	1.12	750	776
dia-PASEF	16	1.13	1.43	1175	1201
dia-PASEF	16	0.83	1.13	775	801

Data analysis of timsTOF Pro measurements. MS-data were analysed using DIA-NN[10] (version 1.8.1) using the library-free mode. The library was generated using the UniProt reference proteome of *M. tuberculsosis* H37Rv (taxon identifier: 83332, downloaded on 09.12.2022). For the precursor ion generation, library generation and Deep-learning based spectra, RTs and IMs prediction were enabled. Trypsin/P with maximum 2 missed cleavages; protein N-terminal M excision on; Carbamidomethyl on C as fixed modification; no variable modification; peptide length from 7 to 30; precursor charge 2–4; precursor m/z from 300 to 1800; fragment m/z from 200 to 1800 for TIMS data. Precursor FDR was set to 0.01; Mass accuracy, MS1 accuracy and Scan window were all set to 0; isotopologues, MBR and Remove likely interferences were on; Neural network classifier in single-pass mode; protein inference at gene level; heuristic protein inference was enabled (--relaxed-prot-inf); quantification strategy was set to Robust LC (high precision); Cross-run normalisation was RT-dependent; Library generation smart profiling; Speed and Ram usage was set to optimal results. LFQ quantities were extracted from the protein groups (pg) results file and were further analysed with Perseus software[7] (version 2.03.1). LFQ intensities were log2 transformed and protein groups with less than four valid values in at least one group were filtered out. Two-sample Students' t-test including permutation-based multiple testing correction (FDR = 0.05) was performed for all relevant comparisons to calculate the fold-change and statistical relevance. Results table were exported and graphs prepared using *Graphpad* Prims 10.01. The mass spectrometry proteomics data have been deposited to the ProteomeXchange Consortium via the PRIDE[11] partner repository.

Membrane integrity assay in *M. tuberculosis* H37Ra

To asses cell permeability upon treatment with compounds, cells were grown until they reached an OD_{600} value of 0.3 - 0.7. The cells were transferred to a 50 mL falcon tube, five sterile glass beads were added, and the cells were vigorously vortexed to homogenise the culture. The culture was left standing

for 10-20 minutes to allow the larger cell clumps to settle at the bottom. The homogenous supernatants were carefully transferred to a new sterile falcon tube, and the optical densities (OD_{600}) were measured. Cells were harvested and washed with 5 mM HEPES-NaOH buffer (pH 7.2 supplemented with 5 mM glucose) and then resuspended in the same buffer to an OD_{600} of 0.4. The assay was conducted in 96-well plates (Nunc flat-bottom transparent 96-well plates: *Thermo Fischer Scientific*) with 100 µL cell-suspension per well. As a negative control the assay was conducted in assay buffer without any cells. The assay was started by adding 1 µL of 1 mM propidium iodide in DMF to each well (10 µM final concentration) and incubating at 37 °C in the *TECAN* Infinite M200 Pro microplate reader to allow the propidium iodide to be integrated into the membranes. During incubation, fluorescence was measured (535 nm excitation and 617 nm emission). After 15 minutes, 1 µL of compounds were added from DMSO-stocks with the appropriate concentrations. The fluorescence was measured for 45 minutes at 37 °C. As a positive control, 16 µg/mL of the detergent benzalkonium chloride (BAC) were added to the cell suspension.

Measuring membrane bound LepB activity

Membrane preparation. A 2-day culture of *M. smegmatis* DSM43756 was inoculated (1:100) into 500 ml 7H9 + ADC broth and incubated for 40 hours (37 °C, 200 rpm). After incubation, the cells were harvested (7000 xg, 10 min, 4 ˚C) and washed with cold PBS (7000 xg, 10 min, 4 ˚C). The cells were reconstituted in 20 mL lysis buffer (50 mM TRIS/HCL pH 7.5) and lysed using a bead beater homogenizer (6 x 5500 rpm, 15 s, 2 min cooling breaks on ice after each run; Precellys Ceramic Kit CK01L, 7.0 mL tubes; Precellys 24 Homogenizer, *Bertin Technologies*). The lysate was centrifuged (12,000 x g, 4°C, 10 min) to remove intact cells and debris. Membranes were collected from the supernatant after centrifugation (39,000 x g, 4°C, 75 min) and reconstituted in cold sodium phosphate buffer (50 mM, pH 7.5). Protein concentrations were determined using Pierce BCA Protein assay kit (*Thermo Fisher Scientific*, *Pierce Biotechnology*).

FRET assay with membrane-bound LepB. LepB activities were measured using a Förster Resonance Energy Transfer (FRET) assay, as described by Rao et al.[12] with some alterations. A synthetic peptide based on the known LepB from *E. coli* based on the sequence of maltose binding protein, modified by 2-aminobenzoic acid (abz) and 3-nitrotyrosine ($Y(NO_2)$ (Y(NO2)-FSASALAKIK(Abz), *Eurogentec*) was used as the substrate. Assays were performed with membrane from *M. smegmatis DMS43756* (12.5 µg/mL total membrane protein concentration) in sodium phosphate buffer pH 7.5. Membranes containing endogenous LepB (100 µL/well final volume, sodium phosphate buffer, 50 mM, pH 7.5) were treated with the respective compound or DMSO (1:100, final assay concentration of DMSO from compound stocks 1%) at 37°C for 5 min. After addition of substrate (10 µM final substrate concentration, final dimethylformamide (DMF) concentration from substrate stock 1%) turnover was monitored by measuring fluorescence in a *Tecan* infinite 200Pro plate reader (340 nm excitation and 510 nm emission wavelengths, fluorescence top reading mode) at 37°C. Substrate turnover was determined and normalized to DMSO-treated samples. The Graphs were plotted using Graphpad Prism and the initial velocities were fitted in the linear range. Statistical significance was determined using ordinary one-way ANOVA (n=3).

TDM/TMM autoradiography assay (MmpL3)

A stationary culture of *M. tuberculosis* H37Ra was inoculated into fresh medium (1:10) and grown to an OD_{600} of 0.4. The culture was aliquoted into culture tubes (5 ml per tube) and the respective compounds were added from DMSO stocks (1:1000) and incubated for 20 minutes at 37 °C, 200 rpm. INH (20 µg/mL) was used as a control to completely inhibit mycolic acid biosynthesis. After pre-incubation with compound, 1 µL of C-14 acetic acid (1 µC/µL in ethanol, *American Radiolabeled* Chemicals) was added to sample and the samples were incubated at 37 °C, 200 rpm. Cells were harvested at different time-points (1.5 mL per time-point) and harvested in micro-centrifuge tubes (7000 xg, 10 min,4 °C). The cells were washed with cold PBS and the Pellet was reconstituted in 300 µL chloroform/methanol (2/1). The samples were vortexed and sonicated in a sonication bath (3x) before adding 100 µL HPLC-grade H_2O and centrifugation for 10 min at 17.000 xg. The organic phase was transferred into glass vials and used for HPTLC. For the HPTLCs, 20 µL of each sample was spotted and chloroform/methanol/water (30/8/1) was used to develop the plates. The HPTLC plates were incubated overnight with phosphor-imaging plates and phosphorescence was imaged using a Typhoon imager (*GE Healthcare*). The spots corresponding to TMMs and TDM were quantified using ImageJ and the background was subtracted before calculating the TDM/TMM ratios. The ratios were normalised to DMSO and the graphs were prepared using Graphpad Prism 10.01. The experiments were conducted in 3 independent replicates. Statistical significance was determined using an ordinary one-way ANOVA (n=3) (comparison to DMSO control).

Expression and purification of Mtb epoxide hydrolases EphF and EphD

Gene fragments were codon-optimized for expression in *E. coli* and synthesised by *Twist Bioscience*. The genes were cloned into pETG41K using gateway cloning. This resulted in fusion genes of MBP-TEV-EpH (see plasmid sequence below). The fusion-proteins were expressed in BL21 DE3 (500 µM IPTG at OD_{600} of 0.6-0.8) at 18°C for 16 hours. Cells were harvested and washed with cold PBS. Cell pellets were reconstituted in lysis buffer (50 mM Hepes pH 7.4, 250 mM NaCl, 1 mM TCEP, 10% glycerol, 0.5 mM PMSF, small amount of DNase) and lysed by sonication (7 min at 30%, 3 min at 60%, 7 min at 30% intensity, Sonopuls HD 2070 ultrasonic rod, *Bandelin electronic GmbH*). The lysate was cleared at 38.000 xg for 45 min and the supernatant was filtered through a 0.45 µm filter prior to purification using an Äkta Pure Protein Purification System (*Cytiva*). Lysate was loaded onto an equilibrated 5 mL HisTrapHP column (*Cytiva*) at a flow-rate of 2 mL/min. The column was first washed with 9.5 column-volumes (CV) of 95% buffer A (50 mM Hepes pH 7.4, 250 mM NaCl, 1 mM TCEP, 10% glycerol) and 5% buffer B (50 mM Hepes pH 7.4, 250 mM NaCl, 1 mM TCEP, 10% glycerol, 500 mM imidazole) at a flow-rate of 5 mL/min and then washed with another 4 CVs of 10% buffer B. The bound proteins were eluted over a gradient of 3 CVs from 10% buffer B to 100% buffer B and another 3 CVs at 100% buffer B. The elution fractions were pooled and loaded onto a 5 mL MBP-Trap column (*Cytiva*). Due to the lower binding capacity of the MBP-trap column, the lysate was loaded and eluted in three separate runs. Each time, the lysate was loaded at a flow-rate of 2 mL/min, washed over 9.5 CVs with 100% buffer A and eluted over 3 CVs with 100% buffer C (50 mM Hepes pH 7.4, 250 mM NaCl, 1 mM TCEP, 10% glycerol, 10 mM maltose). The eluted protein was concentrated, analysed via SDS-PAGE and then further purified by size-exclusion chromatography in buffer A using a Superdex 75pg (*Cytiva*) to remove another co-eluting protein that was identified by SDS-PAGE. MBP-EphD was not active, while MBP-EphF was enzymatically active and could be used for in vitro assays.

pETG41K_ephF

ATCCGGATATAGTTCCTCCTTTCAGCAAAAAACCCCTCAAGACCCGTTTAGAGGCCCCAAGGGGTTATGCTAGTTATTGCTCAGCGGTGGCAGCAGCCAACTCAGCTTCCTTTCGG
GCTTTGTTAGCAGCCGGATCTCAGTGGTGGTGGTGGTGGTGCTCGAGTCACCACTTTGTACAAGaaagctgggtgTCACGTCTCGCGGCGCAAAAAGGCTCTTACACGATCGAGGAC
TAAATCAGGCCGCTGATCTACAATCCAGTGACCGACACCGTCCACCAGTTCAAGCTGAAAATCATCAATCCGTTCTGCGTATCCATCCAGCAAATCCGGAGTCAATACAGGGTCTTC
GGTGCCTGAGAGCCACCGTACGGGGACATGAACACGGGCACCGTCGTATTCACCACGCATCCAAGACAACATTTCTCTGGTCTGAAAGGATCGGTACCATCGAGAACCGGCTTCA
GCATGCCCAGGCTGGCGCATGCACTCTAAATACAGACGCACGTCTTCCTCCGGAAGAGTGTAGCCACCACCCACCCATGATCCCAGTAAGCGAACGAAGCGGGAGTCCGGTGTG
GAGATCACTCGGGGCCCTATCACCGGGAGGCTGATAGGTATCTGATACCAAAAACGCCAGACGTTACGTATCGTAGAGAGACTGCGTTTTACGAATGGAGCACTGGTGTTGACCC
CAAAAAAACCAGTAACTTTTTCCGGATGCCGTAACATCATAATGAAGGCAACCGGGCCACCCCAATCATGAGCCACTAAATCTACGCTTCTTACACCAAGACGATCTAACACTCCC
GCCAAATCATCGGCCATTTCATCCTTACGATAGCTCGATCGCGGGGCGCTTGACCAGCCCGCTCCGCGGAGATCGGGACACAGAACGCGATATCCGTCGGCAGCTAAAGGCCCAA
TTAAGGCGCGCCATTCCCACCAATTCTGGGGAAACCCATGAACTAACATCACCGCCGGGCCCGATGCAGGTCCCGCATCAGCCACGTGGATCGTAACATCATCTCCTAAATCCACA
TAGCGGTGTTCTACACCATCTAATTCGGGCATGGTGACCATgccctgaaaataaagattctcaaagcctgcttTTTGTACAAACTTGTCATGGTACCGCTACCGGGAGTCTGCGCGTCTTTCA
GGGCTTCATCGACAGTCTGACGACCGCTGGCGGCGTTGATCACCGCAGTACGCACGGCATACCAGAAAGCGGACATCTGCGGGATGTTCGGCATGATTTCACCTTTCTGGGCGTT
TTCCATAGTGGCGGCAATACGTGGATCTTTCGCCAACTCTTCCTCGTAAGACTTCAGCGCTACGGCACCCAGCGGTTTGTCTTTATTAACCGCTTCCAGACCTTCATCAGTCAGCAG
ATAGTTTTCGAGGAACTCTTTTGCCAGCTCTTTGTTCGGACTGGCGGCGTTAATACCTGCGCTCAGCACGCCAACGAACGGTTTGGATGGTTGACCCTTGAAGGTCGGCAGTACCG
TTACACCATAATTCACTTTGCTGGTGTCGATGTTGGACCATGCCCACGGGCCGTTGATGGTCATCGCTGTTTCGCCTTTATTAAAGGCAGCTTCTGCGATGGAGTAATCGGTGTCTG
CATTCATGTGTTTGTTTTTAATCAGGTCAACCAGGAAGGTCAGACCCGCTTTCGCGCCAGCGTTATCCACGCCCACGTCTTTAATGTCGTACTTGCCGTTTTCATACTTGAACGCATA
ACCCCCGTCAGCAGCAATCAGCGGCCAGGTGAAGTACGGTTCTTGCAGGTTGAACATCAGCGCGCTCTTACCTTTCGCTTTCAGTTCTTTATCCAGCGCCGGGATCTCTTCCCAGGT
TTTTGGCGGGTTCGGCAGCAGATCTTTGTTATAAATCAGCGATAACGCTTCAACAGCGATCGGGTAAGCAATCAGCTTGCCGTTGTAACGTACGGCATCCCAGGTAAACGGATAC
AGCTTGTCCTGGAACGCTTTGTCCGGGGTGATTTCAGCCAACAGGCCAGATTGAGCGTAGCCACCAAAGCGGTCGTGTGCCCAGAAGATAATGTCAGGGCCATCGCCAGTTGCCG
CAACCTGTGGGAATTTCTCTTCCAGTTTATCCGGATGCTCAACGGTGACTTTAATTCCGGTATCTTTCTCGAATTTCTTACCGACTTCAGCGAGACCGTTATAGCCTTTATCGCCGTTA
ATCCAGATTACCAGTTTACCTTCTTCGATTTTCATGGGGTGATGGTGATGGTGATGTTTCATGGTATATCTCCTTCTTAAAGTTAAACAAAATTATTTCTAGAGGGGAATTGTTATCC
GCTCACAATTCCCCTATAGTGAGTCGTATTAATTTCGCGGGATCGAGATCTCGATCCTCTACGCCGGACGCATCGTGGCCGGCATCACCGGCGCCACAGGTGCGGTTGCTGGCGCC
TATATCGCCGACATCACCGATGGGGAAGATCGGGCTCGCCACTTCGGGCTCATGAGCGCTTGTTTCGGCGTGGGTATGGTGGCAGGCCCCGTGGCCGGGGGACTGTTGGGCGCC
ATCTCCTTGCATGCACCATTCCTTGCGGCGGCGGTGCTCAACGGCCTCAACCTACTACTGGGCTGCTTCCTAATGCAGGAGTCGCATAAGGGAGAGCGTCGAGATCCCGGACACC
ATCGAATGGCGCAAAACCTTTCGCGGTATGGCATGATAGCGCCCGGAAGAGAGTCAATTCAGGGTGGTGAATGTGAAACCAGTAACGTTATACGATGTCGCAGAGTATGCCGGT
GTCTCTTATCAGACCGTTTCCCGCGTGGTGAACCAGGCCAGCCACGTTTCTGCGAAAACGCGGGAAAAAGTGGAAGCGGCGATGGCGGAGCTGAATTACATTCCCAACCGCGTG
GCACAACAACTGGCGGGCAAACAGTCGTTGCTGATTGGCGTTGCCACCTCCAGTCTGGCCCTGCACGCGCCGTCGCAAATTGTCGCGGCGATTAAATCTCGCGCCGATCAACTGG
GTGCCAGCGTGGTGGTGTCGATGGTAGAACGAAGCGGCGTCGAAGCCTGTAAAGCGGCGGTGCACAATCTTCTCGCGCAACGCGTCAGTGGGCTGATCATTAACTATCCGCTGG
ATGACCAGGATGCCATTGCTGTGGAAGCTGCCTGCACTAATGTTCCGGCGTTATTTCTTGATGTCTCTGACCAGACACCCATCAACAGTATTATTTTCTCCCATGAAGACGGTACGC
GACTGGGCGTGGAGCATCTGGTCGCATTGGGTCACCAGCAAATCGCGCTGTTAGCGGGCCCATTAAGTTCTGTCTCGGCGCGTCTGCGTCTGGCTGGCTGGCATAAATATCTCAC
TCGCAATCAAATTCAGCCGATAGCGGAACGGGAAGGCGACTGGAGTGCCATGTCCGGTTTTCAACAAACCATGCAAATGCTGAATGAGGGCATCGTTCCCACTGCGATGCTGGTT
GCCAACGATCAGATGGCGCTGGGCGCAATGCGCGCCATTACCGAGTCCGGGCTGCGCGTTGGTGCGGATATCTCGGTAGTGGGATACGACGATACCGAAGACAGCTCATGTTAT
ATCCCGCCGTTAACCACCATCAAACAGGATTTTCGCCTGCTGGGGCAAACCAGCGTGGACCGCTTGCTGCAACTCTCTCAGGGCCAGGCGGTGAAGGGCAATCAGCTGTTGCCCG
TCTCACTGGTGAAAAGAAAAACCACCCTGGCGCCCAATACGCAAACCGCCTCTCCCCGCGCGTTGGCCGATTCATTAATGCAGCTGGCACGACAGGTTTCCCGACTGGAAAGCGG
GCAGTGAGCGCAACGCAATTAATGTAAGTTAGCTCACTCATTAGGCACCGGGATCTCGACCGATGCCCTTGAGAGCCTTCAACCCAGTCAGCTCCTTCCGGTGGGCGCGGGGCAT
GACTATCGTCGCCGCACTTATGACTGTCTTCTTTATCATGCAACTCGTAGGACAGGTGCCGGCAGCGCTCTGGGTCATTTTCGGCGAGGACCGCTTTCGCTGGAGCGCGACGATGA
TCGGCCTGTCGCTTGCGGTATTCGGAATCTTGCACGCCCTCGCTCAAGCCTTCGTCACTGGTCCCGCCACCAAACGTTTCGGCGAGAAGCAGGCCATTATCGCCGGCATGGCGGCC
CCACGGGTGCGCATGATCGTGCTCCTGTCGTTGAGGACCCGGCTAGGCTGGCGGGGTTGCCTTACTGGTTAGCAGAATGAATCACCGATACGCGAGCGAACGTGAAGCGACTGC
TGCTGCAAAACGTCTGCGACCTGAGCAACAACATGAATGGTCTTCGGTTTCCGTGTTTCGTAAAGTCTGGAAACGCGGAAGTCAGCGCCCTGCACCATTATGTTCCGGATCTGCAT
CGCAGGATGCTGCTGGCTACCCTGTGGAACACCTACATCTGTATTAACGAAGCGCTGGCATTGACCCTGAGTGATTTTTCTCTGGTCCCGCCGCATCCATACCGCCAGTTGTTTACC
CTCACAACGTTCCAGTAACCGGGCATGTTCATCATCAGTAACCCGTATCGTGAGCATCCTCTCTCGTTTCATCGGTATCATTACCCCCATGAACAGAAATCCCCCTTACACGGAGGC
ATCAGTGACCAAACAGGAAAAAACCGCCCTTAACATGGCCCGCTTTATCAGAAGCCAGACATTAACGCTTCTGGAGAAACTCAACGAGCTGGACGCGGATGAACAGGCAGACAT
CTGTGAATCGCTTCACGACCACGCTGATGAGCTTTACCGCAGCTGCCTCGCGCGTTTCGGTGATGACGGTGAAAACCTCTGACACATGCAGCTCCCGGAGACGGTCACAGCTTGTC
TGTAAGCGGATGCCGGGAGCAGACAAGCCCGTCAGGGCGCGTCAGCGGGTGTTGGCGGGTGTCGGGGCGCAGCCATGACCCAGTCACGTAGCGATAGCGGAGTGTATACTGG
CTTAACTATGCGGCATCAGAGCAGATTGTACTGAGAGTGCACCATATATGCGGTGTGAAATACCGCACAGATGCGTAAGGAGAAAATACCGCATCAGGCGCTCTTCCGCTTCCTC
GCTCACTGACTCGCTGCGCTCGGTCGTTCGGCTGCGGCGAGCGGTATCAGCTCACTCAAAGGCGGTAATACGGTTATCCACAGAATCAGGGGATAACGCAGGAAAGAACATGTG
AGCAAAAGGCCAGCAAAAGGCCAGGAACCGTAAAAAGGCCGCGTTGCTGGCGTTTTTCCATAGGCTCCGCCCCCCTGACGAGCATCACAAAAATCGACGCTCAAGTCAGAGGTG
GCGAAACCCGACAGGACTATAAAGATACCAGGCGTTTCCCCCTGGAAGCTCCCTCGTGCGCTCTCCTGTTCCGACCCTGCCGCTTACCGGATACCTGTCCGCCTTTCTCCCTTCGGG
AAGCGTGGCGCTTTCTCATAGCTCACGCTGTAGGTATCTCAGTTCGGTGTAGGTCGTTCGCTCCAAGCTGGGCTGTGTGCACGAACCCCCCGTTCAGCCCGACCGCTGCGCCTTAT
CCGGTAACTATCGTCTTGAGTCCAACCCGGTAAGACACGACTTATCGCCACTGGCAGCAGCCACTGGTAACAGGATTAGCAGAGCGAGGTATGTAGGCGGTGCTACAGAGTTCTT
GAAGTGGTGGCCTAACTACGGCTACACTAGAAGGACAGTATTTGGTATCTGCGCTCTGCTGAAGCCAGTTACCTTCGGAAAAAGAGTTGGTAGCTCTTGATCCGGCAAACAAACC
ACCGCTGGTAGCGGTGGTTTTTTTGTTTGCAAGCAGCAGATTACGCGCAGAAAAAAAGGATCTCAAGAAGATCCTTTGATCTTTTCTACGGGGTCTGACGCTCAGTGGAACGAAA
ACTCACGTTAAGGGATTTTGGTCATGAACAATAAAACTGTCTGCTTACATAAACAGTAATACAAGGGGTGTTATGAGCCATATTCAACGGGAAACGTCTTGCTCTAGGCCGCGATT
AAATTCCAACATGGATGCTGATTTATATGGGTATAAATGGGCTCGCGATAATGTCGGGCAATCAGGTGCGACAATCTATCGATTGTATGGGAAGCCCGATGCGCCAGAGTTGTTT
CTGAAACATGGCAAAGGTAGCGTTGCCAATGATGTTACAGATGAGATGGTCAGACTAAACTGGCTGACGGAATTTATGCCTCTTCCGACCATCAAGCATTTTATCCGTACTCCTGA
TGATGCATGGTTACTCACCACTGCGATCCCCGGGAAAACAGCATTCCAGGTATTAGAAGAATATCCTGATTCAGGTGAAAATATTGTTGATGCGCTGGCAGTGTTCCTGCGCCGGT
TGCATTCGATTCCTGTTTGTAATTGTCCTTTTAACAGCGATCGCGTATTTCGTCTCGCTCAGGCGCAATCACGAATGAATAACGGTTTGGTTGATGCGAGTGATTTTGATGACGAGC
GTAATGGCTGGCCTGTTGAACAAGTCTGGAAAGAAATGCATAAACTTTTGCCATTCTCACCGGATTCAGTCGTCACTCATGGTGATTTCTCACTTGATAACCTTATTTTTGACGAGG
GGAAATTAATAGGTTGTATTGATGTTGGACGAGTCGGAATCGCAGACCGATACCAGGATCTTGCCATCCTATGGAACTGCCTCGGTGAGTTTTCTCCTTCATTACAGAAACGGCTT
TTTCAAAAATATGGTATTGATAATCCTGATATGAATAAATTGCAGTTTCATTTGATGCTCGATGAGTTTTTCTAAGAATTAATTCATGAGCGGATACATATTTGAATGTATTTAGAAA
AATAAACAAATAGGGGTTCCGCGCACATTTCCCCGAAAAGTGCCACCTGAAATTGTAAACGTTAATATTTTGTTAAAATTCGCGTTAAATTTTTGTTAAATCAGCTCATTTTTTAACC
AATAGGCCGAAATCGGCAAAATCCCTTATAAATCAAAAGAATAGACCGAGATAGGGTTGAGTGTTGTTCCAGTTTGGAACAAGAGTCCACTATTAAAGAACGTGGACTCCAACGT
CAAAGGGCGAAAAACCGTCTATCAGGGCGATGGCCCACTACGTGAACCATCACCCTAATCAAGTTTTTTGGGGTCGAGGTGCCGTAAAGCACTAAATCGGAACCCTAAAGGGAG
CCCCCGATTTAGAGCTTGACGGGGAAAGCCGGCGAACGTGGCGAGAAAGGAAGGGAAGAAAGCGAAAGGAGCGGGCGCTAGGGCGCTGGCAAGTGTAGCGGTCACGCTGCG
CGTAACCACCACACCCGCCGCGCTTAATGCGCCGCTACAGGGCGCGTCCCATTCGCCA

EphF activity assay

The inhibitory of compounds the epoxide hydrolase EphF from *M. tuberculosis* was assayed based on the enzymatic conversion of cis-9,10-epoxystearic acid to 9,10-dihydroxystearic acid. Therefore, 500 nM MBP-EphF in HEPES buffer (20 mM HEPES, 300 mM NaCl, 0.1 mM TCEP, pH 7.4) were combined

with compounds (1:100, 1% DMSO) to a total volume of 100 µL 1.5 mL microcentrifuge tubes. The enzyme was pre-incubated with the compounds at 37°C and 300 rpm for 15 min before 500 µM cis-9,10-epoxystearic acid were added to each reaction. To assay non-enzymatic hydrolysis of the epoxide, heat controls were prepared by heat inactivating the enzyme at 98°C and 350 rpm for 15 min. After substrate addition, the reactions were incubated at 37°C and 300 rpm for 15 min. Then, 1 mL ice-cold chloroform was added to stop the enzymatic reaction and to precipitate the protein. The samples were subsequently vortexed and centrifuged at 12 000 x *g* and 0°C for 10 min to obtain a clear separation between the aqueous and the organic chloroform phase. 900 µL of the chloroform phase was transferred to fresh 1.5 mL low-bind microcentrifuge tubes and the liquid was evaporated at 45°C in a centrifugal vacuum concentrator. The dry residuals were reconstituted in 50 µL of 50% MS-grade ACN in ddH_2O by vortexing and placing them in an ultrasonic bath for 5 min twice. Then, samples were centrifuged at 4°C and 12 000 x *g* for 5 min and 22 µL from each sample were transferred to glass MS vials. The amount of epoxide and diol was determined by mass spectrometry. To account for deviations in LC-MS consistencies, each sample was spiked with 5 µL taurocholic acid (final concentration 45 µM) for normalization during MS analysis. Samples were analysed by LC-MS for separation of the diol and epoxide and relative quantification. LC-MS analysis was performed using an UltiMate 3000 micro HPLC system (*Dionex*) coupled to a QExactive Plus (*Thermo Fischer*) via an ESI ion source. The samples were loaded onto a equilibrated an XBridge BEH300 C4 3.5 µm 2.1 x 150 mm column (*Waters*) and separated over a 15 min gradient from 26% to 90% B, followed by 5 min at 95% before re-equilibration at 26% B 7 minutes. The flow-rate was 200 µL/min. The mobile phases A and B were 0.1 % (v/v) formic acid in water and 0.1% (v/v) formic acid in acetonitrile, respectively. The QExactive plus was operated in negative mode and single ion monitoring (SIM) was performed. For the first 9 minutes of the gradient SIM was set to 499 m/z to 529 m/z to measure the eluting taurocholic acid. For the rest of the gradient, SIM window was set to 295 m/z to 317.5 m/z for monitoring of the eluting diol and any remaining epoxide. Area under the curve (AUC) was determined using XCalibur software (*Thermo Fischer Scientific*) using the GENESIS algorithm. AUCs of the diol were normalised to taurocholic acid for relative quantification. All experiments were conducted in at least 5 independent replicated with 2 technical replicates each. Values were normalised to the DMSO control and graphs were plotted using *Graphpad* Prism 10.01. Statistical significance of inhibition (compared to DMSO) was calculated using ordinary one-way ANOVA.

MTT Cytotoxicity Assay

HeLa cells were seeded at a density of 4000 cells per well in a transparent, flat-bottomed 96-well plate (200 µL medium per well). Cells were grown overnight in a humidified atmosphere at 37 °C and 5% CO2 to allow the cells to adhere to the surface. Subsequently, the medium was aspirated and replaced by fresh medium supplemented with compound in concentrations ranging from 2 µM to 500 µM (DMSO content less than 1%) or 1% DMSO as a control. The cells were incubated at 37 °C, 5% CO_2 for 24 h. For the determination of metabolic activity, 20 µL 3-(4,5-dimethyl-2-thiazolyl)-2,5-diphenyl-2H-tetrazolium bromide solution (MTT, 5mg/mL in PBS) were added to each well and the cells were incubated at 37 °C, 5% CO2 for 4 h. Thereafter, the medium was aspirated and the violet formazan crystals were dissolved in 200 µL DMSO per well under shaking (300 r.p.m., 25 min). Absorbance at 570 nm with a reference wavelength of 630 nm was recorded using an Infinite F200 pro plate reader (*Tecan*). Four biological replicates, each consisting of 3 technical replicates, were measured for each data point. Cell viability was normalized with respect to the DMSO control and fitted fitted as

log(inhibitor) vs. response variable slope (four parameters) non-linear regression using *GraphPad* Prism 10.01. Cytotoxicity is reported as the IC50 value, the concentration at which 50% viability is reached.

Drug susceptibility testing in MmpL3 under/over-expressing *M. tuberculosis*

Drug susceptibility assays with WT *M. tuberculosis* H37Rv and the *mmpl3*-TetON strains were performed as described by Grover et al.[13] In short, WT cells were grown in 10 mL 7H9 (10% ADNaCl, 0.2% glycerol, 0.05% tyloxapol) and mutant cells in 7H9 (+25 μg/mL Kanamycin, +25 μg/mL Zeocin, and +500 ng/mL anhydrotetracycline [“ATc”] for *mmpL3*-TetON)) in a 25-cm^2 tissue culture flask with a vented cap. Medium was inoculated with 1:10 with a thawed glycerol stock, incubated for approx. 7 d at 37 °C and 5% CO_2 in a humid incubator, and grown to an OD_{580} of approx. 1. The cultures were pelleted and washed with 7H9 and were diluted to OD_{580} = 0.01 in 10 mL 7H9 (plus selection antibiotics but +/- ATc for *mmpL3*-TetON) and were incubated for 14 d at 37 °C and 5% CO_2 in a humid incubator, with passage to OD_{580} = 0.01 in 10 mL 7H9 (same conditions) at day 7. The cultures were pelleted and washed with fresh 7H9 and were diluted to OD_{580} = 0.01 in an appropriate volume of 7H9 for assay requirements (+/- ATc as appropriate). The diluted cultures were added to assay-ready 384-well plates at 50 μL/well. The flat-bottom 384-well microplates (*Greiner* Bio-One) were prepared by dispensing nanoliter volume of drugs into 2-fold dilutions with 14 doses per drug using an automated drug dispenser (D300e Digital Dispenser, *HP*). The plates were wrapped with aluminum foil in stacks of no more than six and incubated at 37 °C and 5% CO_2 in a humid incubator. The OD_{580} of the plates was read on day 8 of incubation. The OD_{580} values were normalized to the DMSO control (100% growth) and plotted using *Graphpad* Prism 10.01. The curves were fitted as log(inhibitor) vs. response variable slope (four parameters) non-linear regression, with bottom and top parameters constrained to 0 and 100, respectively. MIC-values were calculated using the Gompertz model[14].

Isolation of 227-resistant mutants

M. tuberculosis H37Rv was grown to an OD_{580} of approx. 0.8 to get an inoculum about 10^8 bacteria. The culture was serially diluted and lower dilutions were used to enumerate the cell titer. Dilutions with higher titer were plate on 7H10 agar plates containing various concentrations of **227**. Frequency of resistance was calculated as the number of colonies at a concentration divided by the number of cells in the inoculum. In total, Eight colonies emerged (2 at 8 µM, 6 at 4 µM), were inoculated into 7H9 (+10% ADNaCl), and grown to an OD_{580} of approx. 0.8. The level of resistance was determined by susceptibility testing as described above. For whole-genome-sequencing (WGS), genomic DNA was isolated, sequencing libraries prepared, and sequencing was performed and results analysed as previously published[15].

Pharmacokinetics studies

CD-1 female mice (22-25 g) were used in oral pharmacokinetic studies. All animal studies were ethically reviewed and carried out in accordance with European Directive 2010/63/EU and the GSK Policy on the Care, Welfare and Treatment of Animals. Compounds for oral snapshot pharmacokinetic (PK) profiling were administered by oral gavage as 25mg/kg in a vehicle consisting of 5% Dimethylacetamice

(DMA), and 95% (20% Solutol HS15 in water). Aliquots of 30-40µL of blood were taken by puncture of the lateral tail vein from each mouse (n = 2 per compound) at 30 minutes, 1, 3, and 5 hours post-dose. Intravenous (IV) PK was performed at 5mg/kg dose in 4% Cremophor EL. Blood timepoints were taken from each mouse (n = 3 per route and dose) at 5 minute, 15 minute, 1, 3, 7, and 24 hours post dose. Dose escalation oral PK profiling of **21** formulated in a suspension with 20% Solutol HS15 or in solution with 100% PEG400 vehicle was performed after 3 QD doses of 50, 100, and 200 mg/kg and 400 mg/kg . Blood timepoints were taken from each mouse (n = 3 per route and dose) at 30 minute, 1, 3,5, 7, and 24 hours post dose. Blood was captured in CB300 blood collection tubes containing K_2EDTA and stored on ice. Plasma was recovered after centrifugation and stored at -80°C until analyzed by high pressure liquid chromatography coupled to tandem mass spectrometry. Pharmacokinetic parameters were determined using non-compartmental pharmacokinetic analysis supported by the PK Solver Excel add-in.

LC-MS/MS analytical methods for PK studies

Neat 1mg/mL DMSO stock of compounds were serial diluted in 50/50 Acetonitrile (ACN)/ Milli-Q water to create standard curves solutions. Standards were created by adding 10µLs of spiking solutions to 90µLs of drug free plasma (CD-1 K2EDTA Mouse, Bioreclamation IVT). 10µLs of control, standard, or study sample were added to 100 µLs of ACN protein precipitation solvent containing 100ng/mL of the internal standards Labetalol (Sigma Aldrich). Extracts were vortexed for 5 minutes and centrifuged at 4000 RPM for 5 minutes. 75 µLs of supernatant was transferred for HPLC-MS/MS analysis and diluted with 75 µLs of Milli-Q deionized water.

LC-MS/MS analysis was performed on a Sciex Applied Biosystems Qtrap 6500+ triple-quadrupole mass spectrometer coupled to a Shimadzu Nexera X2 UHPLC system to quantify each drug in plasma. Chromatography was performed on an Agilent SB-C8 (2.1x30 mm; particle size, 3.5µm) using a reverse phase gradient. Milli-Q deionized water with 0.1% formic acid was used for the aqueous mobile phase and 0.1% formic acid in ACN for the organic mobile phase. Multiple-reaction monitoring of parent/daughter transitions in electrospray negative-ionization mode was used to quantify the analytes. The following MRM transitions were used for **227** (319.98/194.40), **21** (362.96/193.90), **22** (377.02/193.80), and Labetalol (327.20/176.00). Sample analysis was accepted if the concentrations of the quality control samples were within 20% of the nominal concentration. Data processing was performed using Analyst software (version 1.6.2; Applied Biosystems Sciex).

Metabolic stability in mouse liver microsomes (MLM)

50mM stocks of compound **21** in DMSO were prepared and diluted in 50/50 ACN/H2O to a final concentration of 500µM. 100 µL of 200 µM sodium phosphate buffer, 20 µL of 50mM MgCl2, 10 µL of 20 mg/mL mouse liver microsomes (Xenotech), 48 µL of Milli-Q Water, and 2 uL of 500 µM compound stock were added together. The mixture was allowed to incubate at 37 °C while mixing at 300 RPM uncovered on a thermomixer. After 5 minutes of incubation, 20 µL of 10 mM NADPH was added to activate microsomal metabolism or 20 µL or Milli-Q water was added to the no-NADPH controls and samples were mixed by gentle aspiration and dispense. Timepoints were taken at 0, 5, 15, 30, and 45 minutes. At each timepoint 10 µL of mixture was pipetted into 100 µL of ACN containing 100 ng/mL Labetalol to quench the reaction and to extract the compounds. Extracts were vortexed for 5 minutes at 1000 RPM and subsequently centrifuged at 4000 RPM for 5 minutes. 75 µL of extract was combined with 75 µL of Milli-Q water for HPLC-MS analysis. HPLC-MS was performed as previously specified.

Analyst software was used to measure peak areas. The peak area ratio (Analyte/IS) vs time was plotted vs time and a half-life ($t_{1/2}$) was determined using PK solver Excel add-in. The intrinsic clearance (Clint) was calculated accordingly as Clint = microsomal protein concentration (V) * 0.693 / $t_{1/2}$.

Molecular Docking MmpL3

We used the crystal structure 6AJH from the pdb[16], which contained MMPL3 in complex with the compound AU1235. In this structure, some disordered regions were not resolved. For completeness, we used the software MODELER to model these unresolved regions[17,18]. However, these regions were far from the binding pockets and did not have any influence on our docking approach. We defined the docking region as a volume around the cavity where the ligand in the crystal structure was resolved. We did not investigate any other potential binding pockets, e.g., at the surface of the protein. For the definition of the docking box, we used the Python Molecule Viewer from the software package MGLTools[19]. We prepared three compounds of interest for the docking, as listed in **FIG XX**. To this end, we prepared the molecules in the software Avogadro[20] and minimized the prepared 3D conformation with openbabel[21]. For subsequent processing, we converted the files into a pdbqt format with openbabel. We used the software autodock-vina for the docking[22,23] [7,8. In the process, we generated 100 modes per ligand, used an exhaustiveness of 32 and an energy range of 6. As a result, we obtained up to 20 docking poses, per ligand. In **FIG XX**, we show the best 3 poses per ligand.

Chemical Synthesis

General synthesis methods and materials

All reagents and solvents were purchased in at least reagent grade from commercial suppliers (Thermo Fisher Scientific Inc., Merck KGaA, Alfa Aesar, Roth, VWR International, Enamine) and were used without further purification. All air and/or moisture sensitive reactions were conducted under argon atmosphere in flame dried glassware. For analytical thin-layer chromatography (TLC) silica gel 60 F-254 precoated aluminium sheets supplied by Merck KGaA were used. The spots were visualized using UV light (λ=254 nm and 366 nm) or a $KMnO_4$-stain (1.50 g $KMnO_4$, 10.0 g K_2CO_3, 1.25 mL NaOHaq (10 wt-%), 200 mL ddH2O). Flash chromatography was performed Silica gel 60 (particle size = 40–63 µM) from Merck KGaA with compressed air. NMR spectra were recorded on Avance-III (AV-HD300, AV-HD400 or AV-HD500) NMR systems (Bruker Co.) with deuterated chloroform ($CDCl_3$), dimethylsulfoxid (DMSO-d_6) or methanol (MeOH-d_4) as solvents and referenced to the residual signal of the corresponding deuterated solvent (^{1}H: $CDCl_3$: □ = 7.26 ppm, DMSO-d_6: □ = 2.50 ppm, MeOH-d_4: □ = 3.31 ppm; ^{13}C: $CDCl_3$: □ = 77.16 ppm, DMSO-d_6: □ = 39.52 ppm, MeOH-d_4: □ = 49.0 ppm). Chemical shifts (□□□are reported in parts per million (ppm) and coupling constants (J) are reported in Hertz (Hz). Peak multiplicity was assigned using the following abbreviations: s = singlet, br s = broad singlet, d = doublet, t = triplet, q = quartet, p = pentet, sept = septet, m = multiplet or unresolved. Reversed phase high performance liquid chromatography (RP-HPLC) for analytical purposes was performed on a Waters 2695 separations module with a Waters 2996 Photodiode Array Detector at wavelengths between 210 and 600 nm with mobile phases A: H_2O + 0.5% TFA, B: MeCN + 0.5% TFA, C: H_2O, or D: MeCN. Separation was performed on a C18 column (Waters XBridge C18, 3.5 µm, 4.6 x 100 mm). Preparative reversed phase HPLC runs were conducted on a Waters system with a Waters 2545 Quaternary Gradient Module, a Waters 2998 Photodiode Array Detector and a Waters Fraction Collector III with the same mobile phases as in the analytical setup. As stationary phases a Waters XBridge prep C18 OBDTM, 30 x 150 mm, 5 µm was used. High resolution mass spectra were recorded using an LTQ-FT Ultra (Thermo Fisher Scientific Inc.) coupled with a Dionex UltiMate 3000 HPLC system and an ESI ion source.

Synthesis of compounds in order of appearance

Original Hit

1-(4-chloro-3-(trifluoromethyl)phenyl)-3-cyclohexylurea (227)

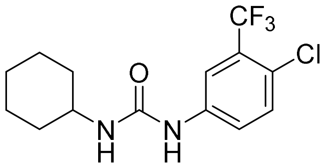

$C_{14}H_{16}ClF_3N_2O$
320.74 g/mol

Cyclohexylamine (175 µL, 150 mg, 1.51 mmol, 1.1 eq.) was dissolved in 10 mL dry dichloromethane (DCM) and 1-chloro-4-isocyanato-2-(trifluoromethyl)benzene (305 mg, 1.37 mmol, 1.0 eq.) was added. The solution was stirred at room temperature overnight. The resulting precipitate was filtered off, rinsed with cold DCM and dried under reduced pressure to yield the desired product (195 mg, 606 µmol, 44%) as a white powder.

^{1}H-NMR (400 MHz, DMSO-d_6): δ [ppm] = 8.78 (s, 1H), 8.06 (d, *J* = 2.2 Hz, 1H), 7.57-7.45 (m, 2H), 6.24 (d, *J* = 7.8 Hz, 1H), 3.52-3.43 (m, 1H, overlapping with the residual water signal), 1.85-1.75 (m, 2H), 1.70-1.61 (m, 2H), 1.58-1.49 (m, 1H), 1.36-1.24 (m, 2H), 1.24-1.11 (m, 3H).
^{13}C-NMR (101 MHz, DMSO-d_6): δ [ppm] = 154.1, 140.2, 131.9, 126.6 (q, *J* = 30.4 Hz), 124.3, 122.6, 121.3, 116.0 (q, *J* = 5.8 Hz), 47. 9, 32.8, 25.2, 24.4.
ESI-HR-MS (m/z) (M-H$^+$) calcd. for $C_{14}H_{15}ClF_3N_2O^-$, 319.0830; found, 319.0830.

Derivatives

1-(4-chloro-3-(trifluoromethoxy)phenyl)-3-cyclohexylurea (1)

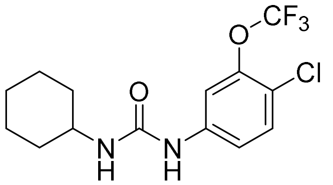

$C_{14}H_{16}ClF_3N_2O_2$
336.74 g/mol

4-Chloro-3-(trifluoromethoxy)aniline (400 mg, 1.89 mmol, 1.0 eq.) was dissolved in DCM (10 mL) and cyclohexyl isocyanate (237 mg, 1.89 mmol, 1.0 eq.) was added. The solution was stirred overnight at room temperature. The volatile components were removed under reduced pressure. The residue was dissolved in dimethylformamide (DMF, 40 mL) and water (80 mL) was added. The resulting precipitate was filtered off and washed with water to yield the product (435 mg, 1.29 mmol, 68%) as a white solid.

TLC (hexane/ethyl acetate = 3/7): R_f = 0.21 [UV]
^{1}H-NMR (500 MHz, DMSO-d_6): δ [ppm] = 8.74 (s, 1H), 7.91-7.85 (m, 1H), 7.48 (d, *J* = 8.8 Hz, 1H), 7.19 (dd, *J* = 8.9, 2.5 Hz, 1H), 6.22 (d, *J* = 7.8 Hz, 1H), 3.52-3.39 (m, 1H), 1.83-1.74 (m, 2H), 1.65 (dt, *J* = 12.7, 3.7 Hz, 2H), 1.59-1.51 (m, 1H), 1.35-1.08 (m, 5H).

^{13}C-NMR (101 MHz, DMSO-d_6): [ppm] = 154.0, 144.0, 141.1, 130.6, 117.4, 116.4, 111.1, 47.8, 32.8, 25.2, 24.3.
ESI-HR-MS (m/z) (M-H$^+$) calcd. for $C_{14}H_{15}ClF_3N_2O_2^-$, 335.0780; found, 335.0780.

1-(5-chloro-4-(trifluoromethyl)pyridin-2-yl)-3-cyclohexylurea (2)

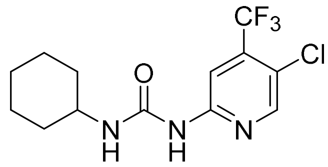

$C_{13}H_{15}ClF_3N_3O$
321.73 g/mol

Cyclohexyl isocyanate (318 mg, 2.54 mmol, 1.0 eq.) and DIPEA (487 µL, 362 mg, 2.80 mmol, 1.1 eq.) were dissolved in DCM (10 mL) and 5-chloro-4-(trifluoromethyl)pyridin-2-amine hydrochloride (500 mg, 2.54 mmol, 1.0 eq.) was added. The reaction was stirred at room temperature overnight. The volatile components were removed under reduced pressure. Purification of the crude product by flash chromatography (hexane/ethyl acetate = 8/2) yielded the product (62 mg, 192 µmol, 8%)

TLC (hexane/ethyl acetate = 3/7): R_f = 0.18 [UV] **^{1}H-NMR** (400 MHz, DMSO-d_6): [ppm] = 9.45 (s, 1H), 8.50 (s, 1H), 8.22 (s, 1H), 7.07 (d, *J* = 7.6 Hz, 1H), 3.58-3.45 (m, 1H), 1.87-1.76 (m, 2H), 1.71-1.59 (m, 2H), 1.56-1.48 (m, 1H), 1.40-1.10 (m, 5H). **^{13}C-NMR** (101 MHz, DMSO-d_6): [ppm] = 153.2, 152.9, 149.1, 135.12 (q, *J* = 31.9 Hz), 123.0, 118.1, 109.0 (q, *J* = 5.5 Hz), 47.7, 32.6, 25.1, 24.1. **ESI-HR-MS** (m/z) (M-H$^+$) calcd. for $C_{13}H_{14}ClF_3N_3O^-$, 320.0783; found, 320.0783.

1-(4-chloro-3-(trifluoromethyl)phenyl)-3-(1-methylcyclopentyl)urea (3)

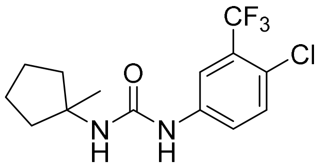

$C_{14}H_{16}ClF_3N_2O$
320.74 g/mol

To dry DCM (10 mL) 1-methyl-1-cyclopentylamine hydrochloride (202 mg, 1.49 mmol, 1.1 eq.), Na_2CO_3 (144 mg, 1.35 mmol, 1.0 eq.) and 1-chloro-4-isocyanato-2-(trifluoromethyl)benzene (300 mg, 1.35 mmol, 1.0 eq.) were added. The reaction was stirred at room temperature overnight and water (10 mL) was added. The precipitate was filtered off and washed with DCM to yield urea **3** (124 mg, 387 µmol, 29%) as a white solid.

^{1}H-NMR (400 MHz, DMSO-d_6): [ppm] = 8.72 (s, 1H), 8.07 (d, *J* = 2.5 Hz, 1H), 7.59-7.33 (m, 2H), 6.22 (s, 1H), 1.96-1.86 (m, 2H), 1.72-1.49 (m, 6H), 1.36 (s, 3H).

^{13}C-NMR (101 MHz, DMSO-d_6): [ppm] = 154.6, 140.7, 132.3, 127.1 (q, *J* = 30.3 Hz), 124.7, 122.5, 122.0, 121.5 (d, *J* = 1.7 Hz), 116.2 (q, *J* = 5.7 Hz), 60.4, 26.1, 23.7.
ESI-HR-MS (m/z) (M-H$^+$) calcd. for $C_{14}H_{15}ClF_3N_2O^-$, 319.0830; found, 319.0830.

1-(4-fluoro-3-(trifluoromethyl)phenyl)-3-(1-methylcyclopentyl)urea (4)

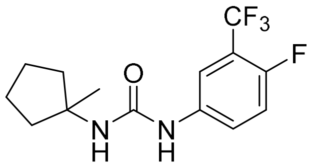

$C_{14}H_{16}F_4N_2O$
304.29 g/mol

1-Methyl-1-cyclopentylamine hydrochloride (255 mg, 1.88 mmol, 1.1 eq.), Na_2CO_3 (181 mg, 1.71 mmol, 1.0 eq.) and 1-fluoro-4-isocyanato-2-(trifluoromethyl)benzene (350 mg, 1.71 mmol, 1.0 eq.) were added to DCM (10 mL). The reaction was stirred overnight at room temperature. Water (10 mL) was added and the mixture was stirred for 1 h at room temperature. The precipitate was filtered off and washed with DCM to yield urea **4** (170 mg, 559 µmol, 33%) as a white solid.

^{1}H-NMR (400 MHz, DMSO-d_6): [ppm] = 8.63 (s, 1H), 7.97 (dd, *J* = 6.5, 2.6 Hz, 1H), 7.49-7.42 (m, 1H), 7.33 (t, *J* = 9.7 Hz, 1H), 6.17 (s, 1H), 1.96-1.88 (m, 2H), 1.71-1.48 (m, 6H), 1.36 (s, 3H).
^{13}C-NMR (101 MHz, DMSO-d_6): [ppm] = 154.8, 154.6, 152.2 (d, *J* = 2.2 Hz), 138.0 (d, *J* = 2.5 Hz), 127.2, 124.5, 123.4 (d, *J* = 7.7 Hz), 121.8, 119.1, 117.8 (d, *J* = 21.2 Hz), 116.7 (qd, *J* = 31.9, 12.7 Hz), 115.2 (q, *J* = 5.0 Hz), 60.3, 26.1, 23.7.
ESI-HR-MS (m/z) (M-H$^+$) calcd. for $C_{14}H_{15}F_4N_2O^-$, 303.1126; found, 303.1126.

1-(1-methylcyclopentyl)-3-phenylurea (5)

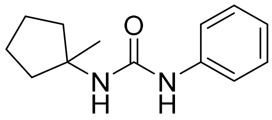

$C_{13}H_{18}N_2O$
218.30 g/mol

1-Methyl-1-cyclopentylamine hydrochloride (313 mg, 2.31 mmol, 1.1 eq.) and Na_2CO_3 (222 mg, 2.10 mmol, 1.0 eq.) and phenyl isocyanate (222 mg, 2.10 mmol, 1.0 eq.) were added to DCM (10 mL). The reaction was stirred overnight at room temperature, water (10 mL) was added and the mixture was stirred for another 1 h at room temperature. The phases were separated and the volatile components of the organic phase were removed under reduced pressure. The residue was purified by flash column chromatography (DCM/MeOH = 98/2) to yield the desired product (116 mg, 531 µmol, 25%) as a white solid.

TLC (DCM/MeOH = 98/2): R_f = 0.09 [UV]

^{1}H-NMR (400 MHz, DMSO-d$_6$): δ [ppm] = 8.20 (s, 1H), 7.34 (dd, *J* = 8.6, 1.0 Hz, 2H), 7.26-7.15 (m, 2H), 6.93-6.73 (m, 1H), 6.04 (s, 1H), 1.97-1.86 (m, 2H), 1.73-1.58 (m, 4H), 1.57-1.48 (m, 2H), 1.36 (s, 3H).
^{13}C-NMR (101 MHz, DMSO-d$_6$): δ [ppm] = 154.9, 141.1, 129.1, 121.2, 117.8, 60.2, 39.5, 26.3, 23.7.
ESI-HR-MS (m/z) (M-H$^+$+FA) calcd. for $C_{14}H_{19}N_2O_3^-$, 263.1401; found, 263.1401.

1-(1-methylcyclopentyl)-3-(2,3,4-trifluorophenyl)urea (6)

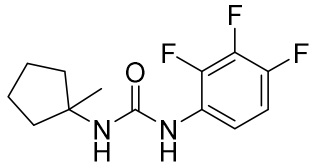

$C_{13}H_{15}F_3N_2O$
272.27 g/mol

1-Methyl-1-cyclopentylamine hydrochloride (259 mg, 1.91 mmol, 1.1 eq.), Na_2CO_3 (181 mg, 1.73 mmol, 1.0 eq.) and 2,3,4-trifluorophenyl isocyanate (300 mg, 1.73 mmol, 1.0 eq.) were added to dry DCM (10 mL). The reaction was stirred overnight at room temperature, water (10 mL) was added and the mixture was stirred for 1 h at room temperature. The phases were separated and the organic phase was washed with brine (1×10 mL). The volatile organic components were removed under reduced pressure. The residue was purified by flash column chromatography (hexane/ethyl acetate = 97/3→8/2) to yield the desired product (215 mg, 790 µmol, 46%) as a white solid.

TLC (hexane/ethyl acetate = 8/2): R$_f$ = 0.37 [UV]
^{1}H-NMR (400 MHz, DMSO-d$_6$): δ [ppm] = 8.23 (d, *J* = 1.8 Hz, 1H), 7.97-7.89 (m, 1H), 7.19-7.06 (m, 1H), 6.58 (s, 1H), 1.95-1.82 (m, 2H), 1.72-1.47 (m, 6H), 1.35 (s, 3H).
^{13}C-NMR (101 MHz, DMSO-d$_6$): δ [ppm] = 154.3, 145.9 (dd, *J* = 9.8, 2.2 Hz), 143.5 (dd, *J* = 9.8, 2.2 Hz), 142.3 (dd, *J* = 11.7, 3.0 Hz), 140.6 (dd, *J* = 16.3, 13.8 Hz), 139.8 (dd, *J* = 11.7, 3.0 Hz), 138.2 (dd, *J* = 16.3, 13.8 Hz), 127.1 (dd, *J* = 7.8, 2.9 Hz), 114.3 (dt, *J* = 6.1, 3.4 Hz), 111.8 (dd, *J* = 17.2, 3.6 Hz), 60.3, 39.4, 26.0, 23.7.
ESI-HR-MS (m/z) (M-H$^+$) calcd. for $C_{13}H_{14}F_3N_2O^-$, 271.1064; found, 271.1064.

1-(3,4-difluorophenyl)-3-(1-methylcyclopentyl)urea (7)

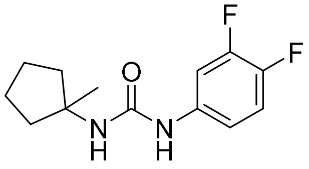

$C_{13}H_{16}F_2N_2O$
254.28 g/mol

1-Methyl-1-cyclopentylamine hydrochloride (240 mg, 1.77 mmol, 1.1 eq.), Na_2CO_3 (171 mg, 1.61 mmol, 1.0 eq.) and 3,4-difluorophenyl isocyanate (250 mg, 1.61 mmol, 1.0 eq.) were

added to dry DCM (10 mL). The reaction was stirred overnight at room temperature. Water (10 mL) was added and the mixture was stirred for 1 h at room temperature. The phases were separated the volatile organic compounds were removed under reduced pressure. The residue was purified by flash column chromatography (DCM/MeOH = 1/0→95/5) to yield the desired product (310 mg, 1.22 mmol, 76%) as a white solid.

TLC (hexane/ethyl acetate = 2/1): R_f = 0.16 [UV]
^{1}H-NMR (500 MHz, DMSO-d_6): δ [ppm] = 8.44 (s, 1H), 7.70-7.56 (m, 1H), 7.25 (q, *J* = 9.2 Hz, 1H), 7.00-6.84 (m, 1H), 6.12 (s, 1H), 1.93-1.85 (m, 2H), 1.70-1.56 (m, 4H), 1.56-1.48 (m, 2H), 1.35 (s, 3H).
^{13}C-NMR (126 MHz, DMSO-d_6): δ [ppm] = 154.7, 150.5 (d, *J* = 13.0 Hz), 148.6 (d, *J* = 13.0 Hz), 145.1 (d, *J* = 12.8 Hz), 143.2 (d, *J* = 12.8 Hz), 138.3 (dd, *J* = 9.5, 2.4 Hz), 117.7 (d, *J* = 17.6 Hz), 113.6 (dd, *J* = 5.6, 3.1 Hz), 106.5 (d, *J* = 22.0 Hz), 60.3, 39.4, 26.2, 23.7.
ESI-HR-MS (m/z) (M-H$^+$) calcd. for $C_{13}H_{15}F_2N_2O^-$, 253.1158; found, 253.1158.

1-(1-methylcyclopentyl)-3-(3,4,5-trifluorophenyl)urea (8)

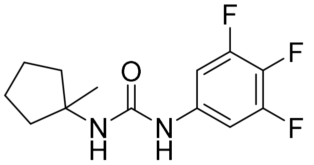

$C_{13}H_{15}F_3N_2O$
272.27 g/mol

To DCM (10 mL) 1-Methyl-1-cyclopentylamine hydrochloride (259 mg, 1.91 mmol, 1.1 eq.) and Na_2CO_3 (184 mg, 1.73 mmol, 1.0 eq.) were added. 3,4,5-trifluorophenyl isocyanate (300 mg, 1.73 mmol, 1.0 eq.) were added and the reaction was stirred overnight at room temperature. After the addition of water (10 mL) the mixture was stirred for another 1 h at room temperature. The phases were separated and the aqueous phase was extracted with DCM (2×10 mL). The organic phases were pooled and the volatile organic components were removed under reduced pressure. The residue was purified by flash column chromatography (hexane/ethyl acetate = 8/2) to yield the desired product (222 mg, 815 µmol, 47%) as a white solid.

TLC (hexane/ethyl acetate = 8/2): R_f = 0.31 [UV]
^{1}H-NMR (400 MHz, DMSO-d_6): δ [ppm] = 8.54 (s, 1H), 7.24 (dd, *J* = 10.8, 6.4 Hz, 2H), 6.19 (s, 1H), 1.95-1.85 (m, 2H), 1.70-1.56 (m, 4H), 1.55-1.48 (m, 2H), 1.34 (s, 3H).
^{13}C-NMR (101 MHz, DMSO-d_6): δ [ppm] = 157.7, 154.5, 151.9 (dd, *J* = 9.9, 5.7 Hz), 149.5 (dd, *J* = 9.9, 5.7 Hz), 137.6 (td, *J* = 12.2, 3.2 Hz), 134.7 (t, *J* = 15.8 Hz), 132.3 (t, *J* = 15.9 Hz), 101.6 (d, *J* = 24.1 Hz), 60.3, 39.4, 26.0, 23.7.
ESI-HR-MS (m/z) (M-H$^+$) calcd. for $C_{13}H_{14}F_3N_2O^-$, 271.1064; found, 271.1063.

1-(4-chloro-3-(trifluoromethyl)phenyl)-3-cyclopentylurea (9)

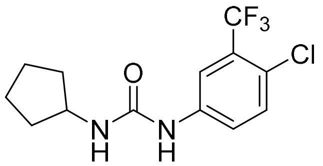

$C_{13}H_{14}ClF_3N_2O$
306.71 g/mol

Cyclopentylamine (148 mg, 1.74 mmol, 1.1 eq.) was dissolved in dry DCM (10 mL) and 1-chloro-4-isocyanato-2-(trifluoromethyl)benzene (350 mg, 1.58 mmol, 1.0 eq.) was added. The reaction was stirred overnight at room temperature. The precipitate was filtered off to yield the compound **9** (263 mg, 907 µmol, 57%) as a white solid.

^{1}H-NMR (400 MHz, DMSO-d$_6$): δ [ppm] = 8.74 (s, 1H), 8.07 (s, 1H), 7.53 (s, 2H), 6.34 (d, *J* = 7.1 Hz, 1H), 3.94 (h, *J* = 6.7 Hz, 1H), 1.89-1.79 (m, 2H), 1.70-1.48 (m, 4H), 1.45-1.33 (m, 2H).
^{13}C-NMR (101 MHz, DMSO-d$_6$): δ [ppm] = 154.9, 140.6, 132.3, 127.1 (q, *J* = 30.5 Hz), 124.7, 122.7, 122.0, 121.7 (d, *J* = 1.7 Hz), 116.5 (q, *J* = 5.8 Hz), 51.5, 33.2, 23.6.
ESI-HR-MS (m/z) (M-H$^+$) calcd. for $C_{13}H_{13}ClF_3N_2O^-$, 305.0674; found, 305.0674.

1-(4-chloro-3-(trifluoromethyl)phenyl)-3-cyclobutylurea (10)

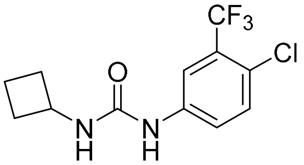

$C_{12}H_{12}ClF_3N_2O$
292.69 g/mol

Cyclobutylamine (124 mg, 1.74 mmol, 1.1 eq.) was dissolved in DCM (10 mL) and 1-chloro-4-isocyanato-2-(trifluoromethyl)benzene (350 mg, 1.58 mmol, 1.0 eq.) was added. The solution was stirred overnight at room temperature, and the precipitate was filtered off to yield the desired product (215 mg, 778 µmol, 49%) as a white powder.

^{1}H-NMR (400 MHz, DMSO-d$_6$): δ [ppm] = 8.81 (s, 1H), 8.05 (d, *J* = 2.0 Hz, 1H), 7.64-7.49 (m, 2H), 6.60 (d, *J* = 7.9 Hz, 1H), 4.18-4.07 (m, 1H), 2.23-2.15 (m, 2H), 1.94-1.83 (m, 2H), 1.68-1.53 (m, 2H).
^{13}C-NMR (101 MHz, DMSO-d$_6$): δ [ppm] = 154.3, 140.5, 132.3, 127.0 (q, *J* = 30.4 Hz), 124.7, 122.9, 122.0, 121.9 (d, *J* = 1.7 Hz), 116.7 (q, *J* = 5.4 Hz), 45.1, 31.2, 14.9.
ESI-HR-MS (m/z) (M-H$^+$) calcd. for $C_{12}H_{11}ClF_3N_2O^-$, 291.0517; found, 291.0517.

1-(4-chloro-3-(trifluoromethyl)phenyl)-3-cyclopropylurea (11)

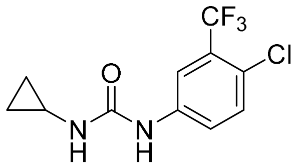

$C_{11}H_{10}ClF_3N_2O$
278.66 g/mol

Cyclopropylamine (113 mg, 1.99 mmol, 1.1 eq.) was dissolved in DCM (10 mL) and 1-chloro-4-isocyanato-2-(trifluoromethyl)benzene (400 mg, 1.81 mmol, 1.0 eq.) was added. The reaction was stirred overnight at room temperature and the precipitate was filtered off. Purification by flash column chromatography (DCM/MeOH = 98/2) yielded the desired product (89.8 mg, 322 µmol, 18%) as a white solid.

TLC (DCM/MeOH = 95/5): R_f = 0.26 [UV]
^{1}H-NMR (400 MHz, DMSO-d_6): [ppm] = 8.80 (s, 1H), 8.09 (d, *J* = 2.5 Hz, 1H), 7.65-7.50 (m, 2H), 6.62 (s, 1H), 0.68-0.60 (m, 2H), 0.46-0.37 (m, 2H).
^{13}C-NMR (101 MHz, DMSO-d_6): [ppm] = 156.2, 140.5, 132.3, 127.6-126.3 (m), 124.7, 123.1, 122.0, 116.9 (q, *J* = 5.9, 5.2 Hz), 22.9, 6.9.
ESI-HR-MS (m/z) (M-H$^+$) calcd. for $C_{11}H_9ClF_3N_2O$, 277.0361; found, 277.0361.

1-(4-chloro-3-(trifluoromethyl)phenyl)-3-(1-methylcyclohexyl)urea (12)

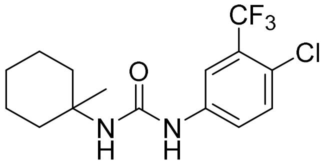

$C_{15}H_{18}ClF_3N_2O$
334.77 g/mol

1-Chloro-4-isocyanato-2-(trifluoromethyl)benzene (489mg, 2.21 mmol, 1.0 eq.) was dissolved in DCM (10 mL). Diisopropylethylamine (422 µl, 313 mg, 2.43 mmol, 1.1 eq.) and 1-methylcyclohexan-1-amine hydrochloride (330 mg, 2.20 mmol, 1.0 eq.) were added and the solution was stirred at room temperature overnight. The volatile components were removed under reduced pressure, and the residue was purified by flash chromatography (hexane/ethyl acetate = 85/15). The product was dissolved in DMF (50 mL) and precipitated by adding water. Filtration yielded the pure product (431 mg, 1.29 mmol, 58%) as a white powder.

TLC (hexane/ethyl acetate = 85/15): R_f = 0.31 [UV] **^{1}H-NMR** (400 MHz, DMSO-d_6): [ppm] = 8.80 (s, 1H), 8.06 (d, *J* = 2.5 Hz, 1H), 7.51 (d, *J* = 8.7 Hz, 1H), 7.44 (dd, *J* = 8.8, 2.5 Hz, 1H), 5.94 (s, 1H), 1.99-1.88 (m, 2H), 1.49-1.39 (m, 5H), 1.32-1.18 (m, 6H). **^{13}C-NMR** (101 MHz, DMSO-d_6): [ppm] = 153.8, 140.2, 131.8, 126.6 (q, *J* = 30.4 Hz), 124.2, 121.9, 121.1-120.7 (m), 115.6 (q, *J* = 5.8 Hz), 51.6, 36.4, 26.9, 25.2, 21.5. **ESI-HR-MS** (m/z) (M-H$^+$) calcd. for $C_{15}H_{17}ClF_3N_2O^-$, 333.0987; found, 333.0987.

1-(4-chloro-3-(trifluoromethyl)phenyl)-3-(2-methylcyclohexyl)urea (13)

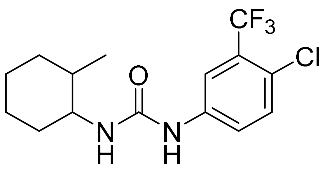

$C_{15}H_{18}ClF_3N_2O$
334.77 g/mol

To DCM (10 mL) 2-methylcyclohexan-1-amine (169 mg, 1.49 mmol, 1.1 eq.) and 1-chloro-4-isocyanato-2-(trifluoromethyl)benzene (300 mg, 1.35 mmol, 1.0 eq.) were added. The reaction was stirred overnight at room temperature. Filtration of the precipitate yielded the product (136 mg, 405 µmol, 30%) as a white powder.

^{1}H-NMR (400 MHz, DMSO-d$_6$): [ppm] = 8.77 (s, 1H), 8.07 (s, 1H), 7.52 (s, 2H), 6.15 (d, *J* = 8.7 Hz, 1H), 3.18-3.08 (m, 1H), 1.86-1.80 (m, 1H), 1.74-1.64 (m, 2H), 1.62-1.56 (m, 1H), 1.34-1.09 (m, 4H), 1.08-0.95 (m, 1H), 0.88 (d, *J* = 6.5 Hz, 3H). **^{13}C-NMR** (101 MHz, DMSO-d$_6$): [ppm] = ^{13}C NMR (101 MHz, DMSO-*d*$_6$) δ 154.9, 140.7, 132.3, 127.1 (q, *J* = 30.4 Hz), 124.7, 122.6, 121.6, 116.5 (d, *J* = 5.7 Hz), 54.5, 38.1, 34.5, 33.9, 25.8, 25.7, 19.6.

The NMR spectra contain the signals of rotamers. Only the chemical shifts of the main rotamer are listed.

ESI-HR-MS (m/z) (M-H$^+$) calcd. for $C_{15}H_{17}ClF_3N_2O^-$, 333.0987; found, 333.0987.

1-(4-chloro-3-(trifluoromethyl)phenyl)-3-(3-methylcyclohexyl)urea (14)

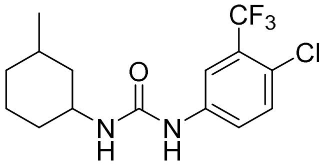

$C_{15}H_{18}ClF_3N_2O$
334.77 g/mol

3-Methylcyclohexan-1-amine hydrochloride (485 mg, 3.23 mmol, 1.1 eq.) was added to 1N NaOH$_{aq}$ (34 mL) and stirred at room temperature for 1h. The solution was extracted with diethylether (3×25 mL). The combined organic phases were washed with brine (1×25 mL). The volatile components were removed under reduced pressure and the residue dissolved in DCM (10 mL). 1-chloro-4-isocyanato-2-(trifluoromethyl)benzene (650 mg, 2.93 mmol, 1.0 eq.) was added, and the solution was stirred at room temperature overnight. Filtration of the precipitate yielded the product (190 mg, 567 µmol, 19%) as a white powder.

^{1}H-NMR (400 MHz, DMSO-d$_6$): [ppm] = 8.87 (s, 1H), 8.07 (s, 1H), 7.51 (s, 2H), 6.59-6.14 (m, 1H), 3.89-3.37 (m, 1H), 1.89-1.79 (m, 2H), 1.72-1.52 (m, 2H), 1.46-1.36 (m, 1H), 1.34-1.19 (m, 1H), 1.08-0.93 (m, 1H), 0.89-0.83 (m, 3H), 0.83-0.68 (m, 2H).

^{13}C-NMR (101 MHz, DMSO-d$_6$): [ppm] = 154.1, 140.2, 131.7, 126.6 (q, *J* = 30.4 Hz), 124.2, 122.1, 121.2, 116.0 (q, *J* = 5.6 Hz), 48.4, 41.8, 33.8, 32.6, 31.4, 24.5, 22.3.
ESI-HR-MS (m/z) (M-H$^+$) calcd. for $C_{15}H_{17}ClF_3N_2O^-$, 333.0987; found, 333.0987.

1-(4-chloro-3-(trifluoromethyl)phenyl)-3-(4-methylcyclohexyl)urea (15)

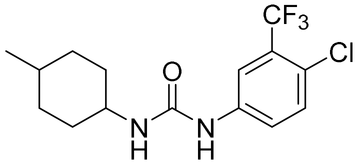

$C_{15}H_{18}ClF_3N_2O$
334.77 g/mol

In DCM (10 mL) 4-methylcyclohexan-1-amine (169 mg, 1.49 mmol, 1.1 eq.) was dissolved, and 1-chloro-4-isocyanato-2-(trifluoromethyl)benzene (300 mg, 1.35 mmol, 1.0 eq.) was added. The reaction was stirred overnight at room temperature. Filtration of the precipitate yielded the product (284 mg, 846 µmol, 63%) as a white powder.

^{1}H-NMR (400 MHz, DMSO-d$_6$): [ppm] = 8.93-8.60 (m, 1H), 8.25-7.99 (m, 1H), 7.64-7.33 (m, 2H), 6.47-6.04 (m, 1H), 3.81-3.33 (m, 1H), 1.88-1.80 (m, 1H), 1.70-1.55 (m, 2H), 1.55-1.46 (m, 2H), 1.45-1.24 (m, 1H), 1.21-1.09 (m, 4H), 1.04-0.93 (m, 1H), 0.92-0.84 (m, 3H).
^{13}C-NMR (101 MHz, DMSO-d$_6$): [ppm] = 154.1, 154.1, 140.1, 140.1, 131.8, 131.77, 127.6-125.7 (m), 124.2, 122.2, 122.0, 121.3-121.1 (m), 116.4-115.4 (m), 48.3, 44.5, 33.6, 32.8, 31.4, 30.5, 29.4, 29.4, 22.1, 21.6.
The observed complexity of the spectra is due to diastereomers.
ESI-HR-MS (m/z) (M-H$^+$) calcd. for $C_{15}H_{17}ClF_3N_2O^-$, 333.0987; found, 333.0987.

1-(4-chloro-3-(trifluoromethyl)phenyl)-3-(1-(hydroxymethyl)cyclohexyl)urea (16)

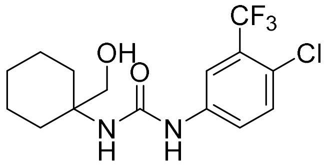

$C_{15}H_{18}ClF_3N_2O_2$
350.77 g/mol

1-(1-Aminocyclohexyl)methanol hydrochloride (206 mg, 1.24 mmol, 1.1 eq.), Na_2CO_3 (125 mg, 1.18 mmol, 1.0 eq.) and 1-chloro-4-isocyanato-2-(trifluoromethyl)benzene (250 mg, 1.18 mmol, 1.0 eq.) were added to DCM (10 mL) and the solution was stirred overnight at room temperature. Water (10 mL) was added, and the precipitate was filtered off. Purification by flash column chromatography (DCM/MeOH = 98/2) yielded the desired product (41.6 mg, 119 µmol, 11%) as a white solid.

TLC (DCM/MeOH = 95/5): R_f = 0.15 [UV]

^{1}H-NMR (400 MHz, DMSO-d$_6$): δ [ppm] = 8.92 (s, 1H), 8.07 (d, *J* = 2.5 Hz, 1H), 7.61-7.36 (m, 2H), 5.83 (s, 1H), 4.71 (t, *J* = 5.7 Hz, 1H), 3.49 (d, *J* = 5.7 Hz, 2H), 1.97 (d, *J* = 13.6 Hz, 2H), 1.56-1.28 (m, 7H), 1.27-1.15 (m, 1H).
^{13}C-NMR (101 MHz, DMSO-d$_6$): δ [ppm] = 154.6, 140.7, 132.3, 127.1 (q, *J* = 30.4 Hz), 124.7, 122.4, 122.0, 121.5 (d, *J* = 1.8 Hz), 116.2 (q, *J* = 5.6 Hz), 66.4, 56.4, 31.6, 25.9, 21.5.
ESI-HR-MS (m/z) (M-H$^+$) calcd. for $C_{15}H_{17}ClF_3N_2O_2^-$, 349.0936; found, 349.0936.

1-(4-chloro-3-(trifluoromethyl)phenyl)-3-(1-(hydroxymethyl)cyclopentyl)urea (17)

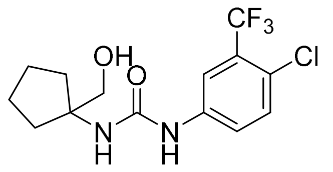

$C_{14}H_{16}ClF_3N_2O_2$
336.74 g/mol

(1-Aminocyclopentyl)methanol (172 mg, 1.49 mmol, 1.1 eq.) was dissolved in DCM (10 mL) and 1-chloro-4-isocyanato-2-(trifluoromethyl)benzene (300 mg, 1.35 mmol, 1.0 eq.) was added. The reaction was stirred at room temperature overnight. The precipitate was filtered off to give the desired product (350 mg, 1.04 mmol, 77%) as a white solid.

^{1}H-NMR (400 MHz, DMSO-d$_6$): δ [ppm] = 8.91 (s, 1H), 8.07 (d, *J* = 2.5 Hz, 1H), 7.56-7.42 (m, 2H), 6.13 (s, 1H), 4.92 (t, *J* = 5.3 Hz, 1H), 3.46 (d, *J* = 5.3 Hz, 2H), 1.86-1.77 (m, 2H), 1.74-1.60 (m, 4H), 1.59-1.45 (m, 2H).
^{13}C-NMR (101 MHz, DMSO-d$_6$): δ [ppm] = 154.8, 140.7, 132.3, 127.1 (q, *J* = 30.6 Hz), 124.7, 122.5, 122.0, 121.5, 116.2 (q, *J* = 5.6 Hz), 65.4, 64.8, 34.7, 24.5.
ESI-HR-MS (m/z) (M-H$^+$) calcd. for $C_{14}H_{16}ClF_3N_2O_2^-$, 335.0780; found, 335.0780.

1-(4-chloro-3-(trifluoromethyl)phenyl)-3-(3-hydroxyadamantan-1-yl)urea (18)

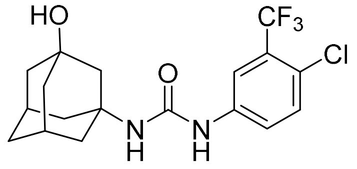

$C_{18}H_{20}ClF_3N_2O_2$
388.81 g/mol

To a solution of 3-aminoadamantan-1-ol (250 mg, 1.49 mmol, 1.0 eq.) in dry DCM (10 mL) 1-chloro-4-isocyanato-2-(trifluoromethyl)benzene (331 mg, 1.49 mmol, 1.0 eq.) was added. The solution was stirred overnight at room temperature. The product was precipitated by the addition of water (25 mL) and filtered off. Residual water was removed by co-evaporation with ethanol to yield the desired product (458 mg, 1.17 mmol, 79%) as a white solid.

^{1}H-NMR (400 MHz, DMSO-d$_6$): δ [ppm] = 8.72 (s, 1H), 8.06 (d, *J* = 2.6 Hz, 1H), 7.65-7.31 (m, 2H), 6.09 (s, 1H), 4.51 (s, 1H), 2.18-2.08 (m, 2H), 1.89-1.70 (m, 6H), 1.60-1.40 (m, 6H).
^{13}C-NMR (101 MHz, DMSO-d$_6$): δ [ppm] = 153.6, 140.1, 131.8, 127.5-125.4 (m), 124.2, 122.1, 121.1, 115.8 (q, *J* = 5.8 Hz), 67.3, 52.7, 49.5, 44.2, 34.8, 30.1.

ESI-HR-MS (m/z) (M-H$^+$) calcd. for $C_{18}H_{19}ClF_3N_2O_2^-$, 387.1093; found, 387.1094.

1-(bicyclo[1.1.1]pentan-1-yl)-3-(4-chloro-3-(trifluoromethyl)phenyl)urea (19)

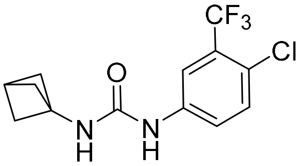

$C_{13}H_{12}ClF_3N_2O$
304.70 g/mol

Bicyclo[1.1.1]pentan-2-amine hydrochloride (250 mg, 2.13 mmol, 1.1 eq.) and Na_2CO_3 (206 mg, 1.94 mmol, 1.0 eq.) and 1-chloro-4-isocyanato-2-(trifluoromethyl)benzene (430 mg, 1.94 mmol, 1.0 eq.) were added to DCM (10 mL). The reaction was stirred at room temperature overnight, and water (10 mL) was added. The precipitate was filtered off and purified by flash column chromatography (DCM/MeOH = 99/1) to yield the desired product (74,5 mg, 245 µmol, 13%) as a white solid.

TLC (DCM): R_f = 0.16 [UV]
^{1}H-NMR (400 MHz, DMSO-d$_6$): δ [ppm] = 8.76 (s, 1H), 8.06 (s, 1H), 7.53 (d, *J* = 1.4 Hz, 2H), 6.98 (s, 1H), 2.40 (s, 1H), 1.99 (s, 6H).
^{13}C-NMR (101 MHz, DMSO-d$_6$): δ [ppm] = 154.6, 140.3, 1323, 127.0 (q, *J* = 30.4 Hz), 124.7, 123.0, 122.0 (d, *J* = 1.8 Hz), 122.0, 116.9-116.3 (m), 52.7, 49.1, 24.6.
ESI-HR-MS (m/z) (M-H$^+$) calcd. for $C_{13}H_{11}ClF_3N_2O^-$, 303.0517; found, 303.0517.

1-(4-chloro-3-(trifluoromethyl)phenyl)-3-(2,2-dimethyl-1,3-dioxan-5-yl)urea (20)

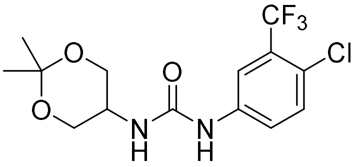

$C_{14}H_{16}ClF_3N_2O_3$
352.73 g/mol

1-Chloro-4-isocyanato-2-(trifluoromethyl)benzene (338 mg, 1.52 mmol, 1.0 eq.) was added to a solution of 2,2-dimethyl-1,3-dioxan-5-amine (200 mg, 1.52 mmol, 1.0 eq.) in dry DCM (10 mL). The reaction was stirred at room temperature overnight. Water (25 mL) was added, the phases were separated, and the organic phase was washed with water (25 mL). The volatile components of the organic phase were removed under reduced pressure, the residue was dissolved in dimethylformamide (5 mL) and precipitated with water (25 mL). The precipitate was filtered off, and residual water was removed by co-evaporation with methanol (MeOH). Purification with flash chromatography (DCM/MeOH = 97/3) yielded the product (366 mg, 1.03 mmol, 68%) as a white solid.

TLC (DCM/MeOH = 97/3): R_f = 0.30 [UV]

^{1}H-NMR (400 MHz, DMSO-d_6): δ [ppm] = 9.14 (s, 1H), 8.06 (d, *J* = 2.2 Hz, 1H), 7.75-7.34 (m, 2H), 6.67 (d, *J* = 7.8 Hz, 1H), 4.09-3.90 (m, 2H), 3.67-3.49 (m, 3H), 1.37 (d, *J* = 14.3 Hz, 6H).
^{13}C-NMR (101 MHz, DMSO-d_6): δ [ppm] = 154.3, 139.9, 131.9, 126.7 (q, *J* = 30.5 Hz), 124.2, 122.2, 122.0-120.3 (m), 116.0 (q, *J* = 5.7 Hz), 97.7, 62.9, 43.4, 26.6, 20.8.
ESI-HR-MS (m/z) (M-H$^+$) calcd. for $C_{14}H_{15}ClF_3N_2O_3^-$, 351.0729; found, 351.0728.

1-(4-chloro-3-(trifluoromethyl)phenyl)-3-(5,9-dioxaspiro[3.5]nonan-7-yl)urea (21)

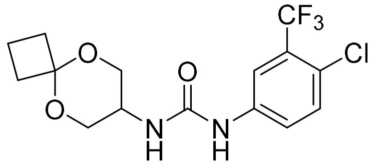

$C_{15}H_{16}ClF_3N_2O_3$
364.74 g/mol

5,9-Dioxaspiro[3.5]nonan-7-amine (200 mg, 1.40 mmol, 1.0 eq.) was dissolved in dry DCM (10 mL), and 1-chloro-4-isocyanato-2-(trifluoromethyl)benzene (309 mg, 1.40 mmol, 1.0 eq.) was added. The solution was stirred overnight at room temperature. Water (25 mL) was added, and the phases separated. The organic phase was washed with water (25 mL), and the volatile components were removed under reduced pressure. The residue was dissolved in dimethylformamide (5 mL) and precipitated with water (25 mL). The precipitate was filtered of and residual water was removed by co-evaporation with methanol (MeOH). Flash chromatography (DCM/MeOH = 98/2) yielded the product (261 mg, 718 µmol, 51%) as a white solid.

TLC (DCM/MeOH = 97/3): R_f = 0.30 [UV]
^{1}H-NMR (400 MHz, DMSO-d_6): δ [ppm] = 9.12 (s, 1H), 8.06 (s, 1H), 7.61-7.45 (m, 2H), 6.70 (d, *J* = 7.9 Hz, 1H), 3.96-3.86 (m, 2H), 3.65-3.51 (m, 3H), 2.25-2.09 (m, 4H), 1.72-1.57 (m, 2H).
^{13}C-NMR (101 MHz, DMSO-d_6): δ [ppm] = 154.4, 139.9, 131.9, 126.7 (q, *J* = 30.4 Hz), 124.2, 122.3, 122.0-121.3 (m), 116.0 (q, *J* = 5.8 Hz), 101.0, 63.8, 43.4, 33.3, 30.5, 11.1.
ESI-HR-MS (m/z) (M-H$^+$) calcd. for $C_{15}H_{15}ClF_3N_2O_3^-$, 363.0729; found, 363.0728.

1-(4-chloro-3-(trifluoromethyl)phenyl)-3-(1,4-dioxaspiro[4.5]decan-8-yl)urea (22)

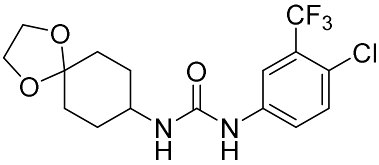

$C_{16}H_{18}ClF_3N_2O_3$
378.78 g/mol

1-Chloro-4-isocyanato-2-(trifluoromethyl)benzene (352 mg, 1.59 mmol, 1.0 eq.) was dissolved in dry DCM (10 mL) and 1,4-dioxaspiro[4.5]decan-8-amine (250 mg, 1.59 mmol, 1.0 eq.) was added. The solution was stirred at room temperature overnight. The precipitate

was filtered of and washed with DCM to yield the product (521 mg, 1.37 mmol, 87%) as a slightly rose powder.

^{1}H-NMR (400 MHz, DMSO-d$_6$): δ [ppm] = 8.75 (s, 1H), 8.06 (d, *J* = 1.7 Hz, 1H), 7.68-7.32 (m, 2H), 6.35 (d, *J* = 7.7 Hz, 1H), 3.85 (s, 4H), 3.65-3.49 (m, 1H), 1.82-1.74 (m, 2H), 1.70-1.61 (m, 2H), 1.59-1.51 (m, 2H), 1.50-1.39 (m, 2H).
^{13}C-NMR (101 MHz, DMSO-d$_6$): δ [ppm] = 154.2, 140.1, 131.8, 126.6 (q, *J* = 30.5 Hz), 124.2, 122.2, 121.5-121.1 (m), 116.0 (q, *J* = 5.6 Hz), 107.3, 63.7, 46.4, 32.3, 29.5.
ESI-HR-MS (m/z) (M-H$^+$) calcd. for $C_{16}H_{17}ClF_3N_2O_3^-$, 377.0885; found, 377.0885.

7-methyl-7-nitro-5,9-dioxaspiro[3.5]nonane (23-2)

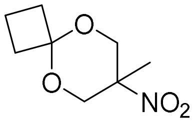

$C_8H_{13}NO_4$
187.20 g/mol

2-Methyl-2-nitro-1,3-propanediol (250 mg, 1.85 mmol, 1.0 eq.) was dissolved in acetonitrile (4 mL). Cyclobutanone (136 µL, 130 mg, 1.85 mmol, 1.0 eq.), triethyl orthoformate (306 µL, 302 mg, 2.04 mmol, 1.1 eq.) and a catalytic amount of p-toluenesulfonic acid were added. The reaction was stirred overnight at room temperature. The volatile components were removed under reduced pressure. The residue was dissolved in water (10 mL) and extracted with DCM (3×10 mL). The pooled organic phases were washed with brine (1×10 mL). The volatile components were removed under reduced pressure to yield the desired product (330 mg, 1.76 mmol, 95%) as an off-white solid.

^{1}H-NMR (500 MHz, DMSO-d$_6$): δ [ppm] = 4.51 (d, *J* = 12.5 Hz, 2H), 3.78 (d, *J* = 12.5 Hz, 2H), 2.25 (q, *J* = 8.2 Hz, 4H), 1.76 (p, *J* = 8.0 Hz, 2H), 1.44 (s, 3H).
^{13}C-NMR (101 MHz, DMSO-d$_6$): δ [ppm] = 101.7, 82.3, 65.7, 33.4, 30.2, 19.8, 11.4.

7-methyl-5,9-dioxaspiro[3.5]nonan-7-amine (23-1)

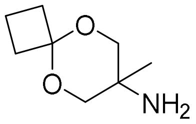

$C_8H_{15}NO_2$
157.21 g/mol

Compound **23-2** (330 mg, 1.76 mmol) was dissolved in MeOH (15 mL) and Palladium (10%) on carbon (20 mg) was added. The reaction was stirred overnight at room temperature under a hydrogen atmosphere. The mixture was filtered over Celite® and the volatile components were removed under reduced pressure to yield the desired product (120 mg) as a yellow oil. The product was used without further purification.

^{1}H-NMR (400 MHz, DMSO-d_6): ⍰ [ppm] = 3.54 (d, *J* = 11.3 Hz, 2H), 3.40 (d, *J* = 11.3 Hz, 2H), 2.21-2.14 (m, 4H), 1.73-1.63 (m, 2H), 0.96 (s, 3H).
^{13}C-NMR (101 MHz, DMSO-d_6): ⍰ [ppm] = 101.4, 71.2, 47.1, 33.8, 30.3, 21.1, 11.5.
ESI-HR-MS (m/z) (M+H$^+$) calcd. for $C_8H_{16}NO_2^+$, 158.1176; found, 158.1176.

1-(4-chloro-3-(trifluoromethyl)phenyl)-3-(7-methyl-5,9-dioxaspiro[3.5]nonan-7-yl)urea (23)

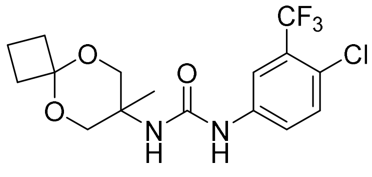

$C_{16}H_{18}ClF_3N_2O_3$
378.78 g/mol

Compound **23-1** (120 mg, 1.1 eq.) was dissolved in DCM (5 mL) and 1-chloro-4-isocyanato-2-(trifluoromethyl)benzene (154 mg, 694 µmol, 1.0 eq.) was added. The reaction was stirred overnight at room temperature and the volatile organic compounds were removed under reduced pressure. The residue was purified by flash column chromatography (DCM/MeOH = 1/0→98/2) to yield the desired product (160 mg, 422 µmol, 61%) as a white solid.

TLC (DCM): R_f = 0.06 [UV]
^{1}H-NMR (400 MHz, DMSO-d_6): ⍰ [ppm] = 9.09 (s, 1H), 8.06 (d, *J* = 2.5 Hz, 1H), 7.52 (d, *J* = 8.8 Hz, 1H), 7.46 (dd, *J* = 8.8, 2.5 Hz, 1H), 6.35 (s, 1H), 3.86 (d, *J* = 11.6 Hz, 2H), 3.53 (d, *J* = 11.6 Hz, 2H), 2.21 (t, *J* = 8.0 Hz, 2H), 2.17-2.11 (m, 2H), 1.65 (p, *J* = 8.0 Hz, 2H), 1.18 (s, 3H).
^{13}C-NMR (101 MHz, DMSO-d_6): ⍰ [ppm] = 154.9, 140.4, 132.3, 127.1 (q, *J* = 30.4 Hz), 124.7, 122.6, 122.0, 121.9 (d, *J* = 1.8 Hz), 116.3 (q, *J* = 5.8 Hz), 101.2, 67.3, 49.4, 33.9, 30.7, 19.1, 11.5.
ESI-HR-MS (m/z) (M-H$^+$) calcd. for $C_{16}H_{17}ClF_3N_2O_3^-$, 377.0885; found, 377.0887.

1-(4-chloro-3-(trifluoromethyl)phenyl)-3-(piperidin-1-yl)urea (24)

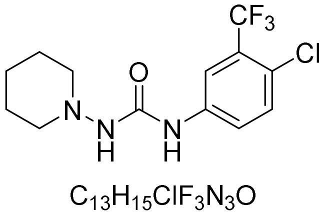

$C_{13}H_{15}ClF_3N_3O$
321.72 g/mol

1-Chloro-4-isocyanato-2-(trifluoromethyl)benzene (350 mg, 1.58 mmol, 1.1 eq.) was added to a solution of piperidin-1-amine (144 mg, 1.44 mmol, 1.0 eq.) in dry DCM (10 mL). The solution was stirred at room temperature overnight. The volatile components were removed under reduced pressure and the residue was purified by flash chromatography (hexane/ethyl acetate = 9/1→8/2) to yield the product (221 mg, 689 µmol, 48%) as a white solid.

TLC (hexane/ethyl acetate = 8/2): R_f = 0.31 [UV]

^{1}H-NMR (400 MHz, DMSO-d_6): δ [ppm] = 8.93 (s, 1H), 8.20 (d, *J* = 2.6 Hz, 1H), 7.95 (dd, *J* = 8.9, 2.6 Hz, 1H), 7.92 (s, 1H), 7.55 (d, *J* = 8.8 Hz, 1H), 1.94-0.87 (m, 4H).
The missing CH_2-groups are very broad and thus can't be integrated.
^{13}C-NMR (101 MHz, DMSO-d_6): δ [ppm] =154.6, 139.4, 131.5, 126.4 (q, *J* = 30.5 Hz), 124.3, 123.6, 122.1 (d, *J* = 2.1 Hz), 117.7 (q, *J* = 5.7 Hz), 56.6, 25.1, 22.8.
ESI-HR-MS (m/z) (M-H$^+$) calcd. for $C_{13}H_{14}ClF_3N_3O^-$, 320.0783; found, 320.0782.

1-(4-chloro-3-(trifluoromethyl)phenyl)-3-morpholinourea (25)

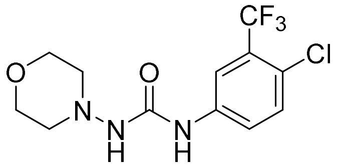

$C_{12}H_{13}ClF_3N_3O_2$
323.70 g/mol

To a solution of morpholine-4-amine (200 mg, 1.96 mmol, 1.0 eq.) in dry DCM (10 mL) 1-chloro-4-isocyanato-2-(trifluoromethyl)benzene (434 mg, 1.96 mmol, 1.0 eq.) was added, and the solution was stirred at room temperature overnight. Water (25 mL) was added, and the phases were separated. The organic phase was washed with brine (1×25 mL) and the volatile components were removed under reduced pressure. Flash chromatography (DCM/MeOH = 97/3) yielded the product (144 mg, 445 µmol, 23%) as a white solid.

TLC (DCM/MeOH = 97/3): R_f = 0.27 [UV]
^{1}H-NMR (400 MHz, DMSO-d_6): δ [ppm] = 9.00 (s, 1H), 8.18 (d, *J* = 2.6 Hz, 1H), 8.03 (s, 1H), 7.95 (dd, *J* = 8.9, 2.6 Hz, 1H), 7.56 (d, *J* = 8.8 Hz, 1H), 3.71 (s, 4H), 2.74 (s, 4H).
^{13}C-NMR (101 MHz, DMSO-d_6): δ [ppm] = 154.6, 139.3, 131.5, 126.4 (q, *J* = 30.5 Hz), 124.3, 123.7, 122.7-122.0 (m), 117.8 (q, *J* = 5.6 Hz), 65.8, 55.7.
ESI-HR-MS (m/z) (M-H$^+$) calcd. for $C_{12}H_{12}ClF_3N_3O_2^-$, 322.0576; found, 322.0575.

tert-butyl 4-(3-(4-chloro-3-(trifluoromethyl)phenyl)ureido)piperidine-1-carboxylate (26)

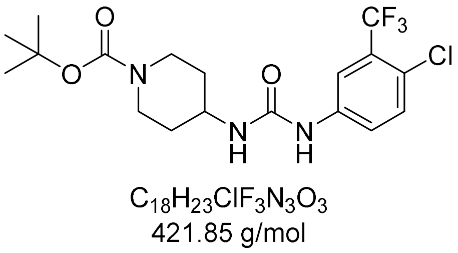

$C_{18}H_{23}ClF_3N_3O_3$
421.85 g/mol

1-Chloro-4-isocyanato-2-(trifluoromethyl)benzene (553 mg, 2.50 mmol, 1.0 eq.) was dissolved in dry DCM (20 mL) and 1-*Boc*-4-aminopiperidine (500 mg, 2.50 mmol, 1.0 eq.) was added. The reaction was stirred over 3 days at room temperature and the volatile components were removed under reduced pressure. The product was purified by flash chromatography (hexane/ethyl acetate = 2/1) to yield the product (419 mg, 997 µmol, 40%) as a white solid.

TLC (hexane/ethyl acetate = 2/1): R_f = 0.13 [UV]

^{1}H-NMR (400 MHz, DMSO-d_6): δ [ppm] = 8.84 (s, 1H), 8.11-8.00 (m, 1H), 7.58-7.49 (m, 2H), 6.36 (d, *J* = 7.7 Hz, 1H), 3.83 (d, *J* = 13.2 Hz, 2H), 3.72-3.55 (m, 1H), 2.88 (s, 2H), 1.86-1.71 (m, 2H), 1.40 (s, 9H), 1.33-1.21 (m, 2H).
^{13}C-NMR (101 MHz, DMSO-d_6): δ [ppm] = 154.1, 153.9, 140.0, 131.8, 126.6 (q, *J* = 30.6 Hz), 124.2, 122.3, 121.6-121.1 (m), 116.7-115.6 (m), 78.6, 46.3, 31.7, 28.1.
ESI-HR-MS (m/z) (M-H$^+$) calcd. for $C_{18}H_{22}ClF_3N_3O_3^-$, 420.1307; found, 420.1307.

1-(4-chloro-3-(trifluoromethyl)phenyl)-3-(oxetan-3-yl)urea (27)

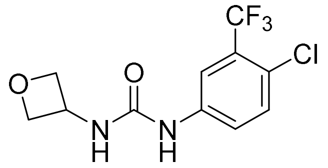

$C_{11}H_{10}ClF_3N_2O_2$
294.66 g/mol

Oxetan-3-amine (150 mg, 2.05 mmol, 1.0 eq.) was dissolved in dry DCM (10 mL) and 1-chloro-4-isocyanato-2-(trifluoromethyl)benzene (455 mg, 2.05 mmol, 1.0 eq.) was added. The reaction was stirred overnight at room temperature and water (25 mL) was added. The phases where separated and the organic phase was washed with water (50 mL). Under reduced pressure the volatile components were removed under reduced pressure and the residue was purified by flash chromatography (DCM/MeOH = 97/3) to yield the product (442 mg, 1.50 mmol, 73%) as a white solid.

TLC (DCM/MeOH = 97/3): R_f = 0.18 [UV]
^{1}H-NMR (400 MHz, DMSO-d_6): δ [ppm] = 9.05 (s, 1H), 8.04 (d, *J* = 2.4 Hz, 1H), 7.67-7.46 (m, 2H), 7.12 (d, *J* = 6.4 Hz, 1H), 4.84-4.59 (m, 3H), 4.45 (t, *J* = 6.0 Hz, 2H).
^{13}C-NMR (101 MHz, DMSO-d_6): δ [ppm] = 154.2, 139.8, 131.8, 126.6 (q, *J* = 30.5 Hz), 124.2, 122.6, 121.7, 116.4 (q, *J* = 5.6 Hz), 77.5, 44.6.
ESI-HR-MS (m/z) (M-H$^+$) calcd. for $C_{11}H_9ClF_3N_2O_2^-$, 293.0310; found, 293.0310.

1-(4-chloro-3-(trifluoromethyl)phenyl)-3-(tetrahydrofuran-3-yl)urea (28)

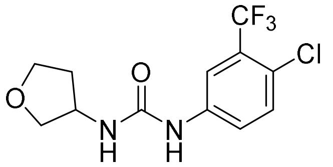

$C_{12}H_{12}ClF_3N_2O_2$
308.69 g/mol

To a solution of tetrahydrofuran-3-amine (250 mg, 2.87 mmol, 1.0 eq.) in dry DCM (10 mL) 1-chloro-4-isocyanato-2-(trifluoromethyl)benzene (636 mg, 2.87 mmol, 1.0 eq.) was added. The reaction was stirred at room temperature overnight. Water (25 mL) was added and the phases were separated. The organic phase was washed with water (1×25 mL) and brine (1×25 mL) and the volatile components were removed under reduced pressure. The crude

product was purified by flash chromatography (DCM/MeOH = 95/5) to yield the product (280 mg, 907 µmol, 32%) as a white solid.

TLC (DCM/MeOH = 95/5): R_f = 0.30 [UV]
^{1}H-NMR (400 MHz, DMSO-d_6): □ [ppm] = 8.82 (s, 1H), 8.06 (s, 1H), 7.53 (s, 2H), 6.61 (d, *J* = 6.3 Hz, 1H), 4.20 (s, 1H), 3.85-3.63 (m, 3H), 3.55- 3.45 (m, 1H), 2.20- 2.00 (m, 1H), 1.83-1.64 (m, 1H).
^{13}C-NMR (101 MHz, DMSO-d_6): □ [ppm] = 154.6, 139.9, 131.8, 126.6 (q, *J* = 30.4 Hz), 124.2, 122.4, 121.6-121.0 (m), 116.2 (q, *J* = 5.8 Hz), 72.8, 66.2, 50.2, 32.5.
ESI-HR-MS (m/z) (M-H$^+$) calcd. for $C_{12}H_{11}ClF_3N_2O_2^-$, 307.0467; found, 307.0466.

1-(4-chloro-3-(trifluoromethyl)phenyl)-3-(3-methyloxetan-3-yl)urea (29)

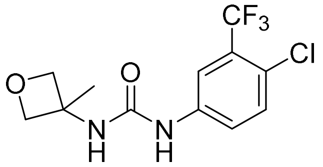

$C_{12}H_{12}ClF_3N_2O_2$
308.69 g/mol

To a solution of 3-methyloxetan-3-amine (100 mg, 1.15 mmol, 1.0 eq.) in dry DCM (4 mL) 1-chloro-4-isocyanato-2-(trifluoromethyl)benzene (254 mg, 1.15 mmol, 1.0 eq.) was added and the reaction was stirred overnight at room temperature. The precipitate was filtered of to yield the product (304 mg, 988 µmol, 86%) as a white solid.

^{1}H-NMR (400 MHz, DMSO-d_6): □ [ppm] = 9.02 (s, 1H), 8.06 (d, *J* = 2.2 Hz, 1H), 7.73-7.26 (m, 2H), 6.84 (s, 1H), 4.61 (d, *J* = 6.1 Hz, 2H), 4.30 (d, *J* = 6.1 Hz, 2H), 1.55 (s, 3H).
^{13}C-NMR (101 MHz, DMSO-d_6): □ [ppm] = 153.9, 139.9, 131.8, 126.61 (q, *J* = 30.5 Hz), 124.2, 122.5, 121.8-121.3 (m), 116.3 (q, *J* = 5.7 Hz), 81.1, 52.7, 23.8.
ESI-HR-MS (m/z) (M-H$^+$) calcd. for $C_{12}H_{11}ClF_3N_2O_2^-$, 307.0467; found, 307.0466.

1-(4-chloro-3-(trifluoromethyl)phenyl)-3-(tetrahydro-2H-pyran-4-yl)urea (30)

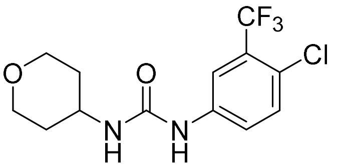

$C_{13}H_{14}ClF_3N_2O_2$
322.71 g/mol

Tetrahydro-2H-pyran-4-amine (200 mg, 1.98 mmol, 1.0 eq.) was dissolved in dry DCM (20 mL) and 1-chloro-4-isocyanato-2-(trifluoromethyl)benzene (438 mg, 1.98 mmol, 1.0 eq.) was added. The reaction was stirred at room temperature overnight and water (25 mL) was added. The product was filtered off and washed with water and DCM. The residual water was removed by co-evaporation with Methanol and purification by flash chromatography (DCM/MeOH = 95/5) yielded the product (209 mg, 649 µmol, 33%) as a white solid.

TLC (DCM/MeOH = 95/5): R_f = 0.30 [UV]
^{1}H-NMR (400 MHz, DMSO-d_6): [ppm] = 8.82 (s, 1H), 8.06 (s, 1H), 7.53 (s, 2H), 6.37 (d, *J* = 7.6 Hz, 1H), 3.81 (d, *J* = 9.6 Hz, 2H), 3.68 (br s, 1H), 3.38 (d, *J* = 11.6 Hz, 2H, overlapping with the water signal), 1.77 (d, *J* = 10.9 Hz, 2H), 1.64-1.15 (m, 2H).
^{13}C-NMR (101 MHz, DMSO-d_6): [ppm] =154.1, 140.0, 131.8, 126.6 (q, *J* = 30.7 Hz), 124.2, 122.3, 121.4, 116.1 (q, *J* = 5.6 Hz), 65.8, 45.5, 32.9.
ESI-HR-MS (m/z) (M-H$^+$) calcd. for $C_{13}H_{13}ClF_3N_2O_2^-$, 321.0623; found, 321.0623.

1-(4-chloro-3-(trifluoromethyl)phenyl)-3-(1-methylpiperidin-4-yl)urea (31)

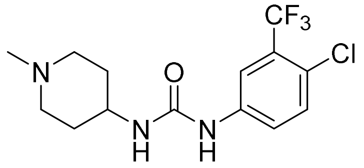

$C_{14}H_{17}ClF_3N_3O$
335.76 g/mol

1-Methylpiperidin-4-amine (227 mg, 1.99 mmol, 1.1 eq.) was dissolved in dry DCM (10 mL) and 1-chloro-4-isocyanato-2-(trifluoromethyl)benzene (400 mg, 1.81 mmol, 1.0 eq.) was added. The reaction was stirred for 3 days at room temperature. The product was filtered, rinsed with DCM and dried under reduced pressure. This yielded the product (396 mg, 1.18 mol, 65%) as a white powder.

^{1}H-NMR (400 MHz, DMSO-d_6): [ppm] = 8.79 (s, 1H), 8.06 (d, *J* = 2.1 Hz, 1H), 7.56-7.46 (m, 2H), 6.29 (d, *J* = 7.7 Hz, 1H), 3.48-3.37 (m, 1H), 2.68- 2.57 (m, 2H), 2.14 (s, 3H), 1.98 (t, *J* = 10.5 Hz, 2H), 1.82-1.71 (m, 2H), 1.48-1.31 (m, 2H).
^{13}C-NMR (101 MHz, DMSO-d_6): [ppm] = 154.2, 140.1, 131.8, 126.6 (q, *J* = 30.6 Hz), 124.2, 122.2, 121.3, 116.0 (q, *J* = 5.7 Hz), 53.9, 46.0, 31.9.
ESI-HR-MS (m/z) (M-H$^+$) calcd. for $C_{14}H_{16}ClF_3N_3O^-$, 334.0939; found, 334.0941.

1-(4-chloro-3-(trifluoromethyl)phenyl)-3-(1-ethynylcyclohexyl)urea (32)

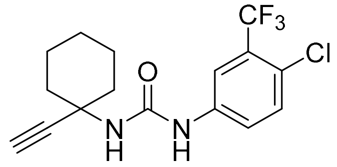

$C_{16}H_{16}ClF_3N_2O$
344.76 g/mol

1-Ethynylcyclohexylamine (153 mg, 1.24 mmol, 1.1 eq.) was dissolved in dry DCM (10 mL) and 1-chloro-4-isocyanato-2-(trifluoromethyl)benzene (250 mg, 1.13 mmol, 1.0 eq.) was added. The reaction was stirred overnight at room temperature and the volatile components were removed under reduced pressure. The residue was purified by flash column chromatography (hexane/ethyl acetate = 8/2) to yield the desired product (252 mg, 731 µmol, 65%) as a white solid.

TLC (hexane/ethyl acetate = 8/2): R_f = 0.22 [UV]
^{1}H-NMR (400 MHz, DMSO-d_6): [ppm] = 8.81 (s, 1H), 8.08 (d, *J* = 2.5 Hz, 1H), 7.55 (d, *J* = 8.8 Hz, 1H), 7.47 (dd, *J* = 8.8, 2.5 Hz, 1H), 6.48 (s, 1H), 3.19 (s, 1H), 2.04-1.94 (m, 2H), 1.78-1.69 (m, 2H), 1.58-1.44 (m, 6H).
^{13}C-NMR (101 MHz, DMSO-d_6): [ppm] = 153.9, 140.3, 132.4, 127.1 (q, *J* = 30.5 Hz), 124.7, 122.8, 122.0 (d, *J* = 1.9 Hz), 116.4 (q, *J* = 5.8 Hz), 87.2, 73.5, 50.7, 37.2, 25.2, 22.3.
ESI-HR-MS (m/z) (M-H$^+$) calcd. for $C_{16}H_{15}ClF_3N_2O^-$, 343.0830; found, 343.0831.

1-(4-chloro-3-(trifluoromethyl)phenyl)-3-(1,1-dioxidotetrahydrothiophen-3-yl)urea (33)

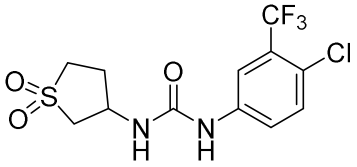

$C_{12}H_{12}ClF_3N_2O_3S$
356.74 g/mol

1-Chloro-4-isocyanato-2-(trifluoromethyl)benzene (410 mg, 1.85 mmol, 1.0 eq.) was dissolved in dry DCM (10 mL) and 3-aminotetrahydrothiophene-1,1-dioxide hydrochloride (252 mg, 1.85 mmol, 1.0 eq.) and *N*, *N*-diisopropylethylamine (354 µL, 263 mg, 2.04 mmol, 1.1 eq.) were added. The reaction was stirred at room temperature overnight, and the volatile components were removed under reduced pressure. Flash column chromatography (DCM/MeOH = 95/5) yielded the product (258 mg, 723 µmol, 39%) as a white solid.

TLC (DCM/Methanol= 95/5): R_f = 0.29 [UV]
^{1}H-NMR (400 MHz, DMSO-d_6): [ppm] = 9.10 (s, 1H), 8.05 (d, *J* = 2.4 Hz, 1H), 7.64-7.50 (m, 2H), 6.81 (d, *J* = 7.4 Hz, 1H), 4.54-4.37 (m, 1H), 3.49-3.38 (m, 1H), 3.31-3.22 (m, 1H), 3.22-3.11 (m, 1H), 3.07-2.94 (m, 1H), 2.47-2.34 (m, 1H), 2.18-2.03 (m, 1H).
^{13}C-NMR (101 MHz, DMSO-d_6): [ppm] = 154.3, 139.7, 131.9, 126.7 (q, *J* = 30.5 Hz), 124.2, 122.6, 122.1-121.7 (m), 116.4 (q, *J* = 5.7 Hz), 55.5, 50.5, 46.5, 28.9.
ESI-HR-MS (m/z) (M-H$^+$) calcd. for $C_{12}H_{11}ClF_3N_2O_3S^-$, 355.0136; found, 355.0136.

1-(4-chloro-3-(trifluoromethyl)phenyl)-3-(1,1-dioxidotetrahydro-2H-thiopyran-4-yl)urea (34)

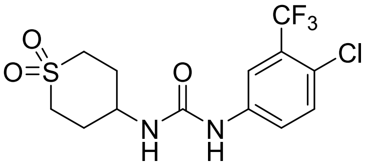

$C_{13}H_{14}ClF_3N_2O_3S$
370.77 g/mol

To a solution of 1-chloro-4-isocyanato-2-(trifluoromethyl)benzene (371 mg, 1.68 mmol, 1.0 eq.) in dry DCM (10 mL) and 4-aminotetrahydro-2H-thiopyran-1,1-dioxide (250 mg, 1.68 mmol, 1.0 eq.) was added. The reaction was stirred at room temperature overnight and the

volatile components were removed under reduced pressure. Flash column chromatography (DCM/MeOH = 95/5) yielded the product (296 mg, 801 µmol, 48%) as a white solid.

TLC (DCM/Methanol= 95/5): R_f = 0.27 [UV]
^{1}H-NMR (400 MHz, DMSO-d_6): [ppm] = 8.83 (s, 1H), 8.05 (d, *J* = 2.1 Hz, 1H), 7.62-7.48 (m, 2H), 6.61 (d, *J* = 7.7 Hz, 1H), 3.93- 3.75 (m, 1H), 3.32-3.22 (m, 2H), 3.11-2.95 (m, 2H), 2.19-2.05 (m, 2H), 2.02-1.86 (m, 2H).
^{13}C-NMR (101 MHz, DMSO-d_6): [ppm] = 154.2, 139.9, 131.8, 126.6 (q, *J* = 30.6 Hz), 124.2, 122.5, 121.8-121.5 (m), 116.3 (q, *J* = 5.8 Hz), 48.6, 44.7, 29.6.
ESI-HR-MS (m/z) (M-H$^+$) calcd. for $C_{13}H_{13}ClF_3N_2O_3S^-$, 369.0293; found, 369.0293.

1-(4-chloro-3-(trifluoromethyl)phenyl)-3-(2-(trifluoromethyl)-1,3-dioxolan-2-yl)urea (35)

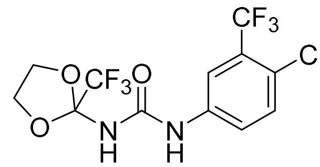

$C_{12}H_9ClF_6N_2O_3$
378.66 g/mol

2-(Trifluoromethyl)-1,3-dioxolan-2-amine (312 mg, 1.99 mmol, 1.1 eq.) was dissolved in dry DCM (10 mL). 1-chloro-4-isocyanato-2-(trifluoromethyl)benzene (400 mg, 1.81 mmol, 1.0 eq.) was added and the solution was stirred at room temperature overnight. Filtration yielded the product (134 mg, 355 µmol, 20%) as slightly white powder.

^{1}H-NMR (400 MHz, DMSO-d_6): [ppm] = 8.98 (s, 1H), 8.02 (d, *J* = 2.5 Hz, 1H), 7.90 (s, 1H), 7.62-7.57 (m, 1H), 7.56-7.50 (m, 1H), 4.48-4.33 (m, 2H), 4.20-4.03 (m, 2H).
^{13}C-NMR (101 MHz, DMSO-d_6): [ppm] = 151.8, 138.7, 132.0, 126.8 (q, *J* = 30.6 Hz), 124.1, 123.1, 123.0-122.8 (m), 121.4, 117.5-116.2 (m), 107.02 (q, *J* = 33.5 Hz), 67.4.
ESI-HR-MS (m/z) (M-H$^+$) calcd. for $C_{12}H_8ClF_6N_2O_3^-$, 377.0133; found, 377.0131.

Methyl 4-(3-(4-chloro-3-(trifluoromethyl)phenyl)ureido)bicyclo[2.2.2]octane-1-carboxylate (36)

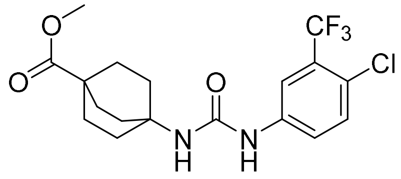

$C_{18}H_{20}ClF_3N_2O_3$
404.81 g/mol

Methyl 4-aminobicyclo[2.2.2]octane-1-carboxylate hydrochloride (300 mg, 1.64 mmol, 1.0 eq.) and DIPEA (314 µL, 233 mg, 1.80 mmol, 1.1 eq.) were dissolved in DCM (10 mL) and 1-chloro-4-isocyanato-2-(trifluoromethyl)benzene (363mg, 1.64 mmol, 1.0 eq.) was added. The reaction was stirred at room temperature overnight. The volatile components were removed under reduced pressure. Flash chromatography (hexane/ethyl acetate =

85/15→75/25) of the residue yielded the product (546 mg, 1.35 mmol, 82%) as a colourless solid.

TLC (hexane/ethyl acetate = 8/2): R_f = 0.13 [UV]
^{1}H-NMR (400 MHz, DMSO-d$_6$): δ [ppm] = 8.72 (s, 1H), 8.05 (d, *J* = 2.5 Hz, 1H), 7.51 (d, *J* = 8.8 Hz, 1H), 7.41 (dd, *J* = 8.8, 2.5 Hz, 1H), 6.04 (s, 1H), 3.56 (s, 3H), 1.98-1.59 (m, 12H).
^{13}C-NMR (101 MHz, DMSO-d$_6$): δ [ppm] = 176.9, 153.9, 140.0, 131.8, 126.60 (q, *J* = 30.3 Hz), 124.2, 122.1, 121.3-121.0 (m), 115.8 (q, *J* = 5.8 Hz), 51.5, 49.5, 37.7, 29.6, 28.2.
ESI-HR-MS (m/z) (M-H$^+$) calcd. for $C_{18}H_{19}ClF_3N_2O_3^-$, 403.1042; found, 403.1041.

1-(4-chloro-3-(trifluoromethyl)phenyl)-3-(1-cyanocyclohexyl)urea (37)

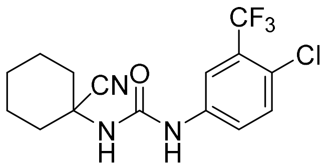

$C_{15}H_{15}ClF_3N_3O$
345.75 g/mol

1-Cyanocyclohexylamine (370 mg, 2.98 mmol, 1.1 eq.) and 1-chloro-4-isocyanato-2-(trifluoromethyl)benzene (600 mg, 2.71 mmol, 1.0 eq.) were added to DCM (20 mL). The reaction was stirred at room temperature overnight. The precipitate was filtered off to yield the desired product (670 mg, 1.94 mmol, 72%) as a white powder.

^{1}H-NMR (400 MHz, DMSO-d$_6$): δ [ppm] = 9.01 (s, 1H), 8.08 (s, 1H), 7.56 (s, 2H), 6.95 (s, 1H), 1.77-1.41 (m, 10H).
^{13}C-NMR (101 MHz, DMSO-d$_6$): δ [ppm] = 154.0, 139.8, 132.4, 127.2 (q, *J* = 30.6 Hz), 124.6, 123.1, 122.9-122.5 (m), 121.9, 121.2, 116.8 (q, *J* = 5.7 Hz), 51.4, 35.2, 24.6, 22.0.
ESI-HR-MS (m/z) (M-H$^+$) calcd. for $C_{15}H_{14}ClF_3N_3O^-$, 344.0783; found, 344.0783.

1-(4-chloro-3-(trifluoromethyl)phenyl)-3-(4-hydroxycyclohexyl)urea (38)

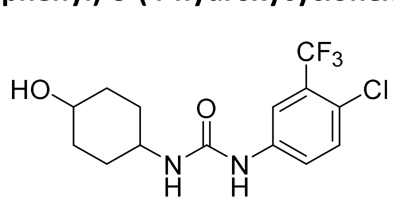

$C_{14}H_{16}ClF_3N_2O_2$
336.74 g/mol

4-Aminocyclohexanol (172 mg, 1.49 mmol, 1.1 eq.) and 1-chloro-4-isocyanato-2-(trifluoromethyl)benzene (300 mg, 1.35 mmol, 1.0 eq.) were added to DCM (10 mL). The reaction was stirred at room temperature overnight. The precipitate was filtered off, washed with DCM and purified by flash column chromatography (DCM/MeOH = 95/5) to yield the desired product (271 mg, 805 µmol, 60%) as a white solid.

TLC (DCM/MeOH = 95/5): R_f = 0.14 [UV]

^{1}H-NMR (400 MHz, DMSO-d_6): δ [ppm] = 8.77 (s, 1H), 8.06 (d, *J* = 1.5 Hz, 1H), 7.51 (s, 2H), 6.19 (d, *J* = 7.7 Hz, 1H), 4.54 (d, *J* = 4.4 Hz, 1H), 3.48-3.35 (m, 2H), 1.88-1.78 (m, 4H), 1.32-1.13 (m, 4H).
^{13}C-NMR (101 MHz, DMSO-d_6): δ [ppm] = 154.7, 140.6, 132.2, 127.1 (q, J = 30.4 Hz), 124.7, 122.6, 122.0, 121.8 (d, J = 1.7 Hz), 116.5 (q, J = 5.7 Hz), 68.5, 48.3, 34.3, 31.1.
ESI-HR-MS (m/z) (M-H$^+$) calcd. for $C_{14}H_{15}ClF_3N_2O_2^-$, 335.0780; found, 335.0780.

1-(*tert*-butyl)-3-(4-chloro-3-(trifluoromethyl)phenyl)urea (39)

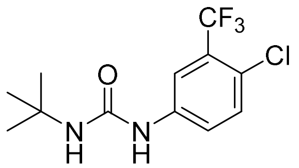

$C_{12}H_{14}ClF_3N_2O$
294.70 g/mol

tert-Butylamine (214 µL, 150 mg, 2.05 mmol, 1.1 eq.) was dissolved in dry DCM (10 mL) and 1-chloro-4-isocyanato-2-(trifluoromethyl)benzene (413 mg, 1.86 mmol, 1.0 eq.) was added. The reaction was stirred at room temperature overnight. The precipitate was filtered of to yield urea **39** (256 mg, 869 µmol, 47%) white powder.

^{1}H-NMR (400 MHz, DMSO-d_6): δ [ppm] = 8.73 (s, 1H), 8.06 (d, *J* = 2.5 Hz, 1H), 7.52 (d, *J* = 8.8 Hz, 1H), 7.45 (dd, *J* = 8.8, 2.5 Hz, 1H), 6.13 (s, 1H), 1.29 (s, 9H).
^{13}C-NMR (101 MHz, DMSO-d_6): δ [ppm] = 154.5, 140.7, 132.3, 127.1 (q, *J* = 30.3 Hz), 124.7, 122.5, 122.0, 121.7-121.3 (m), 116.2 (q, *J* = 5.6 Hz), 50.1, 29.3.
ESI-HR-MS (m/z) (M-H$^+$) calcd. for $C_{12}H_{13}ClF_3N_2O^-$, 293.0674; found, 293.0674.

4-(3-(4-chloro-3-(trifluoromethyl)phenyl)ureido)cyclohexane-1-carboxylic acid (40, pp-4)

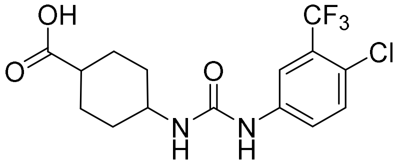

$C_{15}H_{16}ClF_3N_2O_3$
364.75 g/mol

4-Aminocyclohexanecarboxylic acid (388 mg, 2.71 mmol, 1.2 eq.) and 1-chloro-4-isocyanato-2-(trifluoromethyl)benzene (500 mg, 2.26 mmol, 1.0 eq.) were added successively to dry DCM (15 mL). The reaction was stirred at room temperature overnight. The volatile components were removed under reduced pressure and the residue was purified by flash column chromatography (ethyl acetate) to yield the desired product (38 mg, 104 µmol, 5%) as a white solid.

TLC (ethyl acetate): R_f = 0.10 [UV]

^{1}H-NMR (500 MHz, DMSO-d_6): [ppm] = 12.16 (s, 1H), 8.78 (s, 1H), 8.07 (d, *J* = 2.5 Hz, 1H), 7.55 (d, *J* = 8.8 Hz, 1H), 7.50 (dd, *J* = 8.8, 2.5 Hz, 1H), 6.49 (d, *J* = 7.8 Hz, 1H), 3.71 (s, 1H), 2.42-2.30 (m, 1H), 1.74-1.56 (m, 6H), 1.56-1.48 (m, 2H).
ESI-HR-MS (m/z) ($M-H^+$) calcd. for $C_{15}H_{15}ClF_3N_2O_3^-$, 363.0729; found, 363.0729.

1-(4-chloro-3-(trifluoromethyl)phenyl)-3-(3-methyltetrahydrofuran-3-yl)urea (41)

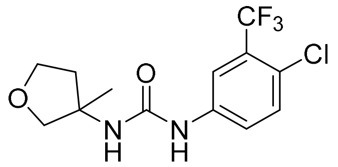

$C_{13}H_{14}ClF_3N_2O_2$
322.71 g/mol

3-Methyl-tetrahydrofuran-3-amine (126 mg, 1.24 mmol, 1.1 eq.) was dissolved in DCM (10 mL) and 1-chloro-4-isocyanato-2-(trifluoromethyl)benzene (250 mg, 1.13 mmol, 1.0 eq.) was added. The reaction was stirred at room temperature overnight and the volatile components were removed under reduced pressure. The residue was purified by flash column chromatography (DCM/MeOH = 98/2) to yield the desired product (233 mg, 722 µmol, 64%) as a white solid.

TLC (DCM/MeOH = 98/2): R_f = 0.09 [UV]
^{1}H-NMR (400 MHz, DMSO-d_6): [ppm] = 8.80 (s, 1H), 8.07 (d, *J* = 2.4 Hz, 1H), 7.54 (d, *J* = 8.8 Hz, 1H), 7.49 (dd, *J* = 8.8, 2.4 Hz, 1H), 6.53 (s, 1H), 3.84-3.76 (m, 3H), 3.53 (d, *J* = 8.7 Hz, 1H), 2.20-2.13 (m, 1H), 1.91-1.82 (m, 1H), 1.42 (s, 3H).
^{13}C-NMR (101 MHz, DMSO-d_6): [ppm] = 154.8, 140.4, 132.3, 127.1 (q, *J* = 30.4 Hz), 124.7, 122.8, 122.0, 121.9 (d, *J* = 1.8 Hz), 116.4 (q, *J* = 5.7 Hz), 77.8, 66.8, 59.3, 39.4, 23.7.
ESI-HR-MS (m/z) ($M-H^+$) calcd. for $C_{13}H_{13}ClF_3N_2O_2^-$, 321.0623; found, 321.0623.

1-(4-chloro-3-(trifluoromethyl)phenyl)-3-(4,4-difluorocyclohexyl)urea (42)

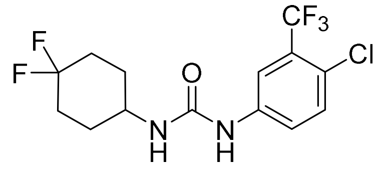

$C_{14}H_{14}ClF_5N_2O$
356.72 g/mol

4,4-Difloroaniline hydrochloride (256 mg, 1.49 mmol, 1.1 eq.) and Na_2CO_3 (144 mg, 1.35 mmol, 1.0 eq.) and 1-chloro-4-isocyanato-2-(trifluoromethyl)benzene (300 mg, 1.35 mmol, 1.0 eq.) were added to DCM (10 mL). The mixture was stirred at room temperature overnight. The volatile components were removed under reduced pressure and the residue was purified by flash column chromatography (DCM/MeOH = 97/3) to yield the desired product (319 mg, 894 µmol, 66%) as a white solid.

TLC (DCM): R_f = 0.16 [UV]

^{1}H-NMR (400 MHz, DMSO-d$_6$): ? [ppm] = 8.80 (s, 1H), 8.07 (d, *J* = 2.0 Hz, 1H), 7.60-7.48 (m, 2H), 6.44 (d, *J* = 7.6 Hz, 1H), 3.75-3.62 (m, 1H), 2.05-1.80 (m, 6H), 1.58-1.45 (m, 2H).
^{13}C-NMR (101 MHz, DMSO-d$_6$): ? [ppm] = 154.7, 140.5, 132.3, 127.1 (q, *J* = 30.5 Hz), 126.4, 124.7, 124.0, 122.8, 122.0, 121.9 (d, *J* = 1.7 Hz), 121.6, 116.6 (q, *J* = 5.7 Hz), 46.0, 31.7 (t, *J* = 24.1 Hz), 28.9-28.6 (m).
ESI-HR-MS (m/z) (M-H$^+$) calcd. for $C_{14}H_{13}ClF_5N_2O^-$, 355.0462; found, 355.0641.

1-(4-bromocyclohexyl)-3-(4-chloro-3-(trifluoromethyl)phenyl)urea (43)

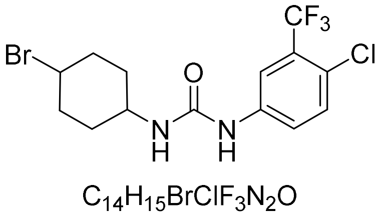

$C_{14}H_{15}BrClF_3N_2O$
399.64 g/mol

4-Bromooaniline hydrochloride (245 mg, 1.14 mmol, 1.1 eq.) and Na_2CO_3 (110 mg, 1.04 mmol, 1.0 eq.) and 1-chloro-4-isocyanato-2-(trifluoromethyl)benzene (230 mg, 1.04 mmol, 1.0 eq.) were added to DCM (10 mL). The mixture was stirred at room temperature overnight and the volatile components were removed under reduced pressure. The residue was purified by flash column chromatography (DCM/MeOH = 1/0→97/3) to yield the desired product (172 mg, 430 µmol, 41%) as a white solid.

TLC (DCM): R_f = 0.20 [UV]
^{1}H-NMR (400 MHz, DMSO-d$_6$): ? [ppm] = 8.76 (d, *J* = 7.8 Hz, 1H), 8.16-7.98 (m, 1H), 7.56-7.48 (m, 2H), 6.50 (d, *J* = 7.6 Hz, 1H), 4.60 (s, 1H), 3.69-3.58 (m, 1H), 2.25-1.28 (m, 8H).
^{13}C-NMR (101 MHz, DMSO-d$_6$): ? [ppm] = 154.6, 140.5, 132.2, 127.1 (q, *J* = 30.5 Hz), 124.7, 122.7, 122.0, 121.9-121.8 (m), 116.5 (q, *J* = 5.7 Hz), 54.6, 47.0, 33.5, 29.2.
ESI-HR-MS (m/z) (M-H$^+$) calcd. for $C_{14}H_{14}BrClF_3N_2O^-$, 396.9936; found, 396.9936.

The spectral complexity results from the presence of diastereomers.

1-(4-chloro-3-(trifluoromethyl)phenyl)-3-(cyclohexylmethyl)urea (44)

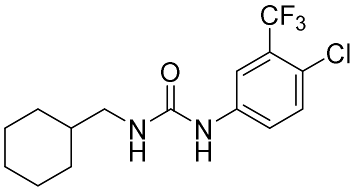

$C_{15}H_{18}ClF_3N_2O$
334.77 g/mol

Cyclohexanemethylamine (200 mg, 1.77 mmol, 1.1 eq.) was dissolved in dry DCM (10 mL) and 1-chloro-4-isocyanato-2-(trifluoromethyl)benzene (356 mg, 1.61 mmol, 1.0 eq.) was added. The reaction was stirred at room temperature overnight. The volatile organic components

were removed under reduced pressure and the residue was purified by flash column chromatography (DCM) to yield the desired product (316 mg, 944 µmol, 59%) as a white solid.

TLC (DCM): R_f = 0.16 [UV]
^{1}H-NMR (400 MHz, DMSO-d_6): δ [ppm] = 8.87 (s, 1H), 8.07 (d, *J* = 2.0 Hz, 1H), 7.56-7.48 (m, 2H), 6.33 (t, *J* = 5.8 Hz, 1H), 2.94 (t, *J* = 6.3 Hz, 2H), 1.72-1.57 (m, 5H), 1.45-1.32 (m, 1H), 1.24-1.05 (m, 3H), 0.95-0.82 (m, 2H).
^{13}C-NMR (101 MHz, DMSO-d_6): δ [ppm] = 155.4, 140.7, 132.2, 127.1 (q, *J* = 30.4 Hz), 124.7, 122.6, 122.0, 121.7 (d, *J* = 1.7 Hz), 116.5 (q, *J* = 5.8 Hz), 45.8, 38.3, 30.8, 26.5, 25.9.
ESI-HR-MS (m/z) (M-H$^+$) calcd. for $C_{15}H_{17}ClF_3N_2O^-$, 333.0987; found, 333.0987.

Phenyl (4-chloro-3-(trifluoromethoxy)phenyl)carbamate (45-1)

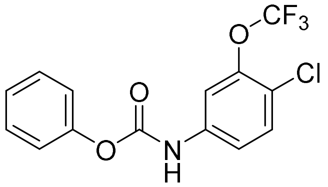

$C_{14}H_9ClF_3NO_3$
331.68 g/mol

4-Chloro-3-(trifluoromethoxy)aniline (500 mg, 2.36 mmol, 1.0 eq.) was dissolved in DCM (5 mL) and pyridine (209 µL, 206 mg, 2.60 mmol, 1.1 eq.) was added. The solution was cooled to 0 °C and phenyl chloroformate (326 µL, 407 mg, 2.60 mmol, 1.1 eq.) was added dropwise. The solution was stirred and allowed to reach room temperature overnight. The reaction mixture was washed with water (3×5 mL) and dried over anhydrous Na_2SO_4. The volatile components were removed under reduced pressure. Flash column chromatography (hexane/ethyl acetate = 95/5) yielded the product (298 mg, 897 µmol, 38%) as a white solid.

^{1}H-NMR (400 MHz, DMSO-d_6): δ [ppm] = 10.68 (s, 1H), 7.82 (s, 1H), 7.64 (d, *J* = 8.9 Hz, 1H), 7.49 (dd, *J* = 8.9, 2.4 Hz, 1H), 7.47-7.40 (m, 2H), 7.30- 7.23 (m, 3H).

1-(4-chloro-3-(trifluoromethoxy)phenyl)-3-(2,2-dimethyl-1,3-dioxan-5-yl)urea (45)

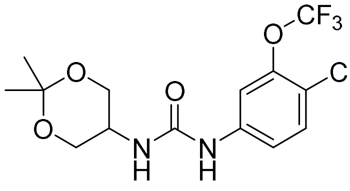

$C_{14}H_{16}ClF_3N_2O_4$
368.74 g/mol

Phenyl (4-chloro-3-(trifluoromethoxy)phenyl)carbamate (45-1) (250 mg, 754 µmol, 1.1 eq.) was dissolved in DMF (7.5 mL). Triethylamine (139 mg, 1.37 mmol, 2.0 eq.) and 2,2-dimethyl-1,3-dioxan-5-amine (89.9 mg, 685 µmol, 1.0 eq.) were added. The solution was stirred 72 hours at room temperature. Water (25 mL) was added and the mixture was cooled down to 4 °C. The precipitate was filtered off and discarded. The remaining liquid phase was extracted

with EtOAc (3×75 mL). The combined organic phases were dried over anhydrous Na_2SO_4. The volatile components were removed under reduced pressure. The residue was purified by flash column chromatography (hexane/ethyl acetate = 1/2) to yield the product (61.1 mg, 116 µmol, 24%) as a white solid.

TLC (hexane/ethyl acetate = 1/2): R_f = 0.16 [UV] **^{1}H-NMR** (400 MHz, DMSO-d$_6$): ? [ppm] = 9.13 (s, 1H), 7.96-7.82 (m, 1H), 7.50 (d, *J* = 8.8 Hz, 1H), 7.21 (dd, *J* = 8.9, 2.5 Hz, 1H), 6.66 (d, *J* = 8.0 Hz, 1H), 4.07-3.99 (m, 2H), 3.66-3.53 (m, 3H), 1.39 (s, 3H), 1.35 (s, 3H). **^{13}C-NMR** (101 MHz, DMSO-d$_6$): ? [ppm] = 154.3, 144.0, 140.9, 130.8, 117.5, 116.7, 97.7, 62.9, 43.3, 26.7, 20.8. **ESI-HR-MS** (m/z) (M-H$^+$) calcd. for $C_{14}H_{16}ClF_3N_2O_4^-$, 367.0678; found, 367.0677.

1-(4-chloro-3-(trifluoromethoxy)phenyl)-3-morpholinourea (46)

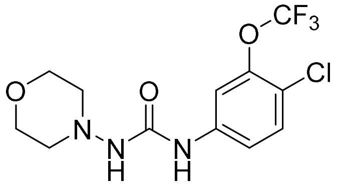

$C_{12}H_{13}ClF_3N_3O_3$
339.70 g/mol

To a solution of phenyl (4-chloro-3-(trifluoromethoxy)phenyl)carbamate (45-1) (250 mg, 754 µmol, 1.1 eq.) in DMF (7.5 mL) NEt_3 (190 µL, 139 mg, 1.37 mmol, 2.0 eq.) and morpholin-4-amine (70.0 mg, 685 µmol, 1.0 eq.) were added. The solution was stirred at room temperature overnight. Water (25 mL) was added, and the mixture was cooled down to 4 °C. The precipitate was filtered off and discarded. The liquid phase was extracted with EtOAc (3×75 mL). The combined organic phases were dried over anhydrous Na_2SO_4. The volatile components were removed under reduced pressure. The residue was washed with hexane to yield the product (111 mg, 327 µmol, 48%). as a white solid.

TLC (hexane/ethyl acetate = 1/1): R_f = 0.13 [UV] **^{1}H-NMR** (400 MHz, DMSO-d$_6$): ? [ppm] = 8.94 (s, 1H), 8.06-7.96 (m, 2H), 7.65 (dd, *J* = 8.9, 2.4 Hz, 1H), 7.53 (d, *J* = 8.9 Hz, 1H), 3.70 (s, 4H), 2.73 (s, 4H). **^{13}C-NMR** (101 MHz, DMSO-d$_6$): ? [ppm] = 154.5, 143.8, 140.3, 130.4, 119.1, 117.6, 112.8, 65.8, 55.7.
ESI-HR-MS (m/z) (M-H$^+$) calcd. for $C_{12}H_{12}ClF_3N_3O_3^-$, 338.0525; found, 338.0524.

1-(4-fluoro-3-(trifluoromethyl)phenyl)-3-(3-methyltetrahydrofuran-3-yl)urea (47)

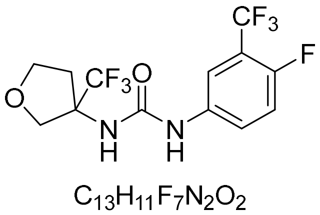

$C_{13}H_{11}F_7N_2O_2$
360.23 g/mol

To dry DCM (10 mL) 3-(trifluoromethyl)tetrahydrofuran-3-amine hydrochloride (257 mg, 1.34 mmol, 1.1 eq.), Na_2CO_3 (129 mg, 1.22 mmol, 1.0 eq.) and 1-fluoro-4-isocyanato-2-(trifluoromethyl)benzene (250 mg, 1.22 mmol, 1.0 eq.) were added. The reaction was stirred at room temperature overnight, water (10 mL) was added and the mixture was stirred for 1 h at room temperature. The precipitate was filtered off, dissolved in MeOH (10 mL) and the volatile components were removed under reduced pressure to obtain the desired product (28 mg, 355 µmol, 29%) as a white solid.

^{1}H-NMR (500 MHz, DMSO-d_6): [ppm] = 8.79 (s, 1H), 7.94 (dd, *J* = 6.4, 2.6 Hz, 1H), 7.58-7.51 (m, 1H), 7.45-7.37 (m, 1H), 7.23 (s, 1H), 4.21 (d, *J* = 10.5 Hz, 1H), 4.04 (d, *J* = 10.5 Hz, 1H), 3.91 (q, *J* = 6.7, 6.0 Hz, 1H), 3.79 (q, *J* = 7.9 Hz, 1H), 2.32 (t, *J* = 7.6 Hz, 2H).
^{13}C-NMR (126 MHz, DMSO-d_6): [ppm] = 155.0 (d, *J* = 2.0 Hz), 154.3, 153.0 (d, *J* = 2.1 Hz), 130.5, 128.3, 126.3, 126.0, 124.6 (d, *J* = 7.9 Hz), 123.7, 122.0, 119.8, 118.0 (d, *J* = 21.4 Hz), 116.8 (qd, *J* = 32.0, 12.9 Hz), 116.2, 73.0, 66.9, 64.5 (q, *J* = 27.8 Hz), 34.7.
ESI-HR-MS (m/z) (M-H$^+$) calcd. for $C_{13}H_{11}F_7N_2O_2^-$, 359.0636; found, 359.0634.

1-(3-(trifluoromethyl)tetrahydrofuran-3-yl)-3-(2,3,4-trifluorophenyl)urea (48)

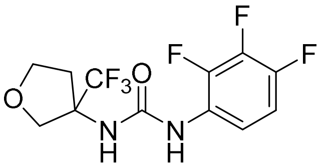

$C_{12}H_{10}F_6N_2O_2$
328.21 g/mol

To dry DCM (10 mL) 3-(trifluoromethyl)tetrahydrofuran-3-amine hydrochloride (304 mg, 1.59 mmol, 1.1 eq.), Na_2CO_3 (153 mg, 1.44 mmol, 1.0 eq.) and 2,3,4-trifluorophenyl isocyanate (250 mg, 1.44 mmol, 1.0 eq.) were added. The reaction was stirred at room temperature overnight. Water (10 mL) was added and the mixture was stirred for 1 h at room temperature. The phases were separated and the organic phase was washed with brine (1×10 mL). The volatile organic components were removed under reduced pressure. The residue was purified by flash column chromatography (hexane/ethyl acetate = 9/1→7/3) to yield the desired product (320 mg, 975 µmol, 68%) as a white solid.

TLC (hexane/ethyl acetate = 9/1): R_f = 0.24 [UV]
^{1}H-NMR (400 MHz, DMSO-d_6): [ppm] = 8.46 (s, 1H), 7.90-7.74 (m, 1H), 7.46 (s, 1H), 7.18 (q, *J* = 9.8 Hz, 1H), 4.19 (d, *J* = 10.4 Hz, 1H), 4.04 (d, *J* = 11.4 Hz, 1H), 3.96-3.87 (m, 1H), 3.82 (q, J = 7.9 Hz, 1H), 2.38-2.24 (m, 2H).

^{13}C-NMR (101 MHz, DMSO-d$_6$): δ [ppm] = 153.9, 146.8 (dd, *J* = 9.8, 2.1 Hz), 144.4 (dd, *J* = 9.8, 2.2 Hz), 143.0 (dd, *J* = 11.7, 3.0 Hz), 141.2-139.8 (m), 138.3 (dd, *J* = 16.3, 13.8 Hz), 131.3, 128.4, 126.0 (dd, *J* = 8.0, 3.1 Hz), 125.6, 122.8, 115.3, 112.0 (dd, *J* = 17.4, 2.6 Hz), 72.8, 67.0, 64.6 (q, *J* = 27.9 Hz), 34.6.
ESI-HR-MS (m/z) (M-H$^+$) calcd. for $C_{12}H_9F_6N_2O_2^-$, 327.0574; found, 327.0573.

1-(3,4-dimethoxyphenyl)-3-(1-methylcyclopentyl)urea (49)

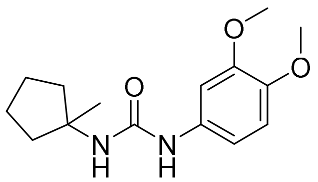

$C_{15}H_{22}N_2O_3$
278.35 g/mol

To DCM (10 mL) 1-Methyl-1-cyclopentylamine hydrochloride (208 mg, 1.53 mmol, 1.1 eq.) and Na_2CO_3 (148 mg, 1.43 mmol, 1.0 eq.) and 3,4-dimethoxyphenyl isocyanate (250 mg, 1.48 mmol, 1.0 eq.) were added. The reaction was stirred overnight at room temperature. Water (10 mL) was added and the mixture was stirred for another 1 h at room temperature. The phases were separated and the organic phase was washed with brine (1×10 mL). The volatile organic components were removed under reduced pressure and the residue was purified by flash column chromatography (DCM/MeOH = 95/5) to yield the desired product (136 mg, 489 µmol, 35%) as a white solid.

TLC (DCM/MeOH = 95/5): R*f* = 0.14 [UV]
^{1}H-NMR (400 MHz, DMSO-d$_6$): δ [ppm] = 8.05 (s, 1H), 7.17 (d, *J* = 2.4 Hz, 1H), 6.80 (d, *J* = 8.7 Hz, 1H), 6.70 (dd, *J* = 8.6, 2.4 Hz, 1H), 5.93 (s, 1H), 3.71 (s, 3H), 3.68 (s, 3H), 1.95-1.87 (m, 2H), 1.72-1.57 (m, 4H), 1.56-1.47 (m, 2H), 1.35 (s, 3H).
^{13}C-NMR (101 MHz, DMSO-d$_6$): δ [ppm] = 155.1, 149.3, 143.7, 135.0, 113.2, 109.5, 103.5, 60.1, 56.4, 55.8, 26.4, 23.7.
ESI-HR-MS (m/z) (M-H$^+$+FA) calcd. for $C_{16}H_{23}N_2O_5^-$, 323.1612; found, 323.1611.

1-cyclopentyl-3-(3-fluoro-4-morpholinophenyl)urea (50)

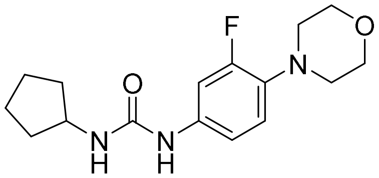

$C_{16}H_{22}FN_3O_2$
307.37 g/mol

3-Fluoro-4-(morpholinyl)aniline (388 mg, 1.98 mmol, 1.1 eq.) was dissolved in DCM (10 mL) and Cyclopentyl isocyanate (200 mg, 1.80 mmol, 1.0 eq.) was added. The reaction was stirred

for 48 h at room temperature. The precipitate was filtered off to yield urea **50** (142 mg, 462 µmol, 26%) as a white powder.

^{1}H-NMR (400 MHz, DMSO-d_6): ⍰ [ppm] = 8.26 (s, 1H), 7.49-7.36 (m, 1H), 6.96-6.84 (m, 2H), 6.12 (d, *J* = 7.2 Hz, 1H), 3.92 (h, *J* = 6.7 Hz, 1H), 3.74-3.69 (m, 4H), 2.95-2.87 (m, 4H), 1.87-1.78 (m, 2H), 1.68-1.58 (m, 2H), 1.58-1.48 (m, 2H), 1.39-1.30 (m, 2H).
^{13}C-NMR (101 MHz, DMSO-d_6): ⍰ [ppm] = 156.6, 155.2, 154.2, 136.6 (d, *J* = 11.1 Hz), 133.8 (d, *J* = 9.2 Hz), 119.8 (d, *J* = 4.3 Hz), 113.8 (d, *J* = 2.8 Hz), 106.4 (d, *J* = 25.7 Hz), 66.7, 51.5 (d, *J* = 2.5 Hz), 51.3, 33.3, 23.6.
ESI-HR-MS (m/z) (M-H$^+$+FA) calcd. for $C_{11}H_{23}FN_3O_4^-$, 352.1678; found, 352.1677.

1-cyclopentyl-3-(3-fluoro-4-(pyrrolidin-1-yl)phenyl)urea (51)

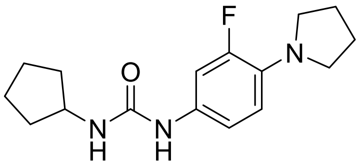

$C_{16}H_{22}FN_3O$
291.37 g/mol

Cyclopentyl isocyanate (200 mg, 1.80 mmol, 1.0 eq.) was added to a solution of 3-fluoro-4-(pyrrolidin-1-yl)aniline (357 mg, 1.98 mmol, 1.1 eq.) in DCM (10 mL). The solution was stirred at room temperature overnight. The precipitate was filtered off and washed with DCM to yield the desired product (288 mg, 988 µmol, 55%) as a white solid.

^{1}H-NMR (400 MHz, DMSO-d_6): ⍰ [ppm] = 8.07 (s, 1H), 7.31 (dd, *J* = 16.2, 2.4 Hz, 1H), 6.84 (dd, *J* = 8.6, 2.0 Hz, 1H), 6.73-6.60 (m, 1H), 6.03 (d, *J* = 7.2 Hz, 1H), 3.96-3.83 (m, 1H), 3.23-3.15 (m, 4H), 1.91-1.77 (m, 6H), 1.69-1.58 (m, 2H), 1.58-1.47 (m, 2H), 1.39-1.29 (m, 2H).
^{13}C-NMR (101 MHz, DMSO-d_6): ⍰ [ppm] = 155.3, 153.2, 150.9, 132.5 (d, J = 10.2 Hz), 132.0 (d, J = 10.1 Hz), 116.3 (d, J = 6.1 Hz), 114.3 (d, J = 2.6 Hz), 107.0 (d, J = 25.8 Hz), 51.3, 50.1 (d, J = 4.3 Hz), 33.3, 24.8, 23.6.
ESI-HR-MS (m/z) (M-H$^+$+FA) calcd. for $C_{17}H_{23}FN_3O_3^-$, 336.1729; found, 336.1727.

4,6-difluoro-N-(1-methylcyclopentyl)-1H-indole-2-carboxamide (52)

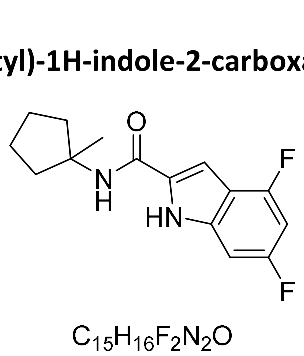

$C_{15}H_{16}F_2N_2O$
278.30 g/mol

1-Methyl-1-cyclopentylamine hydrochloride (268 mg, 1.98 mmol, 1.3 eq.), 4,6-difluoro-1H-indole-2-carboxylic acid (300 mg, 1.52 mmol, 1.0 eq.), 1-ethyl-3-(3-dimethylaminopropyl)carbodiimide hydrochloride (583 mg, 3.04 mmol, 2.0 eq.), 1-

hydroxybenzotriazol (411 mg, 3.04 mmol, 2.0 eq.) and *N*, *N*-diisopropylethylamine (1.59 mL, 1.18 g, 9.13 mmol, 6.0 eq.) were dissolved in DMF (15 mL) and the reaction was stirred at room temperature over the weekend. Water (25 mL) was added and the precipitate was filtered off. The precipitate was purified by flash column chromatography (hexane/ethyl acetate = 9/1) to yield the desired product (236 mg, 848 µmol, 56%) as a white solid.

TLC (hexane/ethyl acetate = 9/1): R_f = 0.13 [UV]
^{1}H-NMR (400 MHz, DMSO-d_6): [ppm] = 11.90 (s, 1H), 7.95 (s, 1H), 7.33 (s, 1H), 7.05-7.01 (m, 2H), 6.84 (td, *J* = 10.4, 1.9 Hz, 1H), 2.22-2.12 (m, 2H), 1.75-1.55 (m, 4H), 1.44 (s, 3H).
^{13}C-NMR (101 MHz, DMSO-d_6): [ppm] = 160.7 (d, *J* = 12.1 Hz), 160.5, 158.4 (d, *J* = 12.1 Hz), 157.4 (d, *J* = 15.5 Hz), 154.9 (d, *J* = 15.5 Hz), 137.9 (dd, *J* = 15.2, 13.4 Hz), 134.0 (d, *J* = 3.3 Hz), 113.6 (d, *J* = 22.6 Hz), 98.9, 96.0-94.5 (m), 61.5, 39.1, 25.8, 23.8.
ESI-HR-MS (m/z) ($M-H^+$) calcd. for $C_{15}H_{15}F_2N_2O^-$, 277.1158; found, 277.1157.

Probes

4-nitrophenyl (1-ethynylcyclohexyl)carbamate (pp-1)

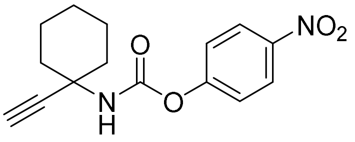

$C_{15}H_{16}N_2O_4$
288.30 g/mol

1-Ethynylcyclohexylamine (250 mg, 2.03 mmol, 1.0 eq.) and pyridine (18 µL, 18 mg, 223 µmol, 0.1 eq.) were dissolved in DCM (10 mL). The solution was cooled to 4 °C using an icebath and 4-nitrophenyl chloroformate (450 mg, 2.23 mmol, 1.1 eq.) dissolved in DCM (10 mL) was added. The reaction was stirred and allowed to reach room temperature overnight. The precipitate was filtered off and the volatile components were removed under reduced pressure. The residue was purified by flash column chromatography (hexane/ethyl acetate = 8/2) to yield the desired product (355 mg, 1.23 mmol, 61%) as a white solid. The product was used without further purification.

TLC (hexane/ethyl acetate): R_f = 0.29 [UV]
ESI-HR-MS (m/z) ($M+H^+$) calcd. for $C_{15}H_{17}N_2O_4^+$, 289.1183; found, 289.1183.

1-(4-azidophenyl)-3-(1-ethynylcyclohexyl)urea (227-p1)

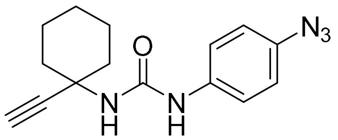

$C_{15}H_{17}N_5O$
283.34 g/mol

Compound **227-p1-1** (200 mg, 694 µmol, 1.0 eq.) was dissolved in DMF (10 mL) and trietylamine (218 µL, 158 mg, 1.56 mmol, 2.3 eq.) and 4-azidoaniline hydrochloride (148 mg, 867 µmol, 1.3 eq.) were added. The reaction was stirred for 4 days at room temperature under the exclusion of light. Water (15 mL) was added and the precipitate was filtered of. The precipitate was purified by flash column chromatography (hexane/ethyl acetate = 2/1) to yield the probe **227-p1** (34 mg, 120 µmol, 17%) as a white solid.

TLC (hexane/ethyl acetate): R_f = 0.51 [UV]
^{1}H-NMR (400 MHz, DMSO-d_6): ⍰ [ppm] = 8.42 (s, 1H), 7.44-7.39 (m, 2H), 7.02-6.97 (m, 2H), 6.30 (s, 1H), 3.17 (s, 1H), 2.02-1.94 (m, 2H), 1.79-1.69 (m, 2H), 1.59-1.46 (m, 5H), 1.33-1.21 (m, 1H).
^{13}C-NMR (101 MHz, DMSO-d_6): ⍰ [ppm] = 153.6, 137.7, 131.7, 119.4, 118.9, 87.1, 72.9, 50.1, 36.8, 24.8, 21.9.
ESI-HR-MS (m/z) (M-H$^+$+FA) calcd. for $C_{16}H_{18}N_5O_3^-$, 328.1415; found, 328.1414.

***tert*-butyl 4-(((4-nitrophenoxy)carbonyl)amino)benzoate (pp-2)**

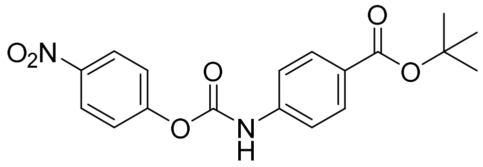

$C_{18}H_{18}N_2O_6$
358.35 g/mol

tert-Butyl 4-aminobenzoate (500 mg, 2.59 mmol, 1.0 eq.) and pyridine (24 µL, 23mg, 285 µmol, 0.1 eq.) were dissolved in DCM (10 mL). The solution was cooled in an icebath to 4 °C and 4-nitrophenyl chloroformate (574mg, 2.85 mmol, 1.1 eq.) was added slowly. The reaction was stirred for 4 days and allowed to reach room temperature. The precipitate was filtered off to yield the desired product (362 mg) as a white solid. The product was used without further purification.

4-(3-cyclohexylureido)benzoic acid (pp-3)

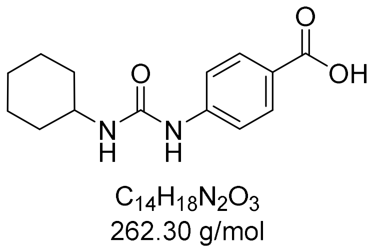

$C_{14}H_{18}N_2O_3$
262.30 g/mol

Compound **227-p2-2** (362 mg, 1.01 mmol, 1.0 eq.) was added to THF (10 mL) and cyclohexylamine (145 µL, 125 mg, 1.26 mmol, 1.25 eq.) and *N*, *N*-diisopropylethylamine (220 µL, 163 mg, 1.26 mmol, 1.25 eq.) were added. The reaction was stirred at room temperature overnight and the volatile components were removed under reduced pressure. The residue was purified by flash column chromatography (DCM/MeOH =

98/2→95/5→95/5+1% AcOH) to yield the saponified product (90 mg, 343 µmol, 34%) as an off white solid.

^{1}H-NMR (500 MHz, DMSO-d$_6$): [ppm] = 8.89 (s, 1H), 7.80 (d, *J* = 8.4 Hz, 2H), 7.45 (d, *J* = 8.4 Hz, 2H), 6.45 (d, *J* = 7.5 Hz, 1H), 3.51-3.42 (m, 1H), 1.83-1.77 (m, 2H), 1.69-1.63 (m, 2H), 1.35-1.12 (m, 6H).
^{13}C-NMR (126 MHz, DMSO-d$_6$): [ppm] = 154.6, 130.8, 116.8, 48.1, 33.3, 25.7, 24.8.
ESI-HR-MS (m/z) (M-H$^+$) calcd. for $C_{14}H_{17}N_2O_3^-$, 261.1245; found, 261.1244.

2-(3-(but-3-yn-1-yl)-3H-diazirin-3-yl)ethyl 4-(3-cyclohexylureido)benzoate (227-p2)

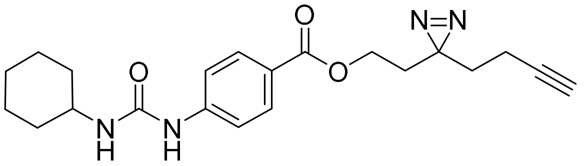

$C_{21}H_{26}N_4O_3$
382.46 g/mol

The minimal photocrosslinker (47 mg, 343 µmol, 1.5 eq.) was dissolved in DMF (1 mL) and compound **227-p2-1** (60 mg, 229 µmol, 1.0 eq.), *N*, *N*-dimethyl-4-aminopyridine (28 mg, 229 µmol, 1.0 eq.) and 1-ethyl-3-(3-dimethylaminopropyl)carbodiimide hydrochloride (75 mg, 389 µmol, 1.7 eq.) were added. The reaction was stirred overnight at room temperature under the exclusion of light. The volatile components were removed under reduced pressure and the residue was purified by flash column chromatography (DCM/MeOH = 99/1) to yield the probe **227-p2** (51 mg, 132 µmol, 58%) as a white solid.

TLC (DCM/MeOH = 99/1): R$_f$ = 0.33 [UV]
^{1}H-NMR (500 MHz, MeOH-d$_4$): [ppm] = 9.51 (d, *J* = 8.9 Hz, 2H), 9.04 (d, *J* = 8.9 Hz, 2H), 5.74 (t, *J* = 6.2 Hz, 2H), 5.17-5.10 (m, 1H), 3.84 (t, *J* = 2.7 Hz, 1H), 3.63 (td, *J* = 7.4, 2.6 Hz, 2H), 3.51-3.47 (m, 2H), 3.45 (t, *J* = 6.2 Hz, 2H), 3.34-3.29 (m, 2H), 3.26 (t, *J* = 7.4 Hz, 2H), 3.22-3.17 (m, 1H), 3.02-2.92 (m, 2H), 2.86-2.76 (m, 3H).
^{13}C-NMR (126 MHz, MeOH-d$_4$): [ppm] = 166.2, 155.4, 144.8, 130.4, 122.7, 117.0, 82.2, 69.0, 59.3, 33.0, 32.1, 31.8, 26.2, 25.3, 24.6, 12.4.
ESI-HR-MS (m/z) (M-H$^+$) calcd. for $C_{21}H_{25}N_4O_3^-$, 381.1932; found, 381.1933.

2-(3-(but-3-yn-1-yl)-3H-diazirin-3-yl)ethyl 4-(3-(4-chloro-3-(trifluoromethyl) phenyl)ureido)cyclohexane-1-carboxylate (227-p3)

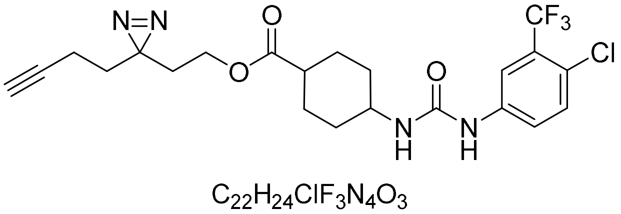

$C_{22}H_{24}ClF_3N_4O_3$
484.90 g/mol

The minimal photocrosslinker (14 mg, 97.8 µmol, 1.5 eq.) was dissolved in DMF (1 mL) and compound **40** (24 mg, 65.3 µmol, 1.0 eq.), *N*, *N*-dimethyl-4-aminopyridine (8 mg, 65.3 µmol, 1.0 eq.) and 1-ethyl-3-(3-dimethylaminopropyl)carbodiimide hydrochloride (21 mg, 111 µmol, 1.7 eq.) were added. The reaction was stirred overnight at room temperature under the exclusion of light. The volatile components were removed under reduced pressure and the residue was purified by flash column chromatography (DCM/MeOH = 98/2) and preparative HPLC to yield the probe **227-p3** (10 mg, 20 µmol, 32%) as a white solid.

TLC (DCM/MeOH = 98/2): R_f = 0.21 [UV]
^{1}H-NMR (500 MHz, MeOH-d_4): [ppm] = 7.93 (d, *J* = 2.5 Hz, 1H), 7.51 (dd, *J* = 8.7, 2.4 Hz, 1H), 7.47-7.42 (m, 1H), 3.99 (t, *J* = 6.1 Hz, 2H), 3.80-3.73 (m, 1H), 2.59-2.52 (m, 1H), 2.31-2.28 (m, 1H), 2.07-2.01 (m, 2H), 1.98-1.90 (m, 2H), 1.81-1.74 (m, 6H), 1.68-1.63 (m, 4H).
^{13}C-NMR (126 MHz, MeOH-d_4): [ppm] = 175.0, 155.4, 139.3, 131.5, 127.8 (d, *J* = 30.9 Hz), 124.0, 123.3, 122.2, 121.8, 116.7 (d, *J* = 5.5 Hz), 82.1, 69.0, 59.0, 32.0, 31.7, 29.4, 27.6, 26.2, 24.5, 12.4.
ESI-HR-MS (m/z) (M-H$^+$+FA) calcd. for $C_{23}H_{25}ClF_3N_4O_5^-$, 529.1471; found, 529.1470.

D3

((1-(2,5-dichlorophenyl)vinyl)oxy)trimethylsilane (D3-1)

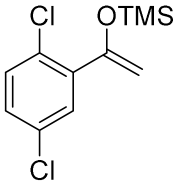

$C_{11}H_{14}Cl_2OSi$
261.22 g/mol

2,5-Dichloroacetophenone (500 mg, 2.65 mmol, 1.0 eq.) and triethylamine (550 µL, 401 mg, 397 mmol, 1.5 eq.) were dissolved in ACN (3 mL) and NaI (555 mg, 3.70 mmol, 1.4 eq.) was added. The mixture was cooled 4 °C with an ice bath and TMSCl (437 µL, 374 mg, 344 mmol, 1.3 eq.) was added. The reaction was stirred and allowed to reach room temperature overnight. The volatile components were removed under reduced pressure. The residue was washed with hexane (5×10 mL). The hexane fractions were pooled and the volatile components were removed under reduced pressure to yield the desired product (527 mg) as a slightly yellow oil. The product was used without further purification.

1-(2,5-dichlorophenyl)-3-(dimethylamino)propan-1-one (D3)

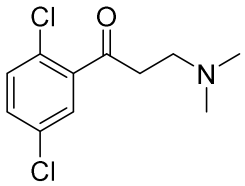

$C_{11}H_{13}Cl_2NO$
246.13 g/mol

Product **D3-1** (527 mg) was dissolved in dichloromethane (DCM) (15 mL) and *N*, *N*-dimethylmethyleniminium iodid (Eschenmoser's salt, 411 mg, 2.22 mmol 1.1 eq.) was added. The reaction was stirred at room temperature overnight and washed with brine (2×15 mL). The volatile organic compounds were removed under reduced pressure. The residue was recrystallized from acetone/DCM to yield the desired product (66 mg, 268 µmol, 13%) as a white solid.

^{1}H-NMR (300 MHz, $CDCl_3$): ? [ppm] = 7.60-7.56 (m, 1H), 7.44-7.36 (m, 2H), 3.74 (t, *J* = 6.9 Hz, 2H), 3.52-3.44 (m, 2H), 2.87 (s, 3H), 2.85 (s, 3H).
^{13}C-NMR (75 MHz, $CDCl_3$): ? [ppm] = 196.8, 138.2, 133.6, 132.8, 132.2, 129.6, 129.5, 52.4, 43.4, 37.8.
ESI-HR-MS (m/z) ($M+H^+$) calcd. for $C_{11}H_{14}Cl_2NO$, 246.0447; found, 246.0447.

NMR Spectra

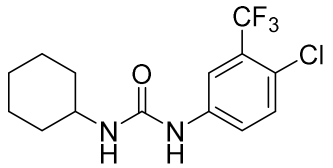

$C_{14}H_{16}ClF_3N_2O$
320.74 g/mol

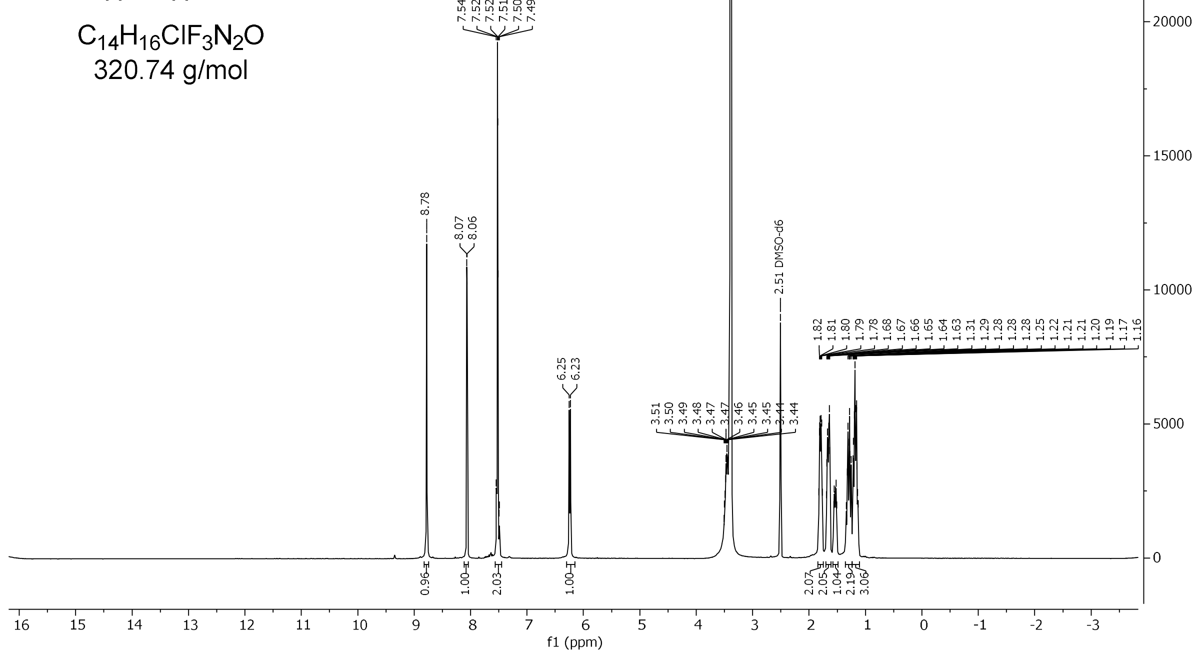

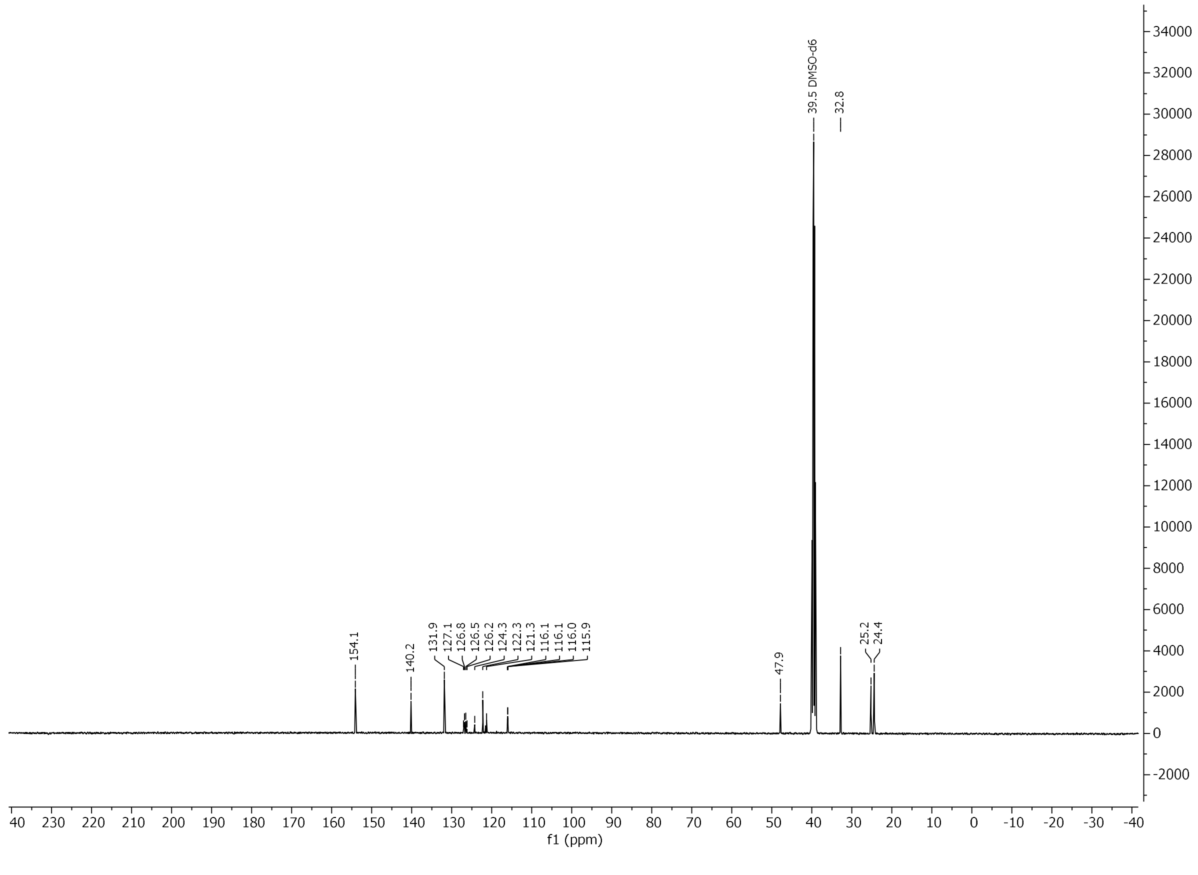

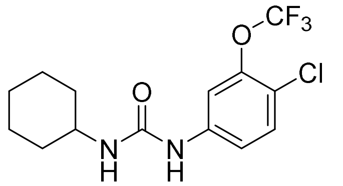

$C_{14}H_{16}ClF_3N_2O_2$
336.74 g/mol

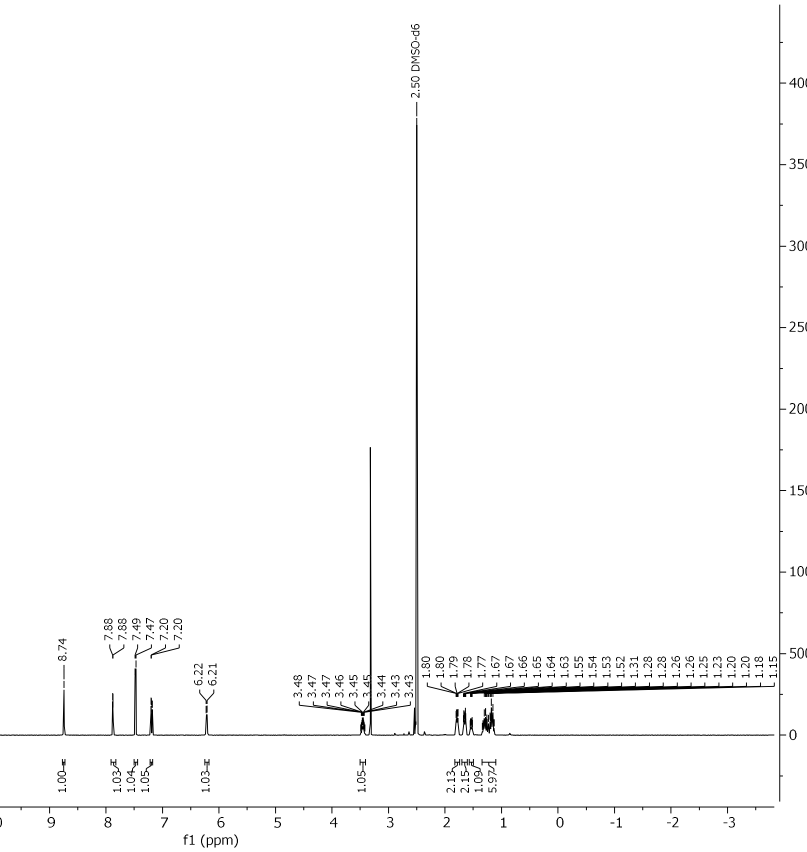

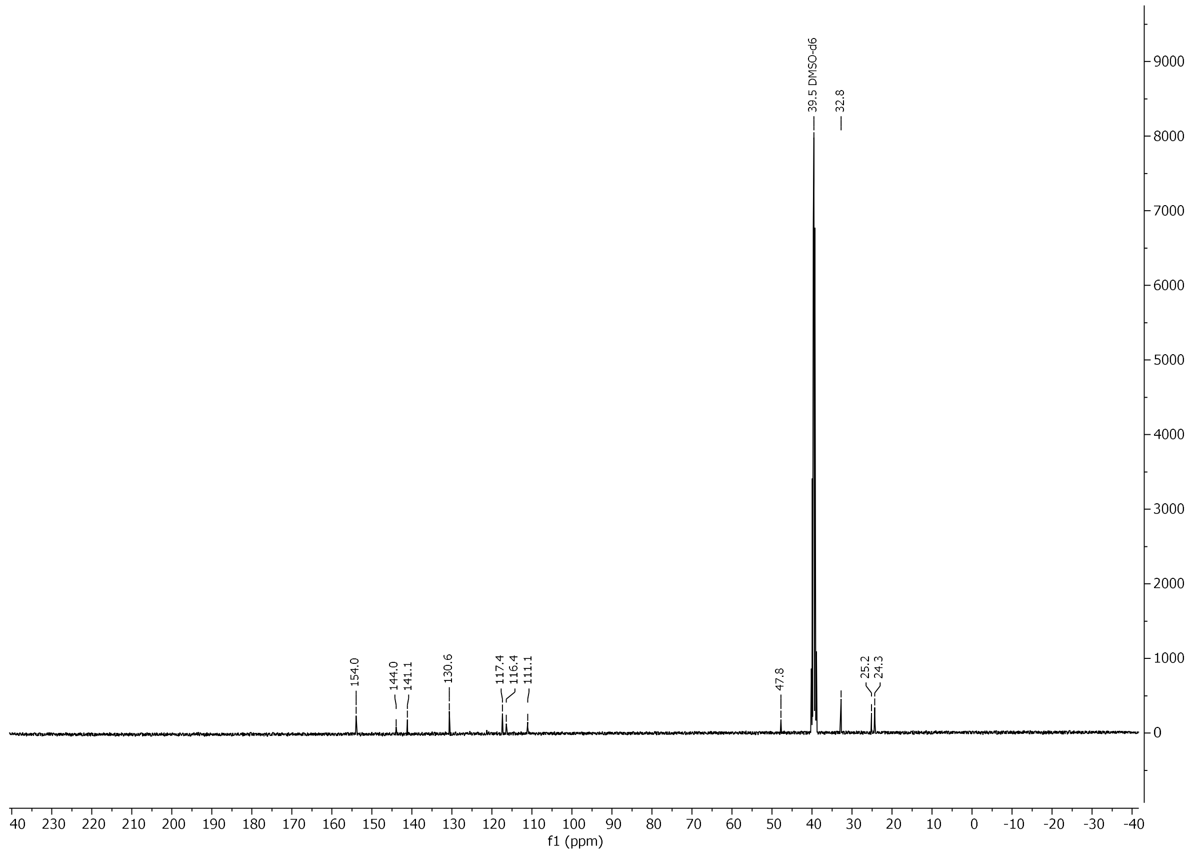

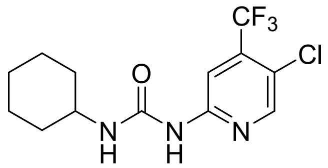

$C_{13}H_{15}ClF_3N_3O$

321.73 g/mol

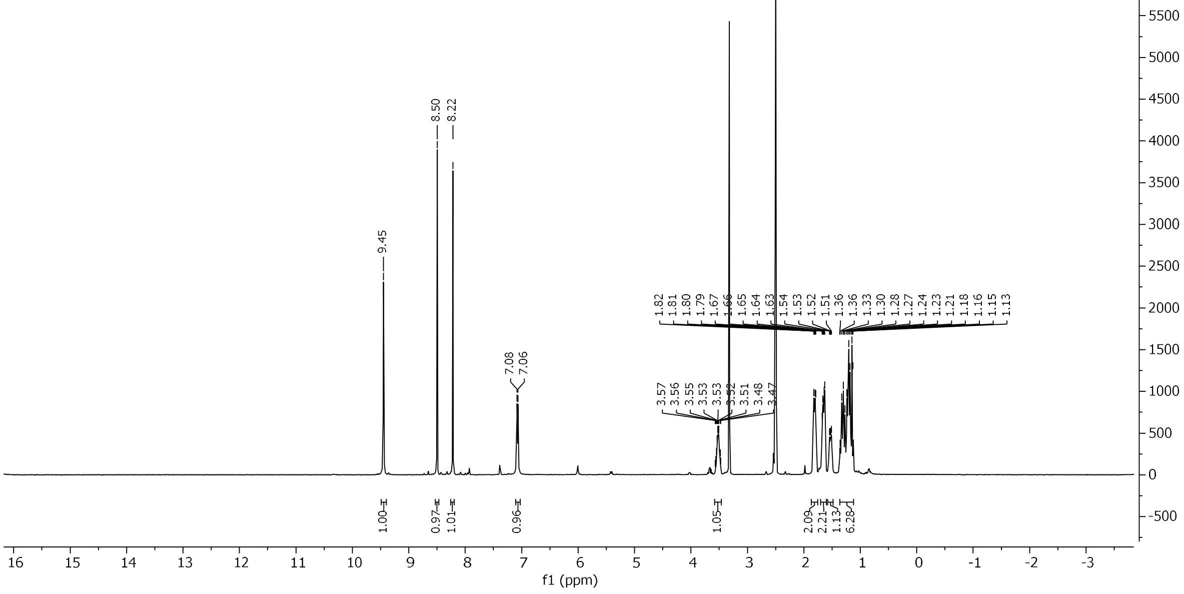

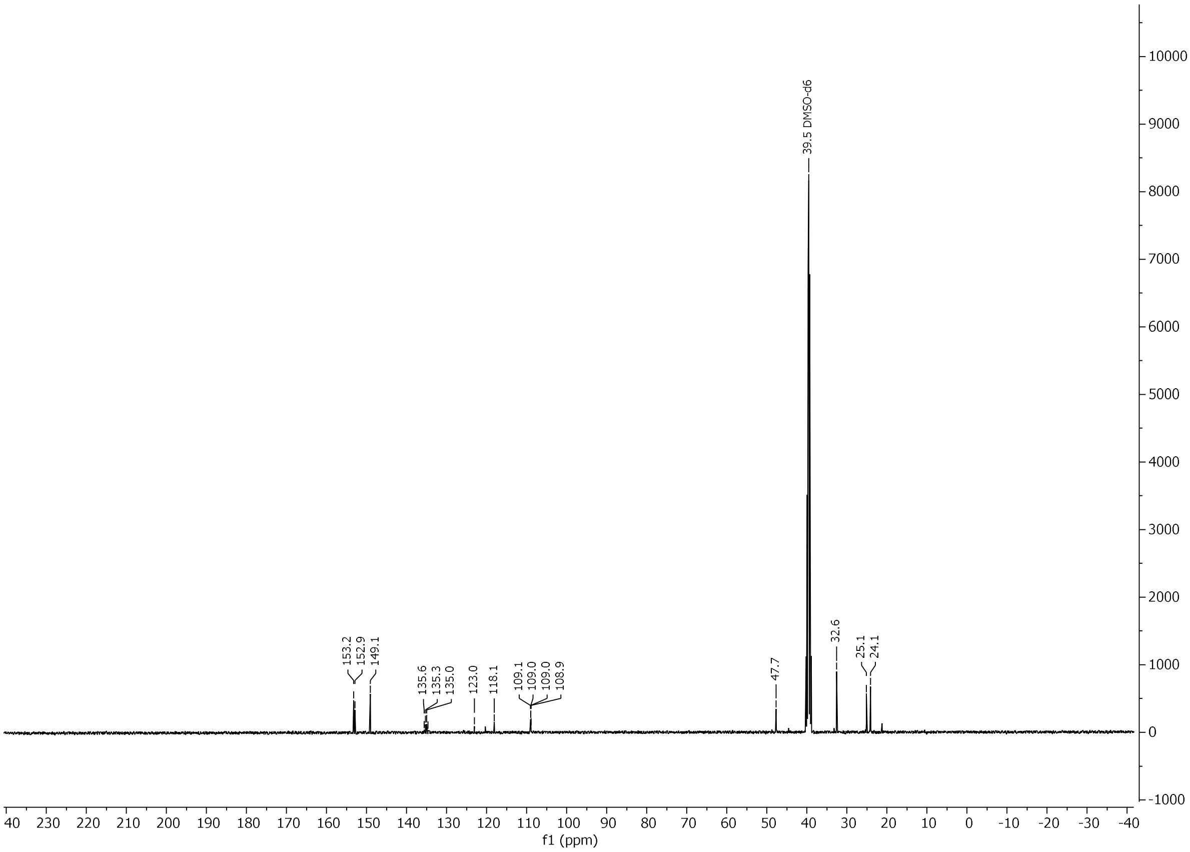

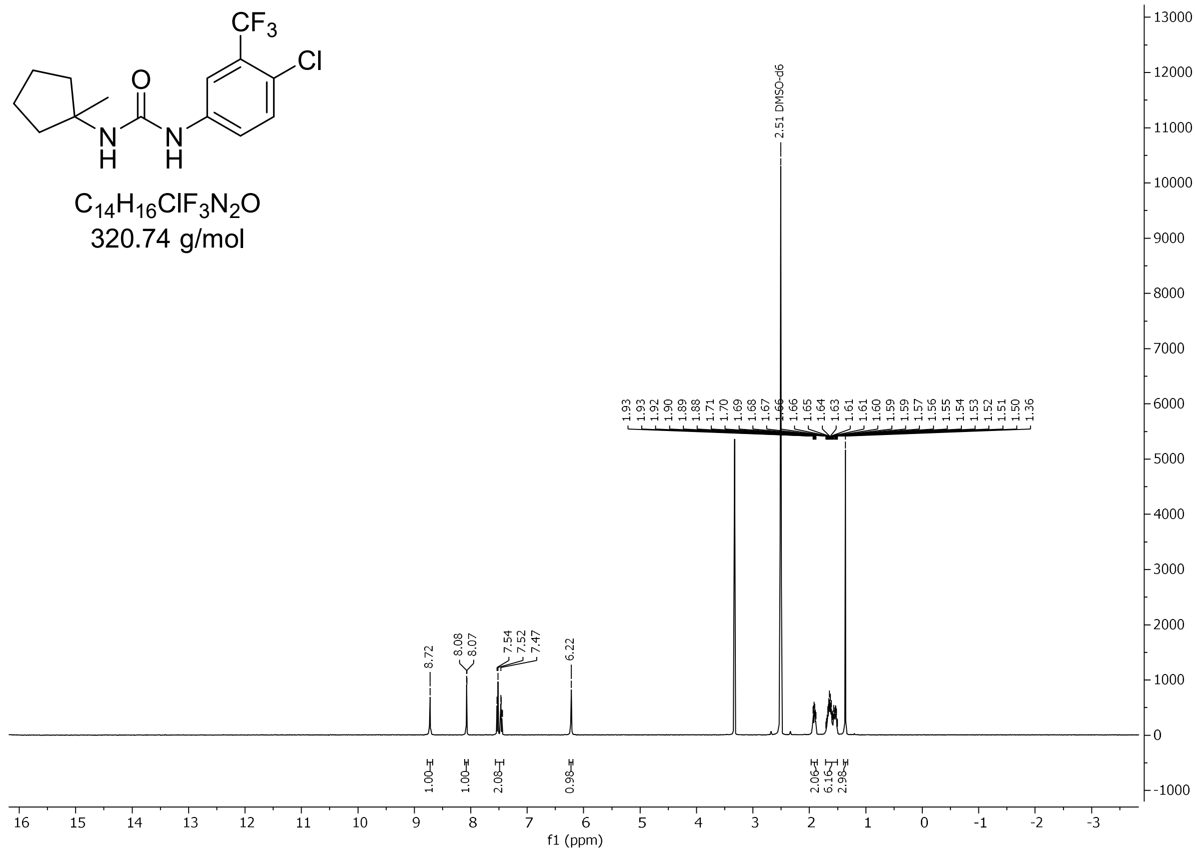
CF3
Cl
O
N
H
N
H
C14H16ClF3N2O
320.74 g/mol
2.51 DMSO-d6
1.93 1.93 1.92 1.90 1.89 1.88 1.71 1.70 1.69 1.68 1.67 1.66 1.66 1.65 1.64 1.63 1.61 1.61 1.60 1.59 1.59 1.57 1.56 1.55 1.54 1.53 1.52 1.51 1.50 1.36
8.72
8.08
8.07
7.54
7.52
7.47
6.22
1.00
1.00
2.08
0.98
2.06
6.16
2.98
f1 (ppm)

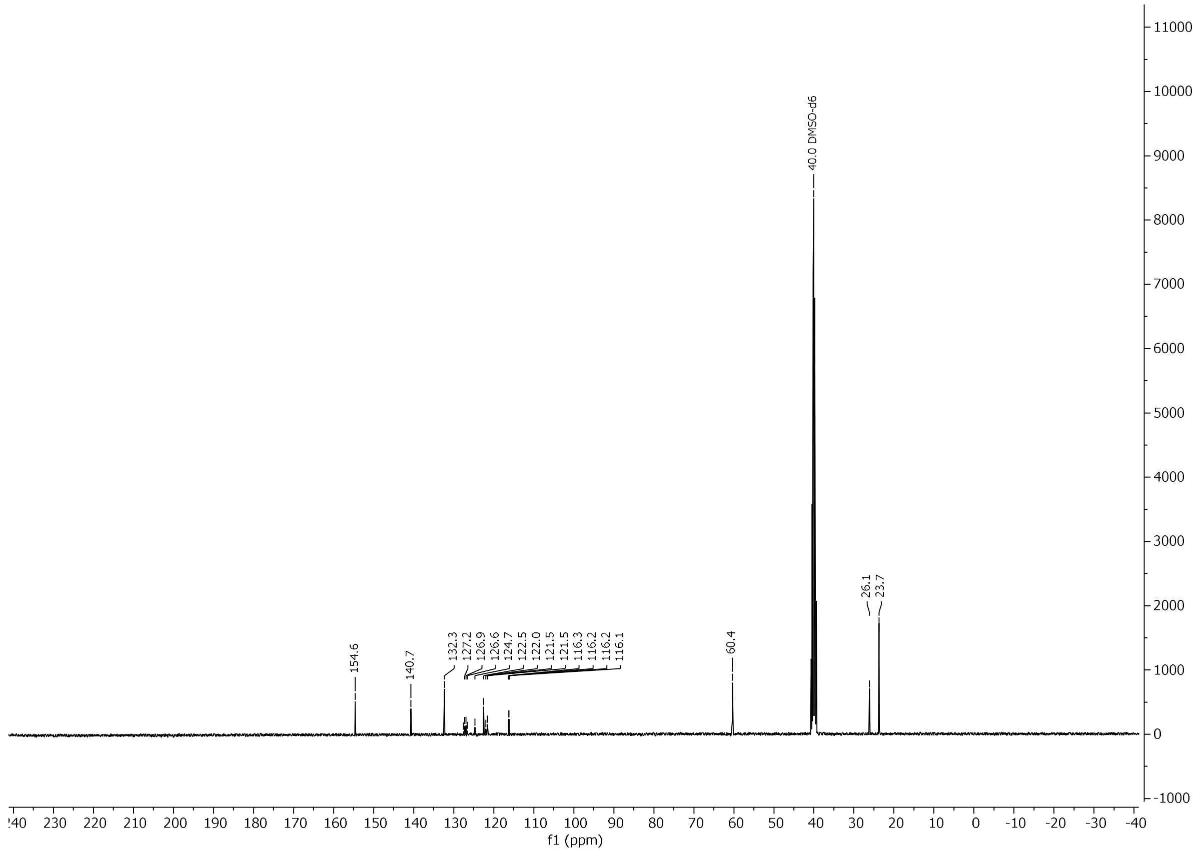
40.0 DMSO-d6
154.6
140.7
132.3 127.2 126.9 126.6 124.7 122.5 122.0 121.5 116.3 116.2 116.1
60.4
26.1
23.7
f1 (ppm)

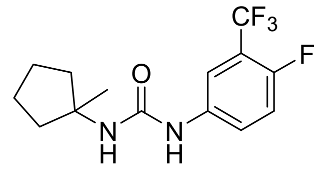

$C_{14}H_{16}F_4N_2O$
304.29 g/mol

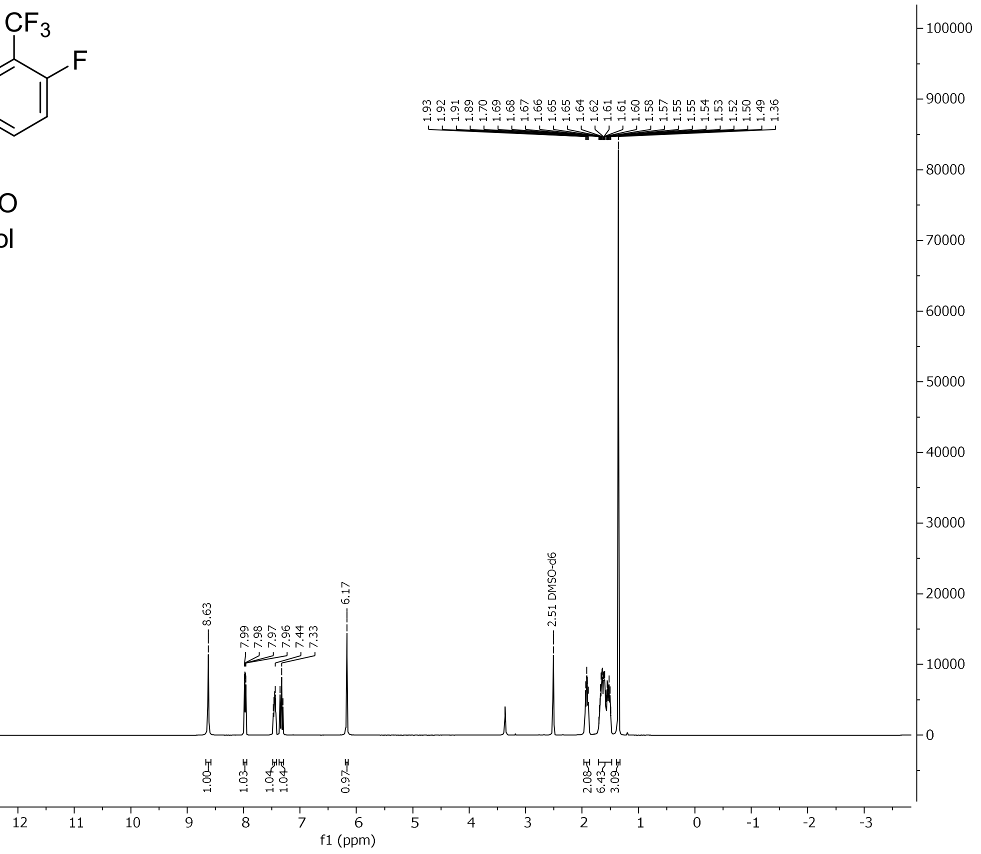

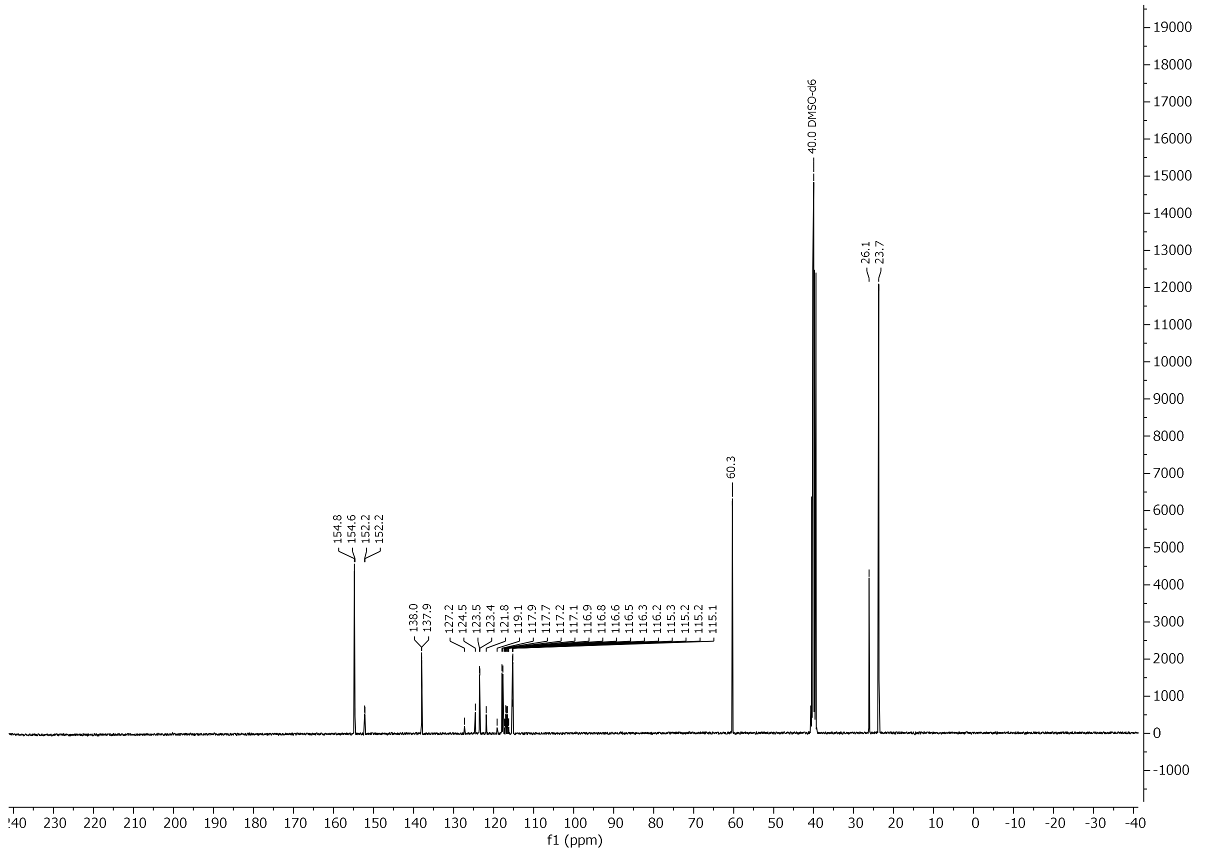

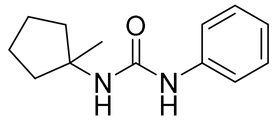

$C_{13}H_{18}N_2O$
218.30 g/mol

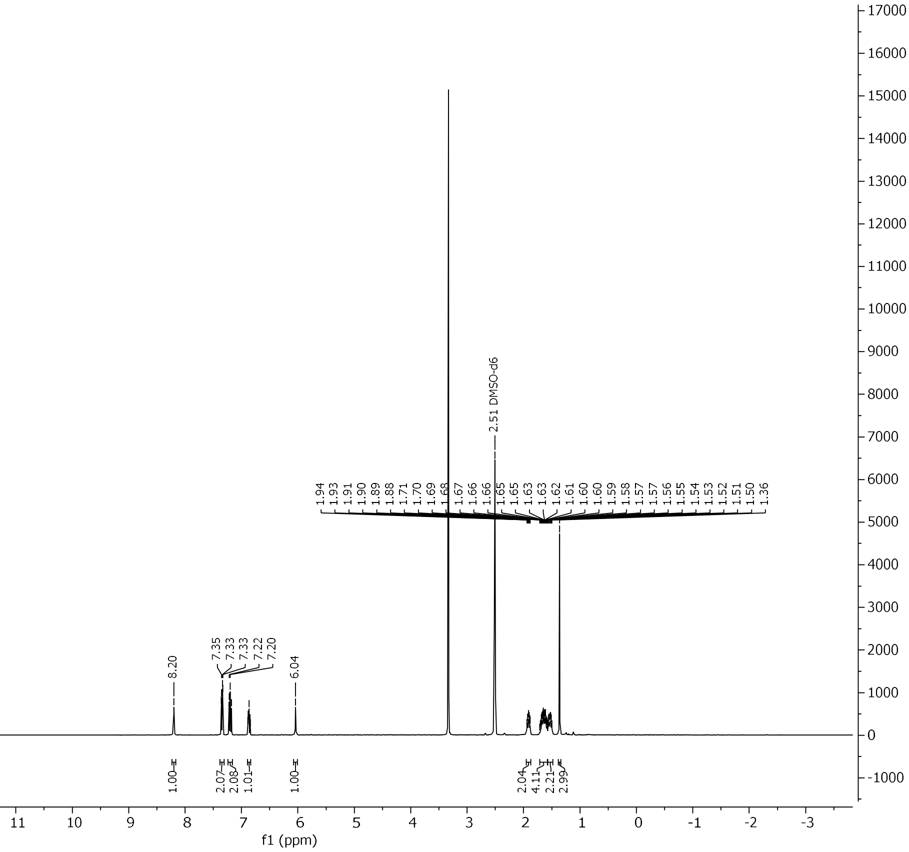

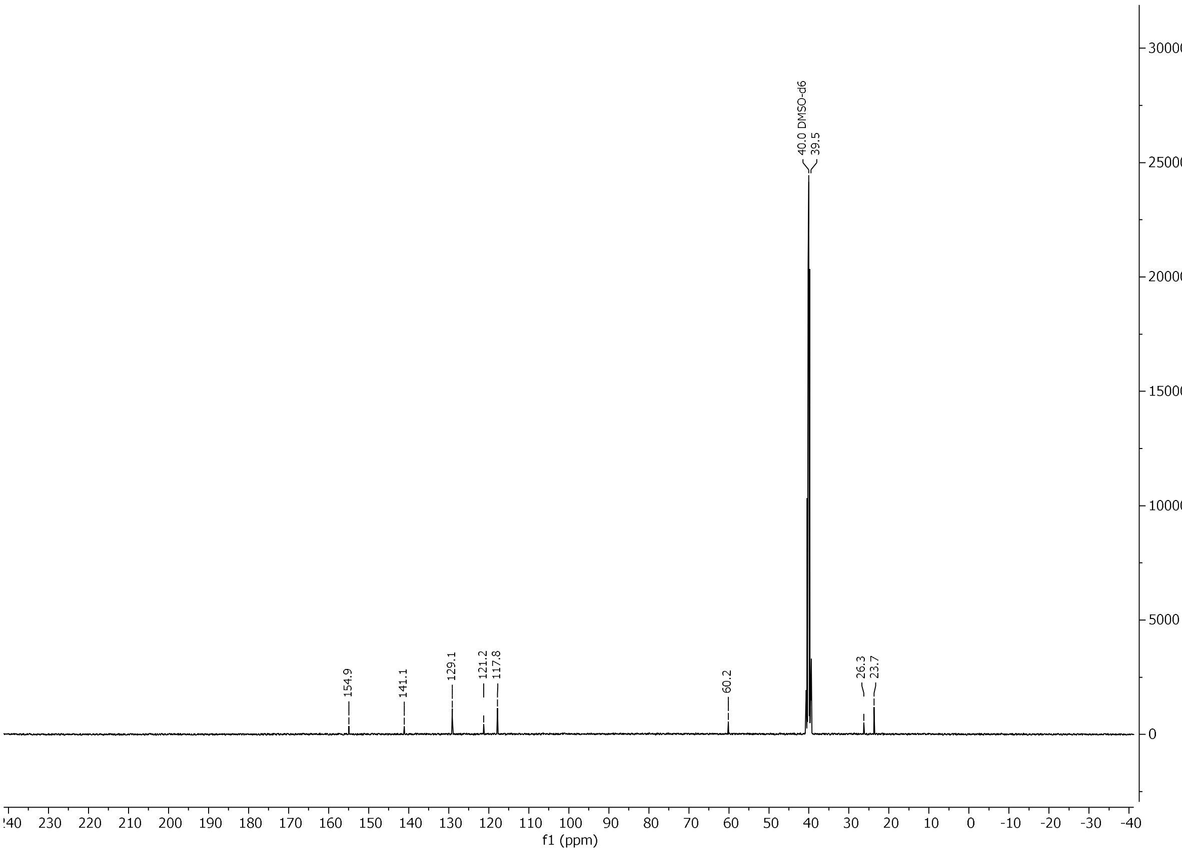

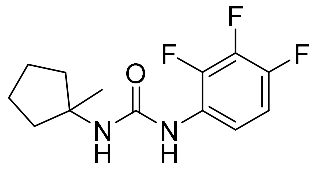

$C_{13}H_{15}F_3N_2O$
272.27 g/mol

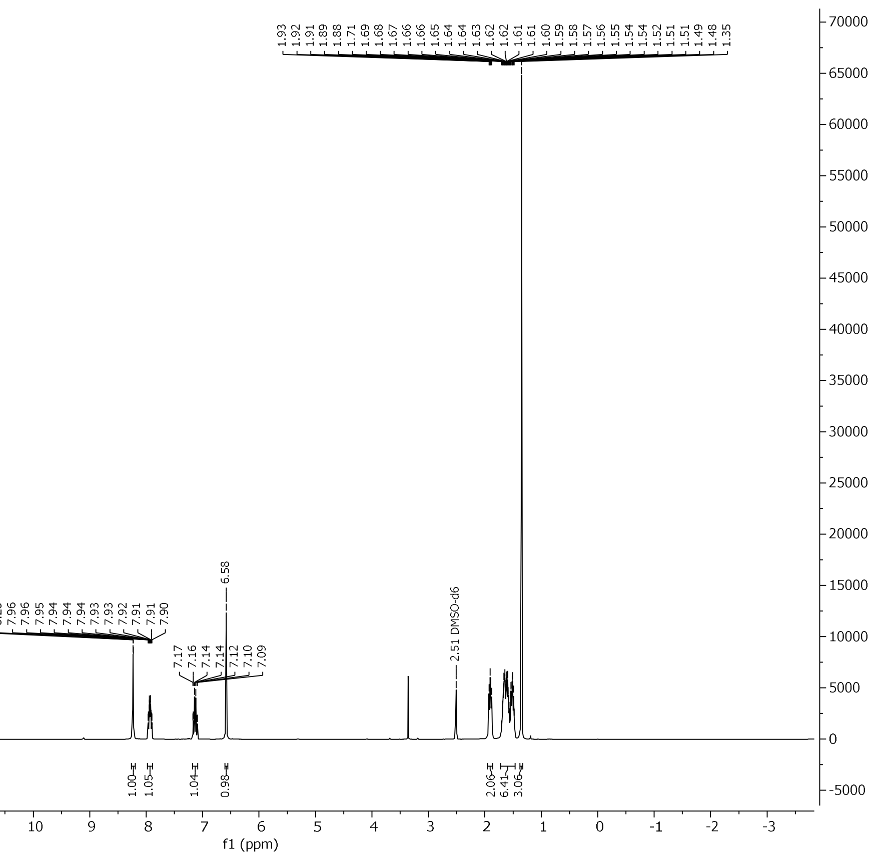

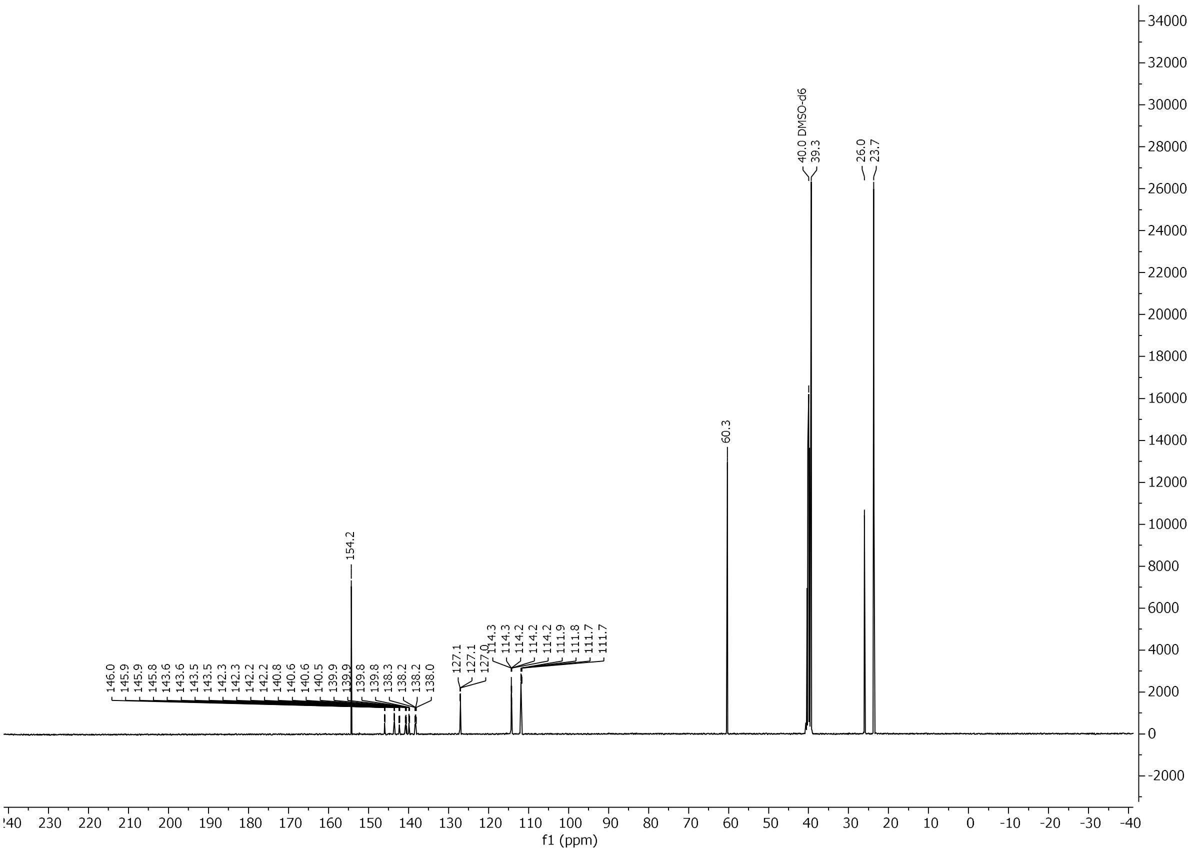

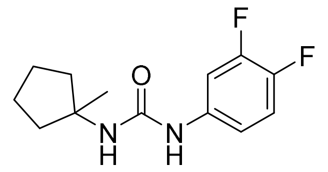

$C_{13}H_{16}F_2N_2O$
254.28 g/mol

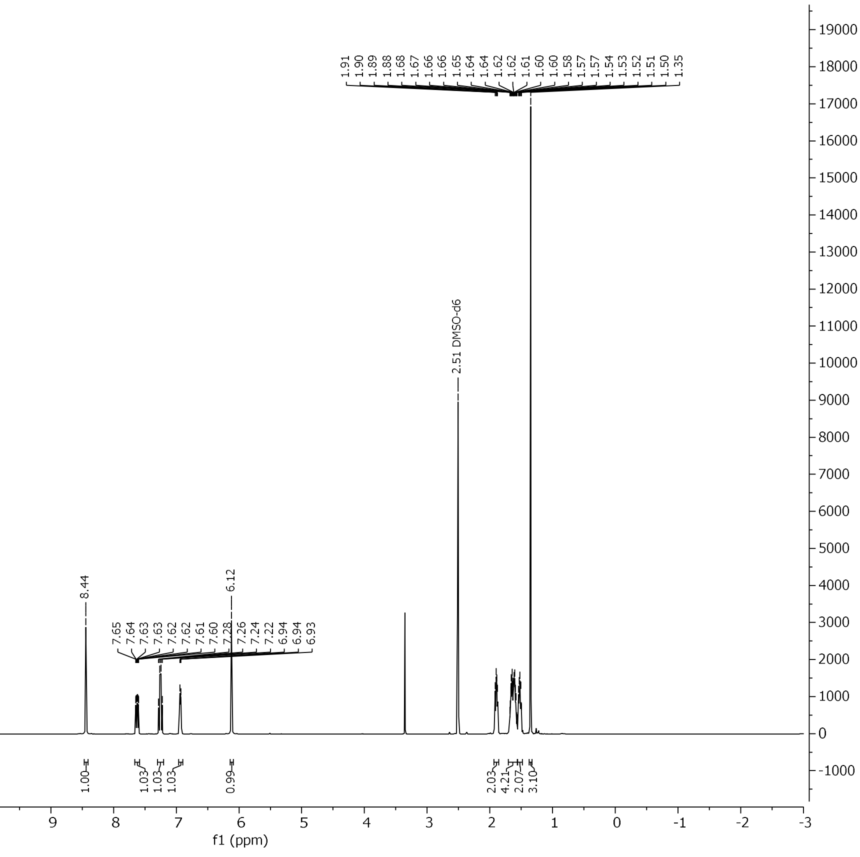

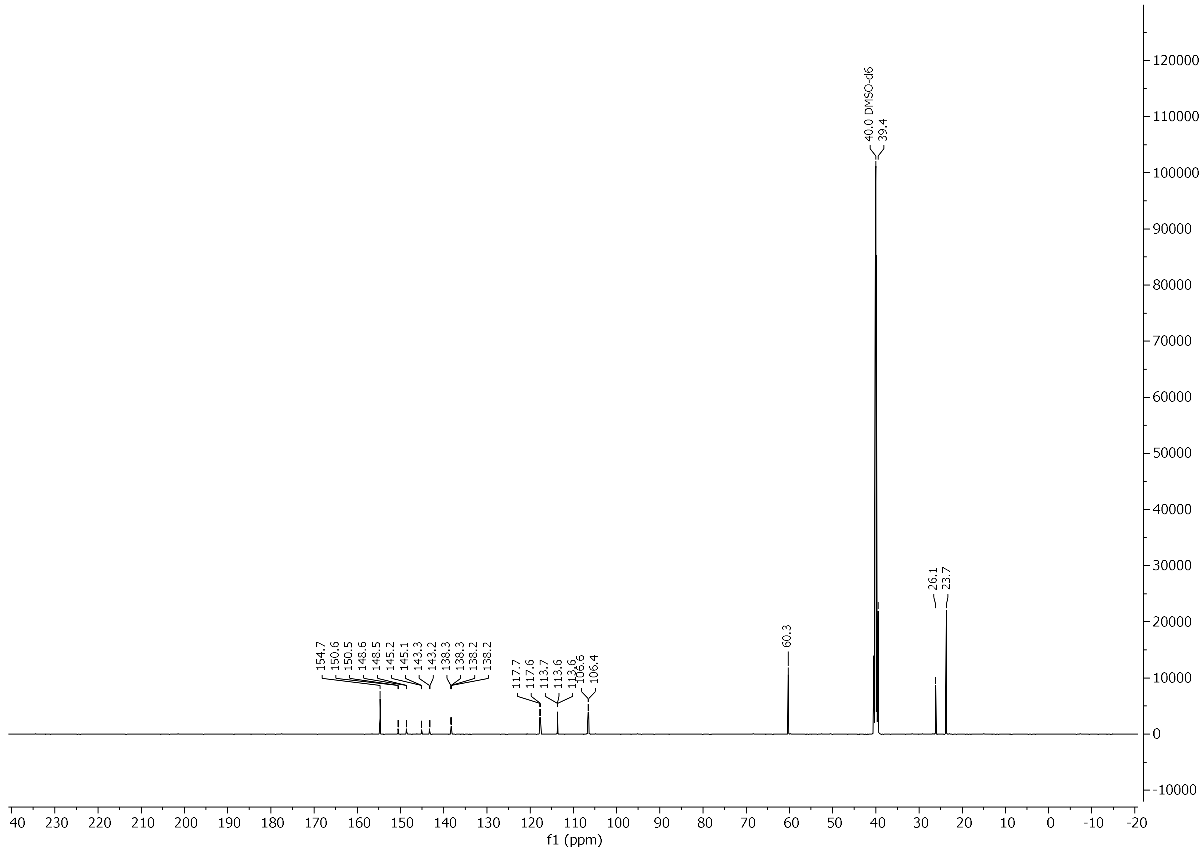

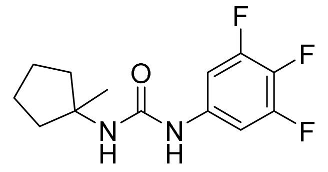

$C_{13}H_{15}F_3N_2O$
272.27 g/mol

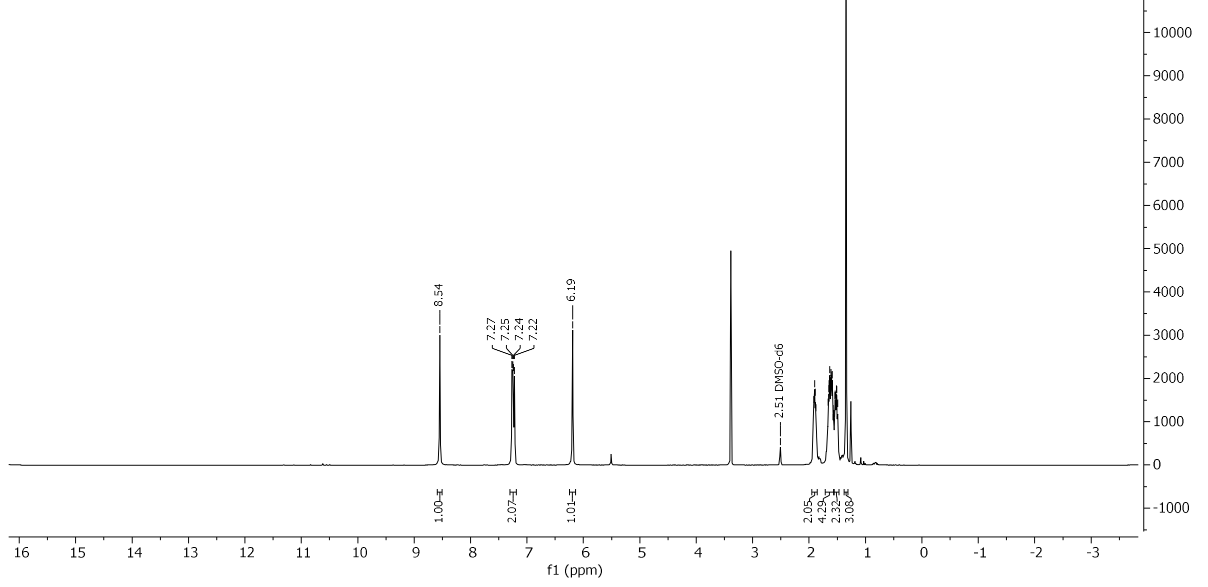

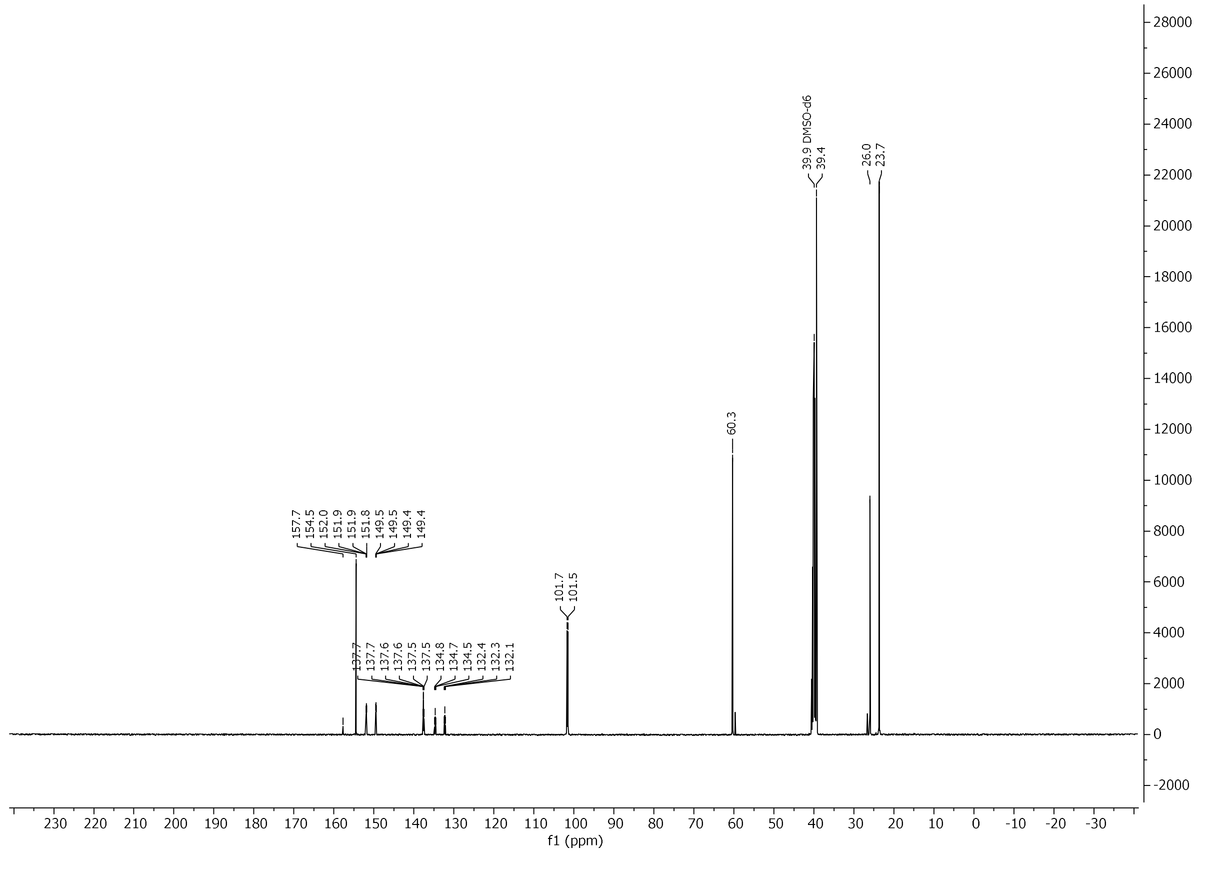

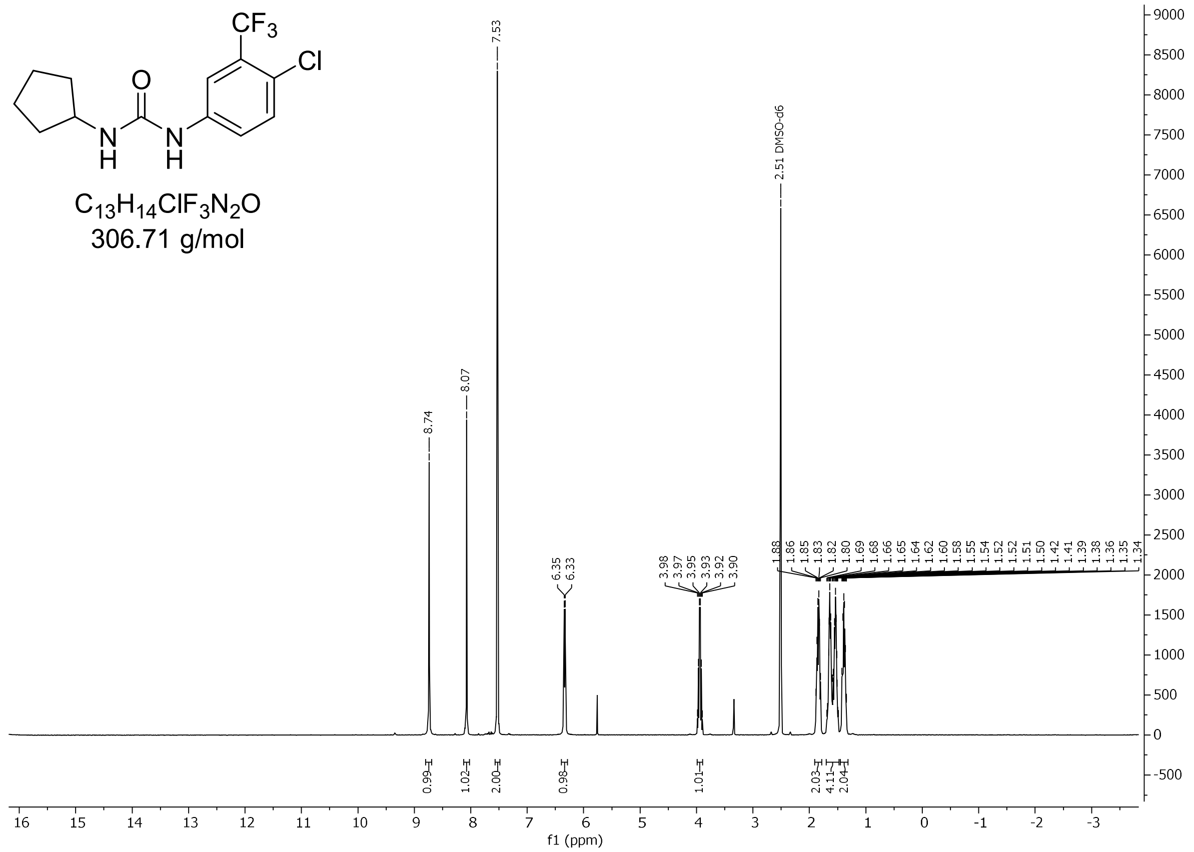
CF3
Cl
O
N
H
N
H
C13H14ClF3N2O
306.71 g/mol
7.53
2.51 DMSO-d6
8.07
8.74
6.35
6.33
3.98
3.97
3.95
3.93
3.92
3.90
0.99
1.02
2.00
0.98
1.01
2.03
4.11
2.04
f1 (ppm)

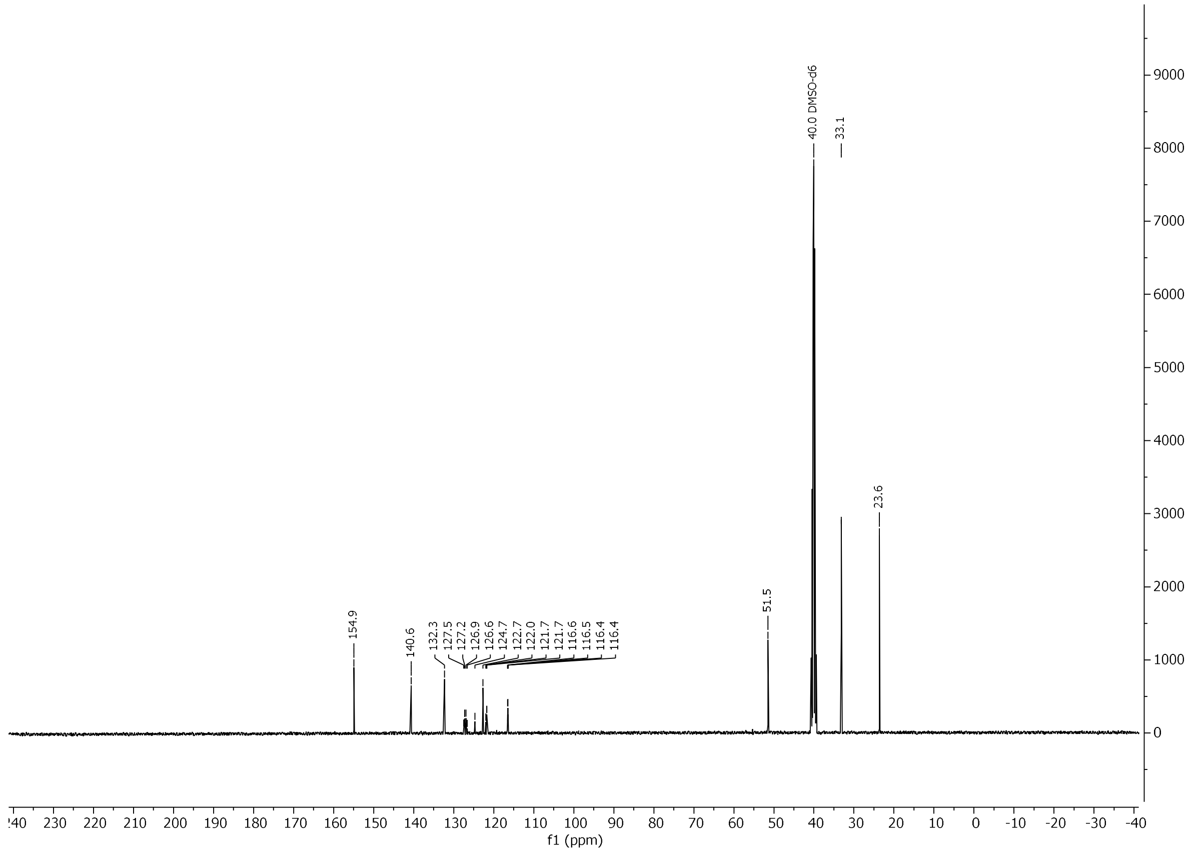
40.0 DMSO-d6
33.1
23.6
51.5
154.9
140.6
132.3
127.5
127.2
126.9
126.6
124.7
122.7
122.0
121.7
121.7
116.6
116.5
116.4
116.4
f1 (ppm)

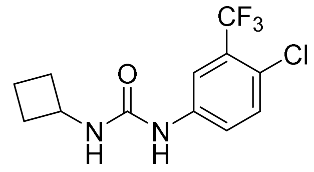

$C_{12}H_{12}ClF_3N_2O$
292.69 g/mol

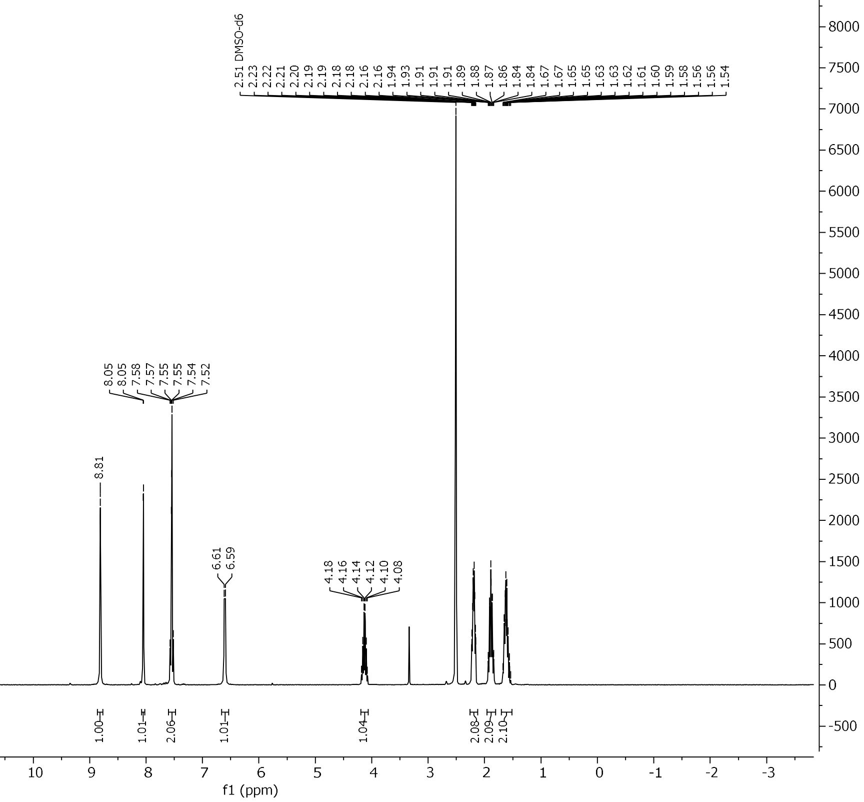

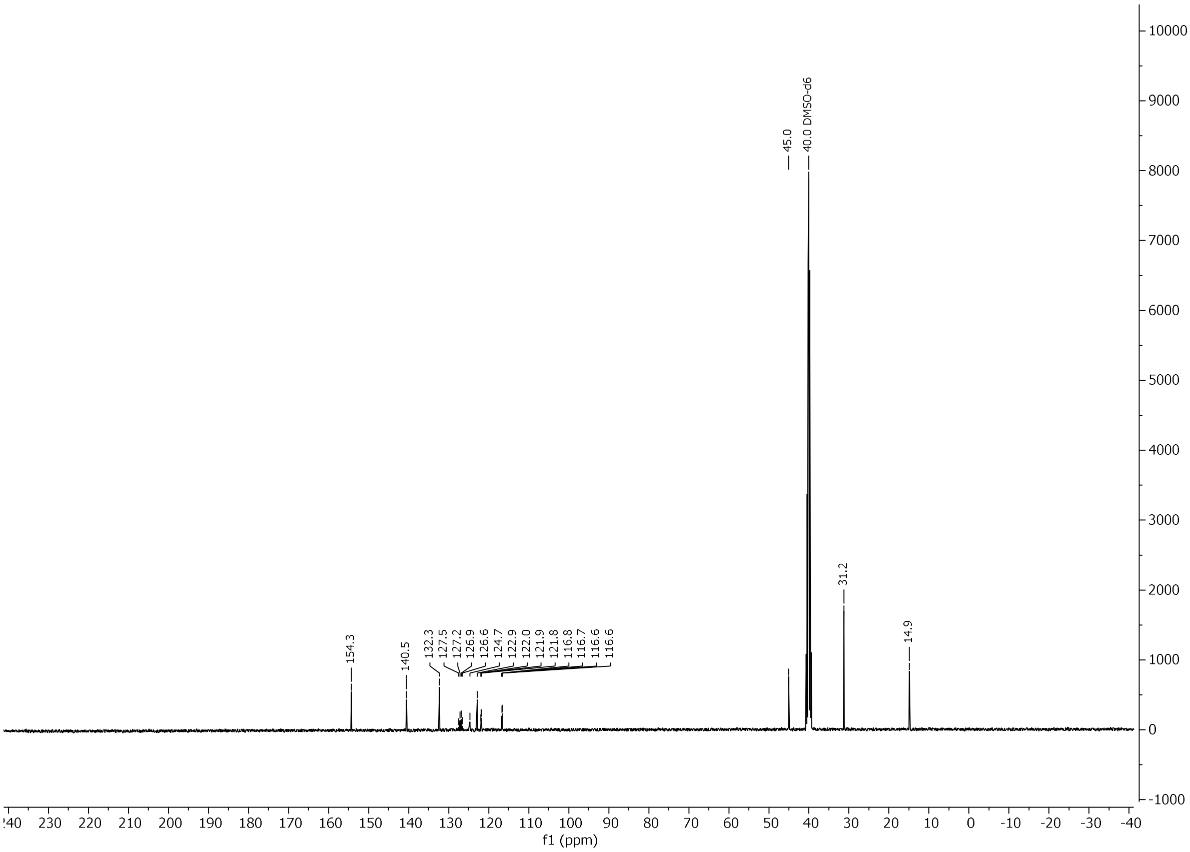

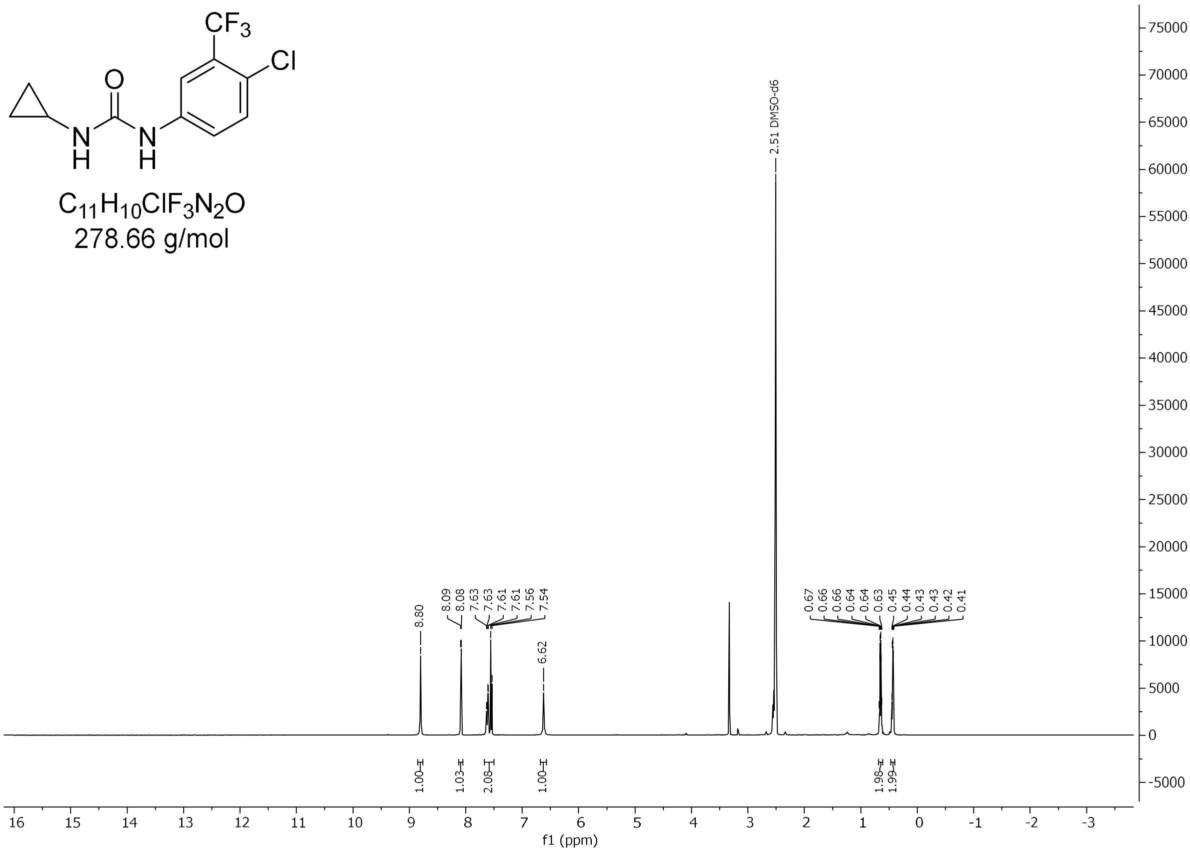

$C_{11}H_{10}ClF_3N_2O$
278.66 g/mol

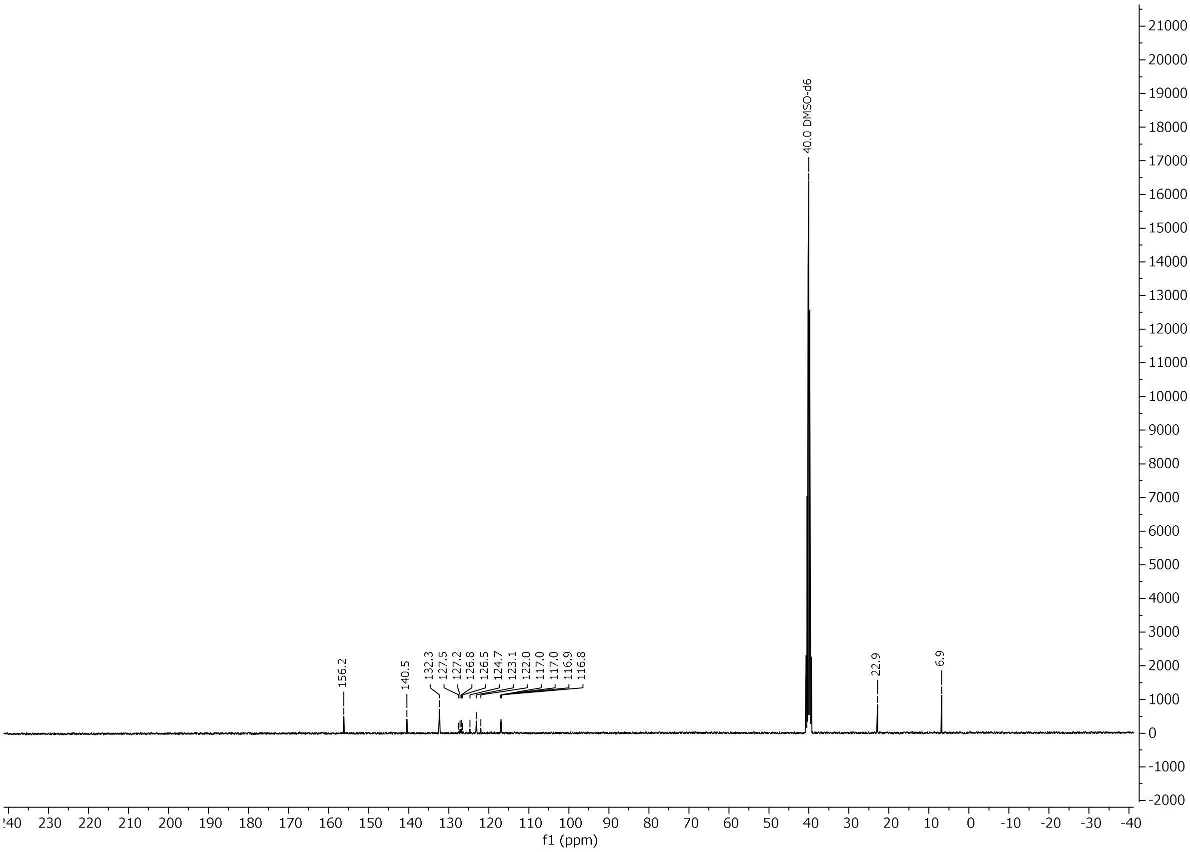

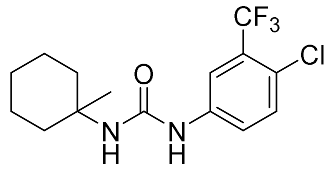

$C_{15}H_{18}ClF_3N_2O$

334.77 g/mol

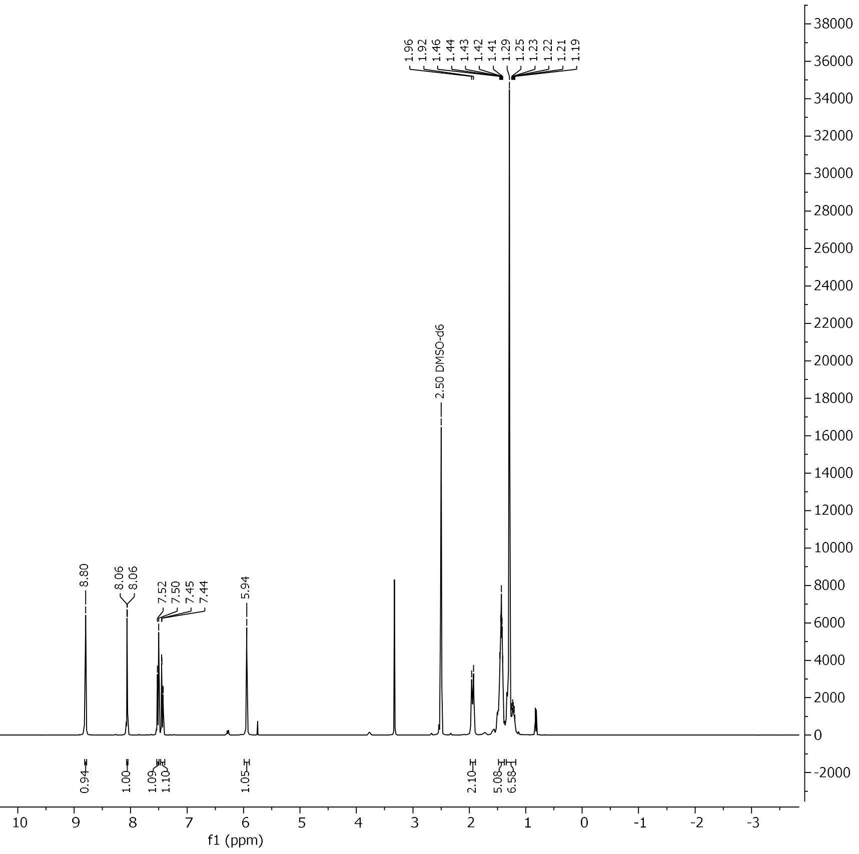

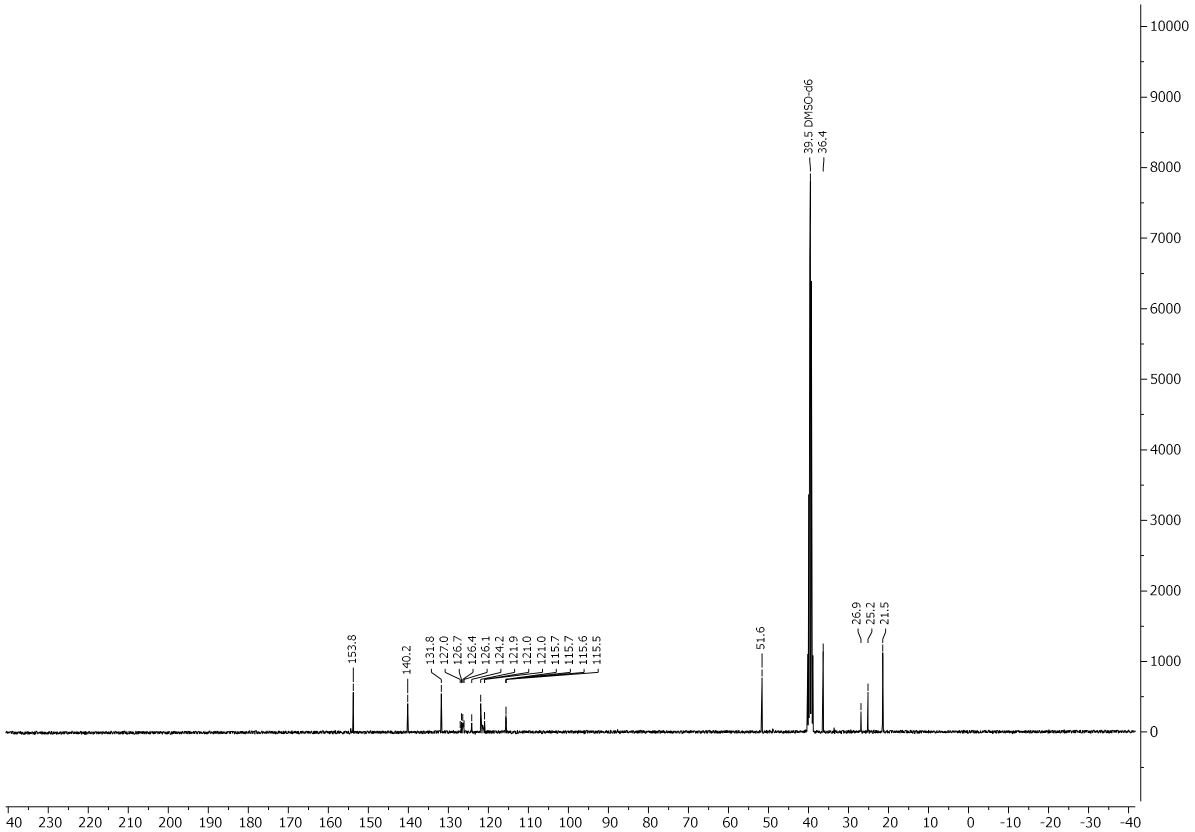

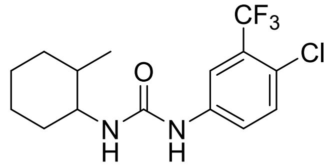

$C_{15}H_{18}ClF_3N_2O$
334.77 g/mol

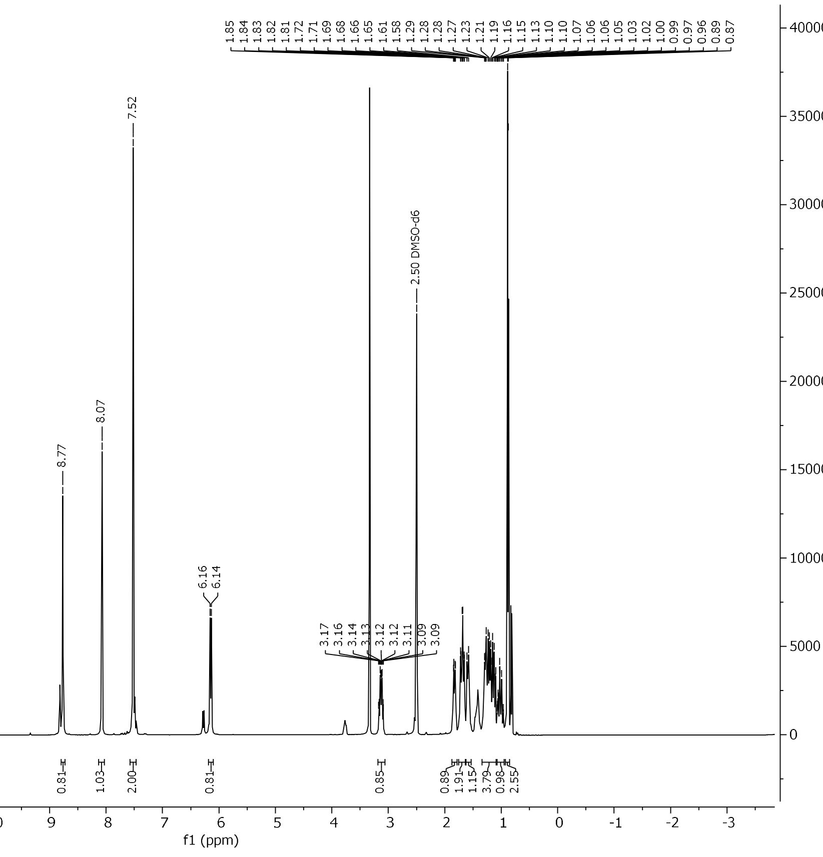

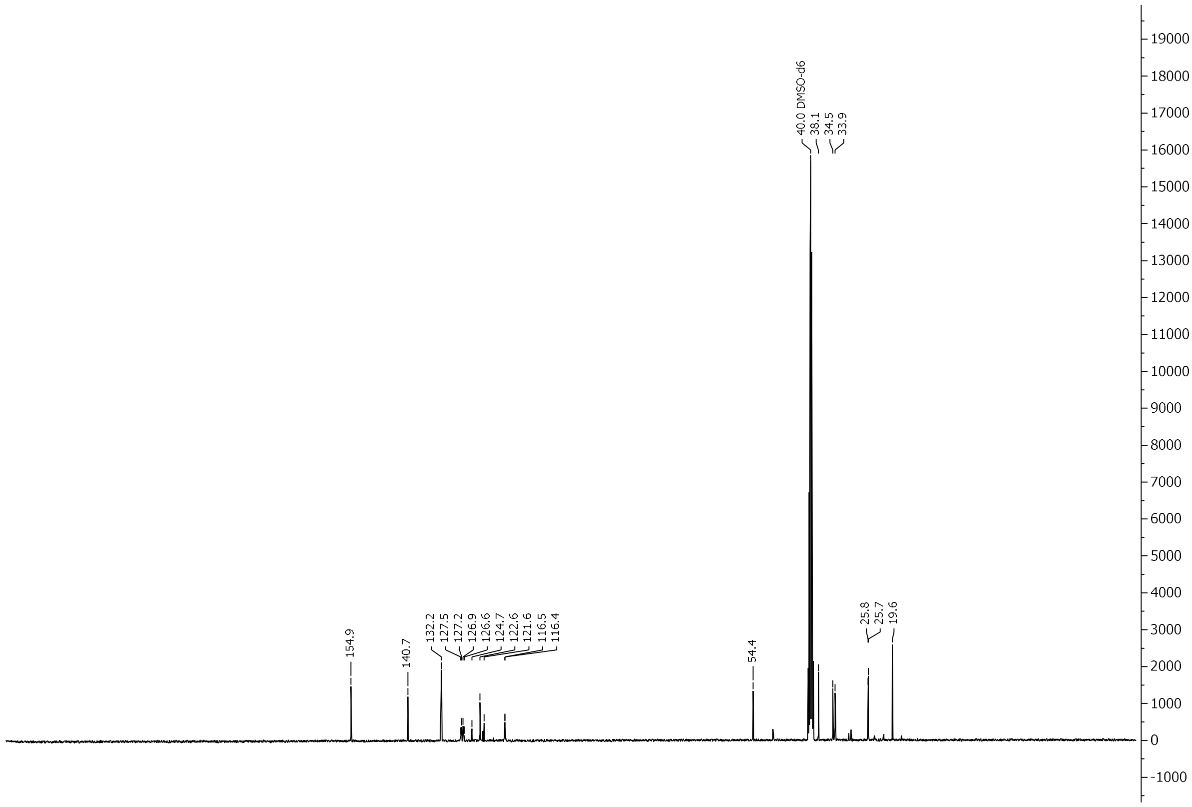

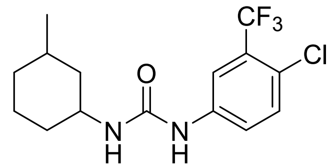

$C_{15}H_{18}ClF_3N_2O$
334.77 g/mol

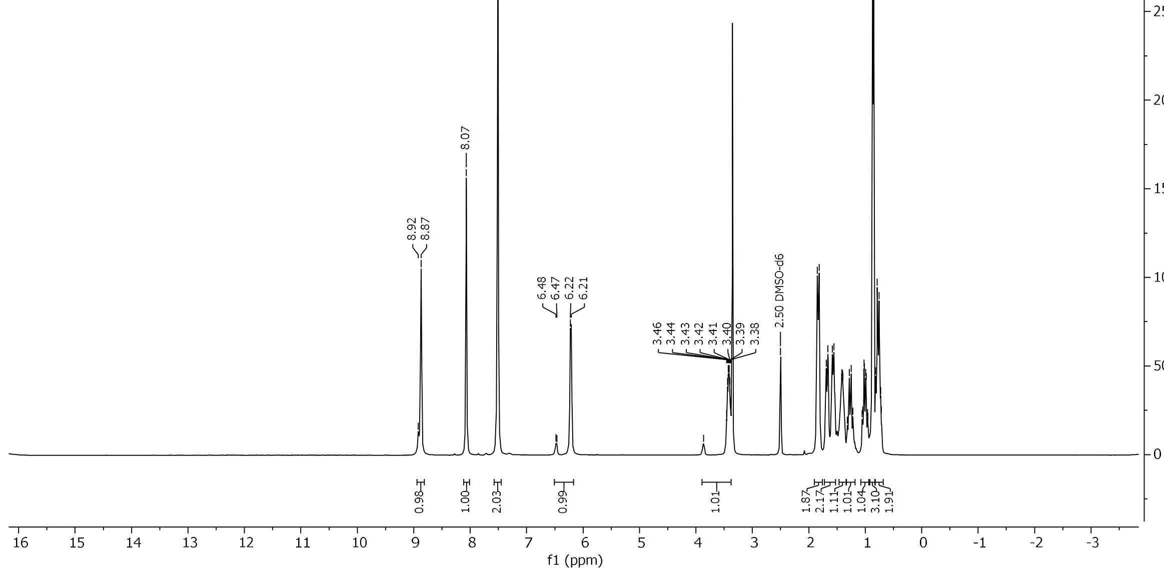

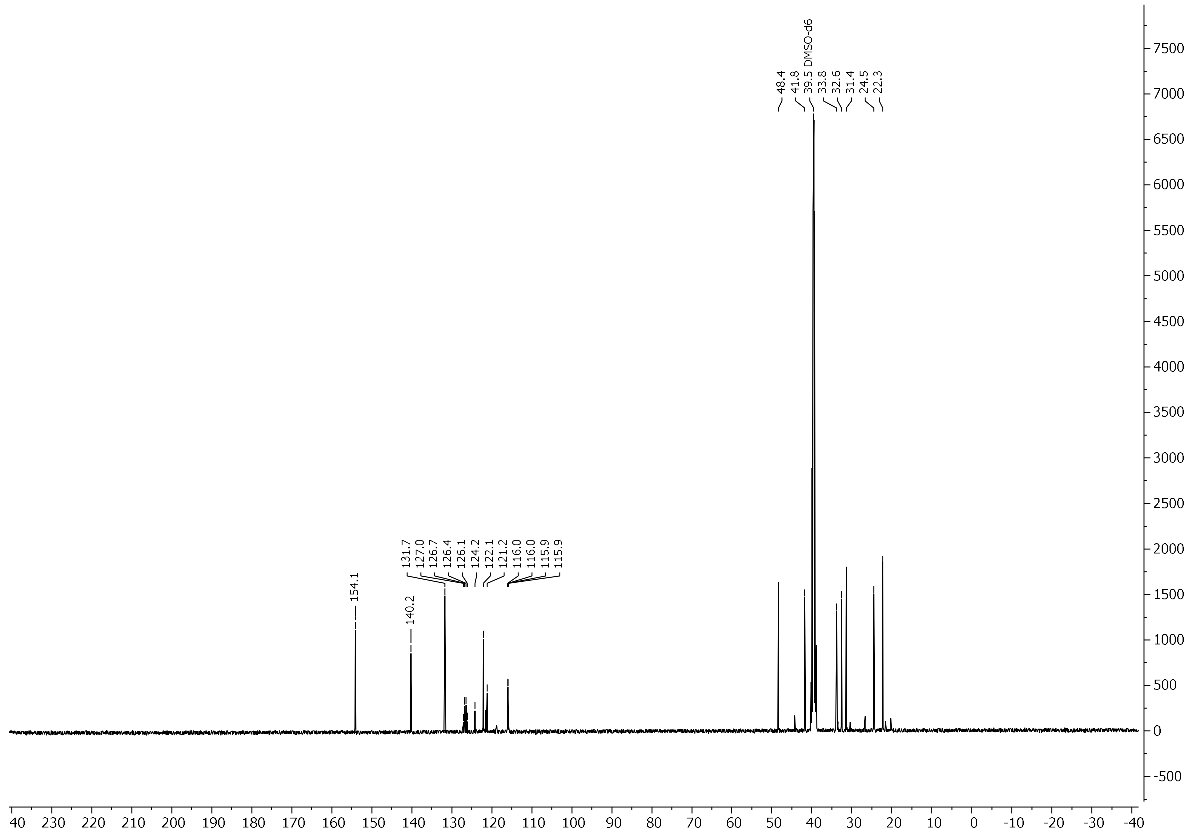

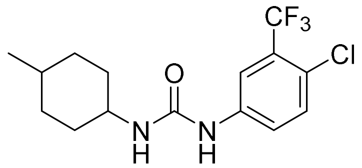

$C_{15}H_{18}ClF_3N_2O$
334.77 g/mol

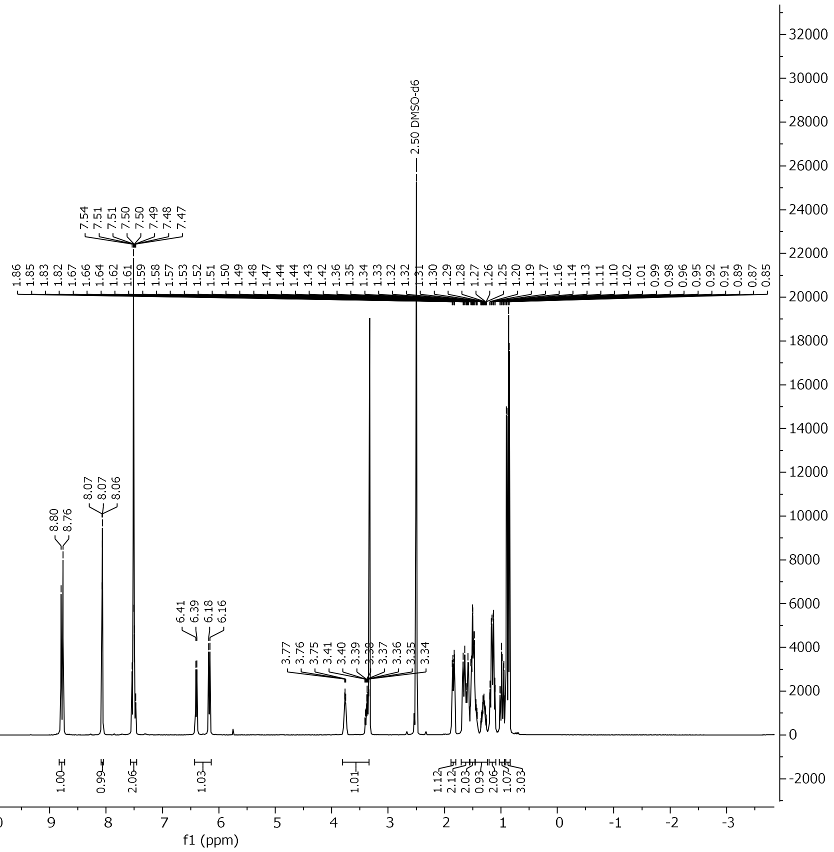

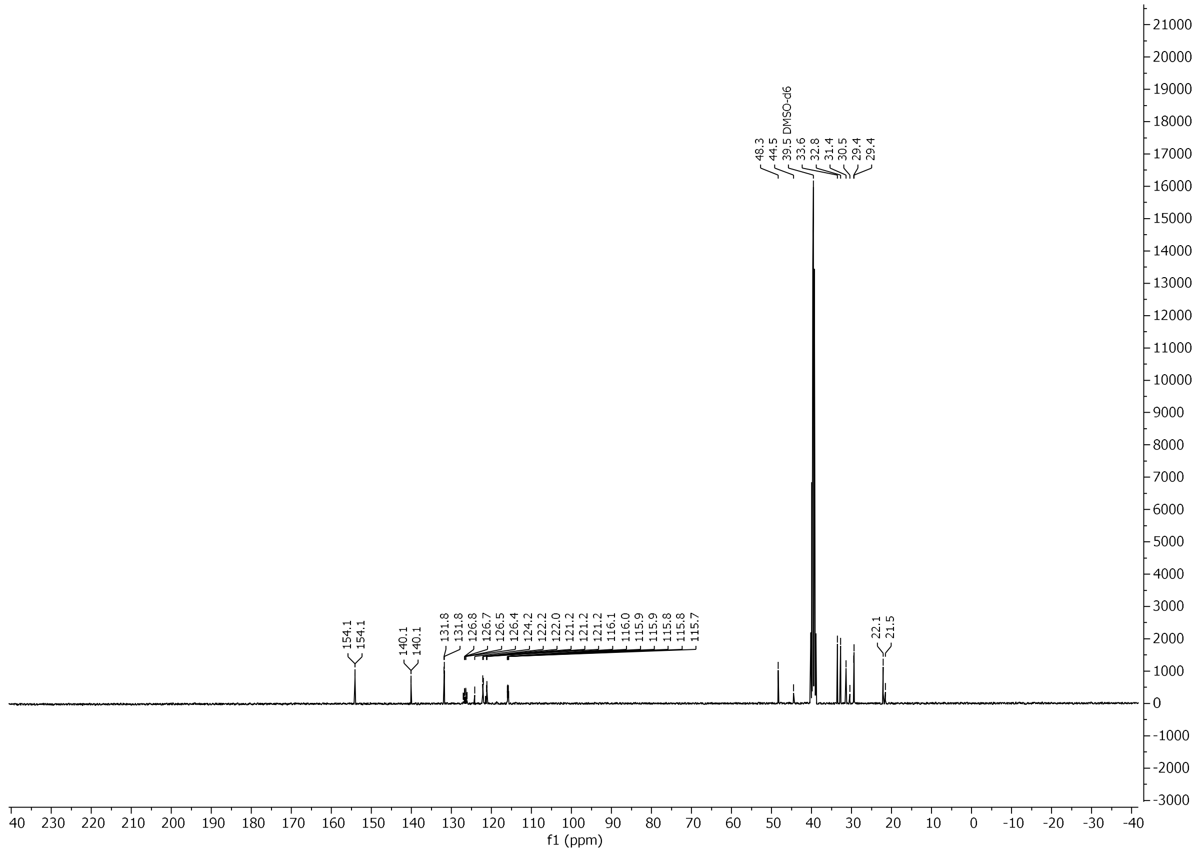

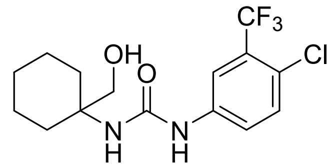

$C_{15}H_{18}ClF_3N_2O_2$
350.77 g/mol

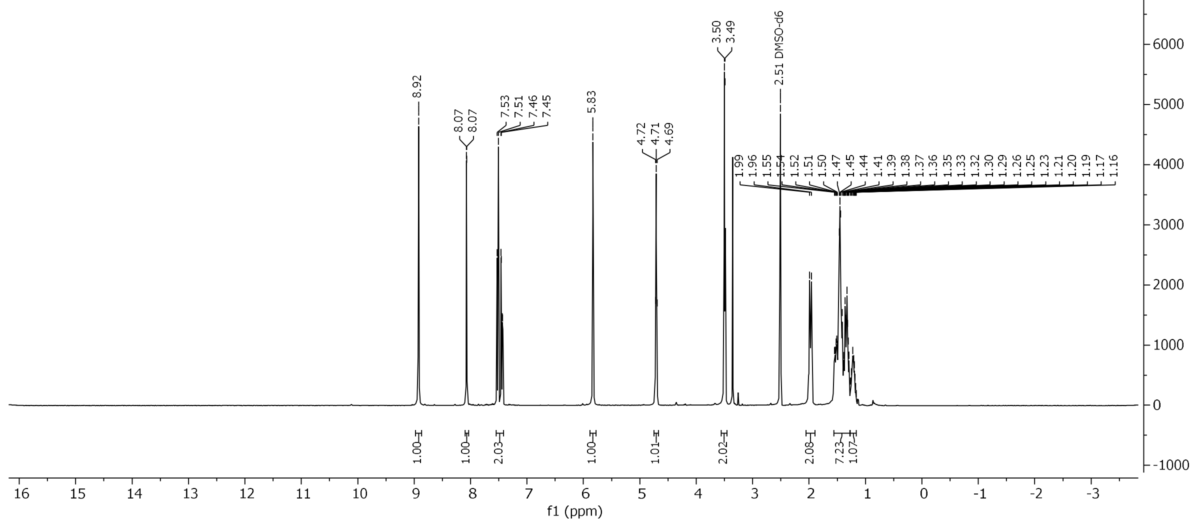

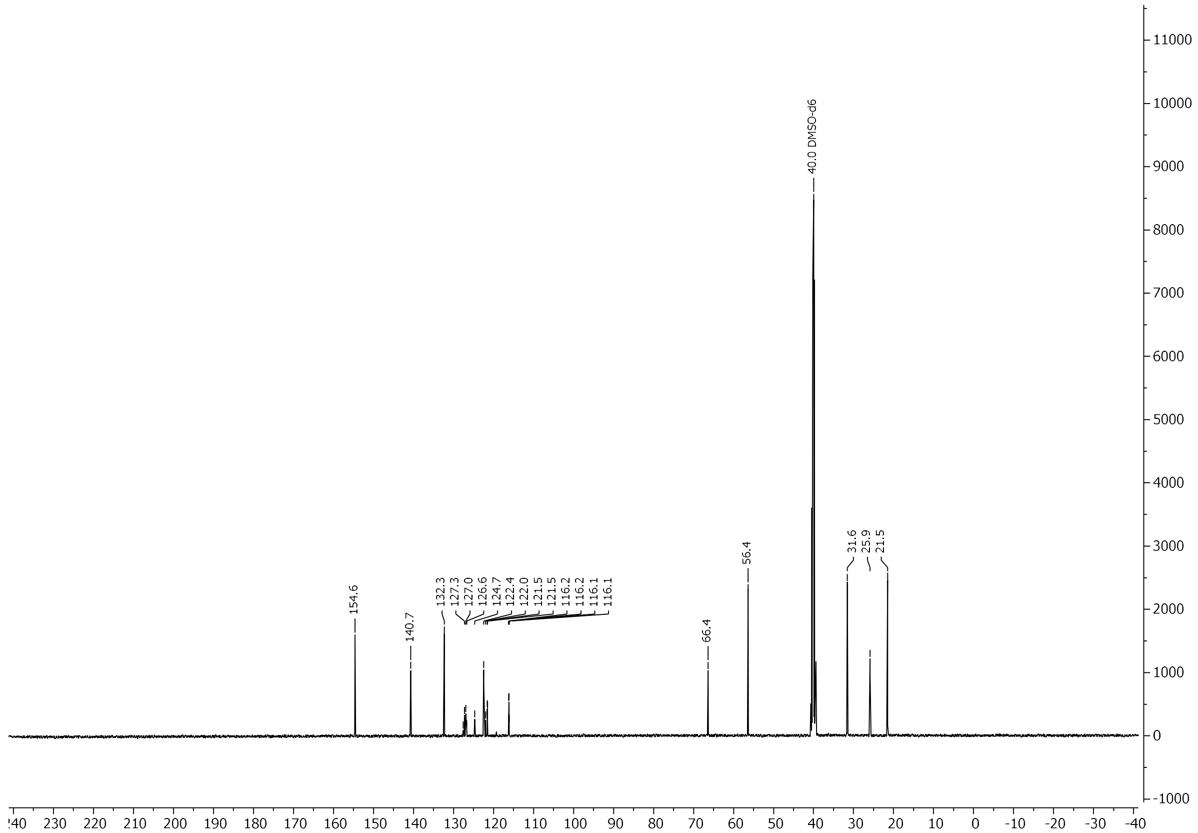

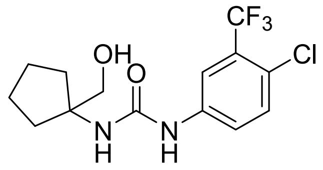

$C_{14}H_{16}ClF_3N_2O_2$
336.74 g/mol

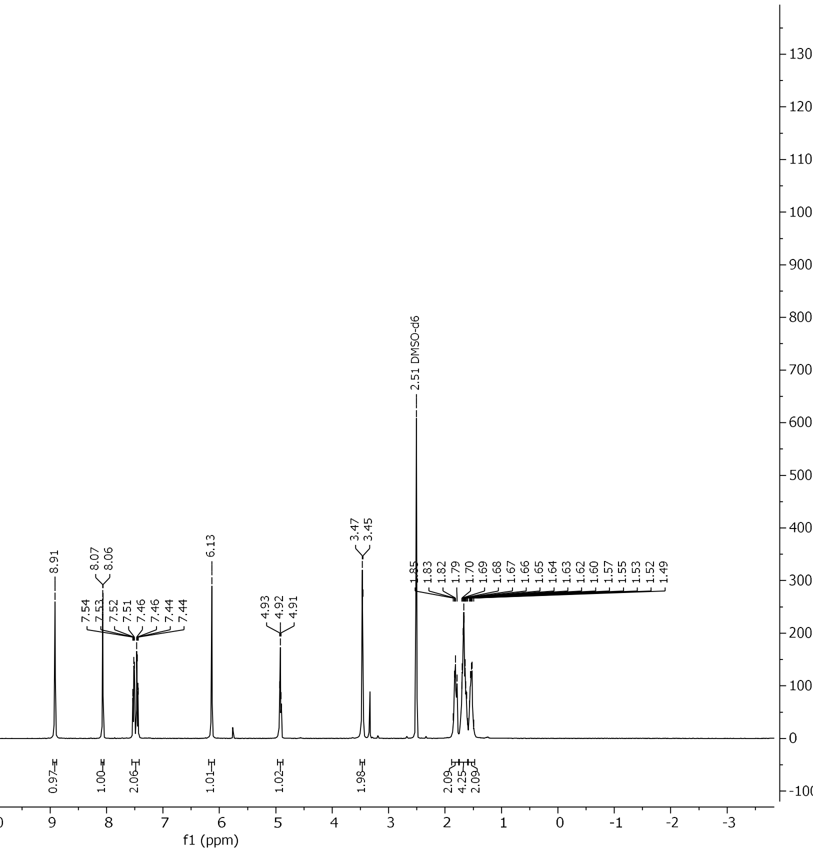

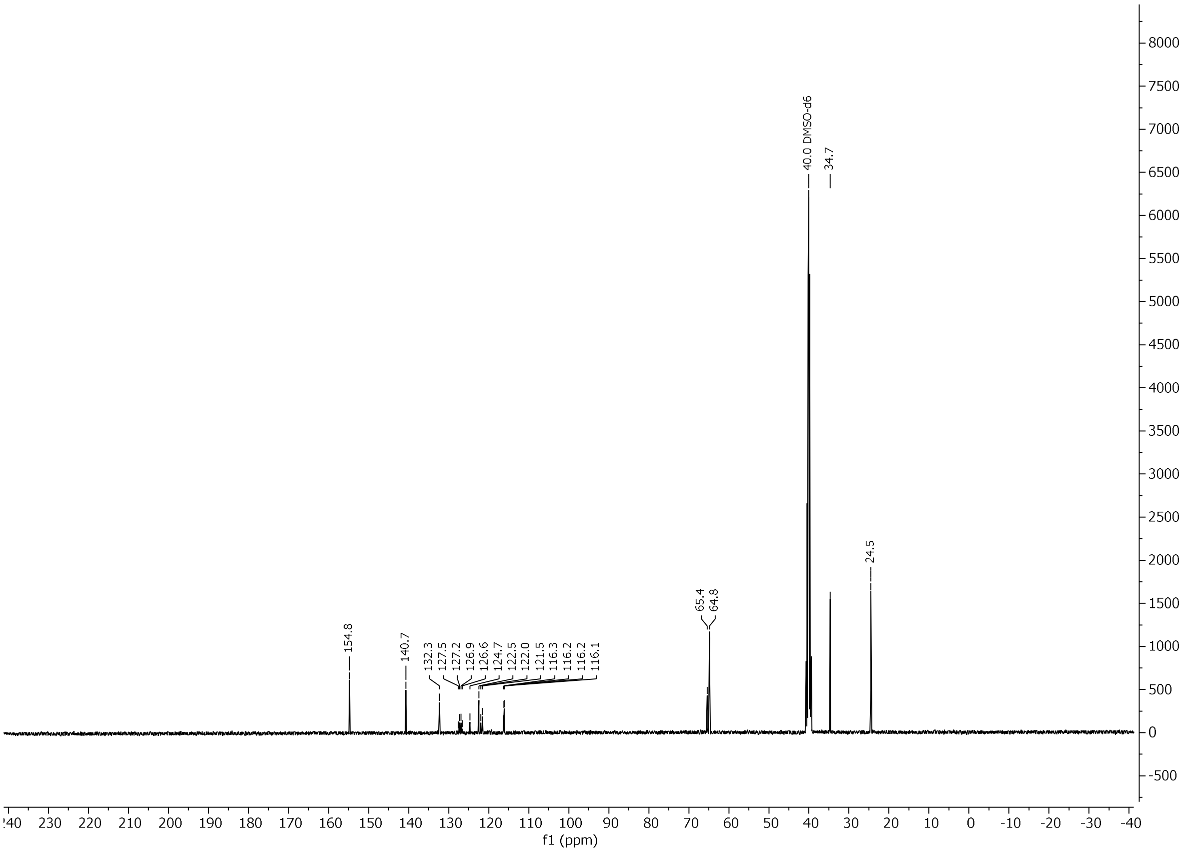

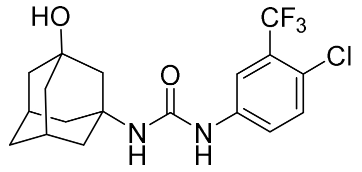

$C_{18}H_{20}ClF_3N_2O_2$
388.81 g/mol

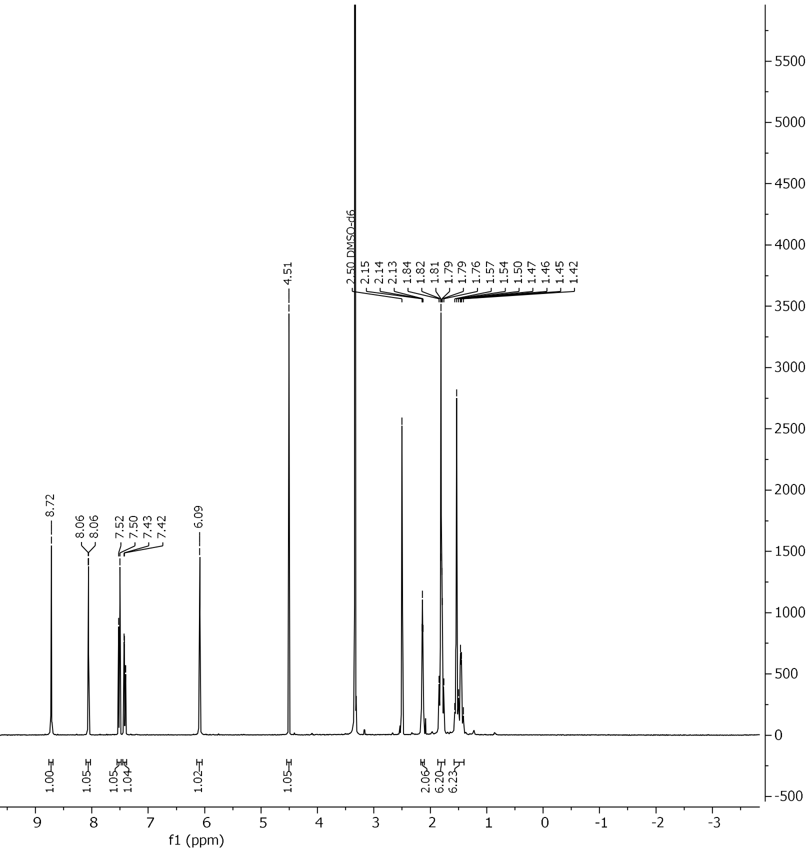

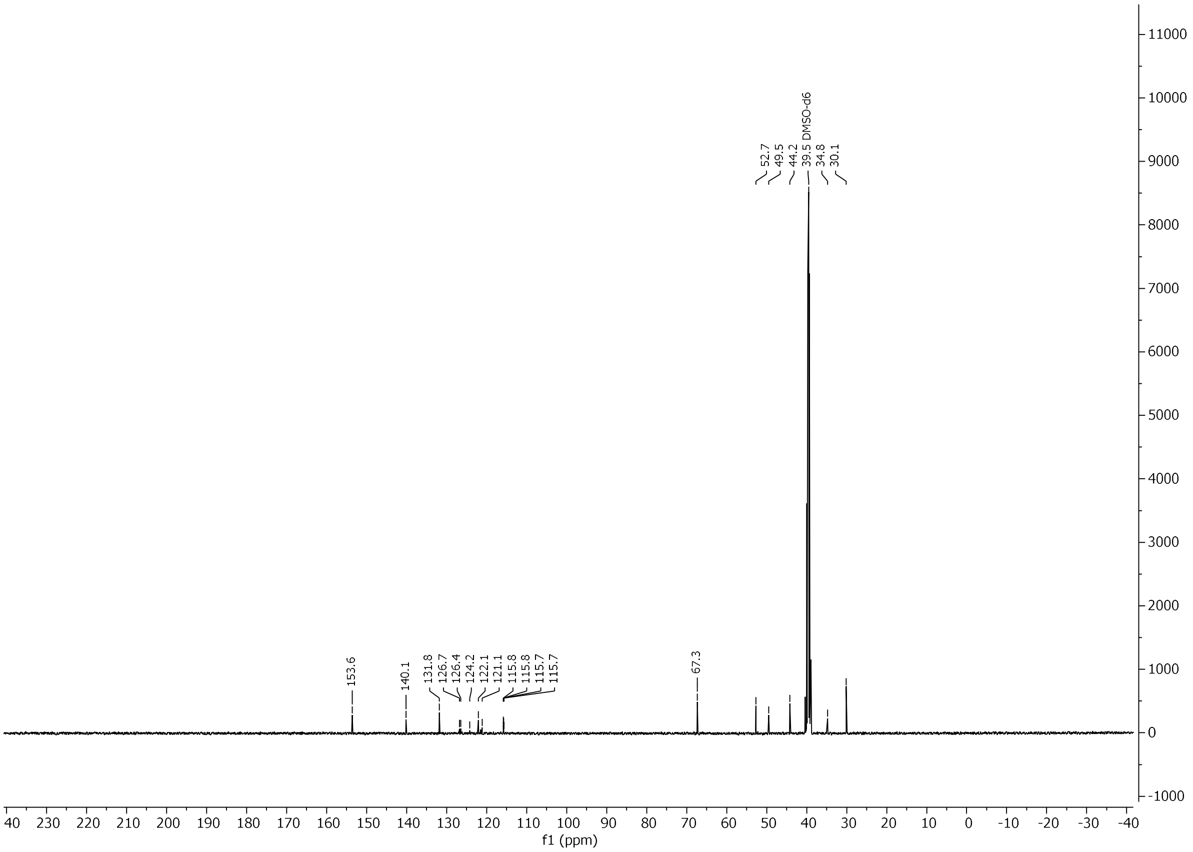

$C_{13}H_{12}ClF_3N_2O$
304.70 g/mol

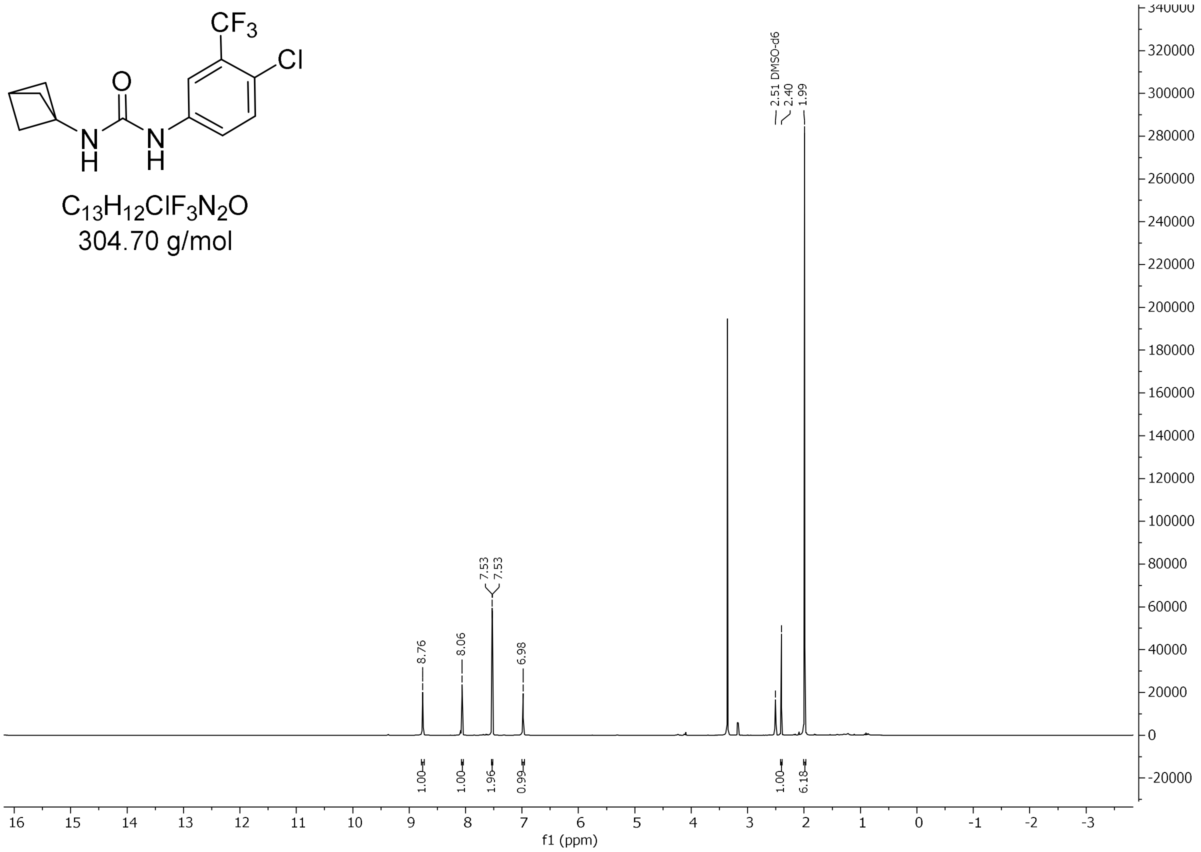

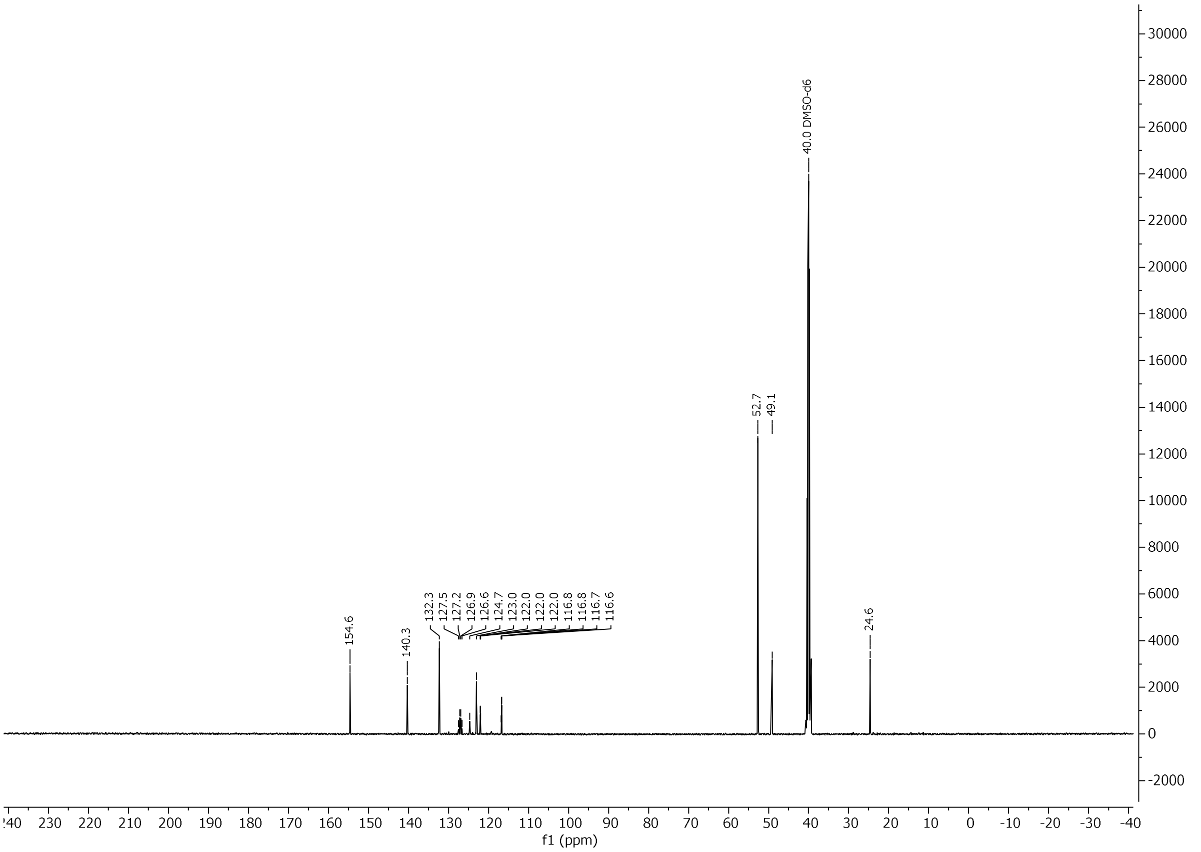

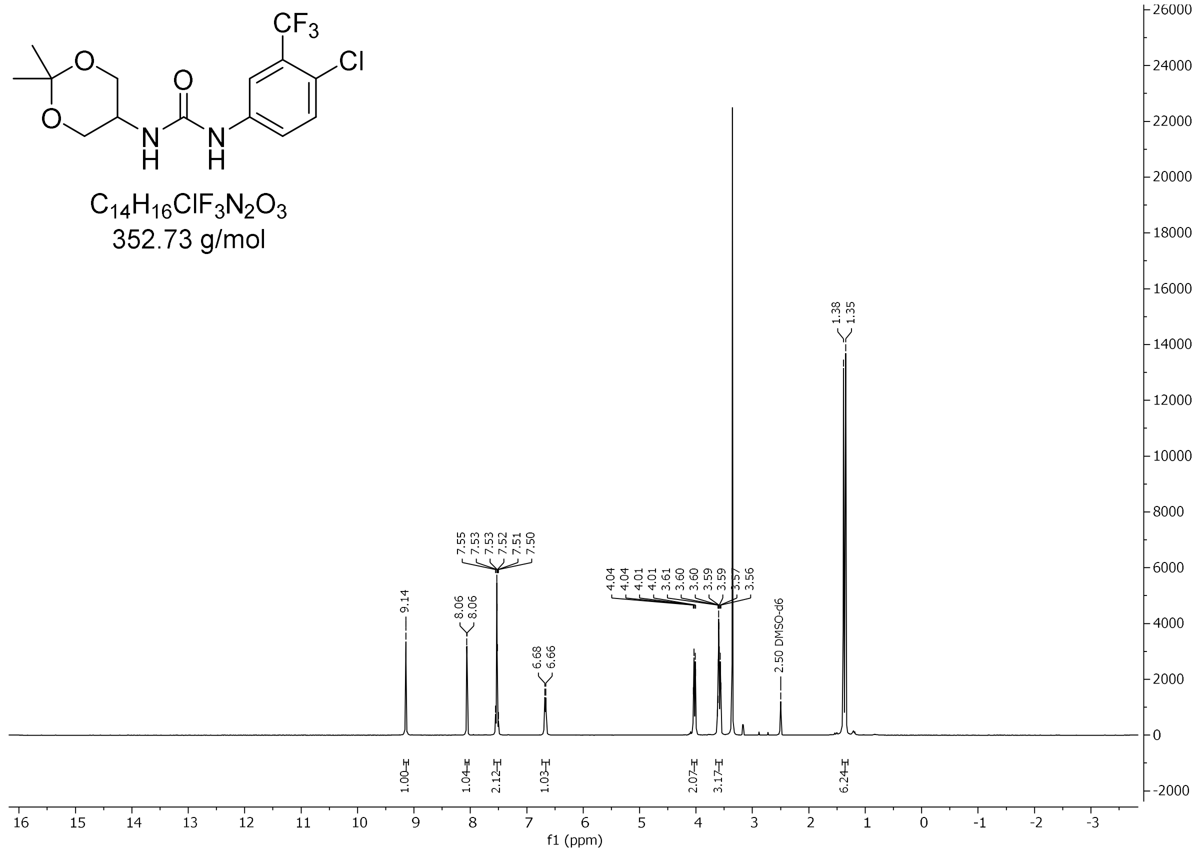
CF3
Cl
O
N
H
C14H16ClF3N2O3
352.73 g/mol
9.14
8.06
8.06
7.55
7.53
7.53
7.52
7.51
7.50
6.68
6.66
4.04
4.04
4.01
4.01
3.61
3.60
3.60
3.59
3.59
3.57
3.56
2.50 DMSO-d6
1.38
1.35
1.00
1.04
2.12
1.03
2.07
3.17
6.24
f1 (ppm)

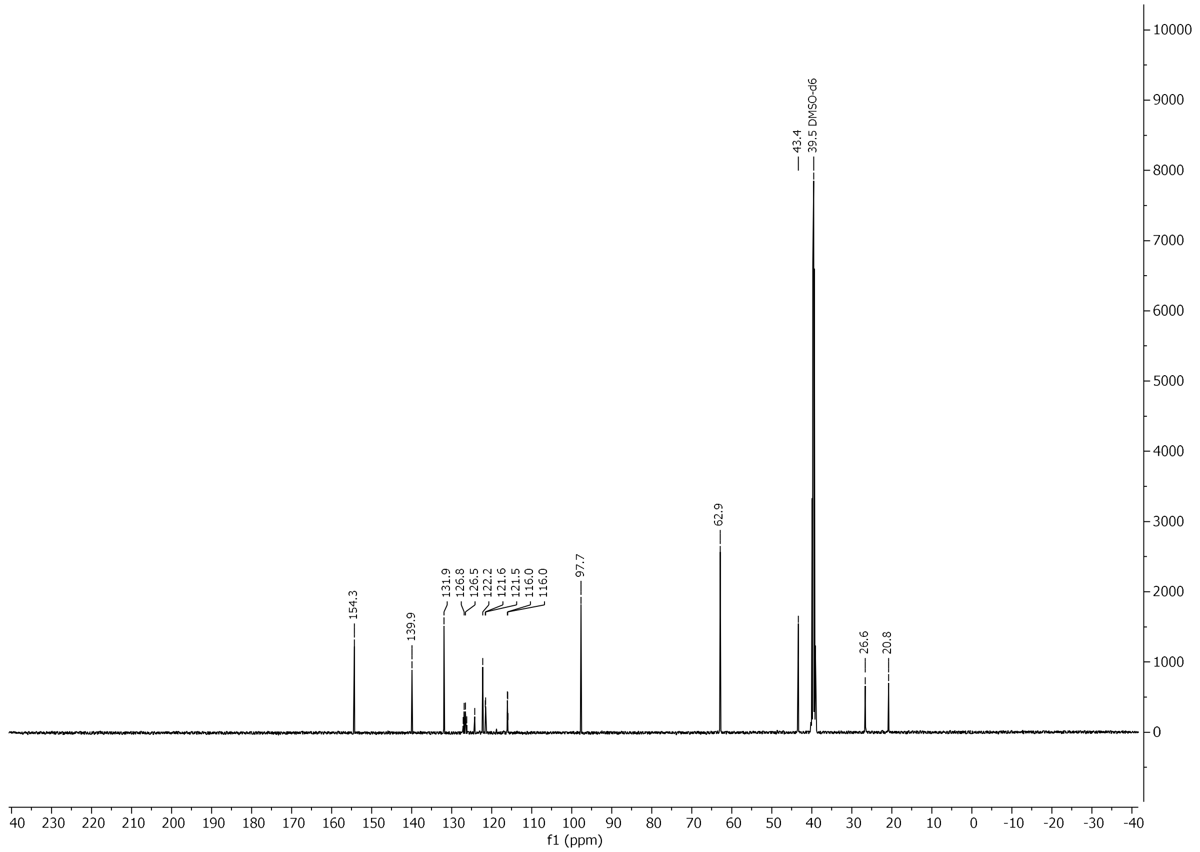
154.3
139.9
131.9
126.8
126.5
122.2
121.6
121.5
116.0
116.0
97.7
62.9
43.4
39.5 DMSO-d6
26.6
20.8
f1 (ppm)

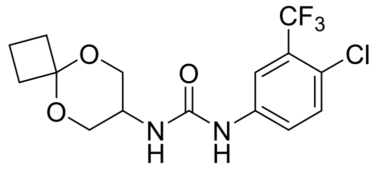

$C_{15}H_{16}ClF_3N_2O_3$
364.74 g/mol

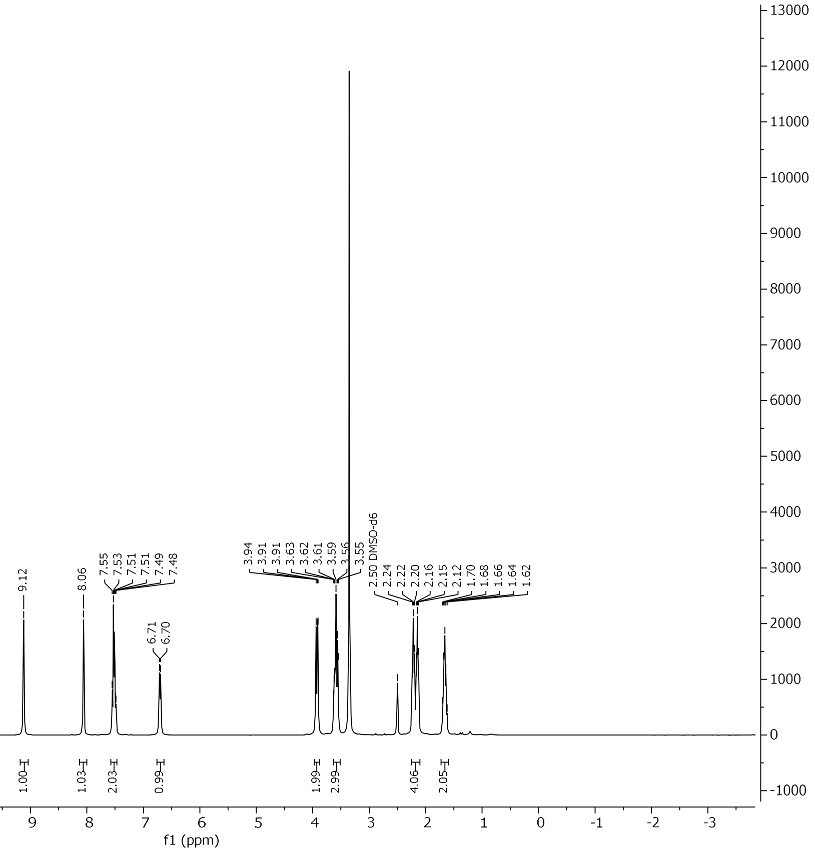

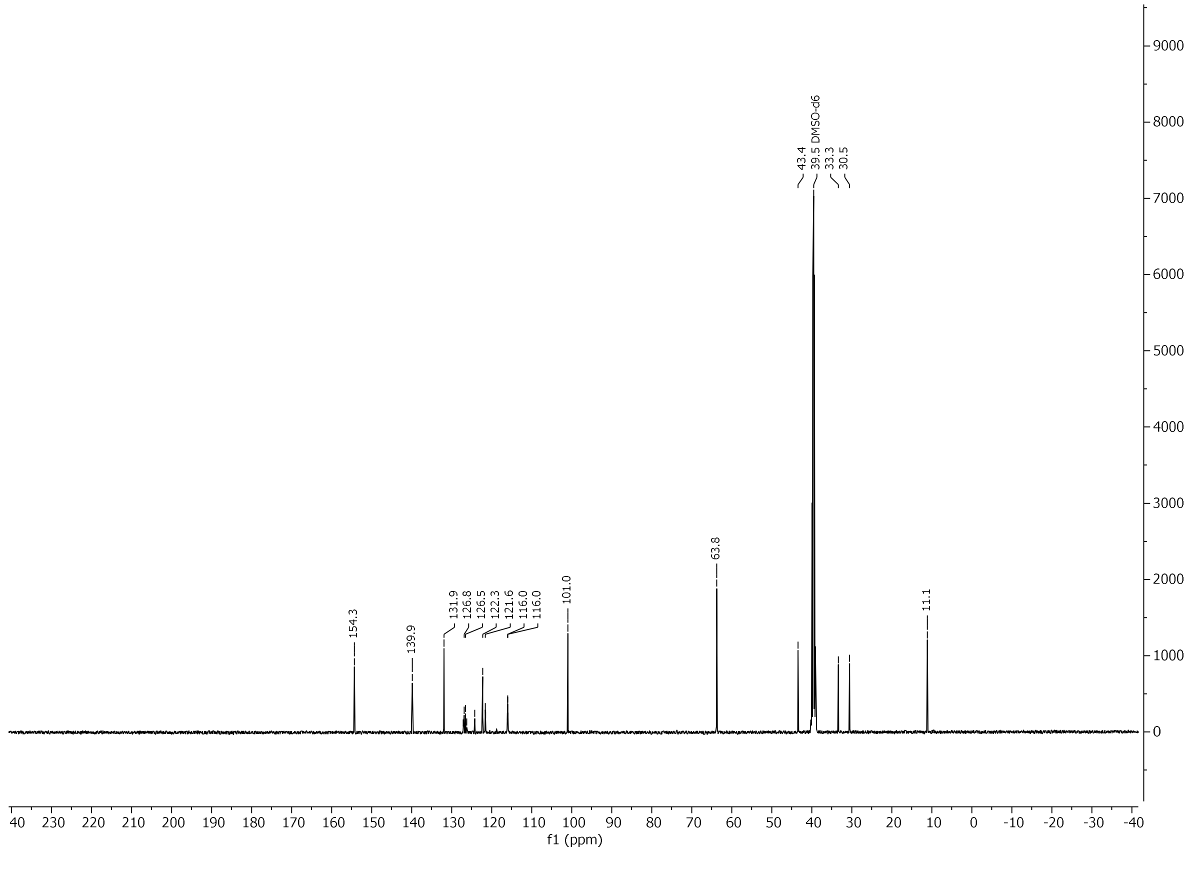

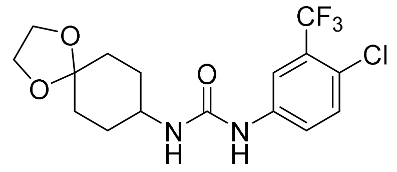

$C_{16}H_{18}ClF_3N_2O_3$
378.78 g/mol

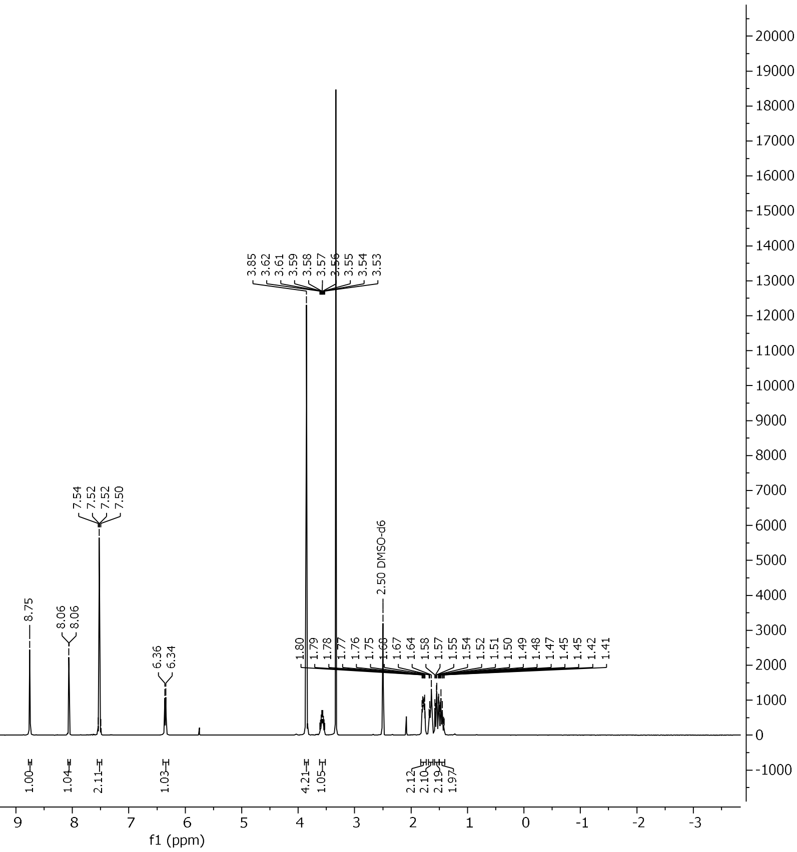

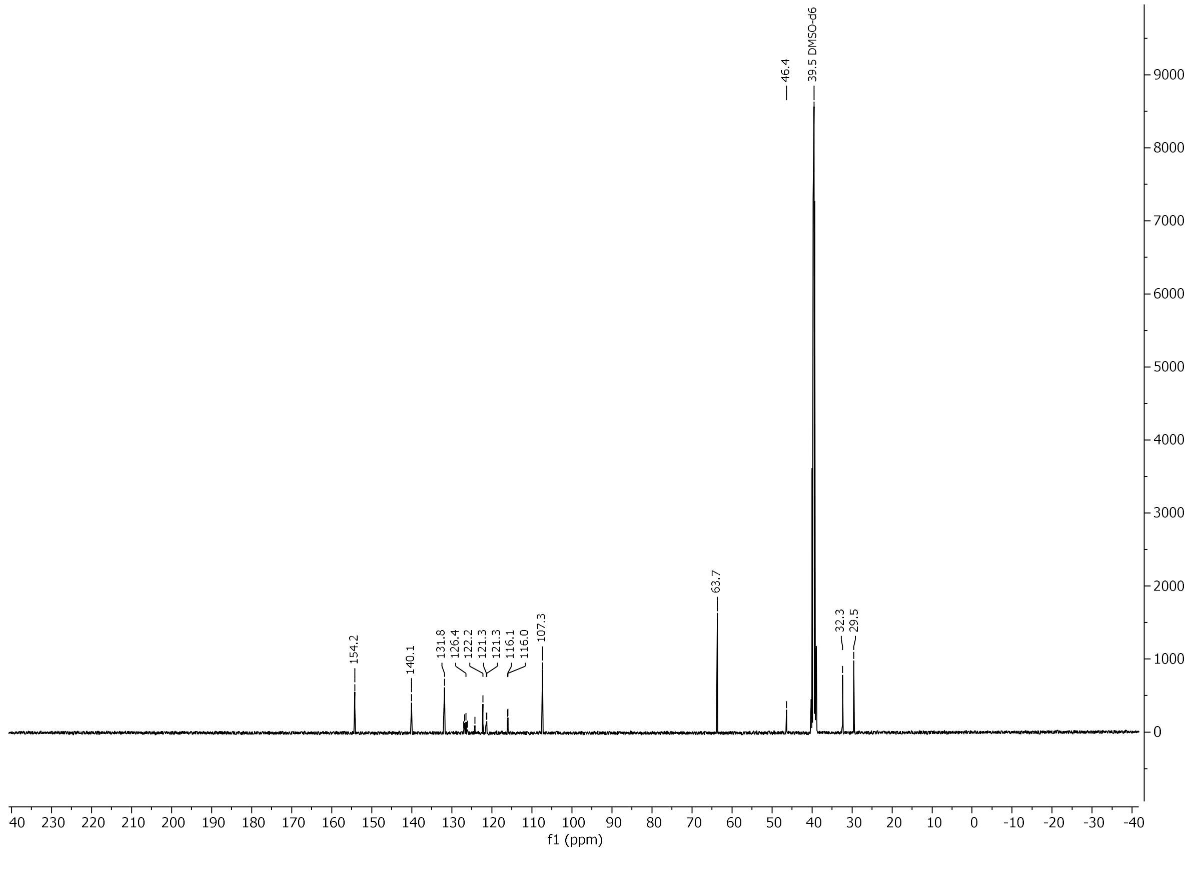

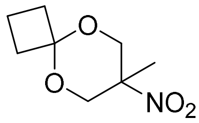

$C_8H_{13}NO_4$
187.20 g/mol

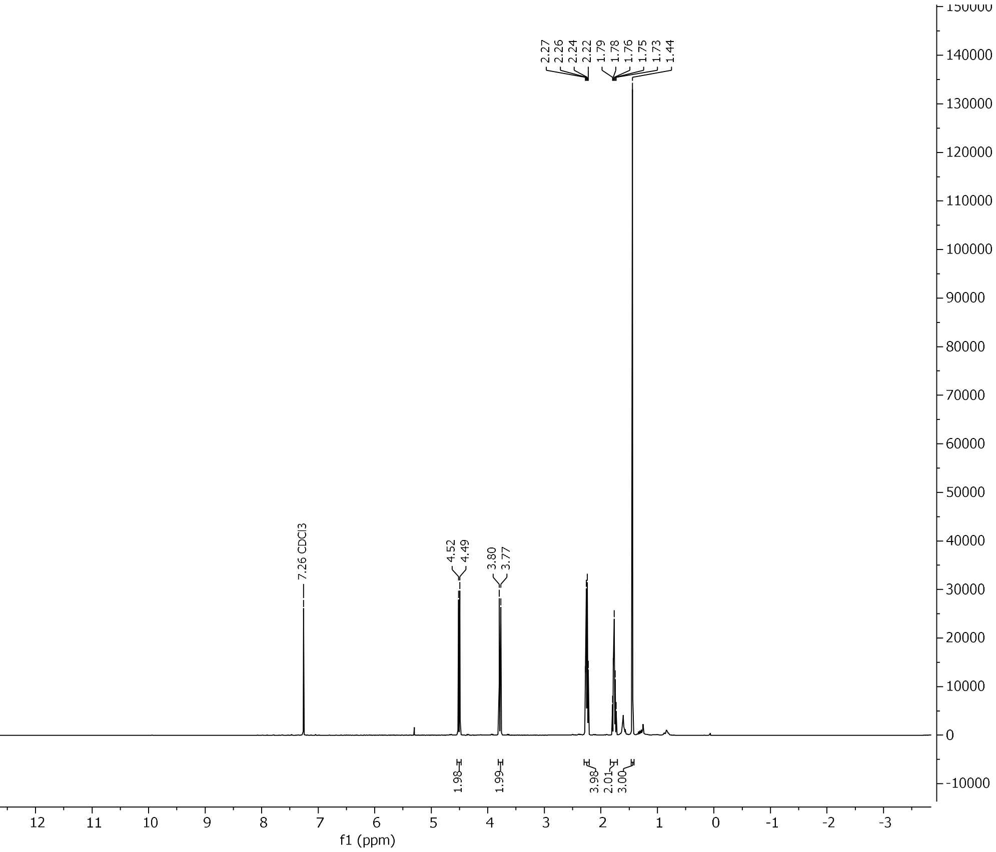

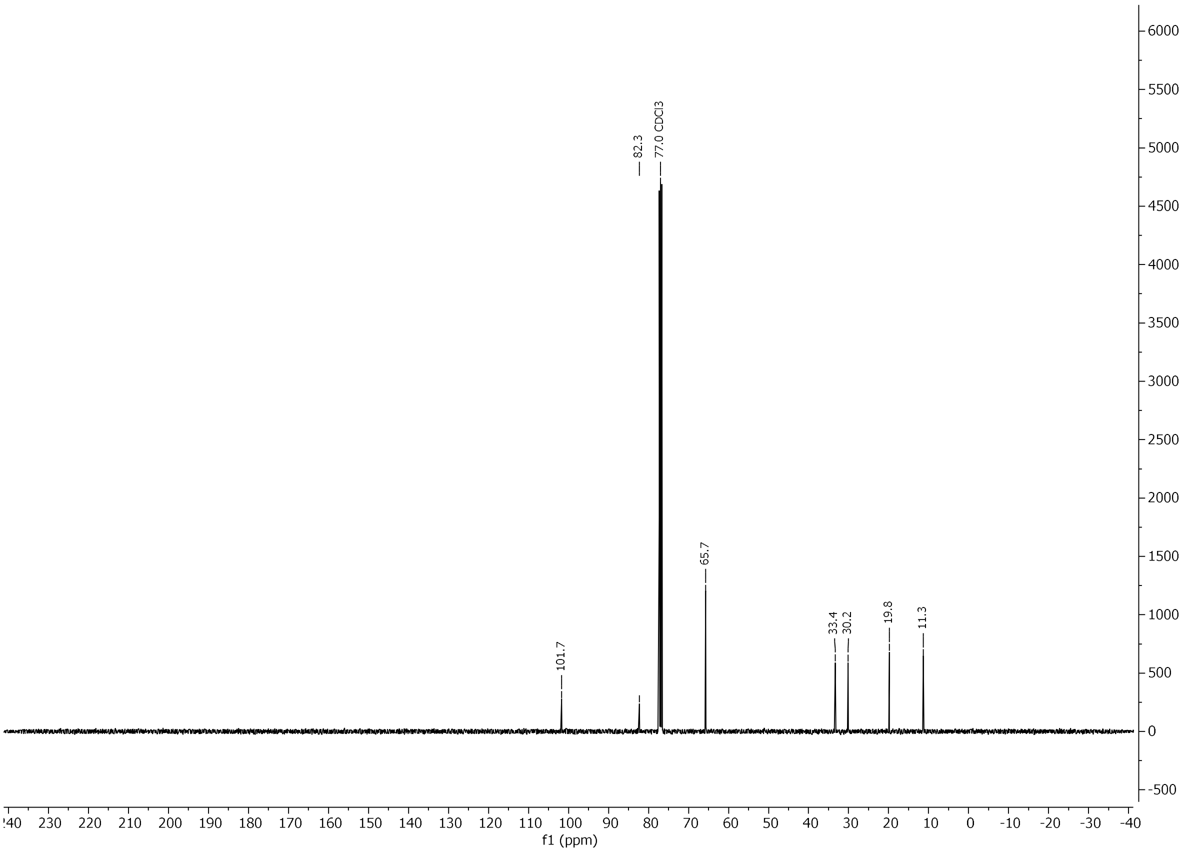

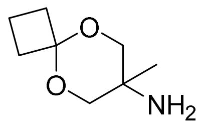

$C_8H_{15}NO_2$
157.21 g/mol

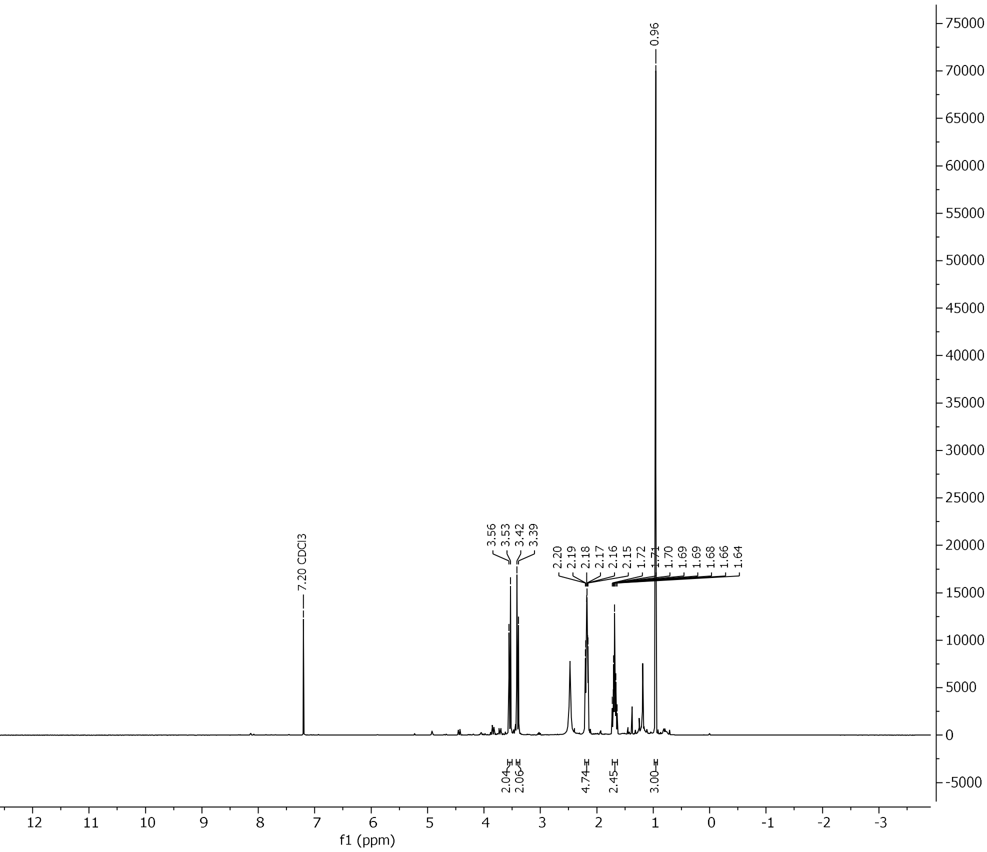

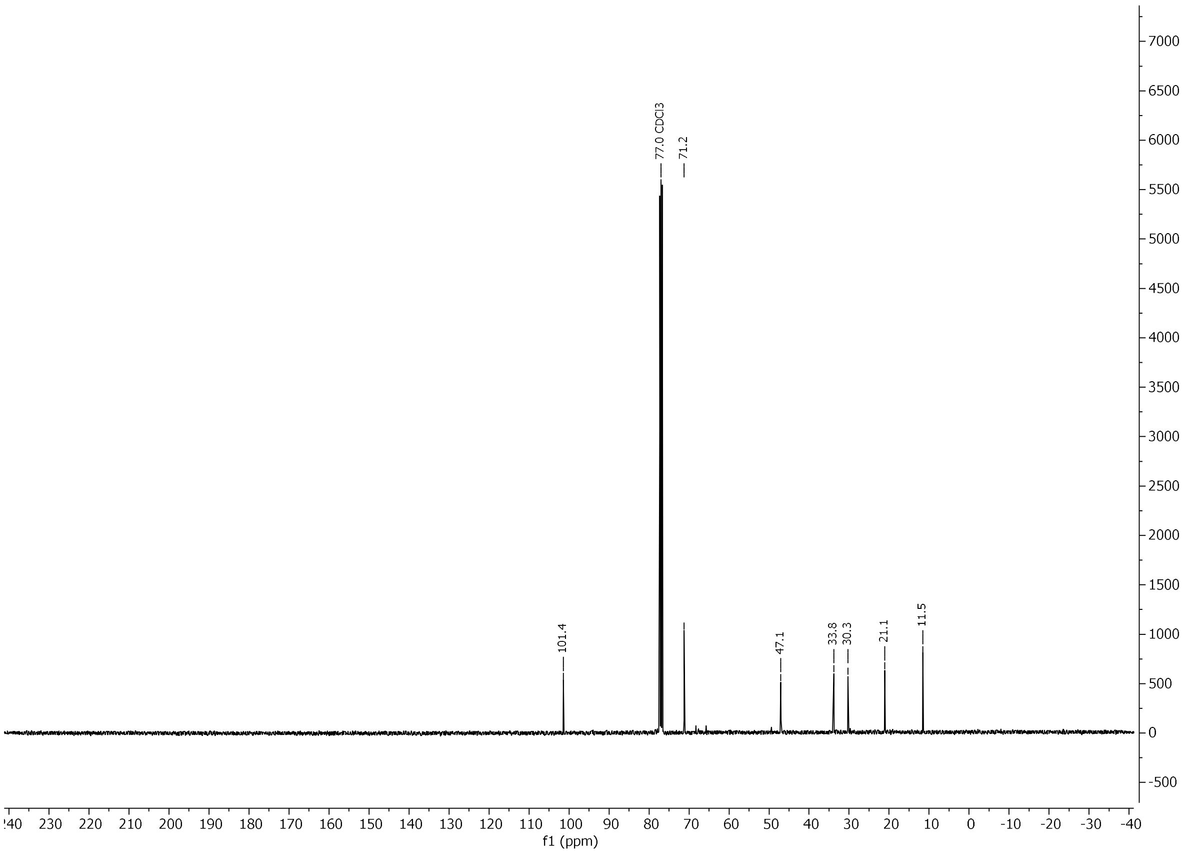

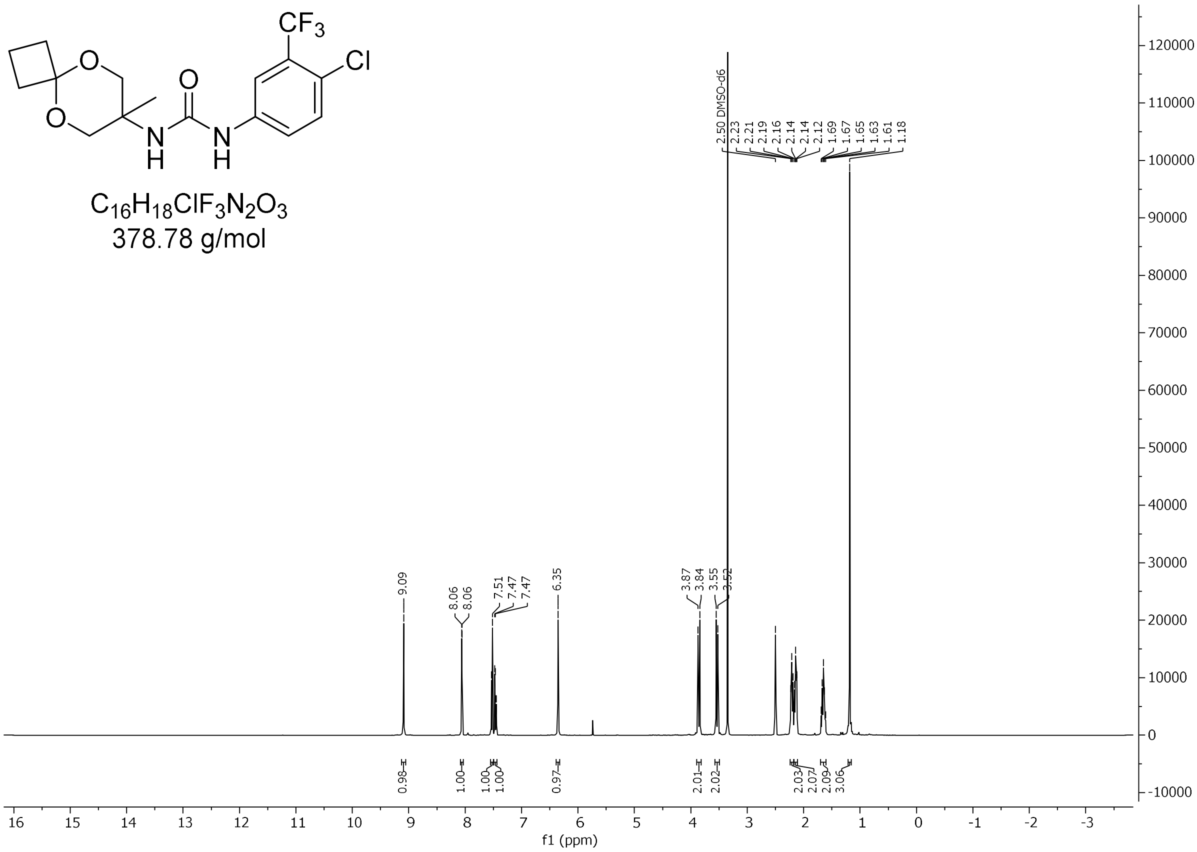
CF3
Cl
O
N
H
C16H18ClF3N2O3
378.78 g/mol
2.50 DMSO-d6
2.23
2.21
2.19
2.16
2.14
2.14
2.12
1.69
1.67
1.65
1.63
1.61
1.18
9.09
8.06
8.06
7.51
7.47
7.47
6.35
3.87
3.84
3.55
3.52
0.98
1.00
1.00
1.00
0.97
2.01
2.02
2.03
2.07
2.09
3.06
f1 (ppm)

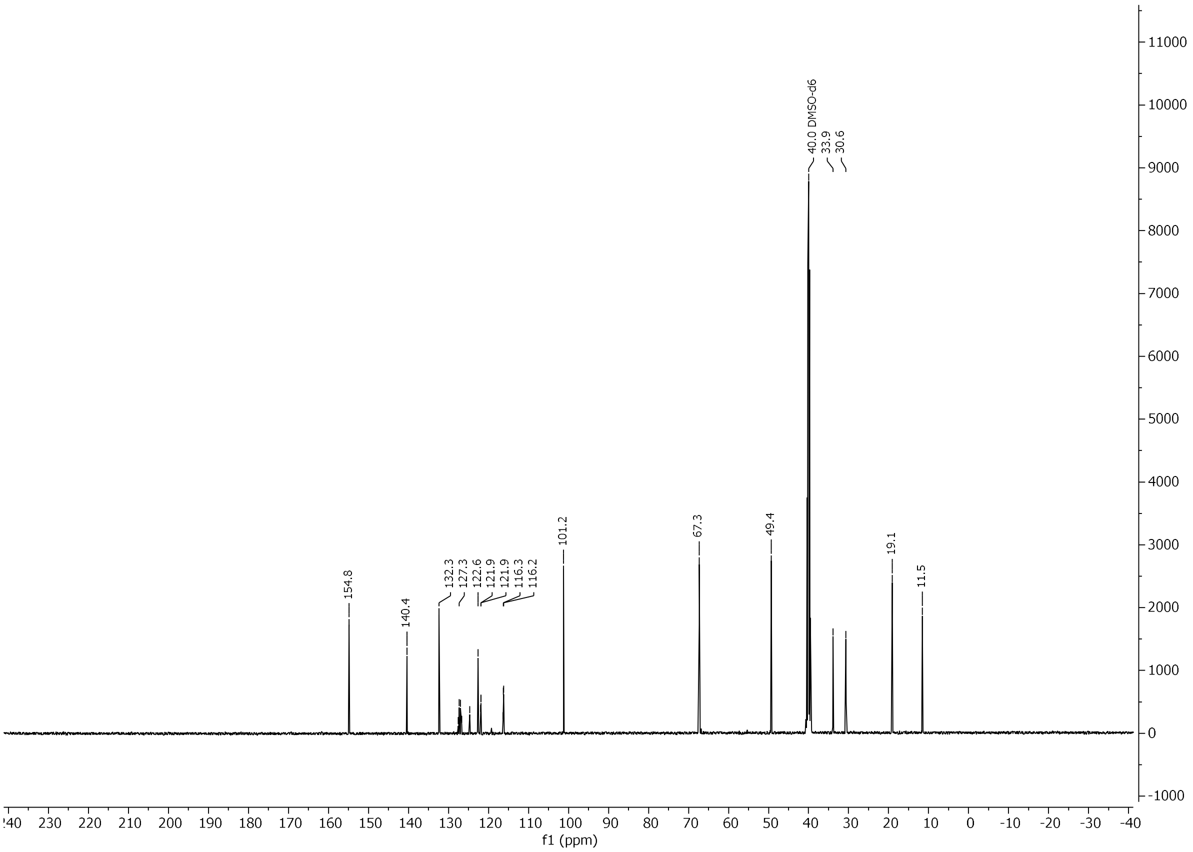
40.0 DMSO-d6
33.9
30.6
154.8
140.4
132.3
127.3
122.6
121.9
121.9
116.3
116.2
101.2
67.3
49.4
19.1
11.5
f1 (ppm)

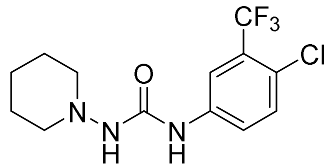

$C_{13}H_{15}ClF_3N_3O$

321.72 g/mol

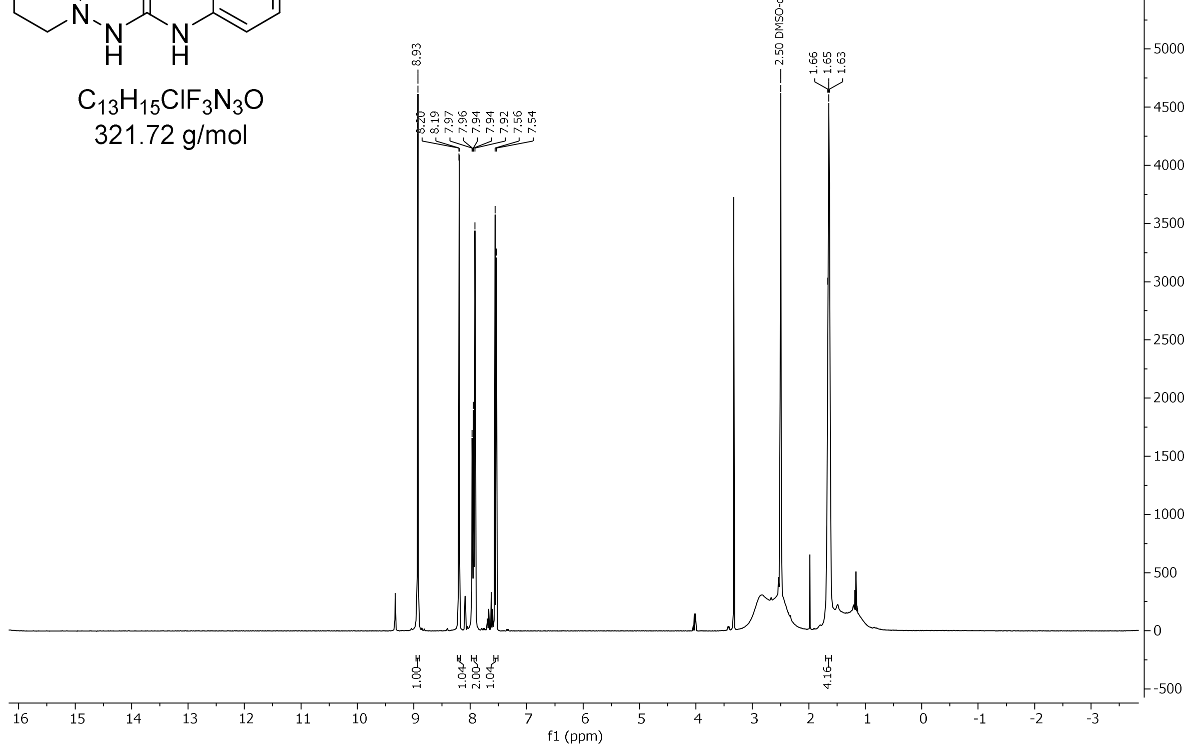

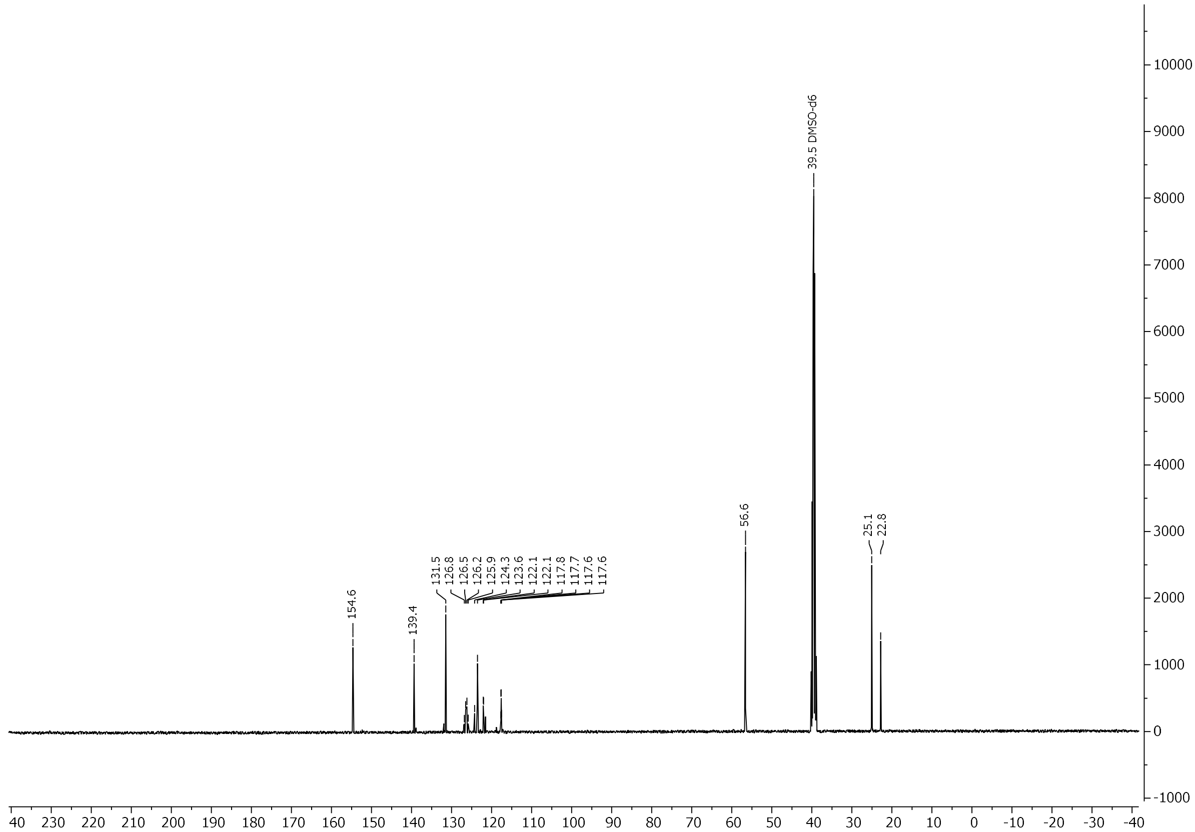

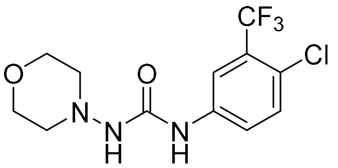

$C_{12}H_{13}ClF_3N_3O_2$
323.70 g/mol

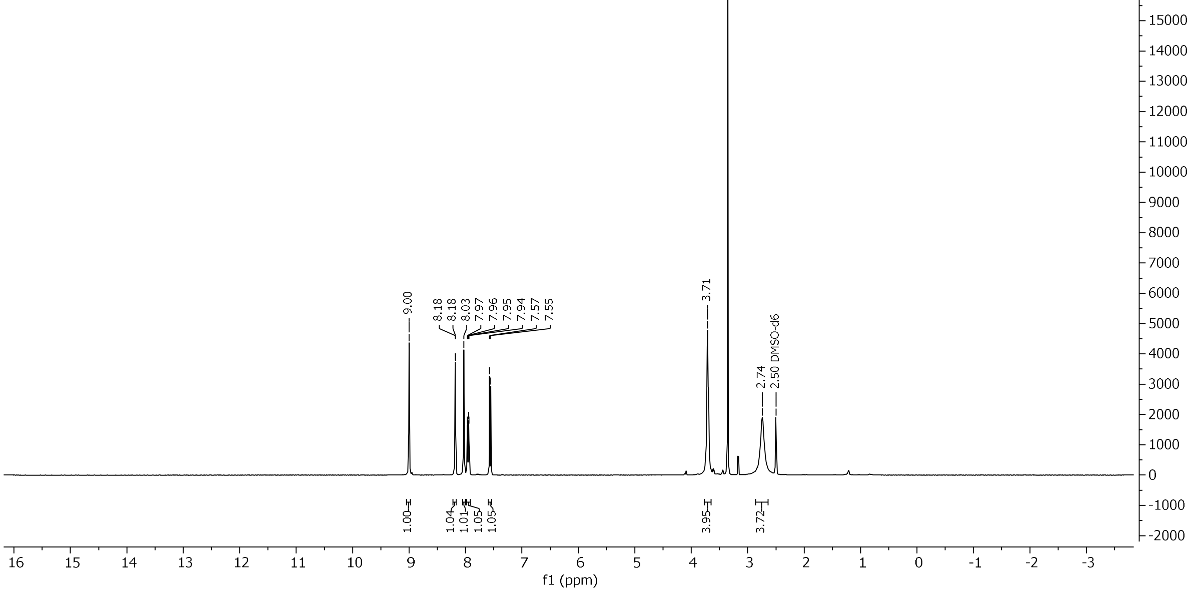

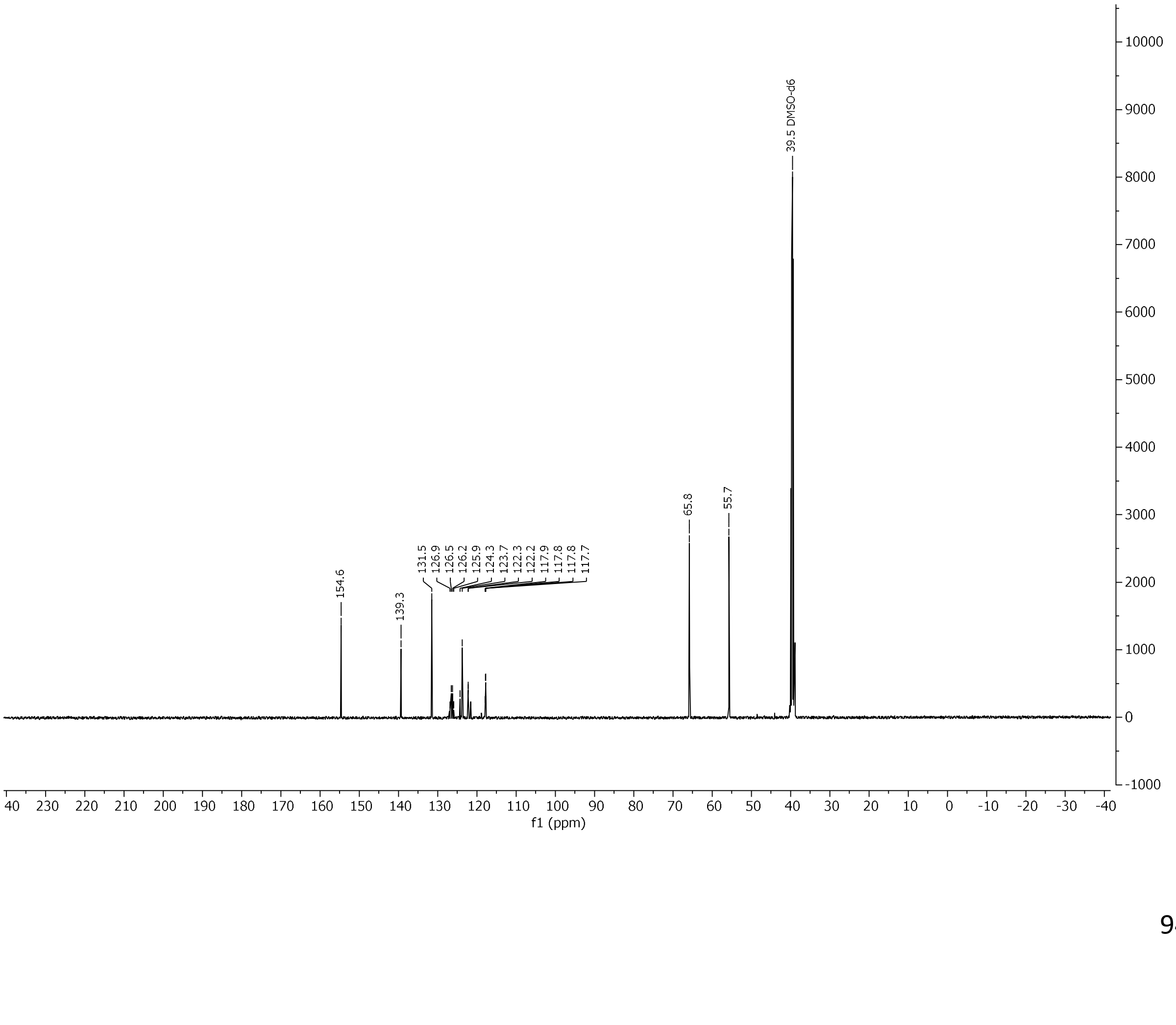

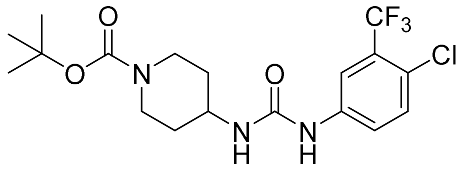

$C_{18}H_{23}ClF_3N_3O_3$
421.85 g/mol

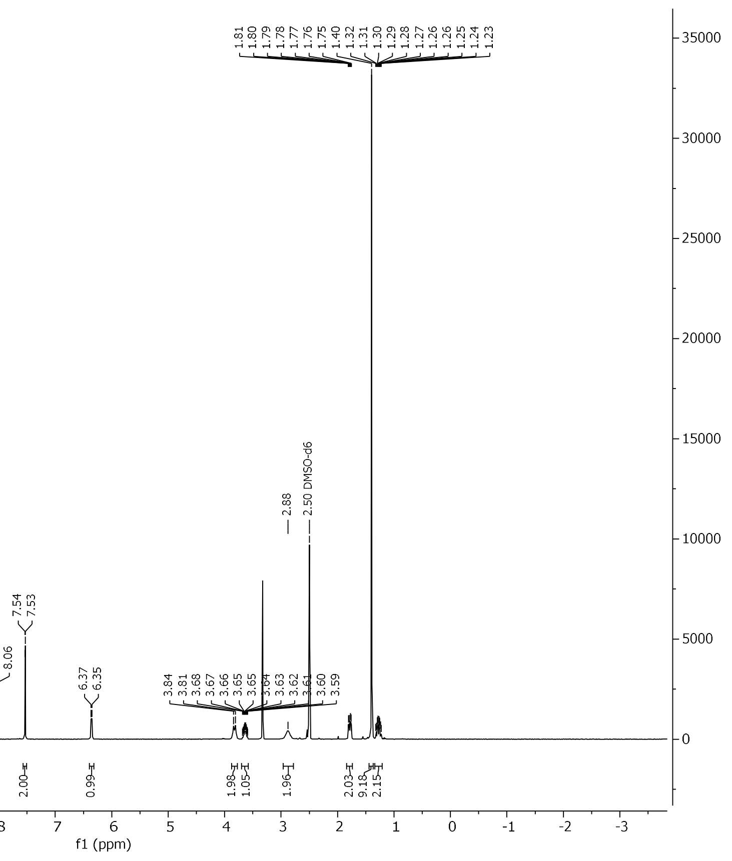

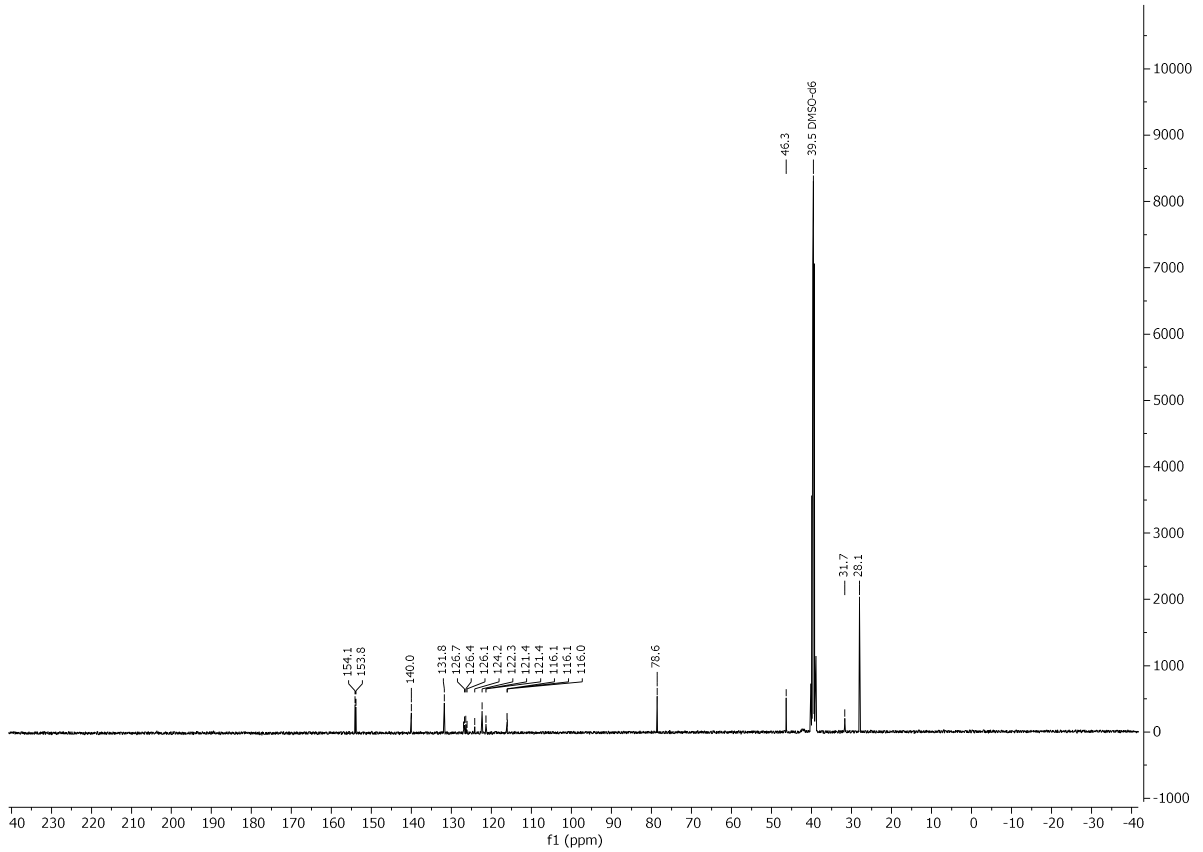

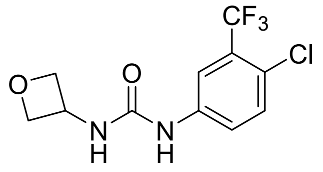

$C_{11}H_{10}ClF_3N_2O_2$
294.66 g/mol

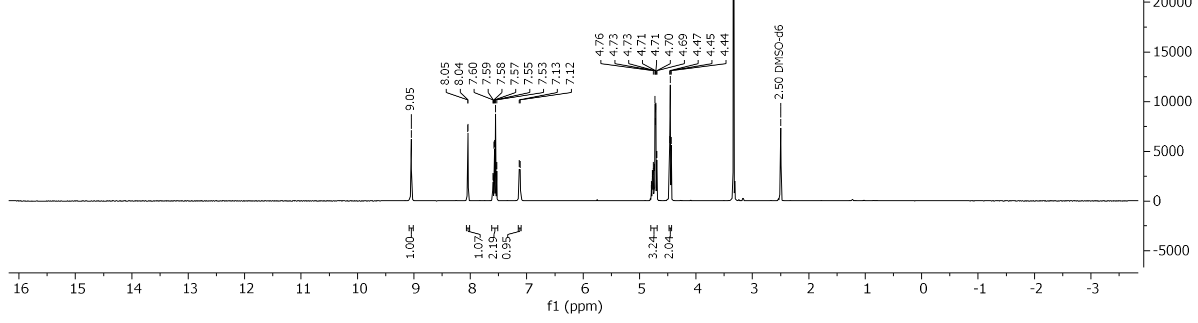

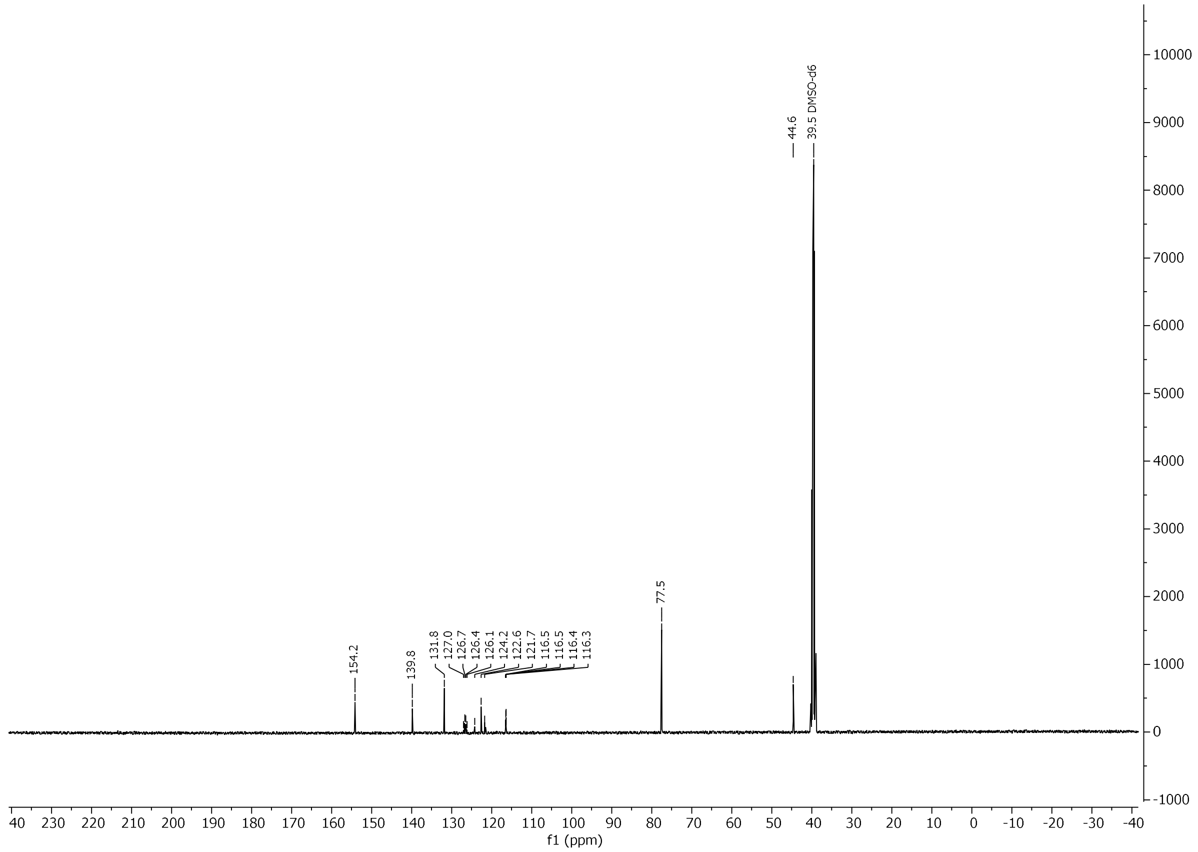

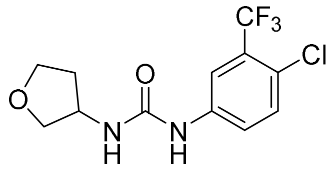

$C_{12}H_{12}ClF_3N_2O_2$
308.69 g/mol

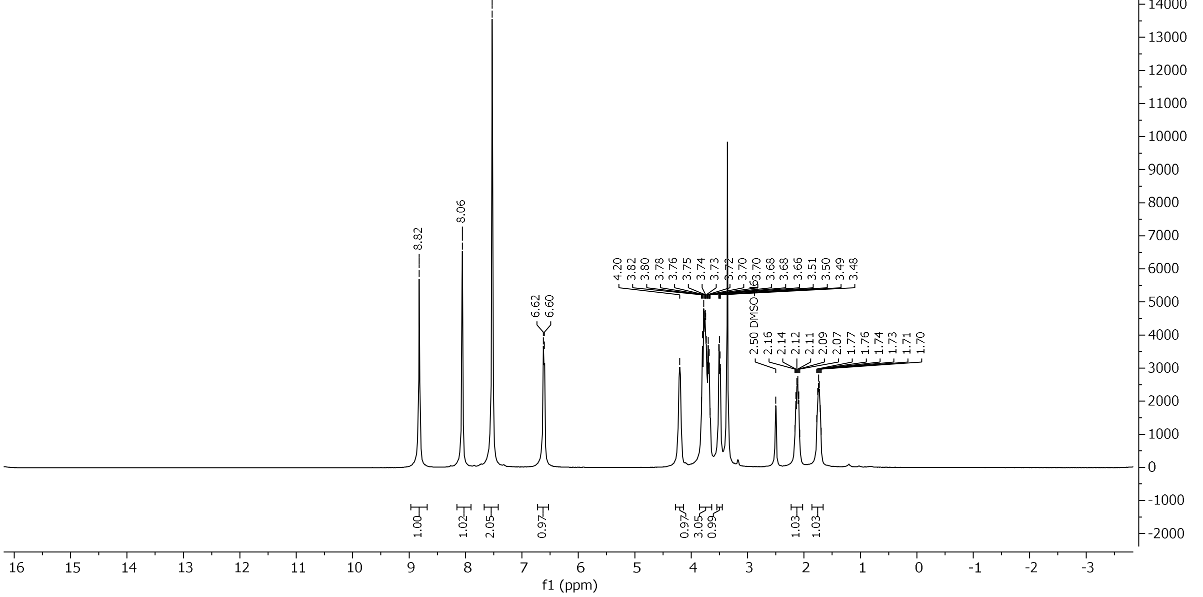

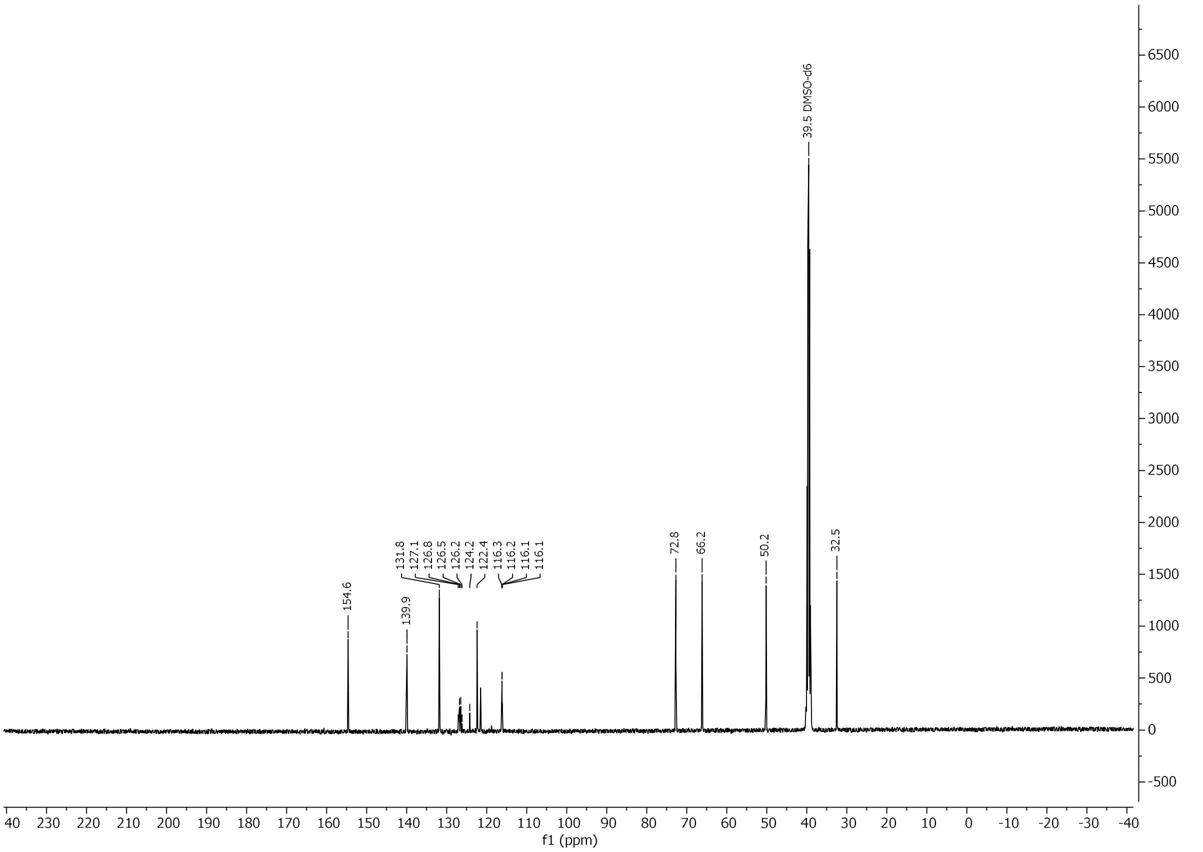

$C_{12}H_{12}ClF_3N_2O_2$
308.69 g/mol

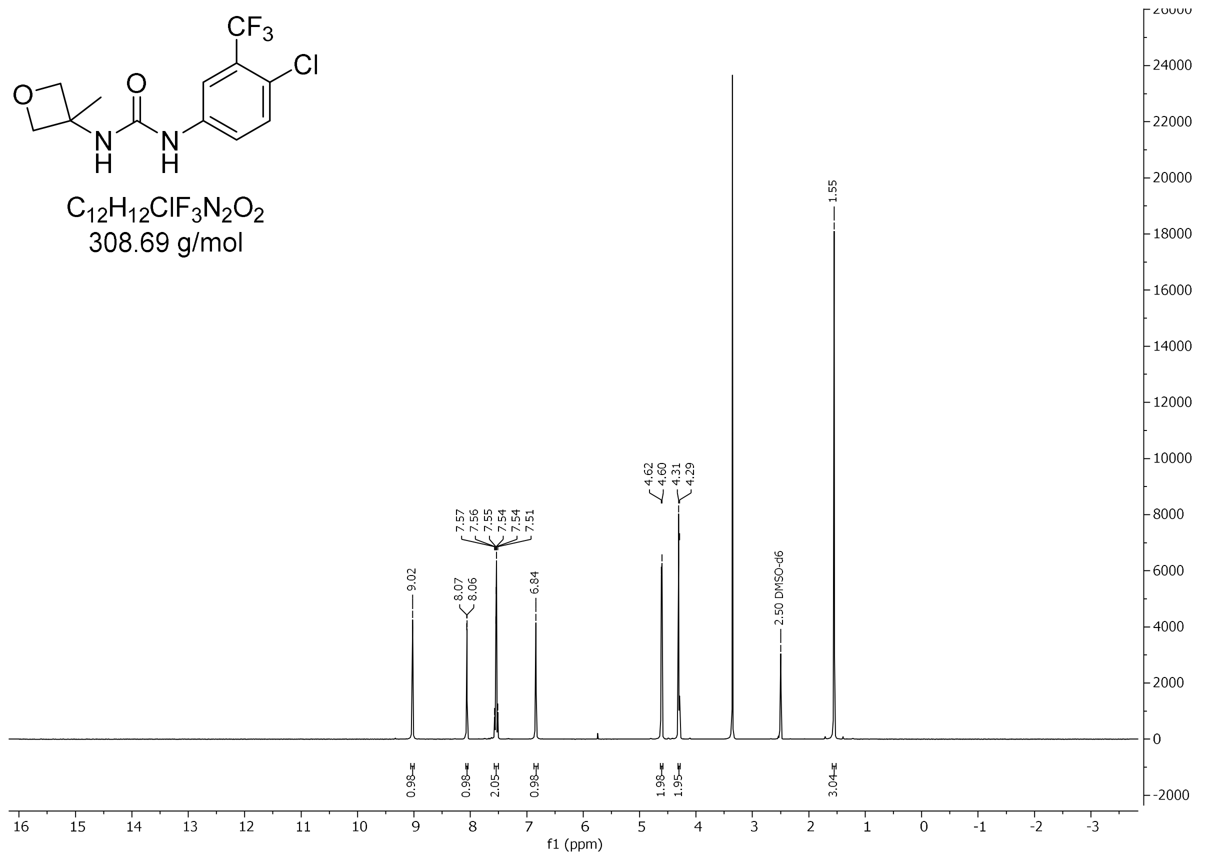

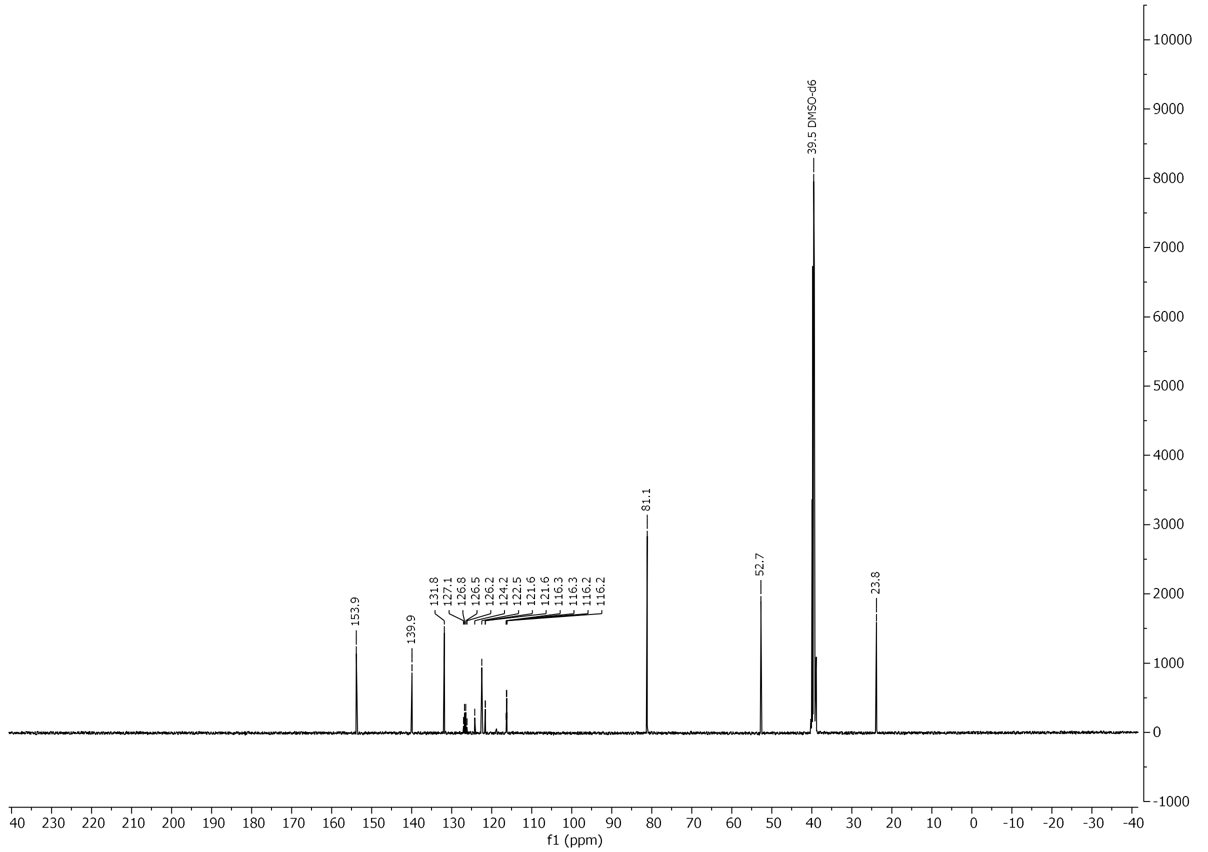

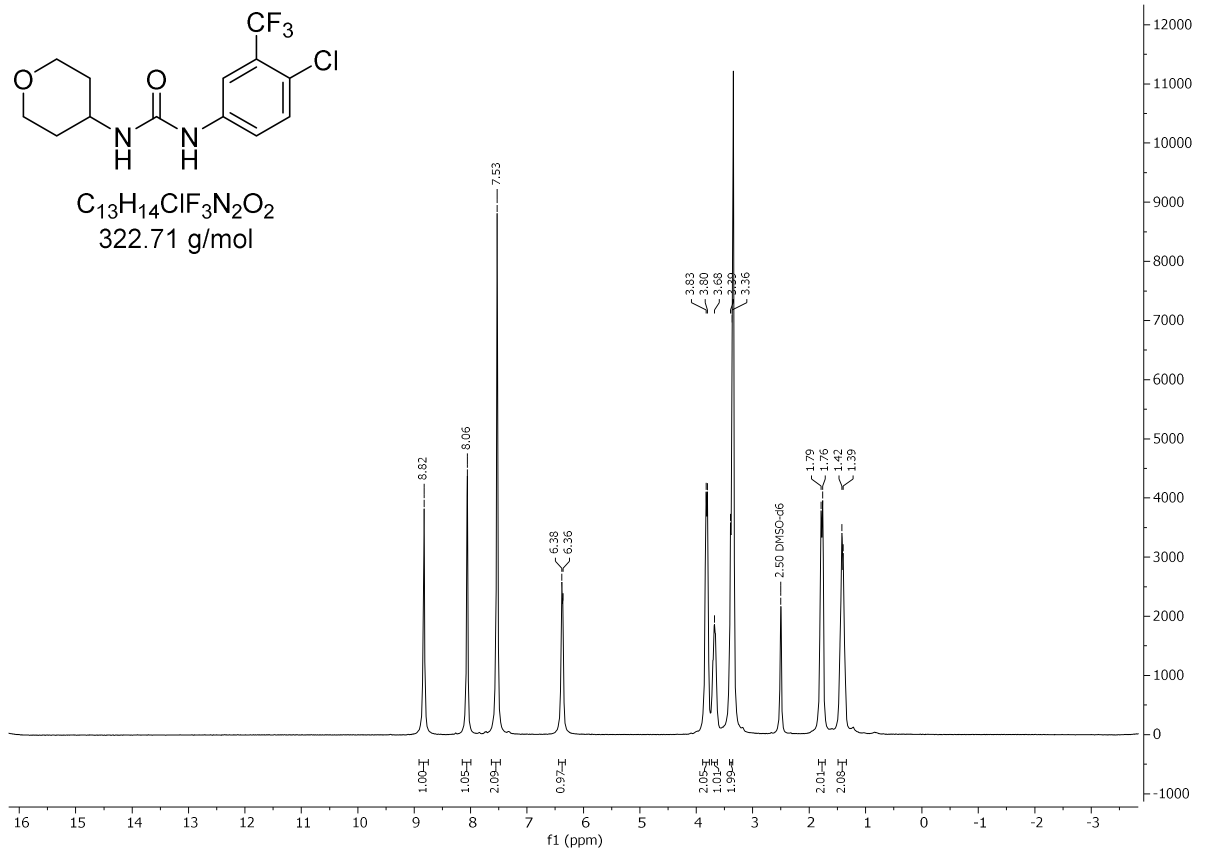
CF3
Cl
O
O
N
H
N
H
C13H14ClF3N2O2
322.71 g/mol
7.53
3.83
3.80
3.68
3.39
3.36
8.82
8.06
6.38
6.36
2.50 DMSO-d6
1.79
1.76
1.42
1.39
1.00
1.05
2.09
0.97
2.05
1.01
1.99
2.01
2.08
f1 (ppm)

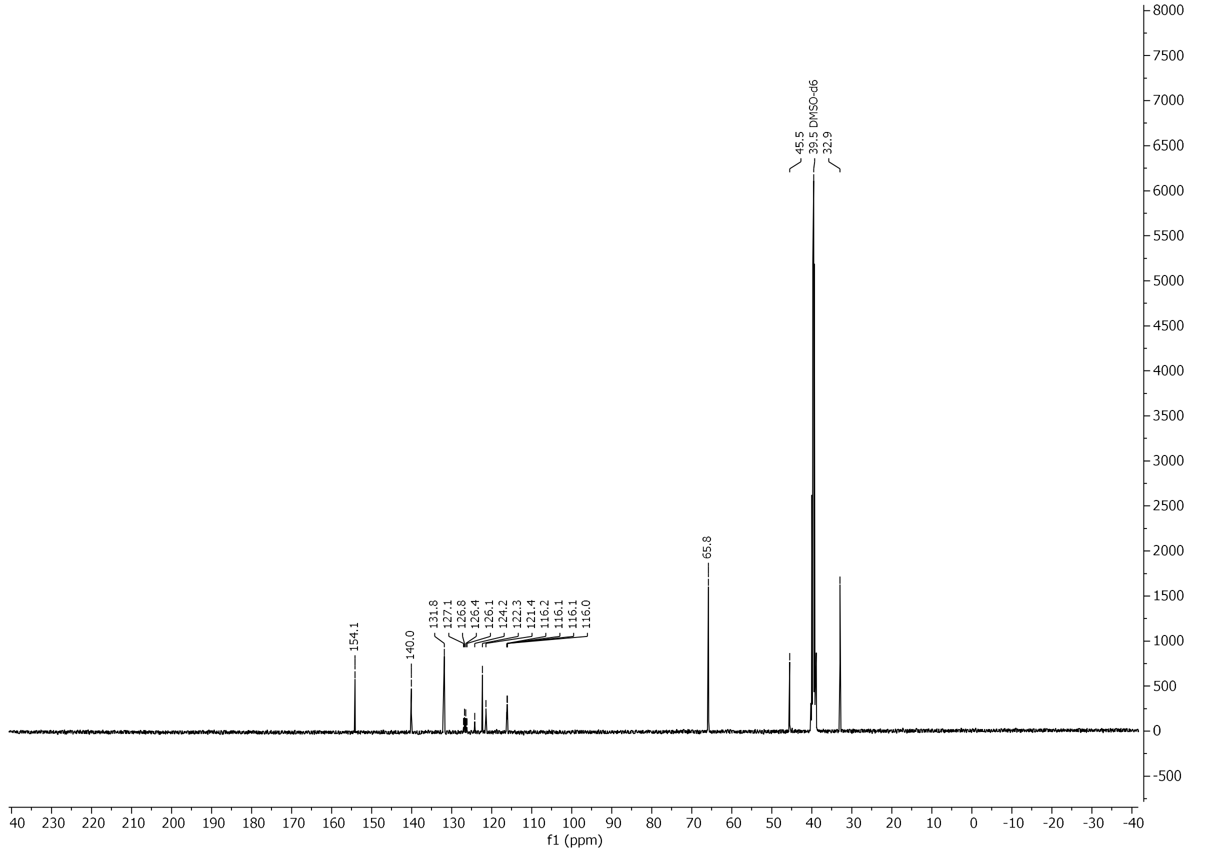
45.5
39.5 DMSO-d6
32.9
65.8
154.1
140.0
131.8
127.1
126.8
126.4
126.1
124.2
122.3
121.4
116.2
116.1
116.1
116.0
f1 (ppm)

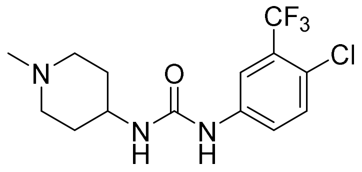

$C_{14}H_{17}ClF_3N_3O$
335.76 g/mol

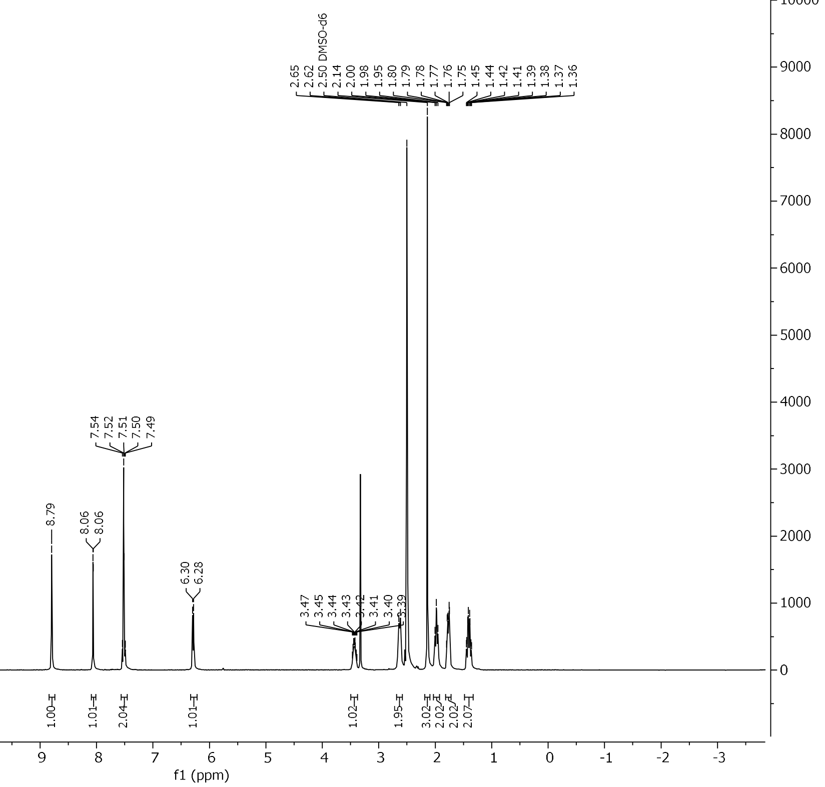

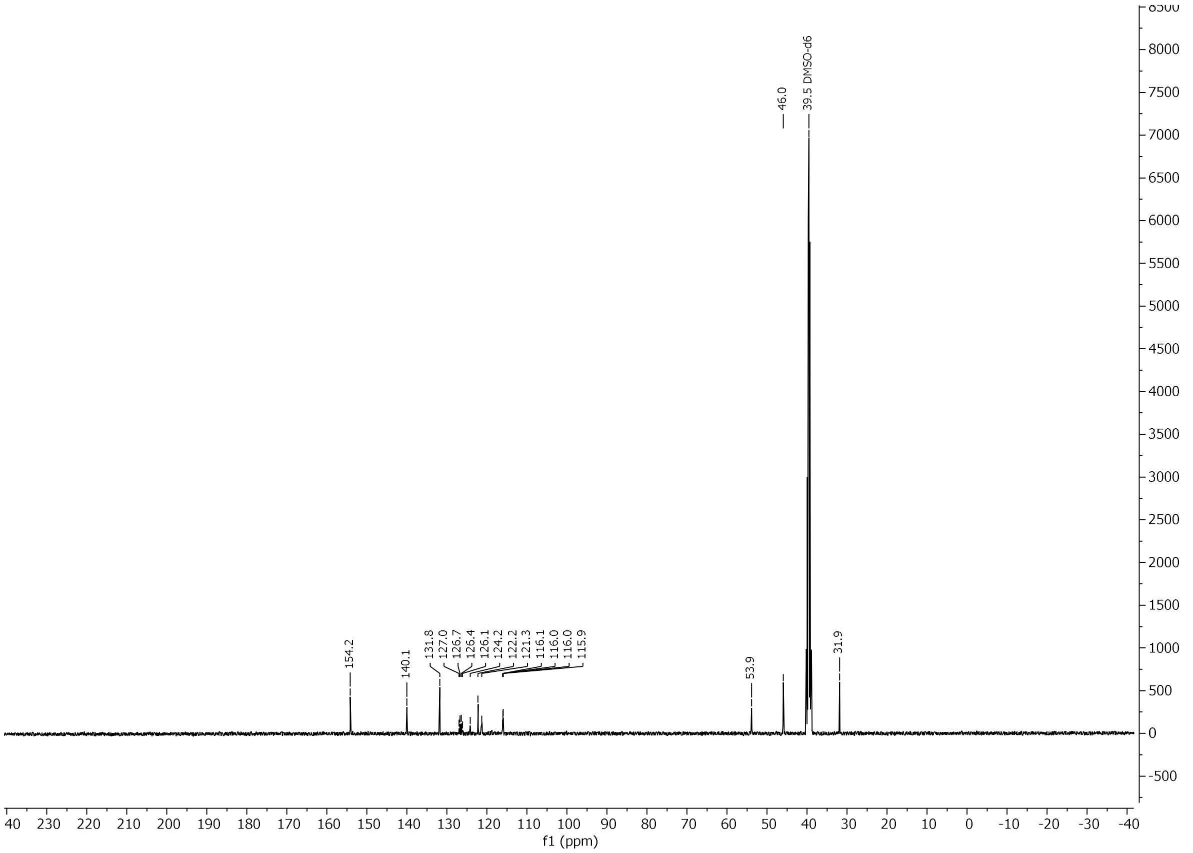

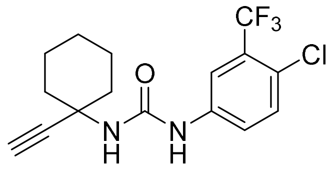

$C_{16}H_{16}ClF_3N_2O$
344.76 g/mol

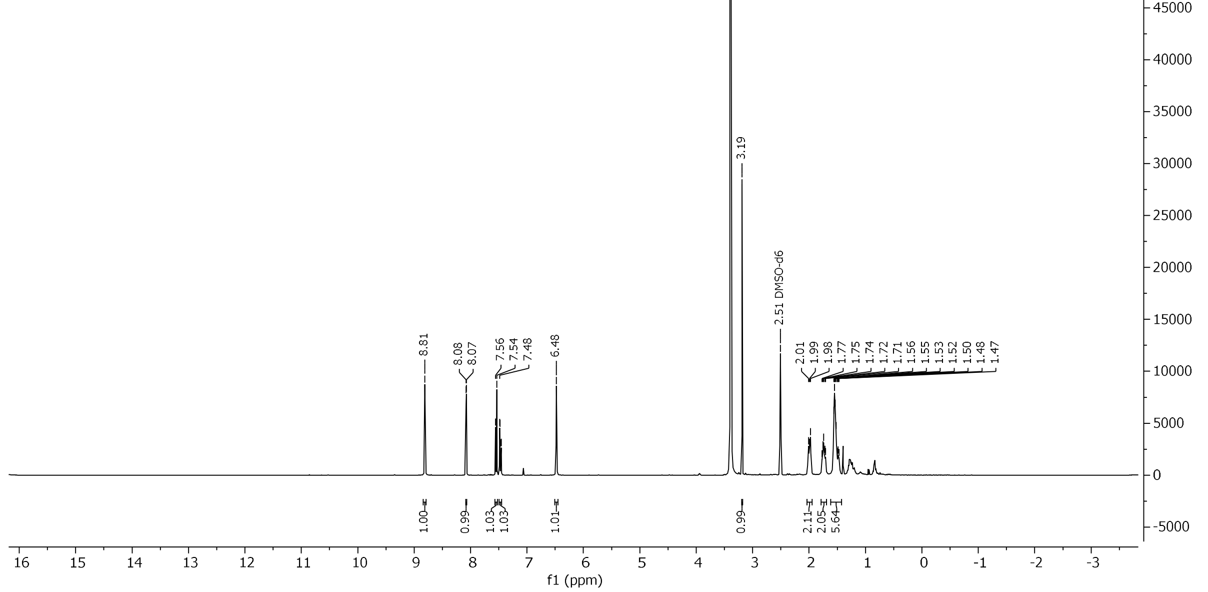

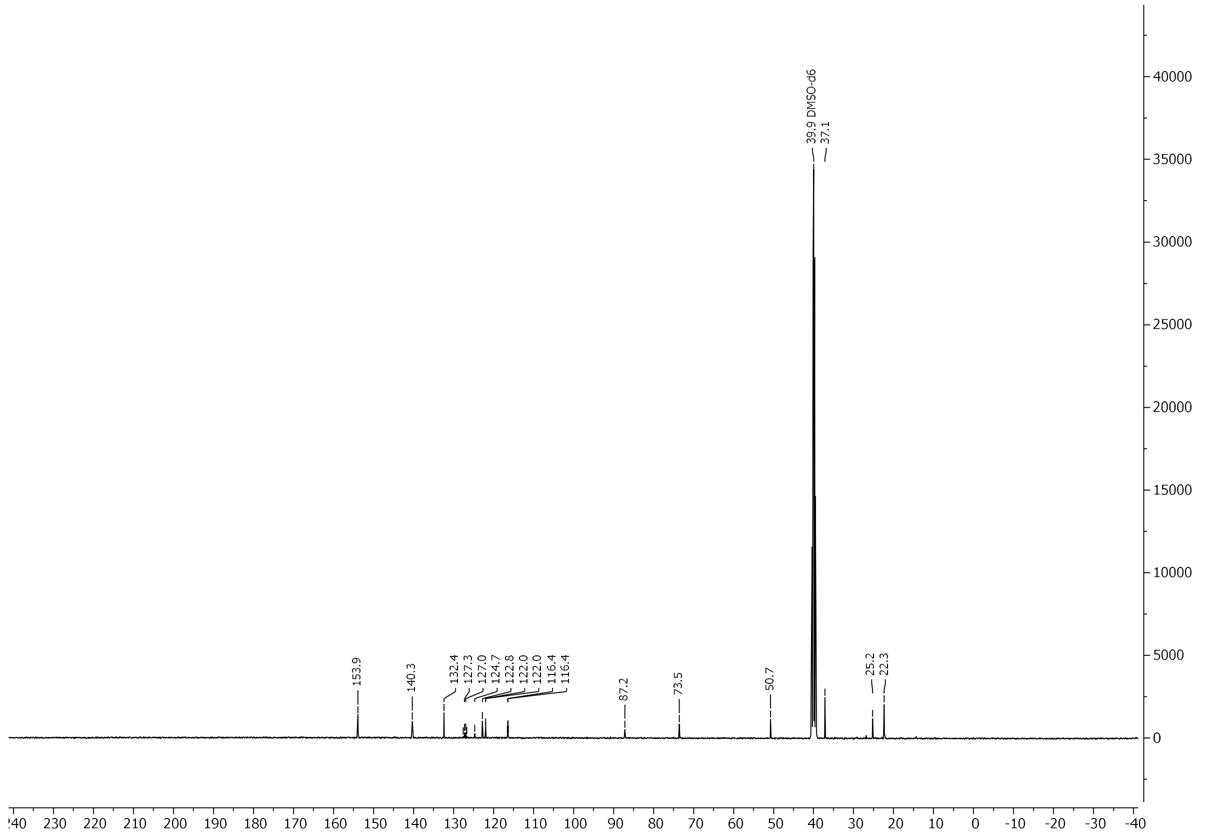

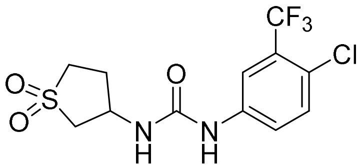

$C_{12}H_{12}ClF_3N_2O_3S$
356.74 g/mol

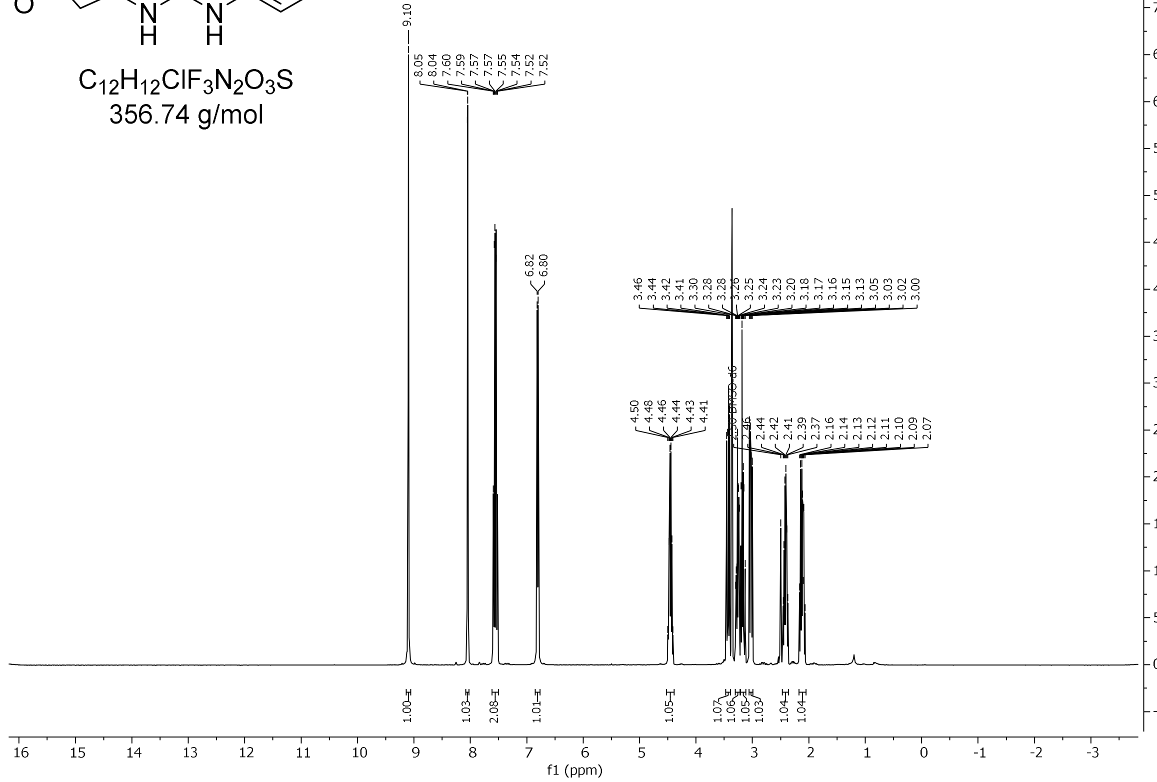

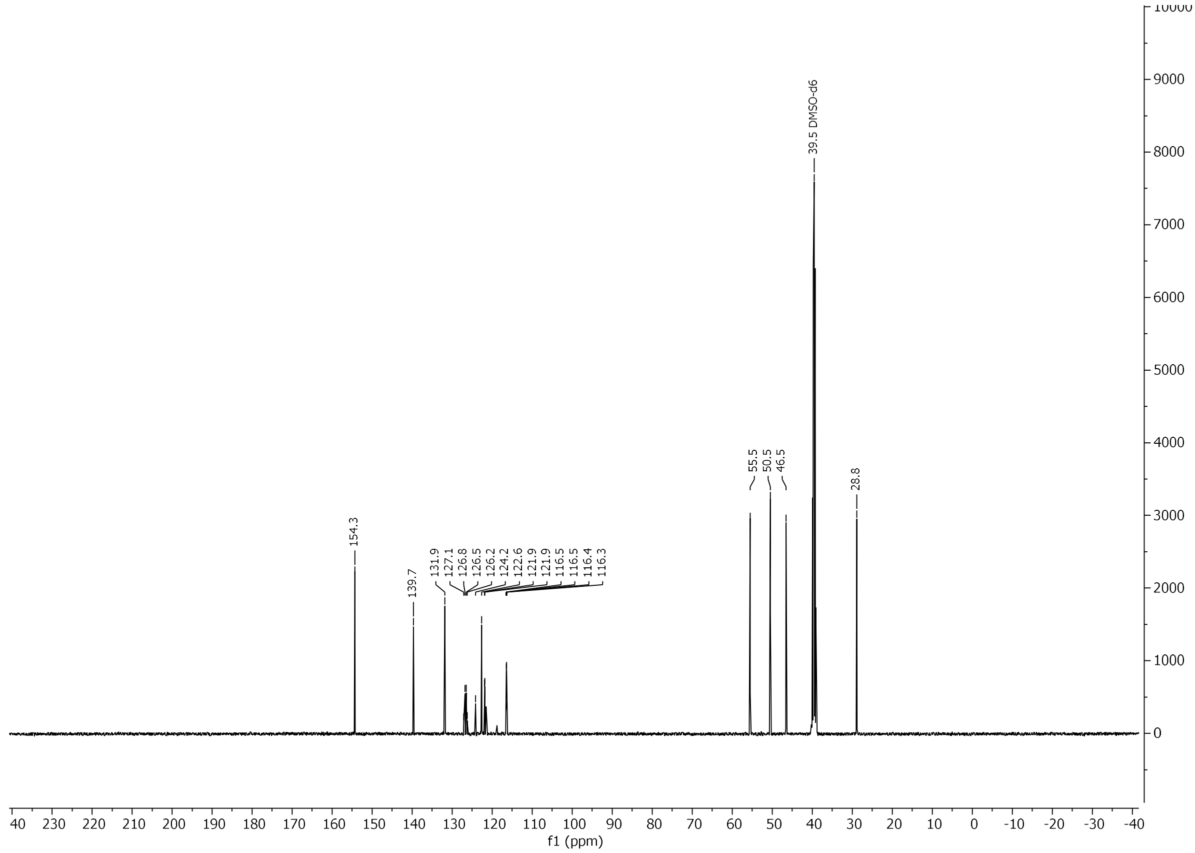

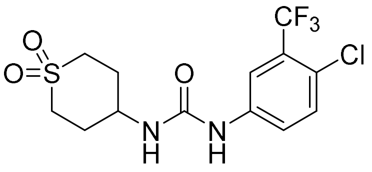

$C_{13}H_{14}ClF_3N_2O_3S$
370.77 g/mol

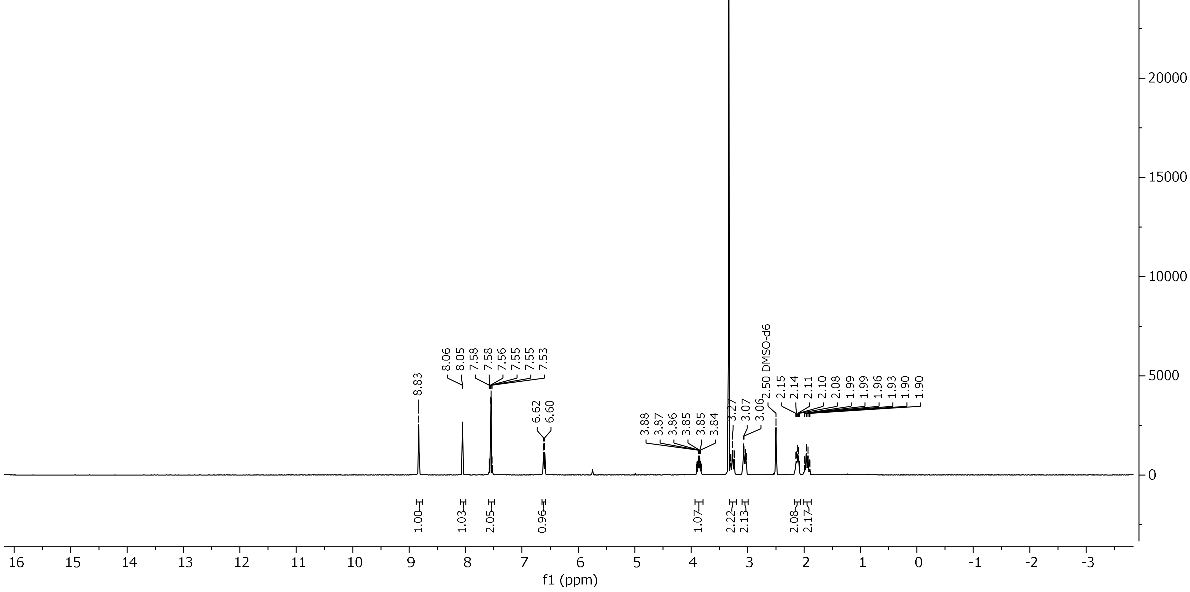

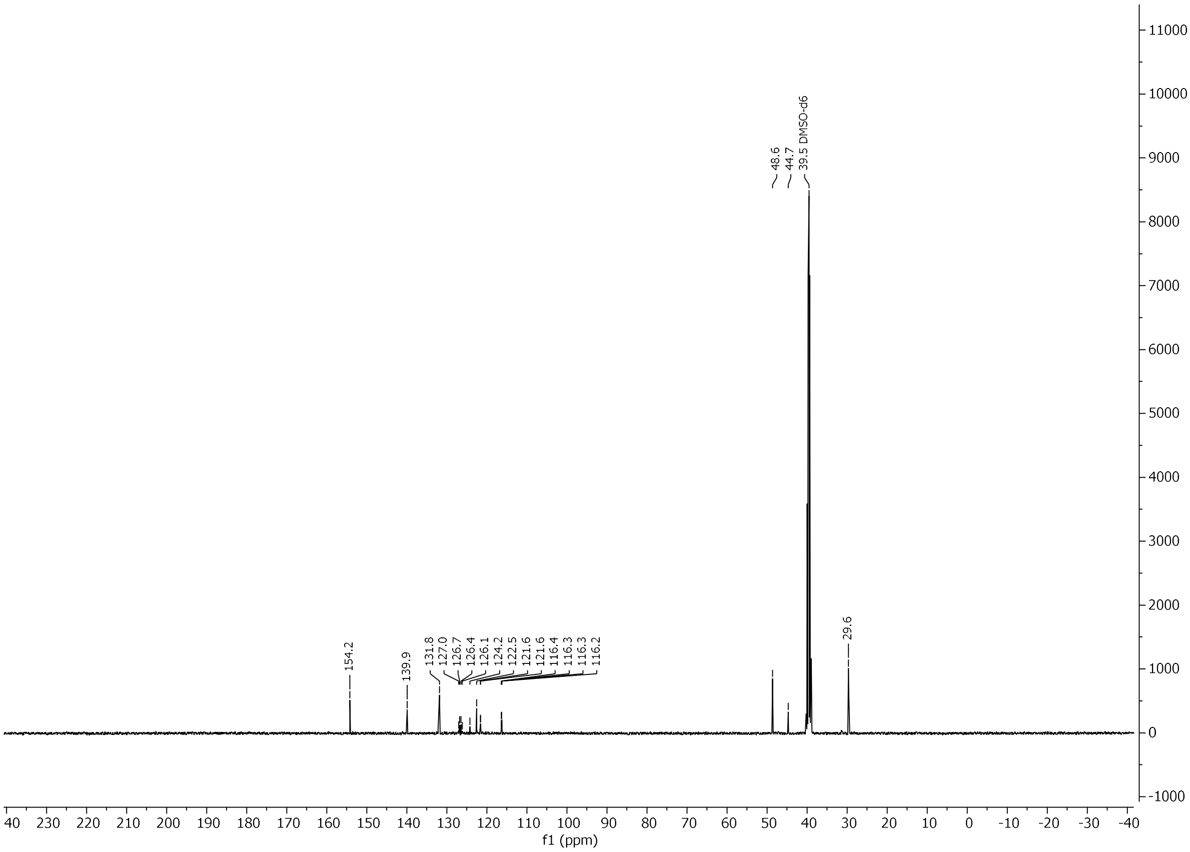

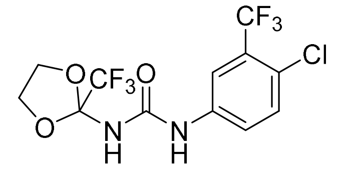

$C_{12}H_9ClF_6N_2O_3$
378.66 g/mol

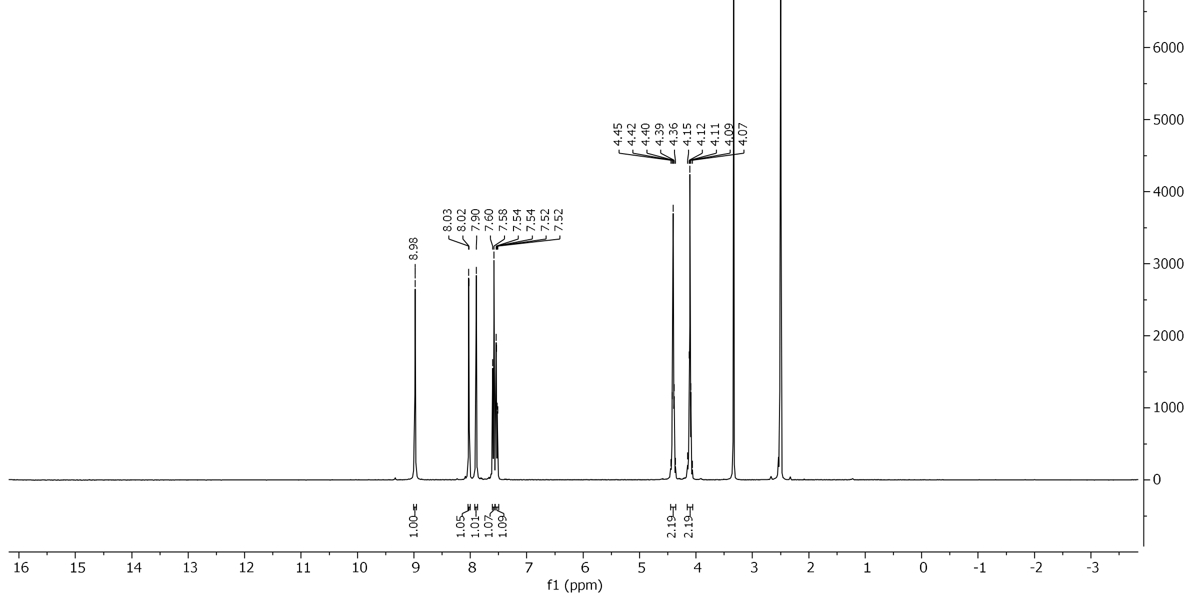

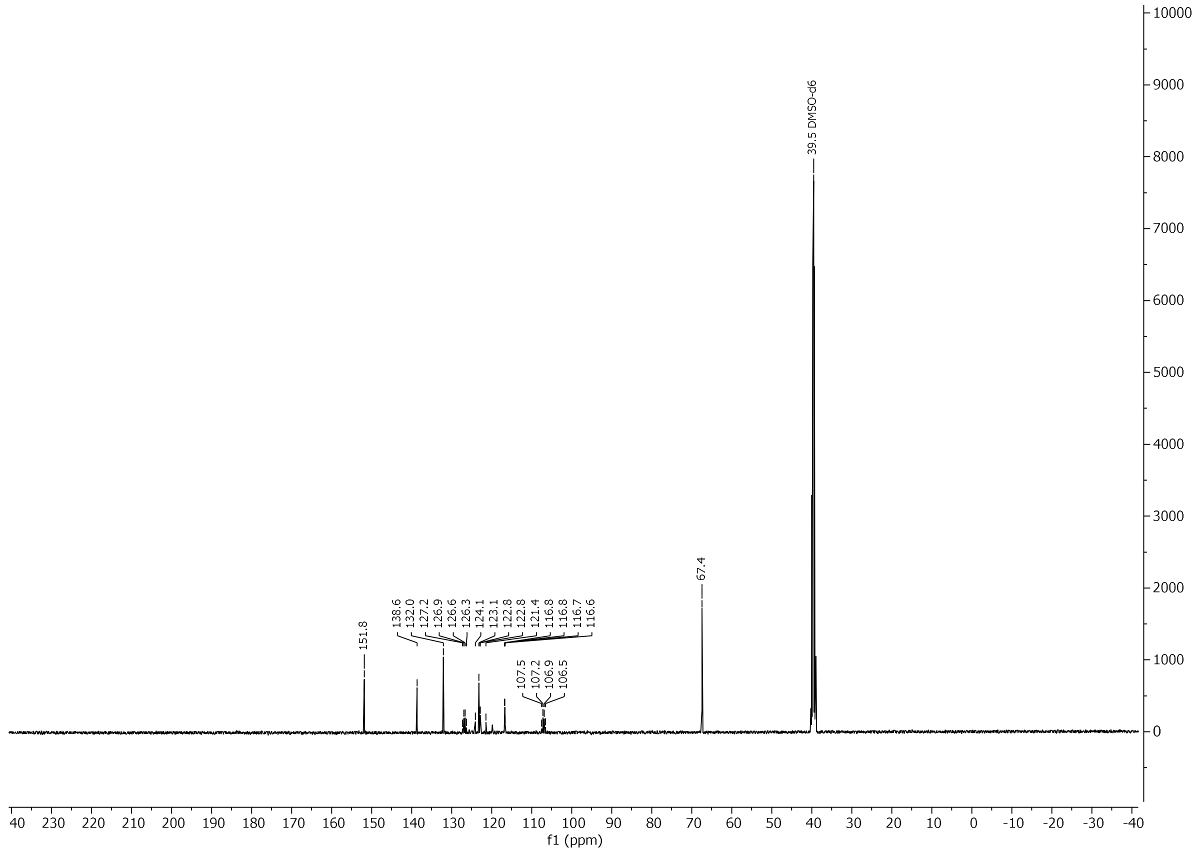

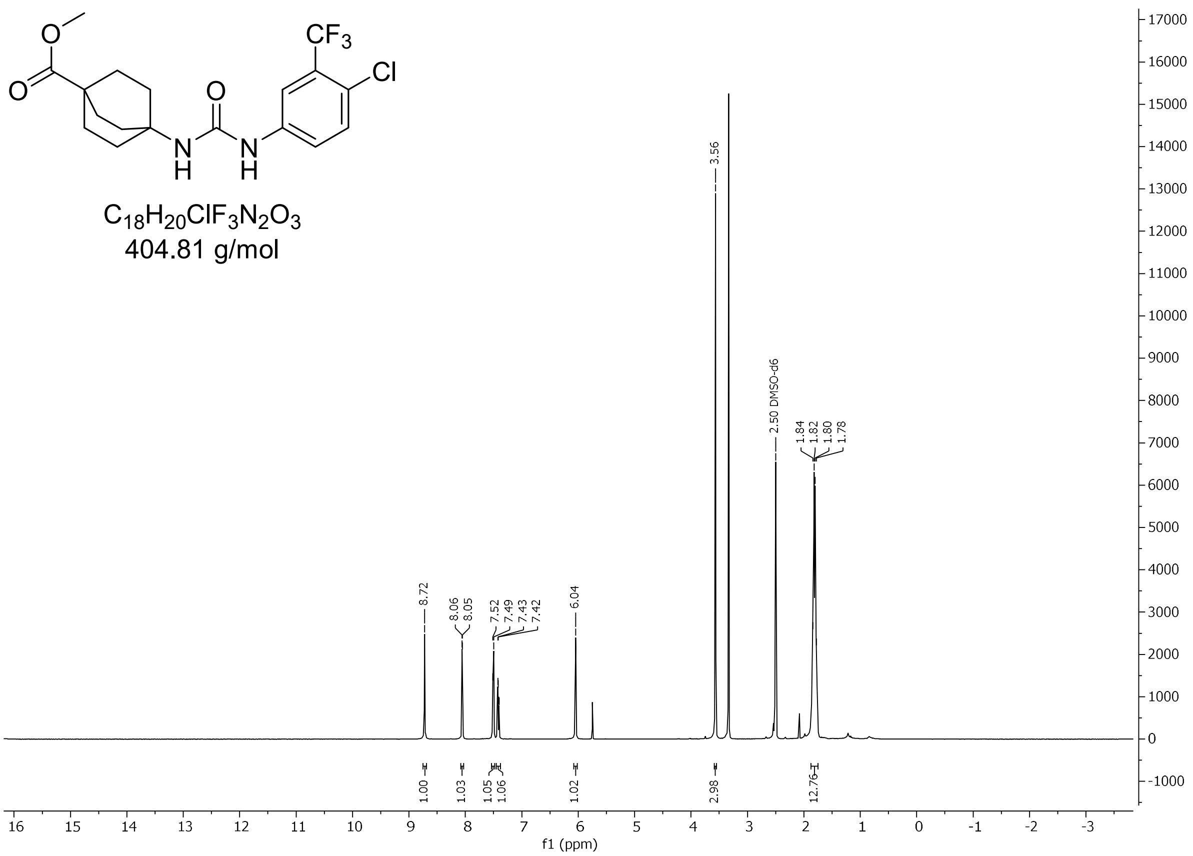
CF3
Cl
O
N
H
C18H20ClF3N2O3
404.81 g/mol
3.56
2.50 DMSO-d6
1.84
1.82
1.80
1.78
8.72
8.06
8.05
7.52
7.49
7.43
7.42
6.04
1.00
1.03
1.05
1.06
1.02
2.98
12.76
f1 (ppm)

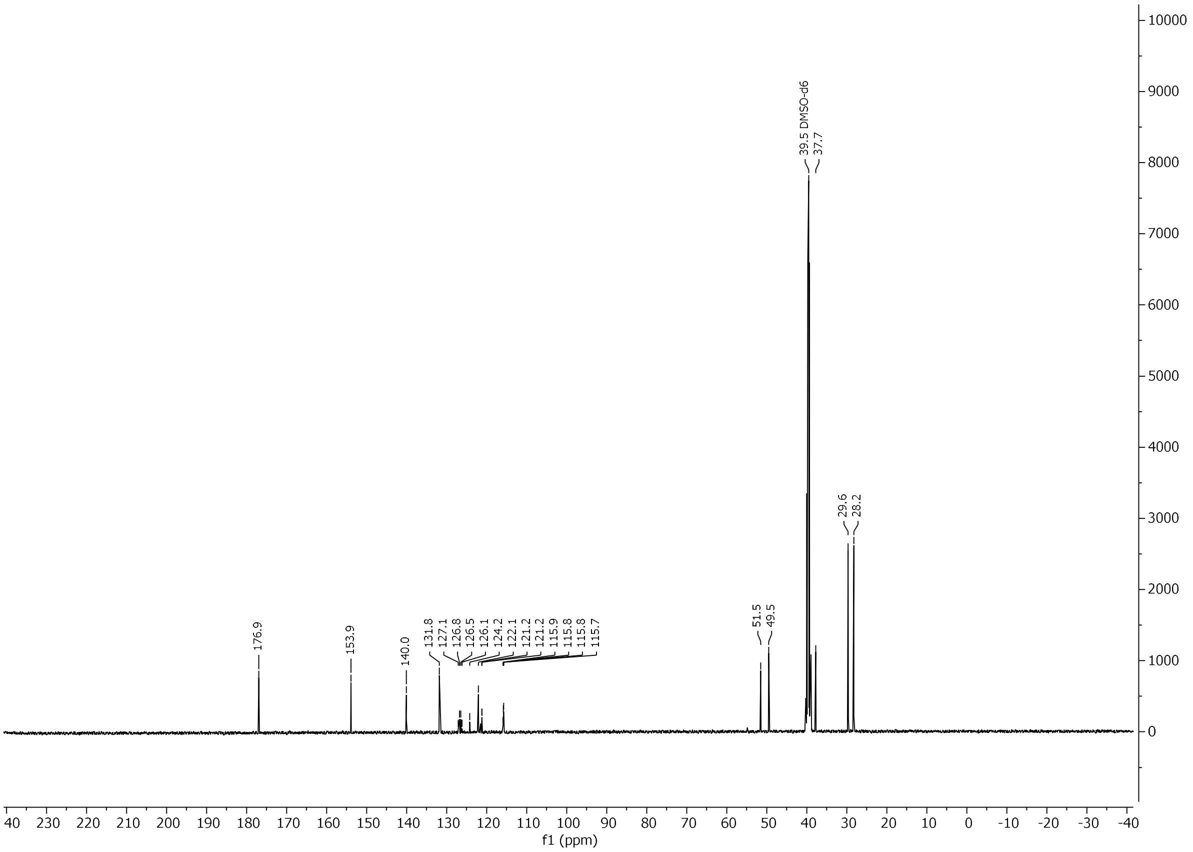
39.5 DMSO-d6
37.7
29.6
28.2
51.5
49.5
176.9
153.9
140.0
131.8
127.1
126.8
126.5
126.1
124.2
122.1
121.2
121.2
115.9
115.8
115.8
115.7
f1 (ppm)

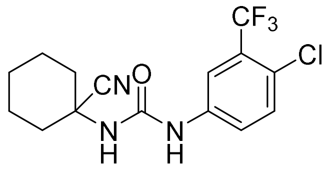

$C_{15}H_{15}ClF_3N_3O$

345.75 g/mol

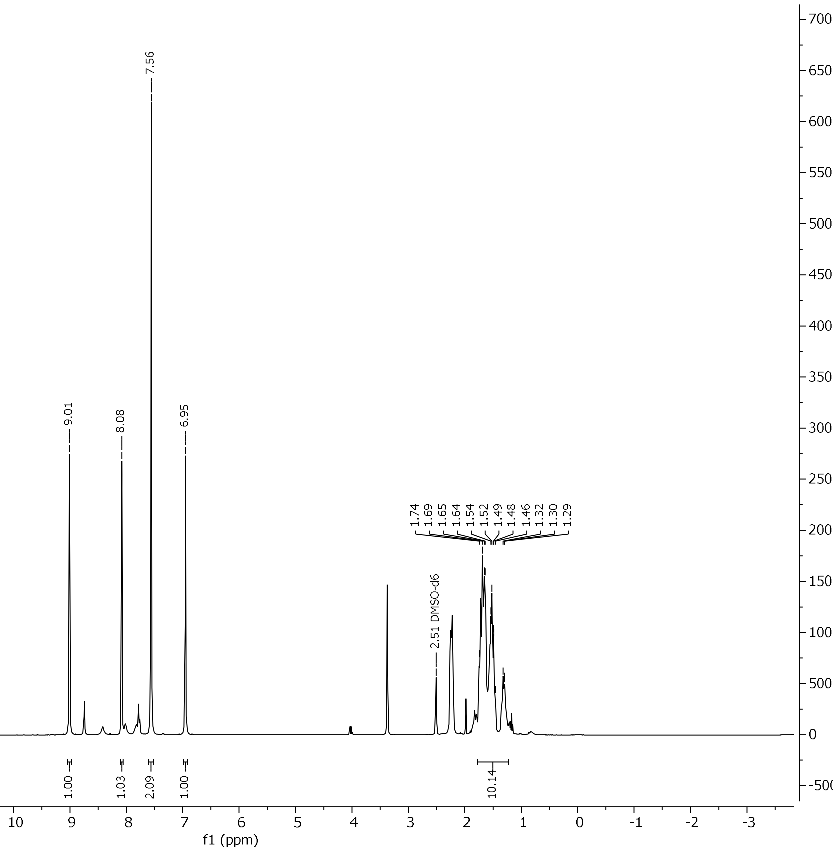

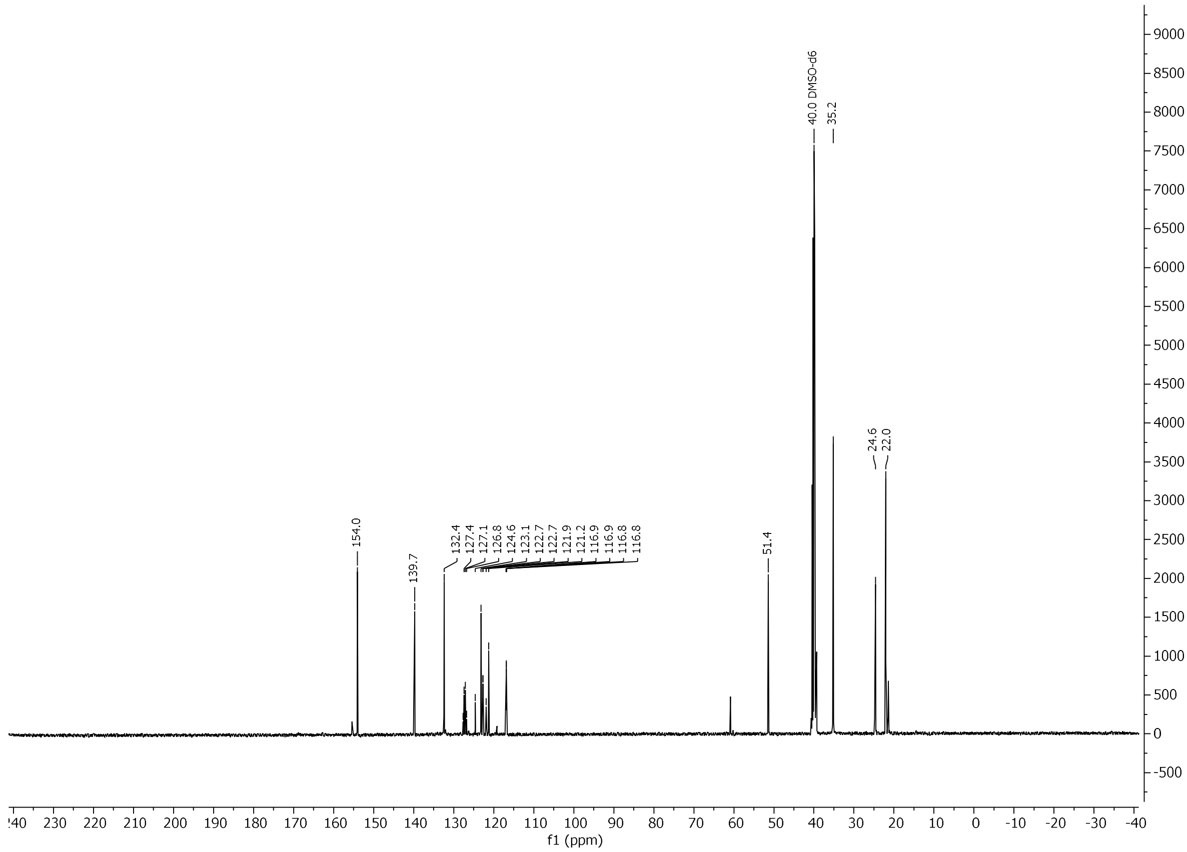

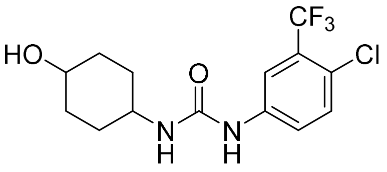

$C_{14}H_{16}ClF_3N_2O_2$
336.74 g/mol

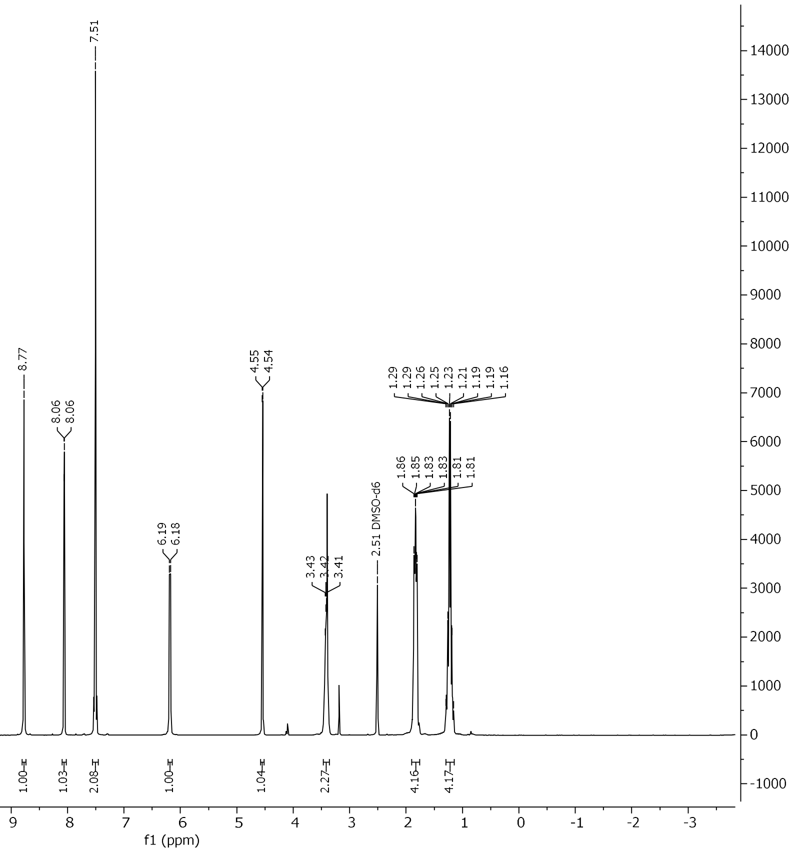

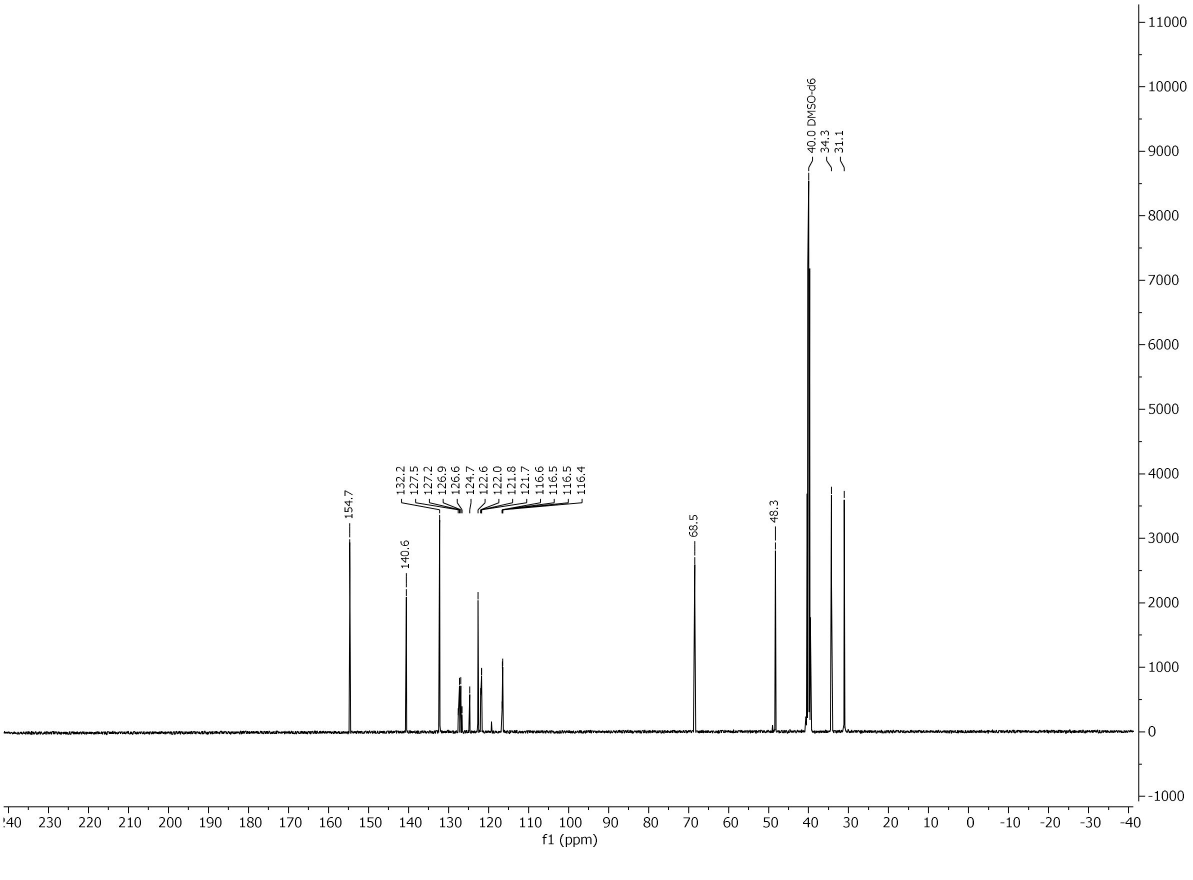

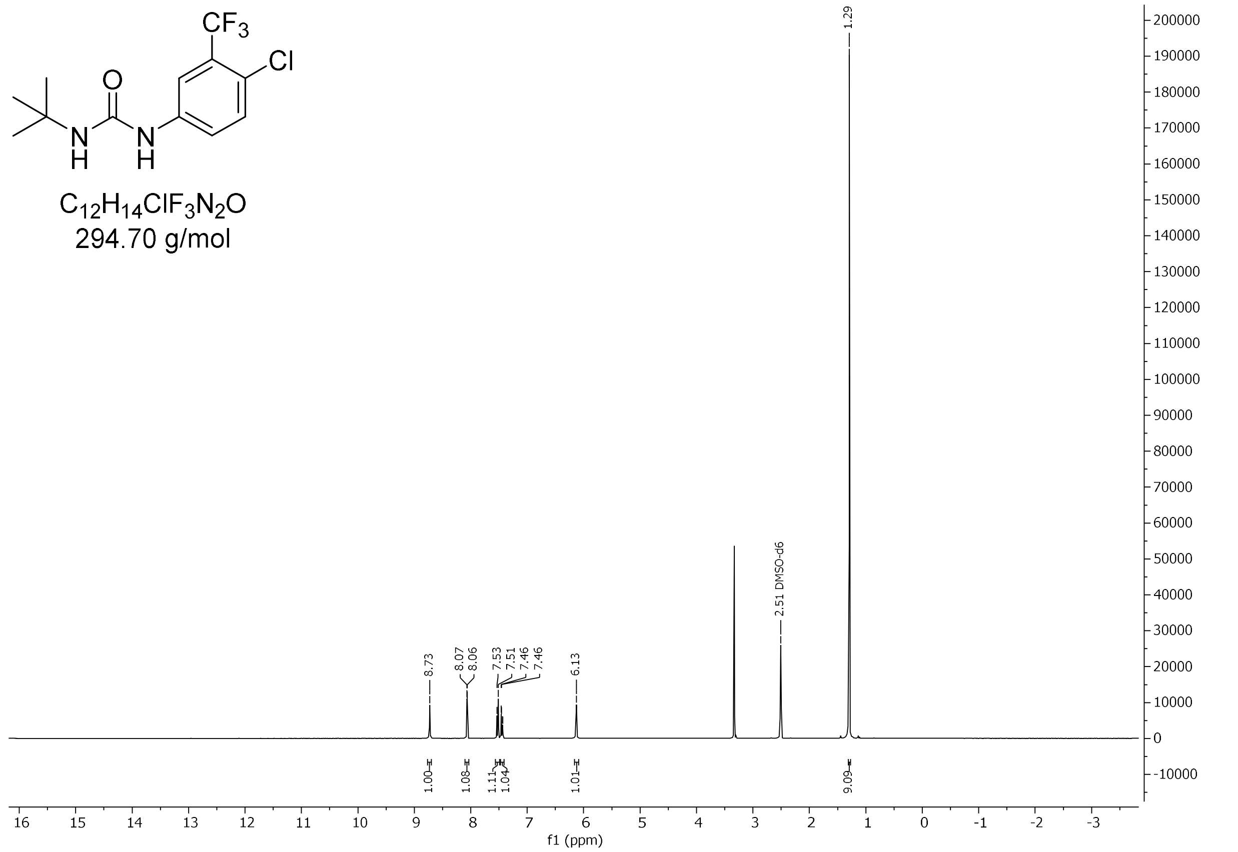
CF3
Cl
O
N
H
N
H
C12H14ClF3N2O
294.70 g/mol
1.29
2.51 DMSO-d6
8.73
8.07
8.06
7.53
7.51
7.46
7.46
6.13
1.00
1.08
1.11
1.04
1.01
9.09
f1 (ppm)

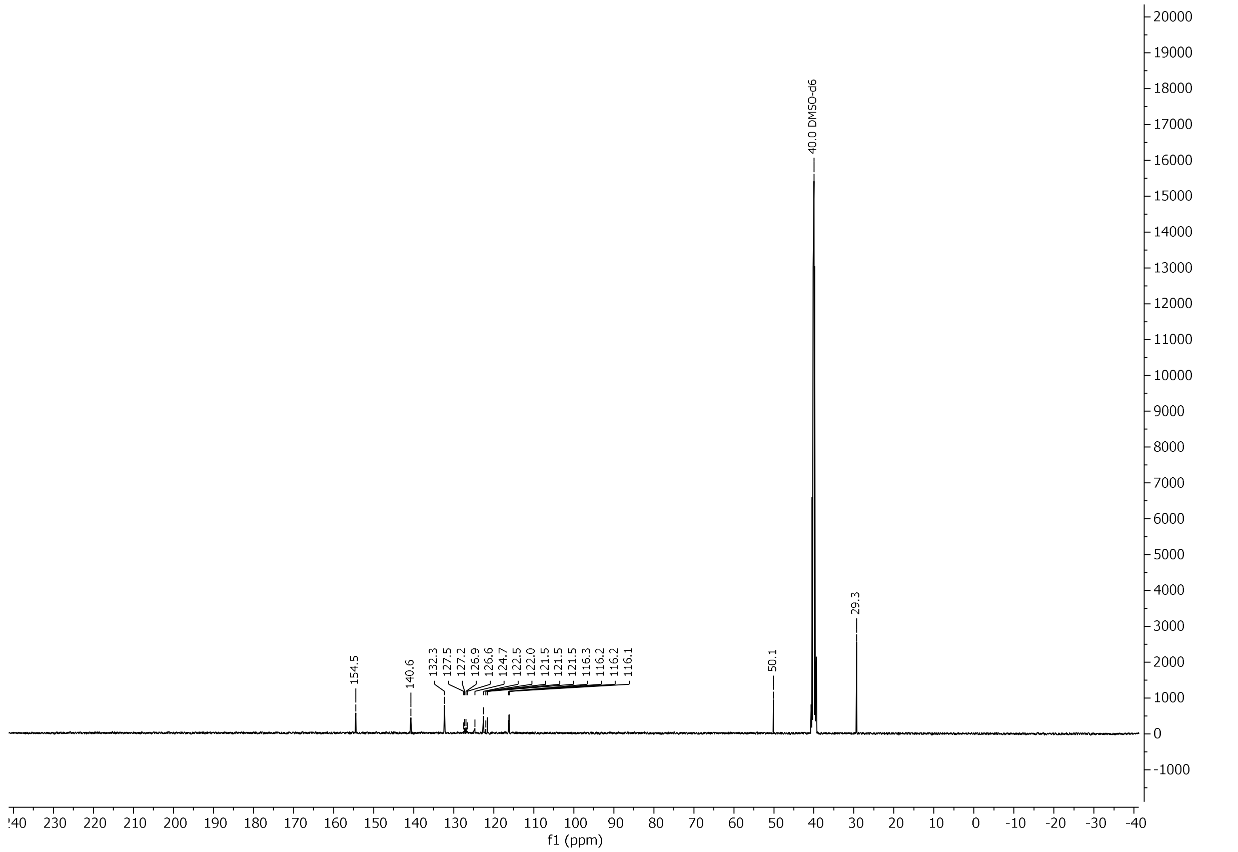
40.0 DMSO-d6
29.3
154.5
140.6
132.3
127.5
127.2
126.9
126.6
124.7
122.5
122.0
121.5
121.5
121.5
116.3
116.2
116.1
50.1
f1 (ppm)

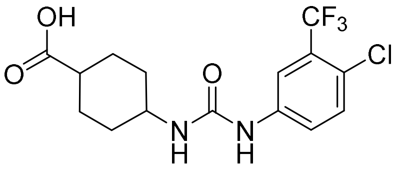

$C_{15}H_{16}ClF_3N_2O_3$
364.75 g/mol

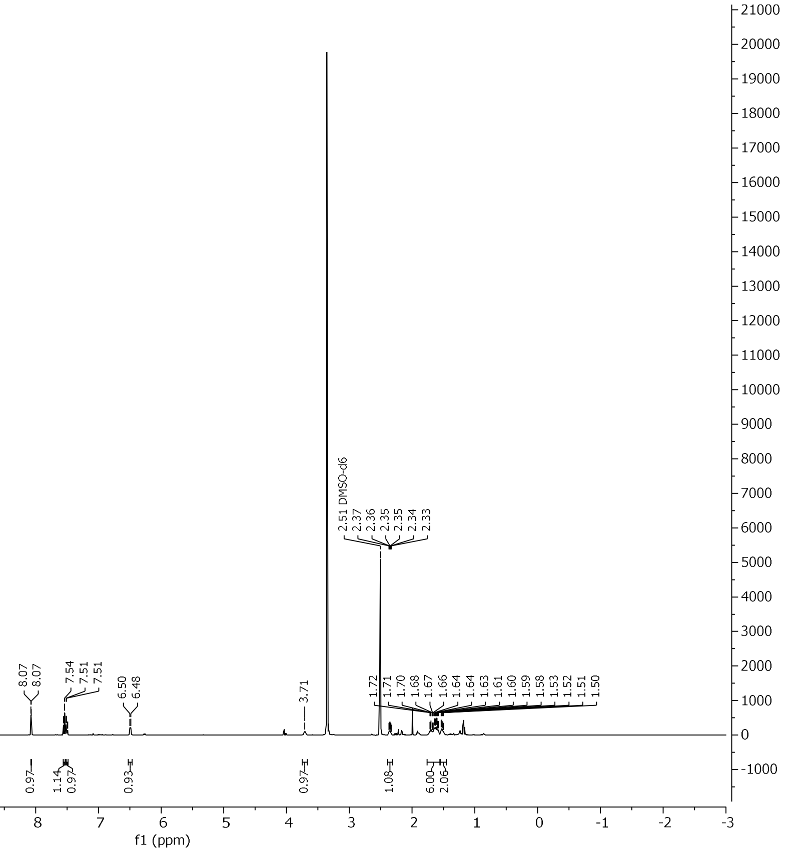

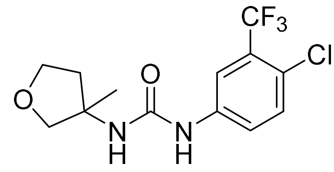

$C_{13}H_{14}ClF_3N_2O_2$
322.71 g/mol

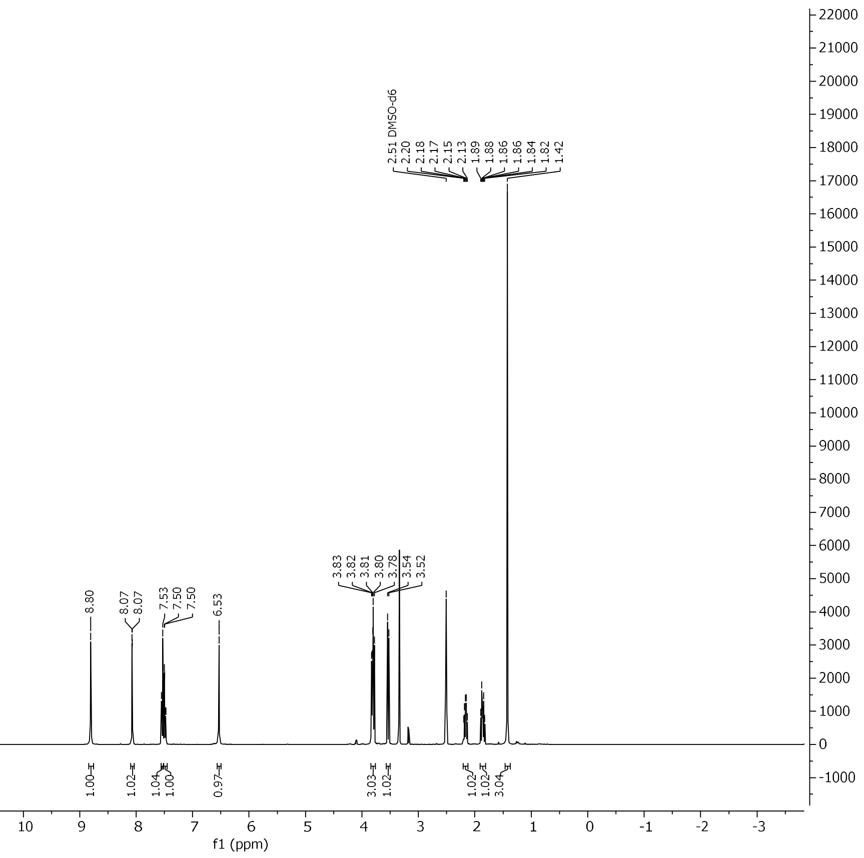

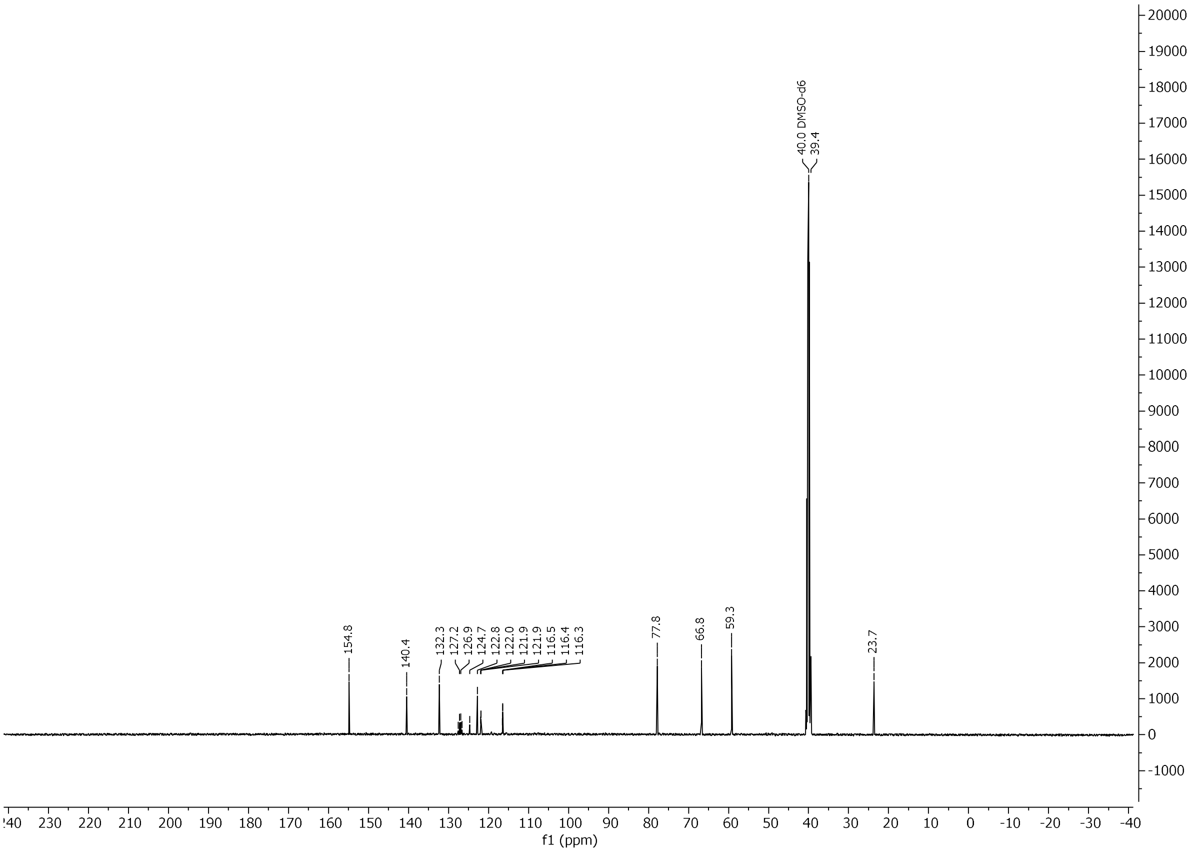

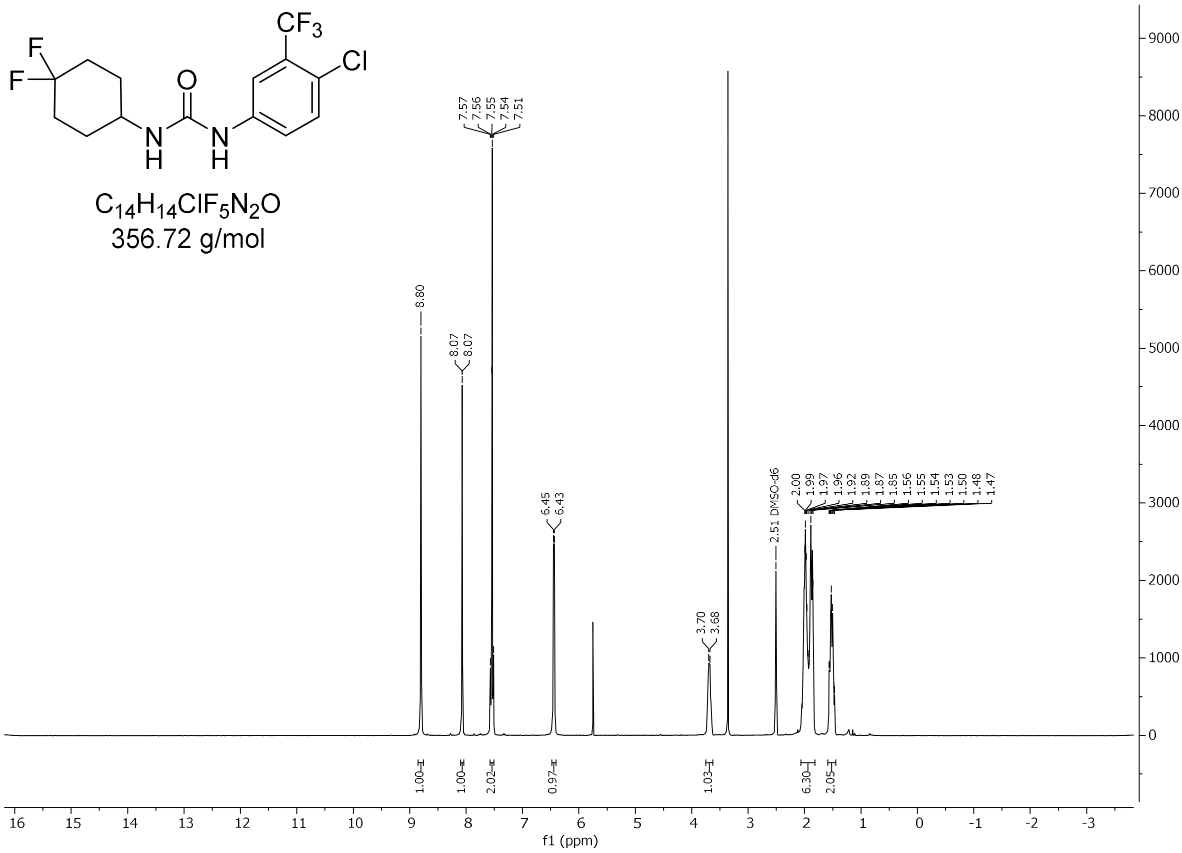
CF3
Cl
F
F
O
N
H
N
H
C14H14ClF5N2O
356.72 g/mol
7.57
7.56
7.55
7.54
7.51
8.80
8.07
8.07
6.45
6.43
3.70
3.68
2.51 DMSO-d6
2.00
1.99
1.97
1.96
1.92
1.89
1.87
1.85
1.56
1.55
1.54
1.53
1.50
1.48
1.47
1.00
1.00
2.02
0.97
1.03
6.30
2.05
f1 (ppm)

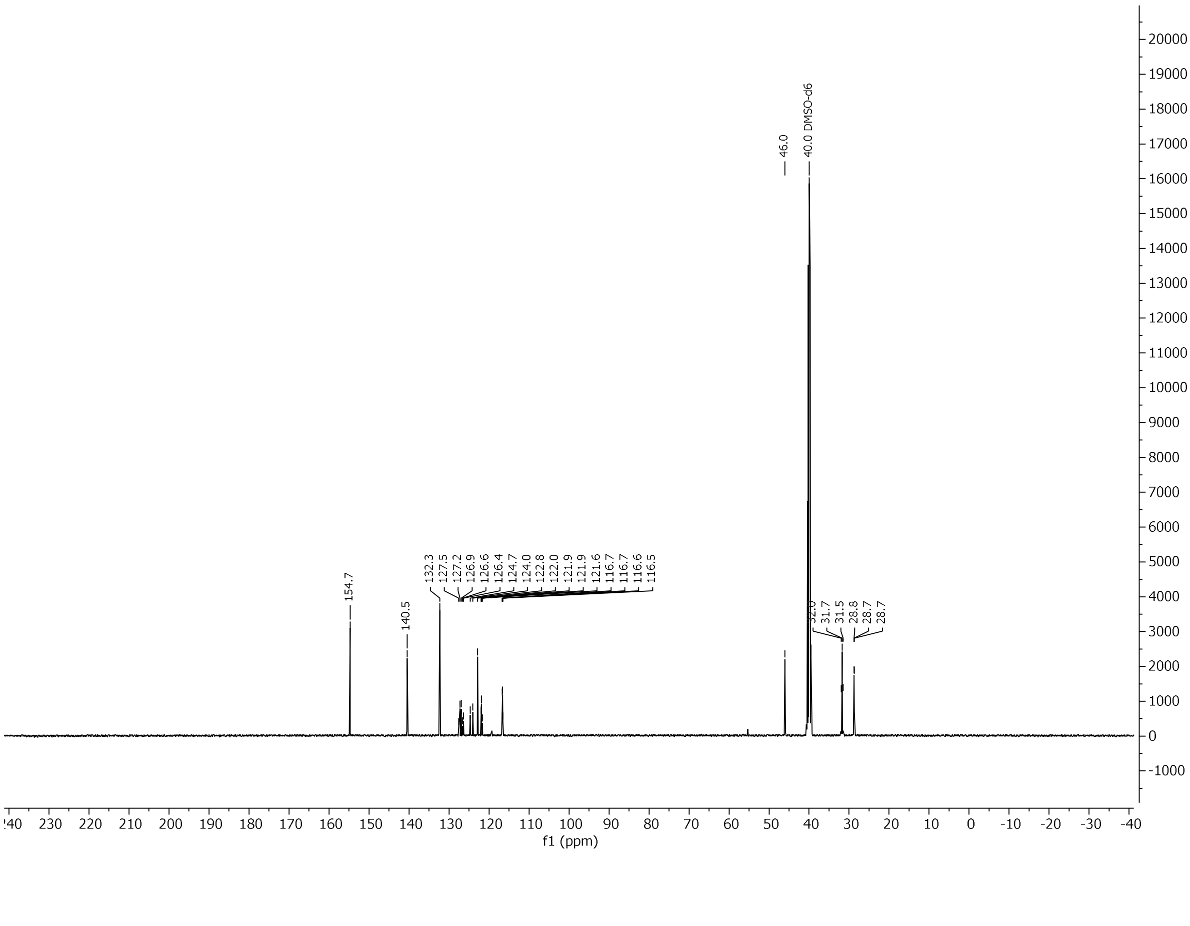
154.7
140.5
132.3
127.5
127.2
126.9
126.6
126.4
124.7
124.0
122.8
122.0
121.9
121.9
121.6
116.7
116.7
116.6
116.5
46.0
40.0 DMSO-d6
32.0
31.7
31.5
28.8
28.7
28.7
f1 (ppm)

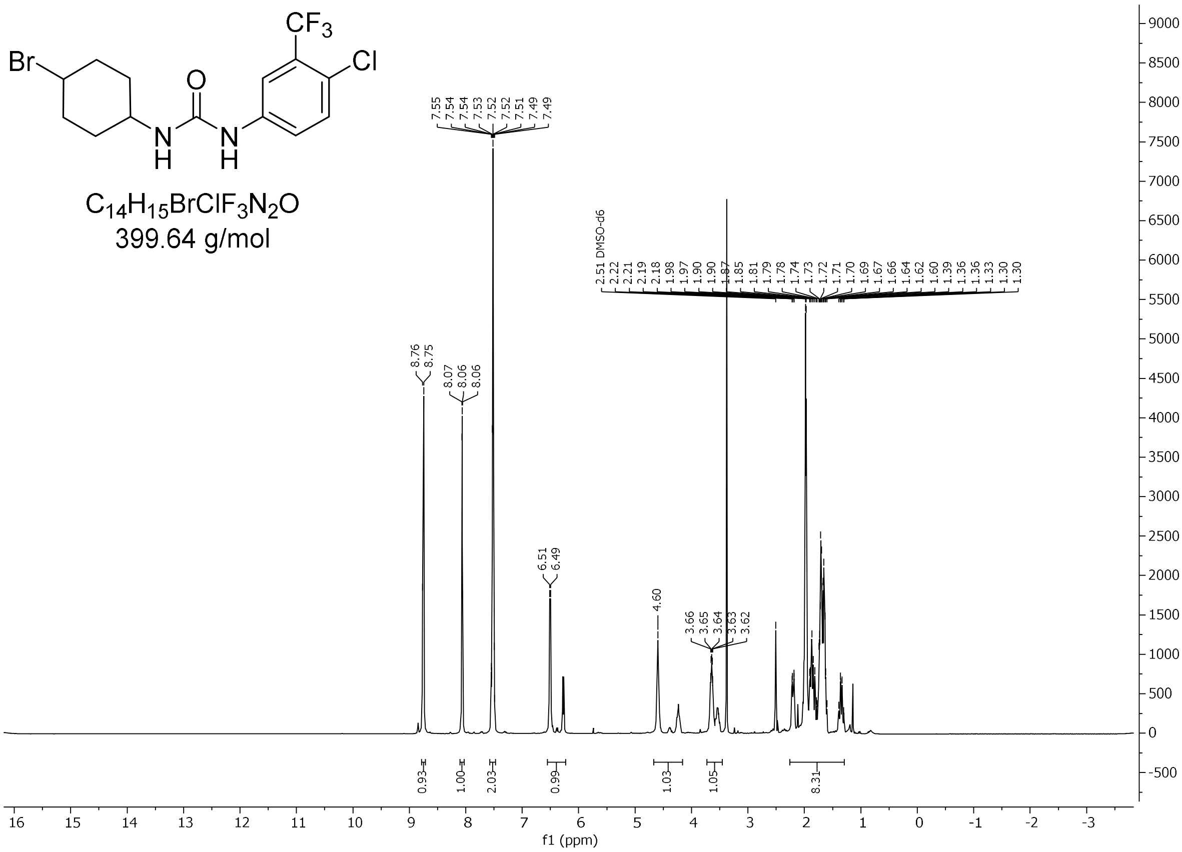
Br
O
N
H
N
H
CF3
Cl
C14H15BrClF3N2O
399.64 g/mol
7.55
7.54
7.54
7.53
7.52
7.52
7.51
7.49
7.49
2.51 DMSO-d6
8.76
8.75
8.07
8.06
8.06
6.51
6.49
4.60
3.66
3.65
3.64
3.63
3.62
0.93
1.00
2.03
0.99
1.03
1.05
8.31
f1 (ppm)

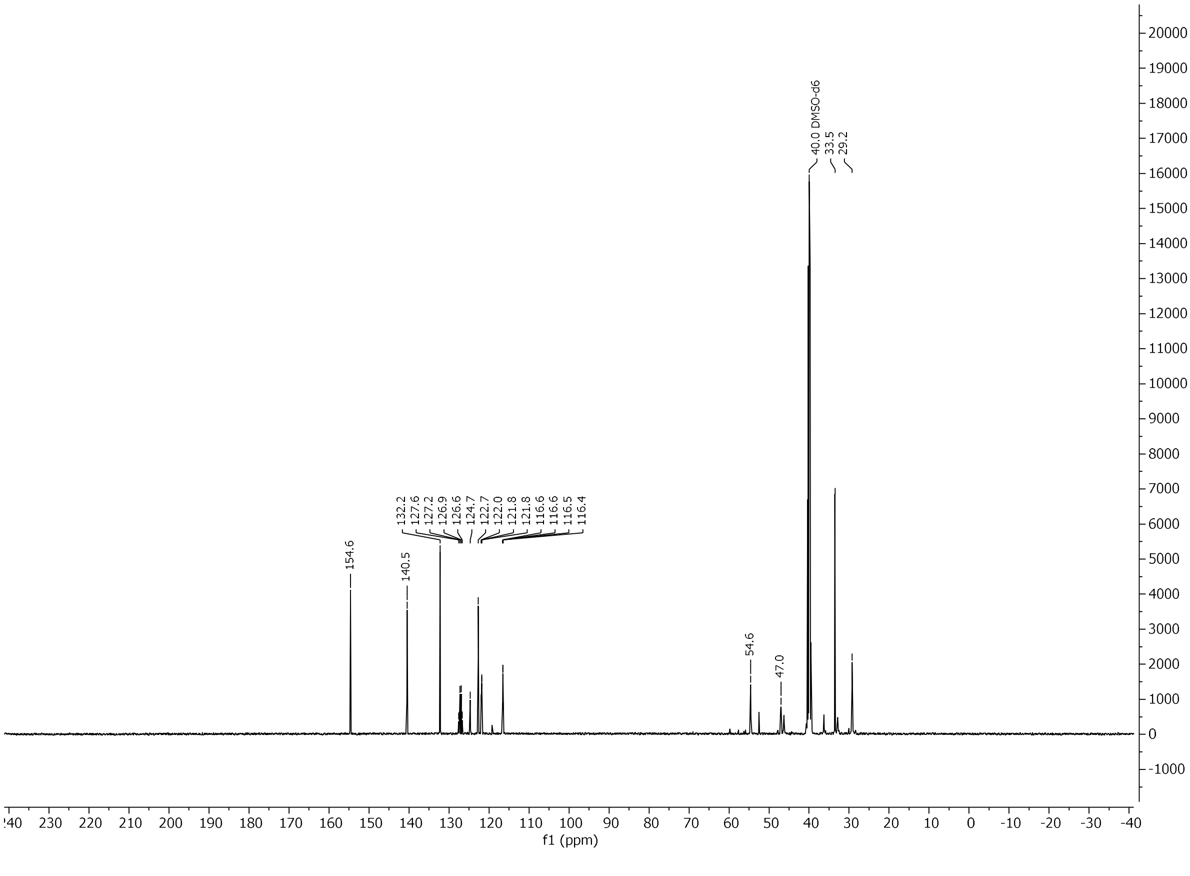
40.0 DMSO-d6
33.5
29.2
154.6
140.5
132.2
127.6
127.2
126.9
126.6
124.7
122.7
122.0
121.8
116.6
116.6
116.5
116.4
54.6
47.0
f1 (ppm)

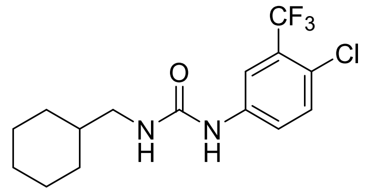

$C_{15}H_{18}ClF_3N_2O$

334.77 g/mol

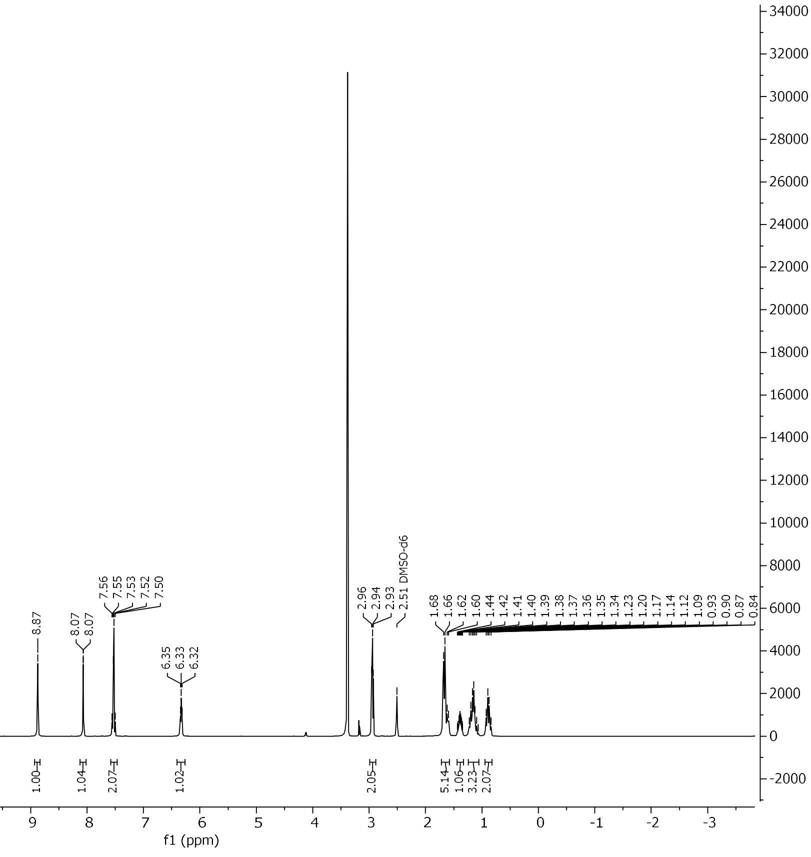

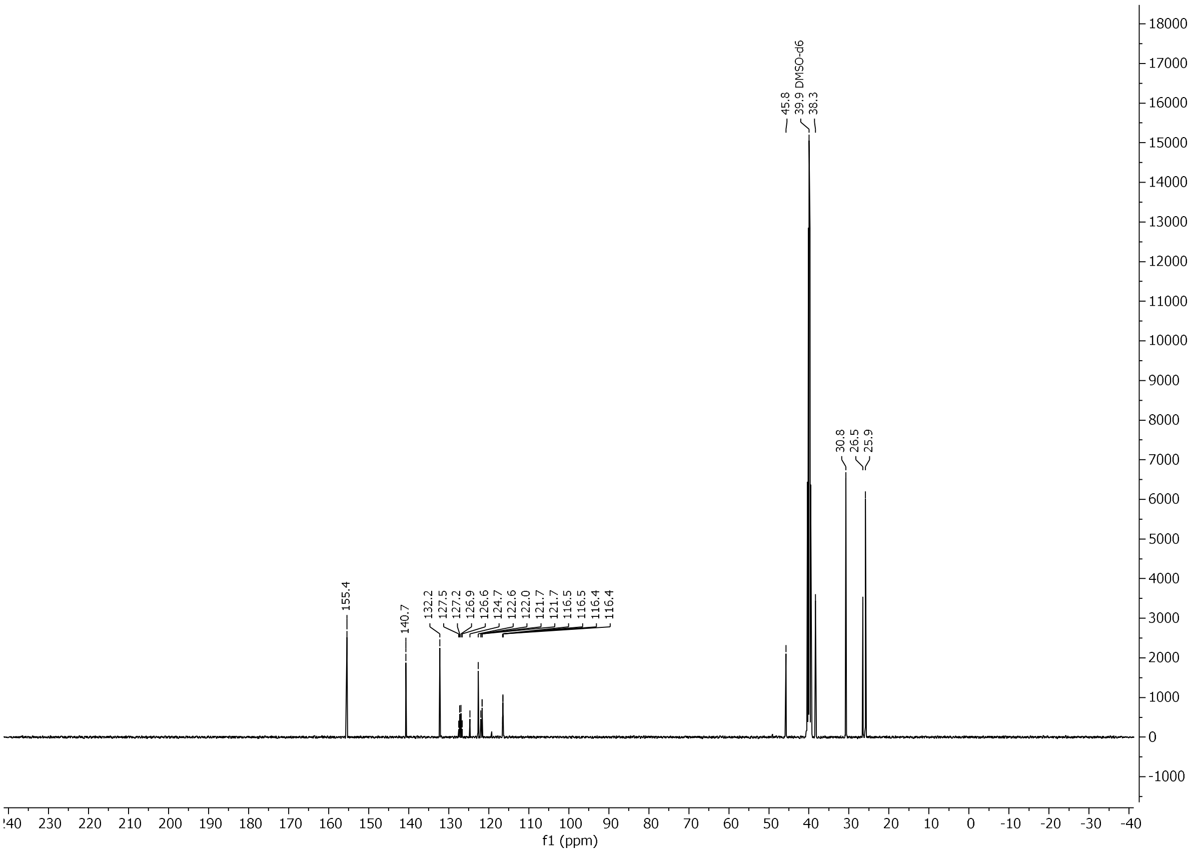

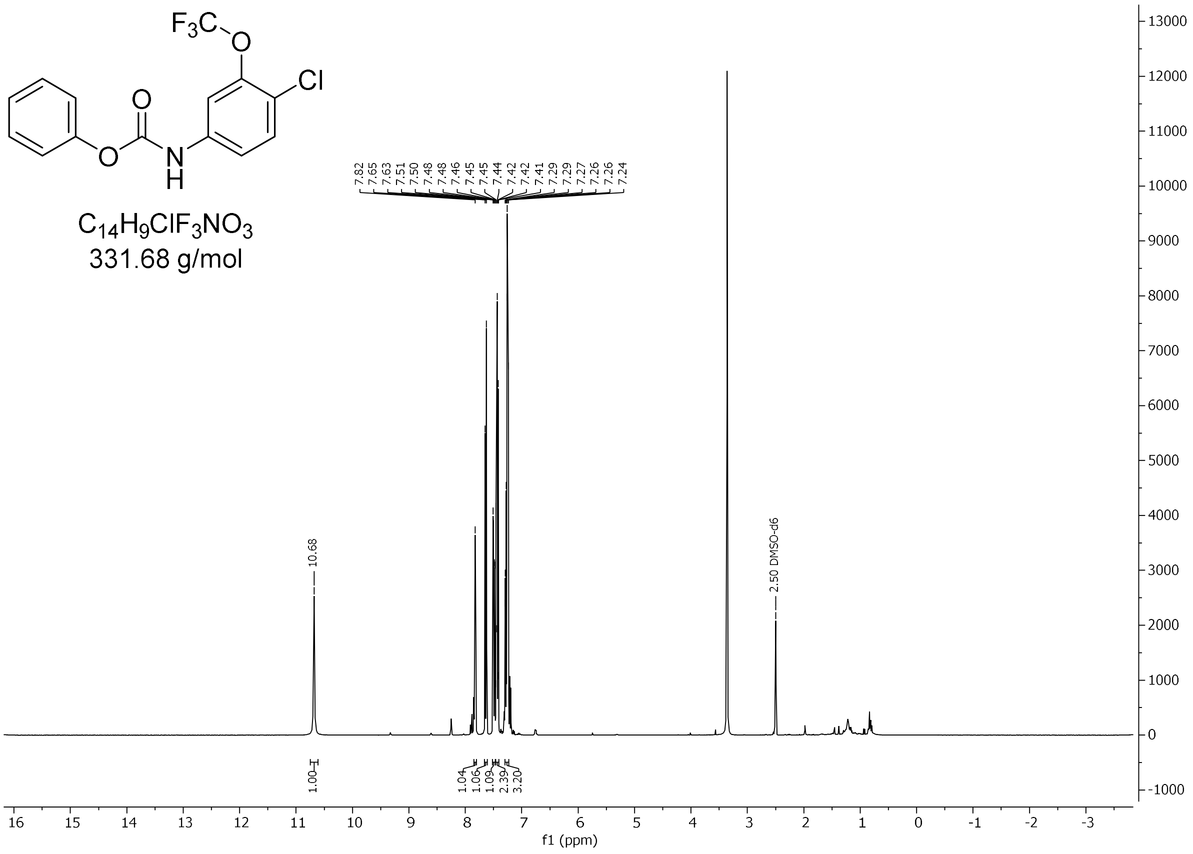
F3C
O
Cl
O
O
N
H
C14H9ClF3NO3
331.68 g/mol
7.82 7.65 7.63 7.51 7.50 7.48 7.48 7.46 7.45 7.45 7.44 7.42 7.42 7.41 7.29 7.29 7.27 7.26 7.26 7.24
10.68
2.50 DMSO-d6
1.00
1.04 1.06 1.09 2.39 3.20
f1 (ppm)

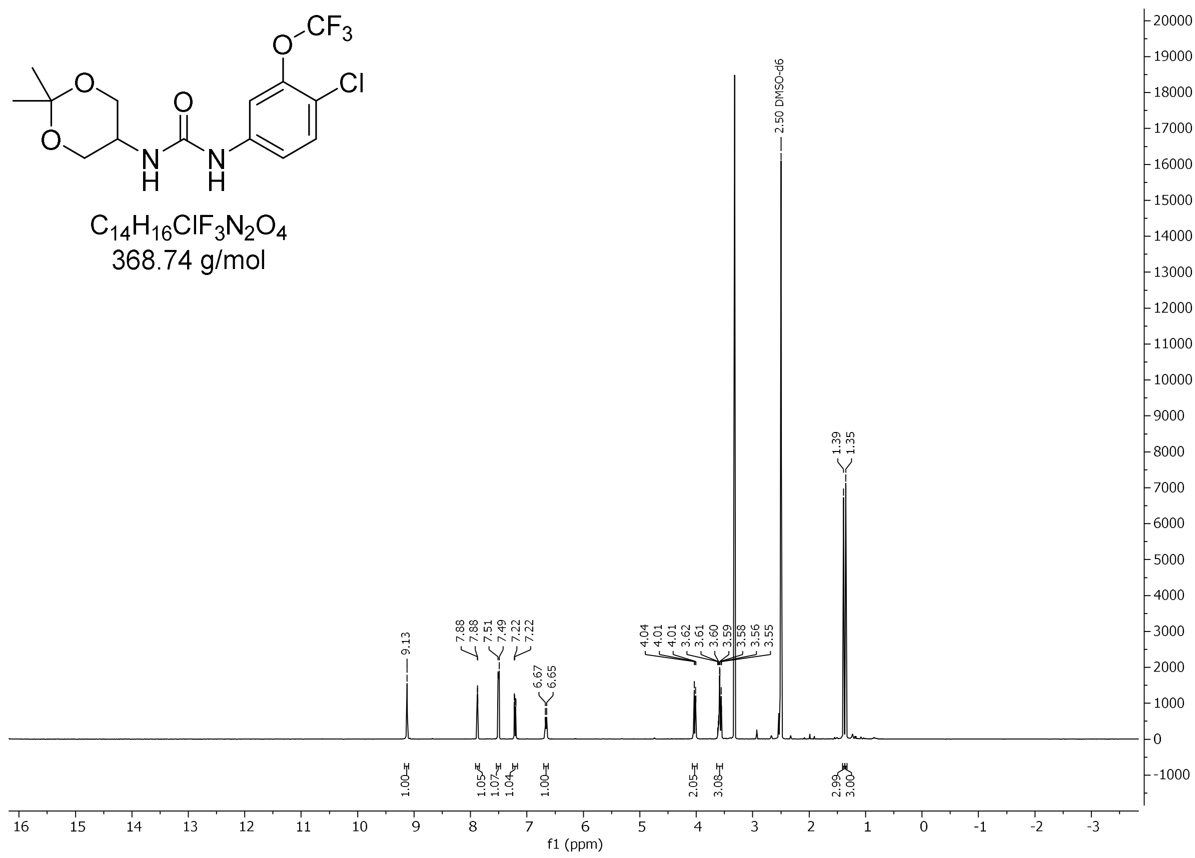

$C_{14}H_{16}ClF_3N_2O_4$
368.74 g/mol
9.13
7.88
7.88
7.51
7.49
7.22
7.22
6.67
6.65
4.04
4.01
4.01
3.62
3.61
3.60
3.59
3.58
3.56
3.55
2.50 DMSO-d6
1.39
1.35
1.00
1.05
1.07
1.04
1.00
2.05
3.08
2.99
3.00
f1 (ppm)

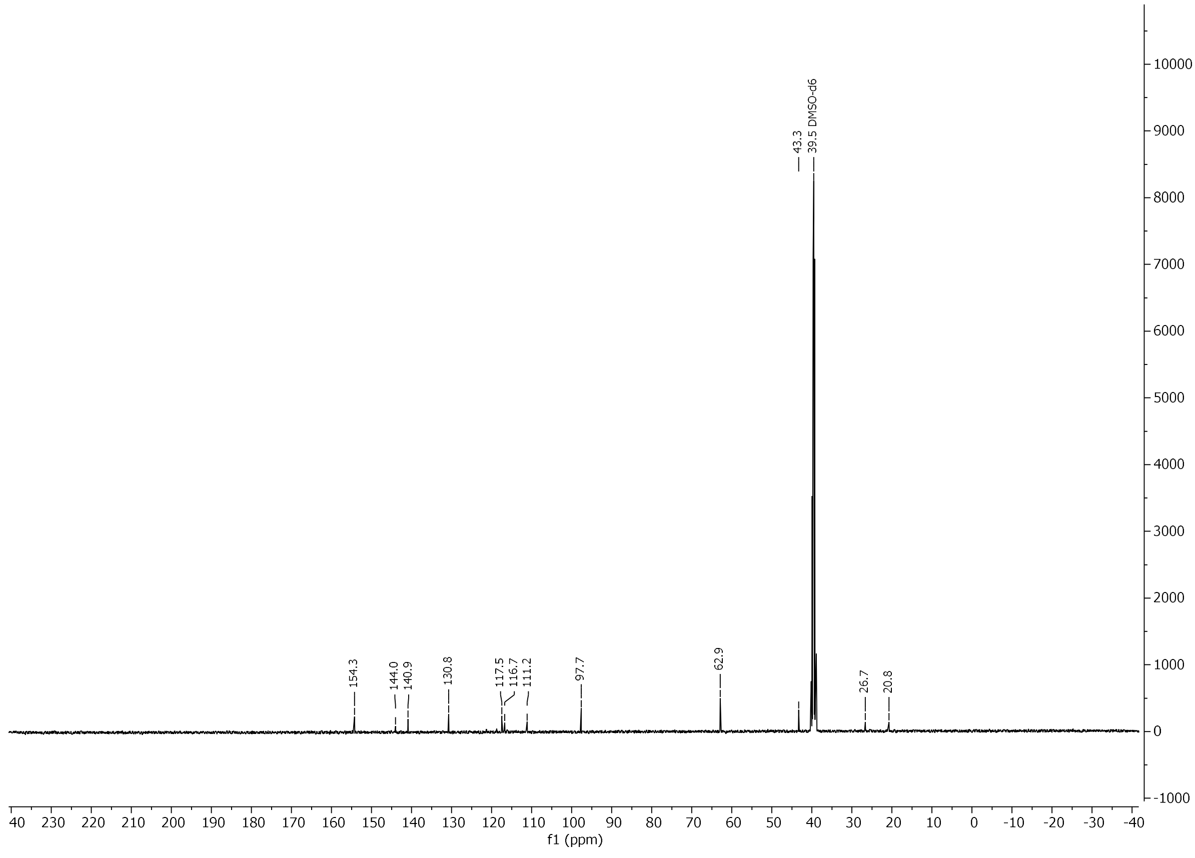

154.3
144.0
140.9
130.8
117.5
116.7
111.2
97.7
62.9
43.3
39.5 DMSO-d6
26.7
20.8
f1 (ppm)

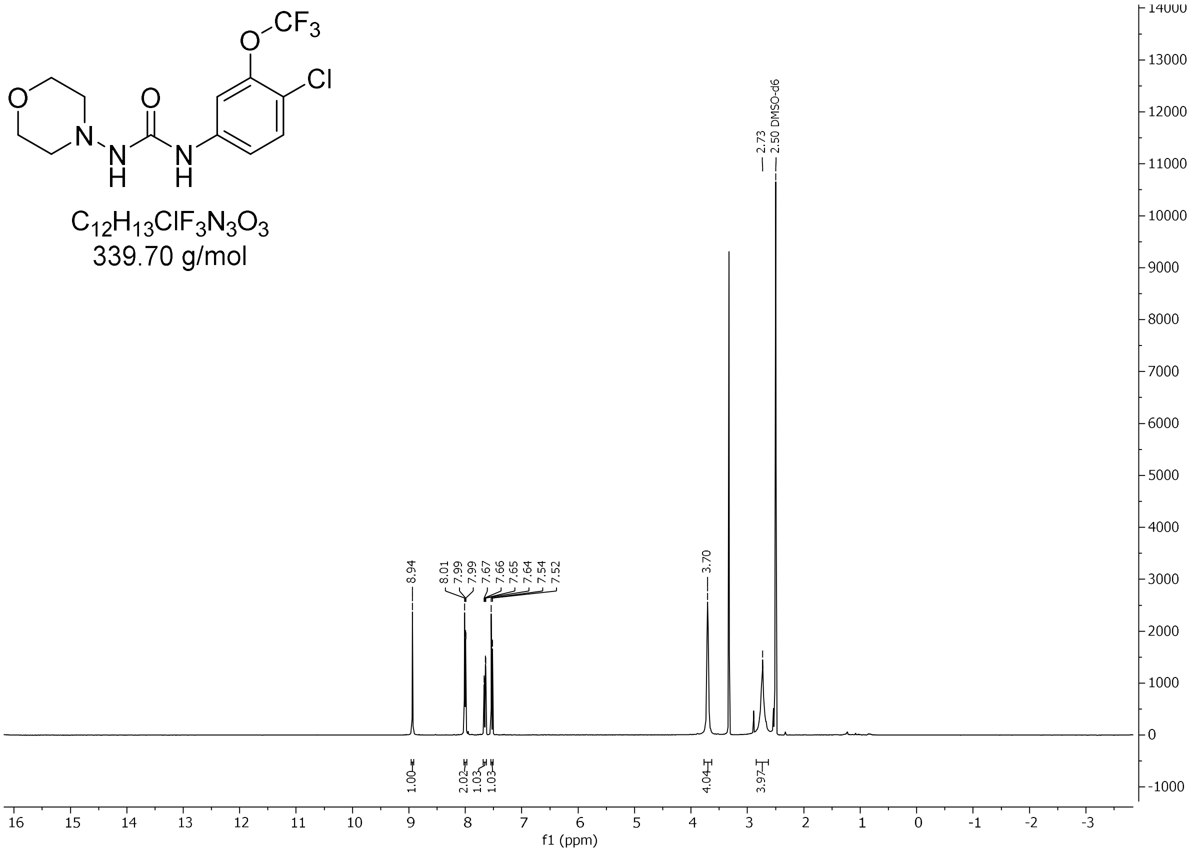
C12H13ClF3N3O3
339.70 g/mol
8.94
8.01
7.99
7.99
7.67
7.66
7.65
7.64
7.54
7.52
3.70
2.73
2.50 DMSO-d6
1.00
2.02
1.03
1.03
4.04
3.97
f1 (ppm)

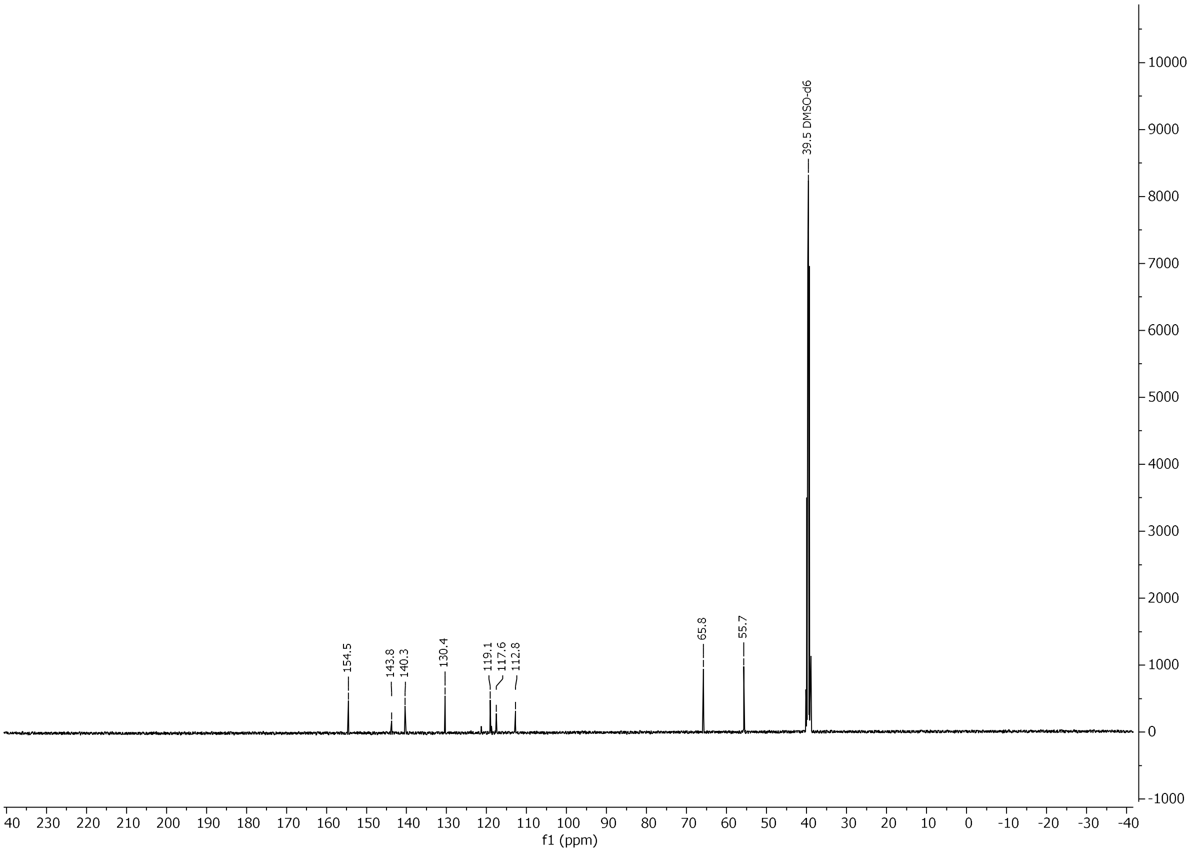
154.5
143.8
140.3
130.4
119.1
117.6
112.8
65.8
55.7
39.5 DMSO-d6
f1 (ppm)

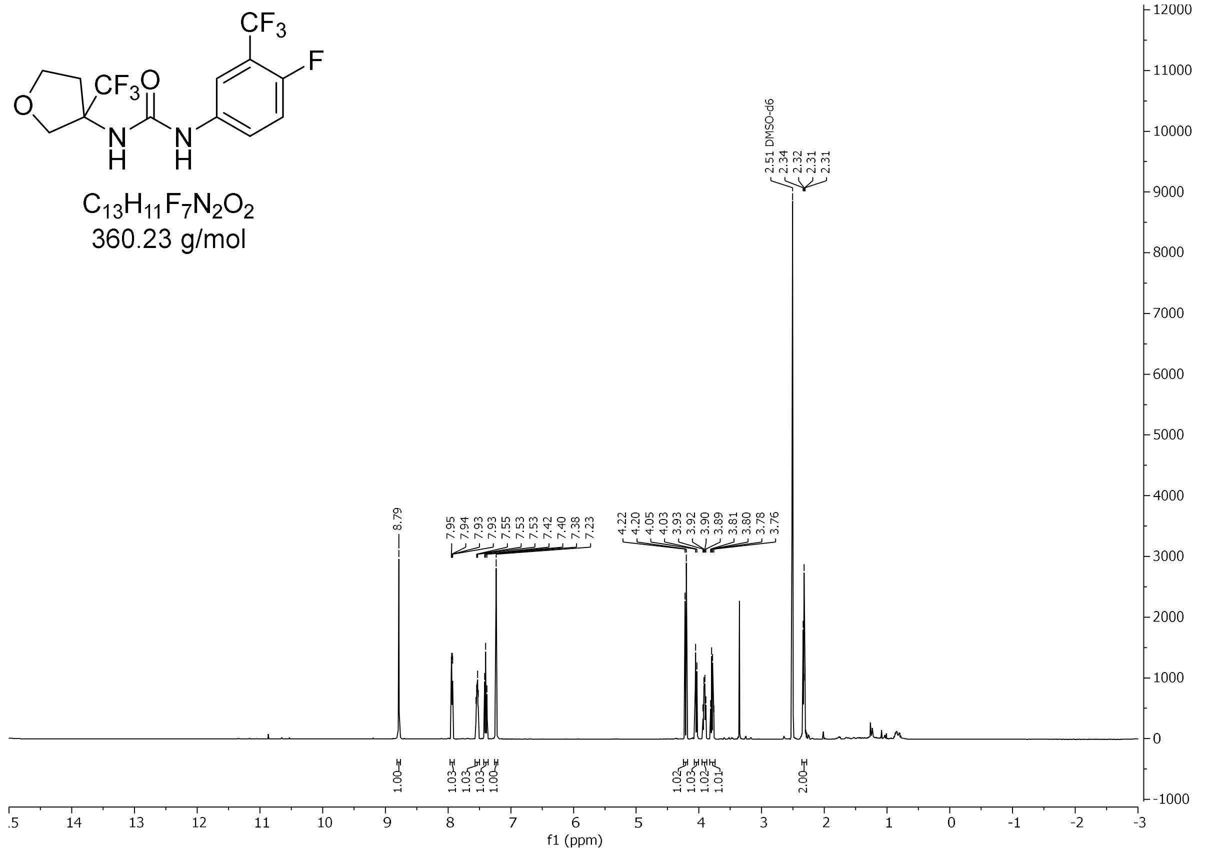
CF3
F
CF3O
O
N
H
N
H
C13H11F7N2O2
360.23 g/mol
2.51 DMSO-d6
2.34
2.32
2.31
2.31
8.79
7.95
7.94
7.93
7.93
7.55
7.53
7.53
7.42
7.40
7.38
7.23
4.22
4.20
4.05
4.03
3.93
3.92
3.90
3.89
3.81
3.80
3.78
3.76
1.00
1.03
1.03
1.03
1.00
1.02
1.03
1.02
1.01
2.00
f1 (ppm)

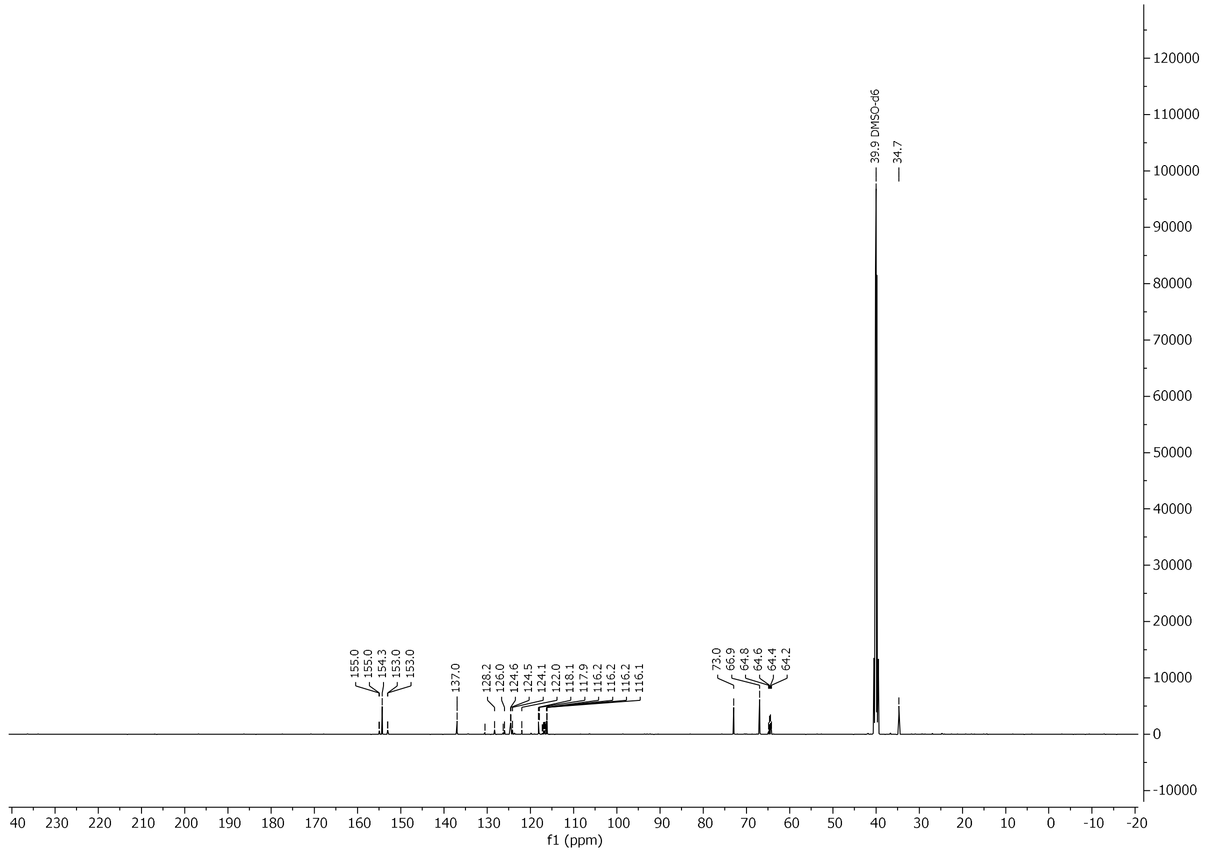
39.9 DMSO-d6
34.7
155.0
155.0
154.3
153.0
153.0
137.0
128.2
126.0
124.6
124.5
124.1
122.0
118.1
117.9
116.2
116.2
116.2
116.1
73.0
66.9
64.8
64.6
64.4
64.2
f1 (ppm)

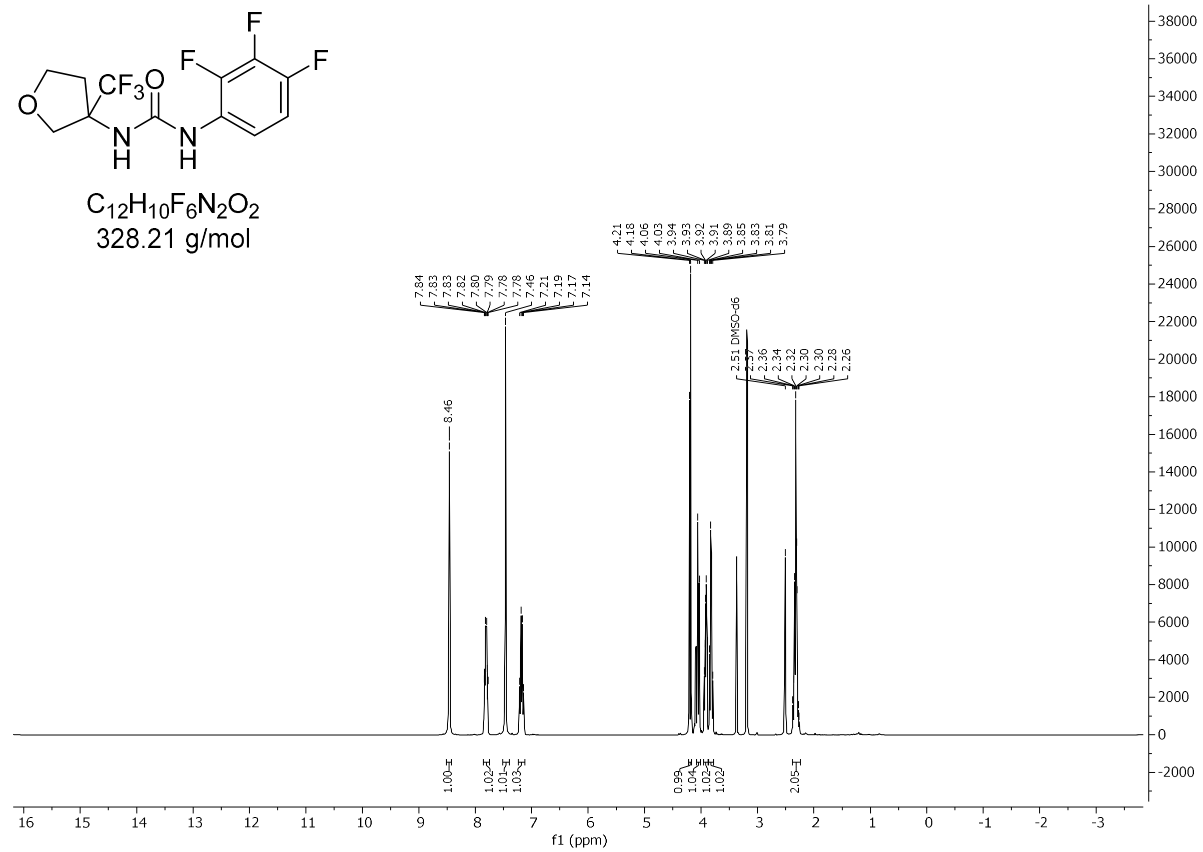
C12H10F6N2O2
328.21 g/mol
f1 (ppm)

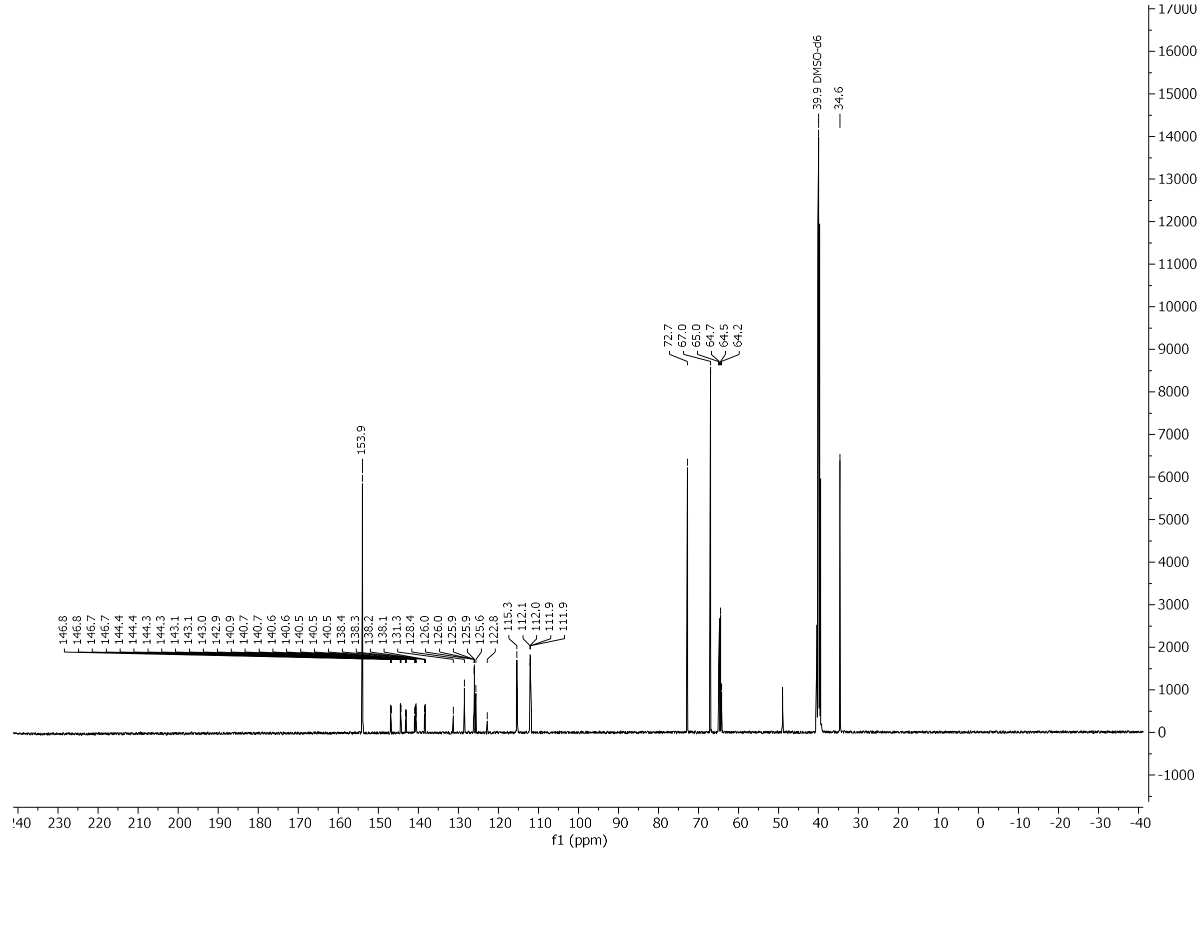
f1 (ppm)

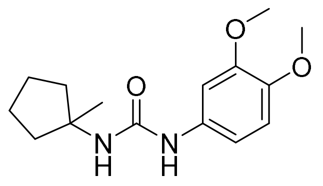

$C_{15}H_{22}N_2O_3$
278.35 g/mol

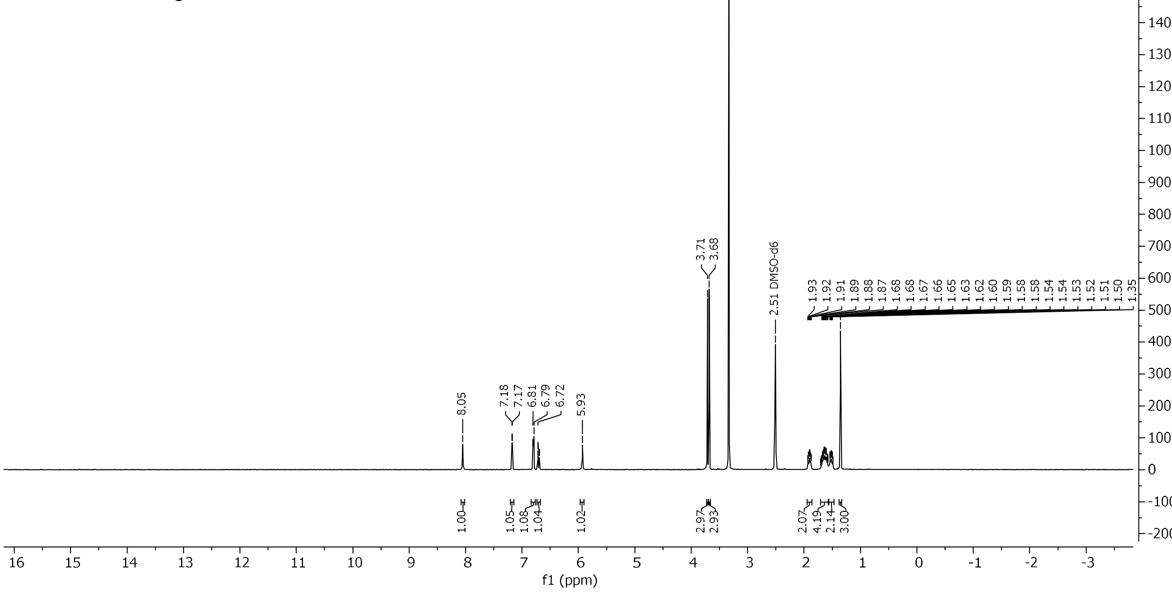

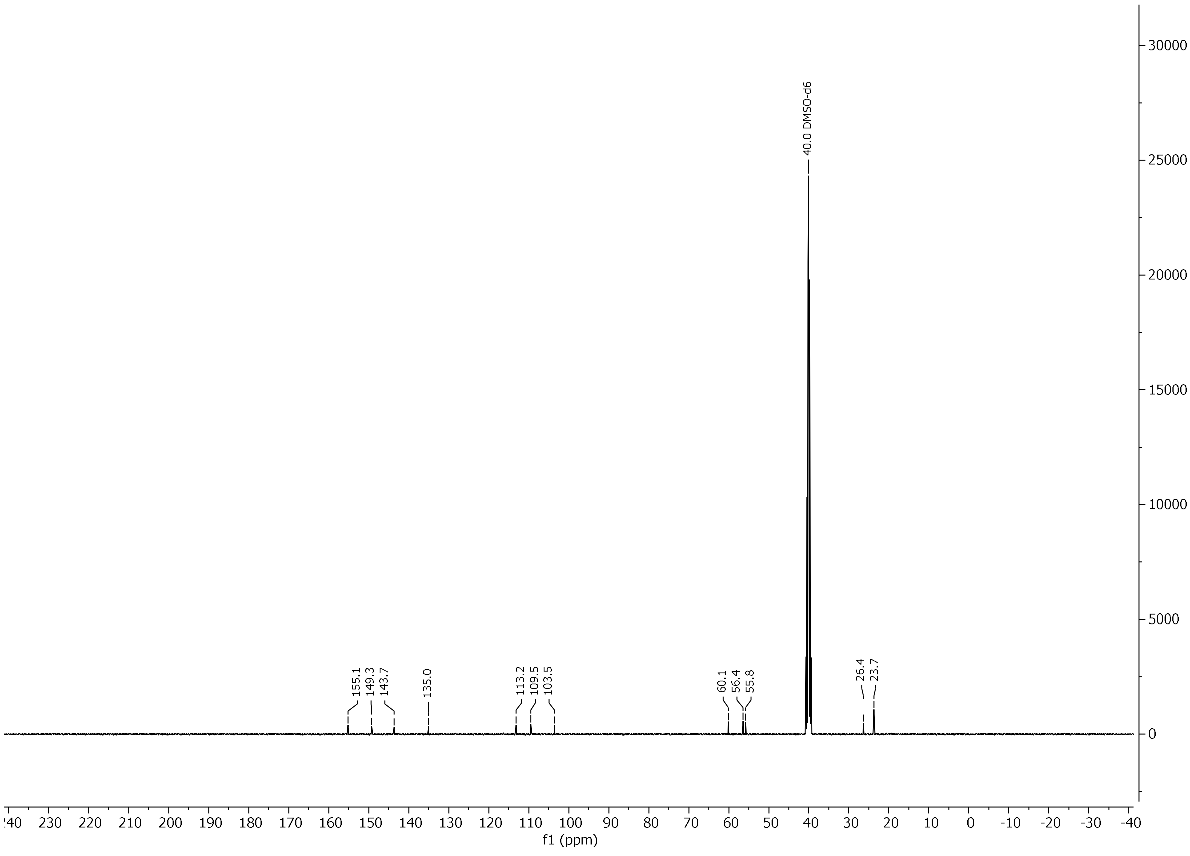

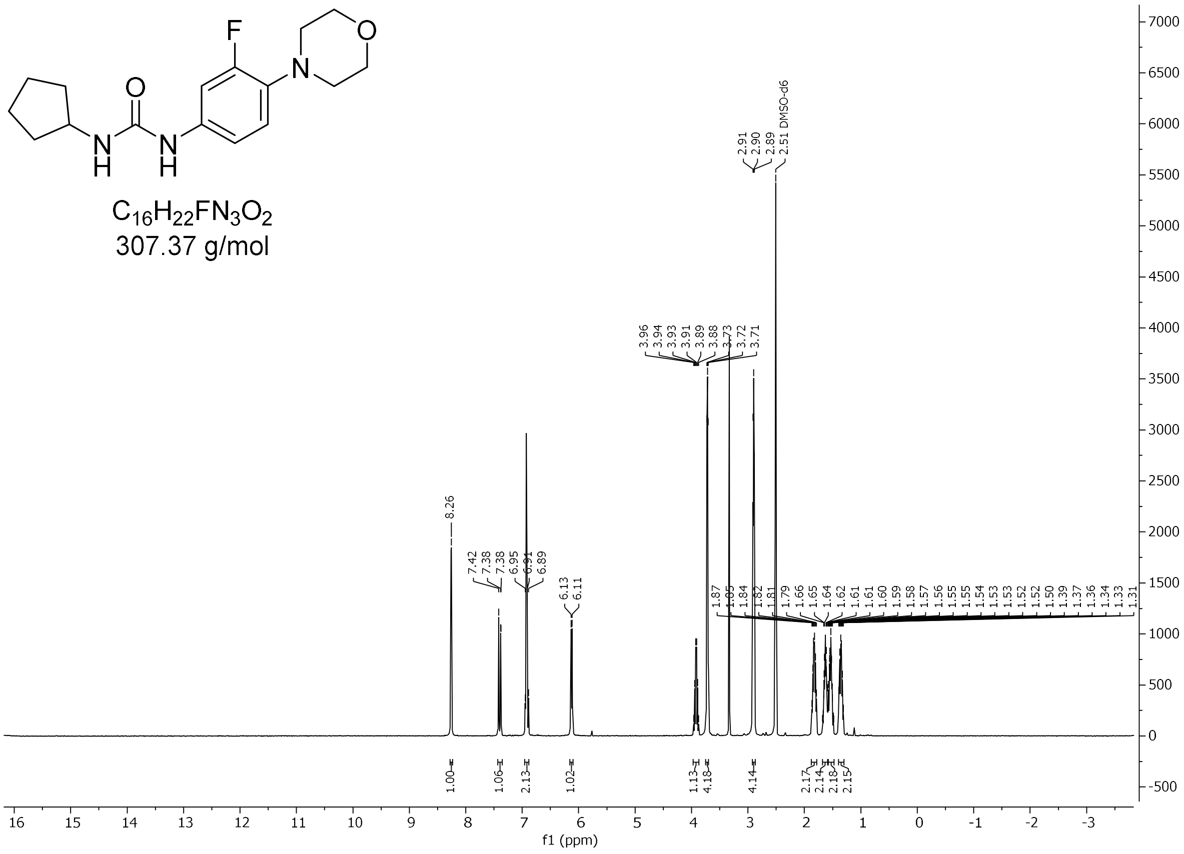

$C_{16}H_{22}FN_3O_2$
307.37 g/mol

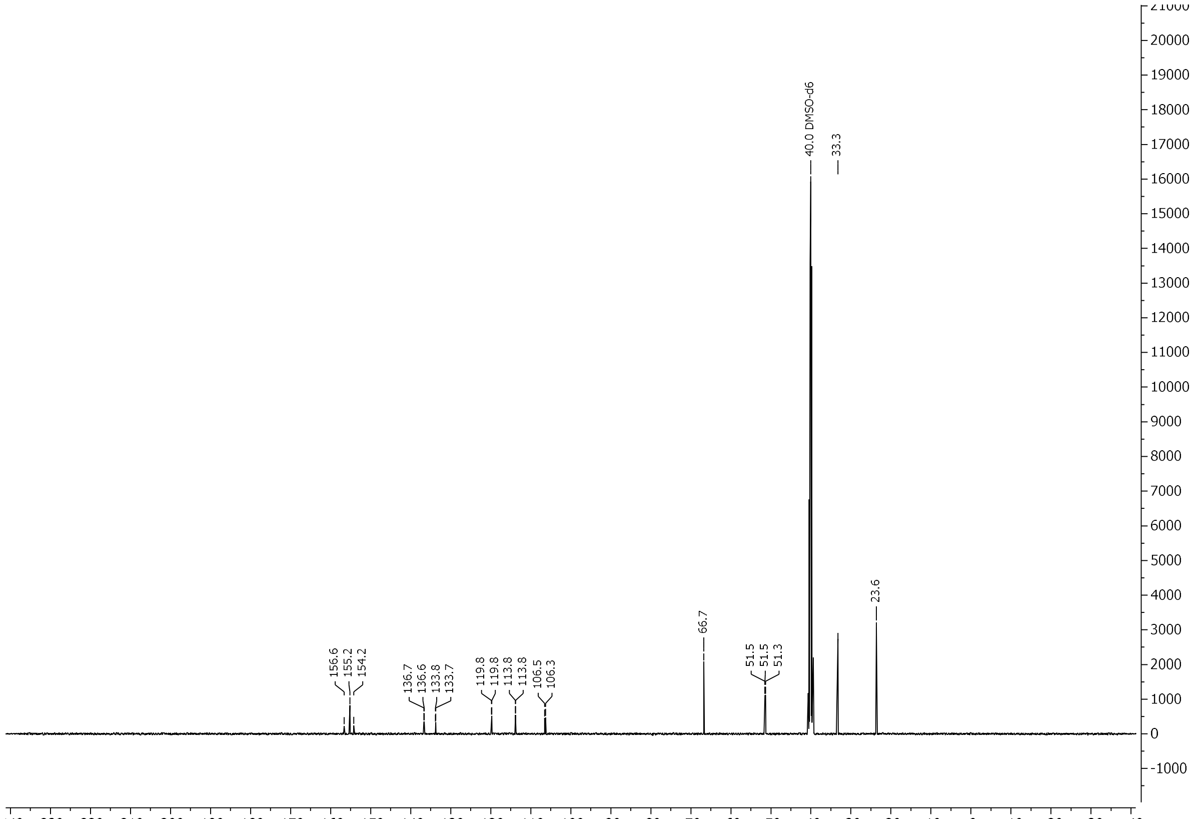

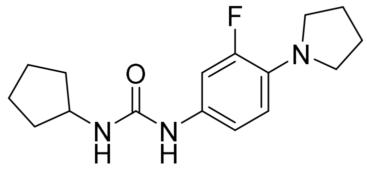

$C_{16}H_{22}FN_3O$
291.37 g/mol

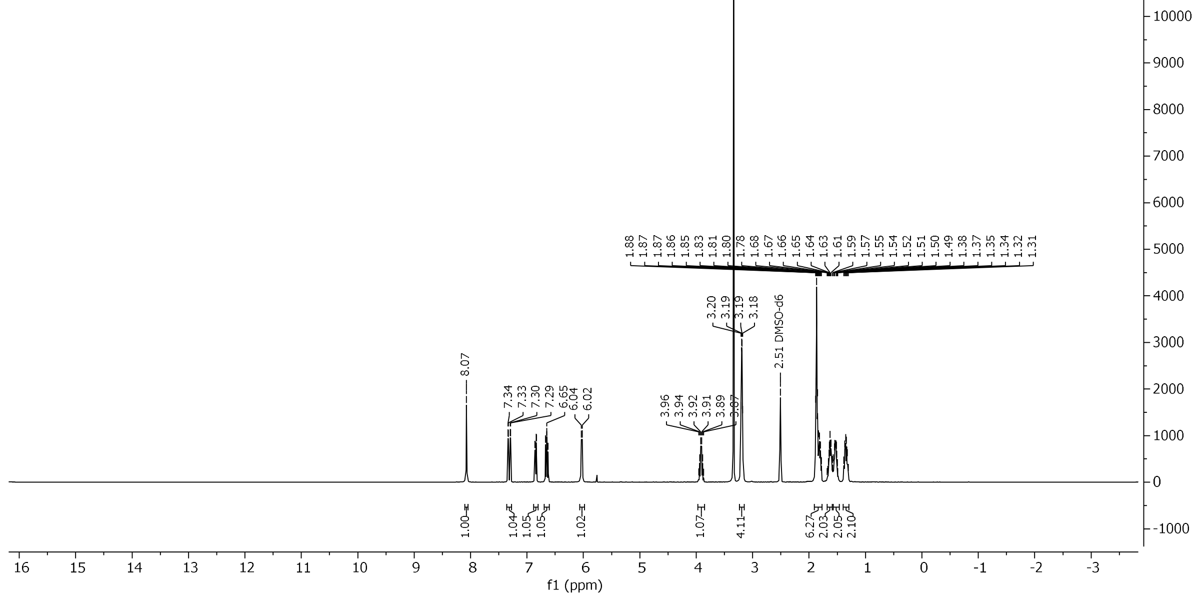

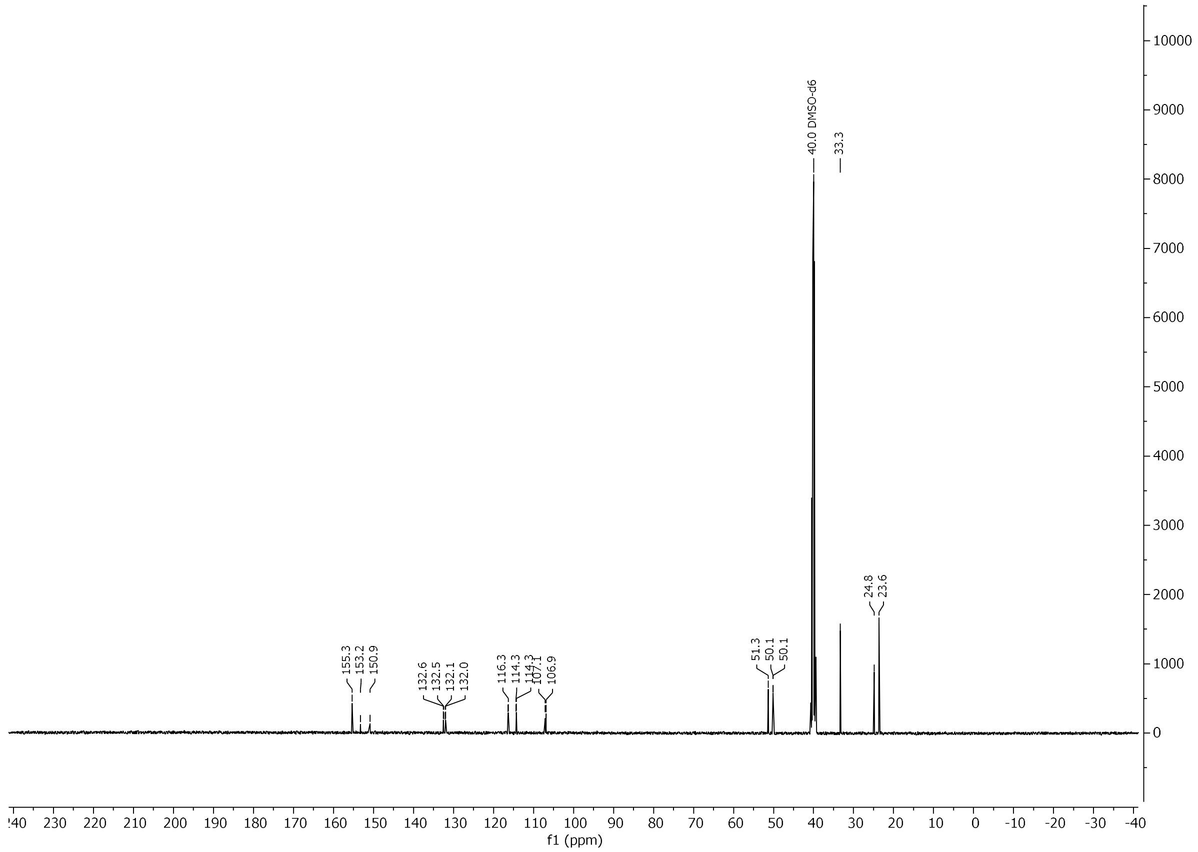

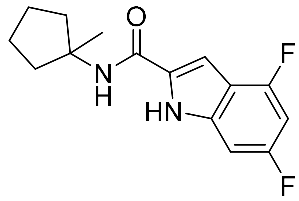

$C_{15}H_{16}F_2N_2O$

278.30 g/mol

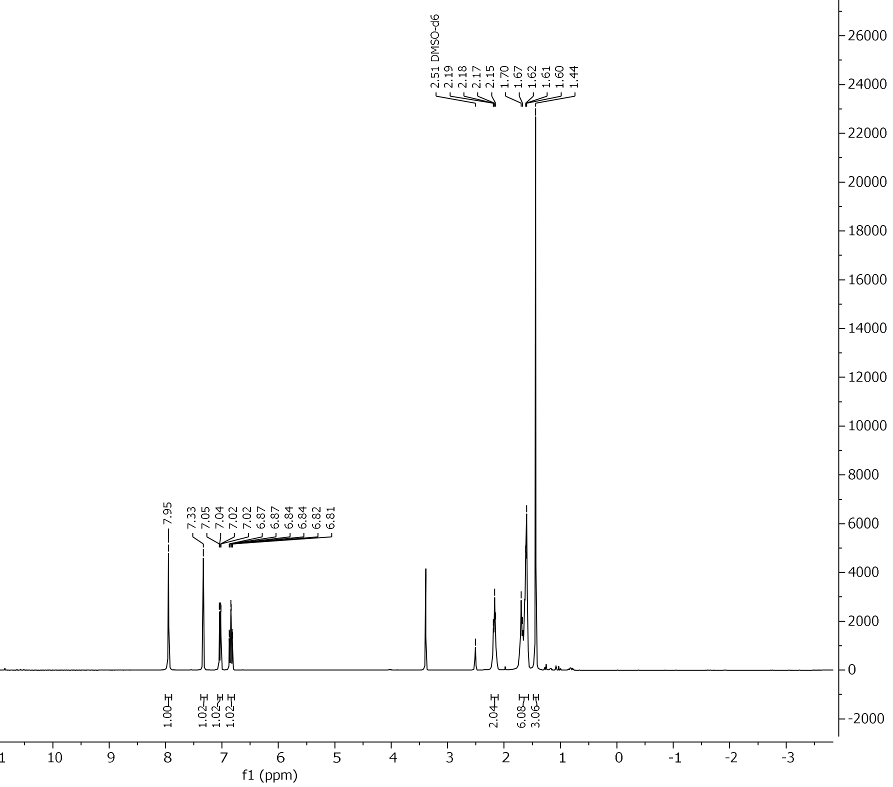

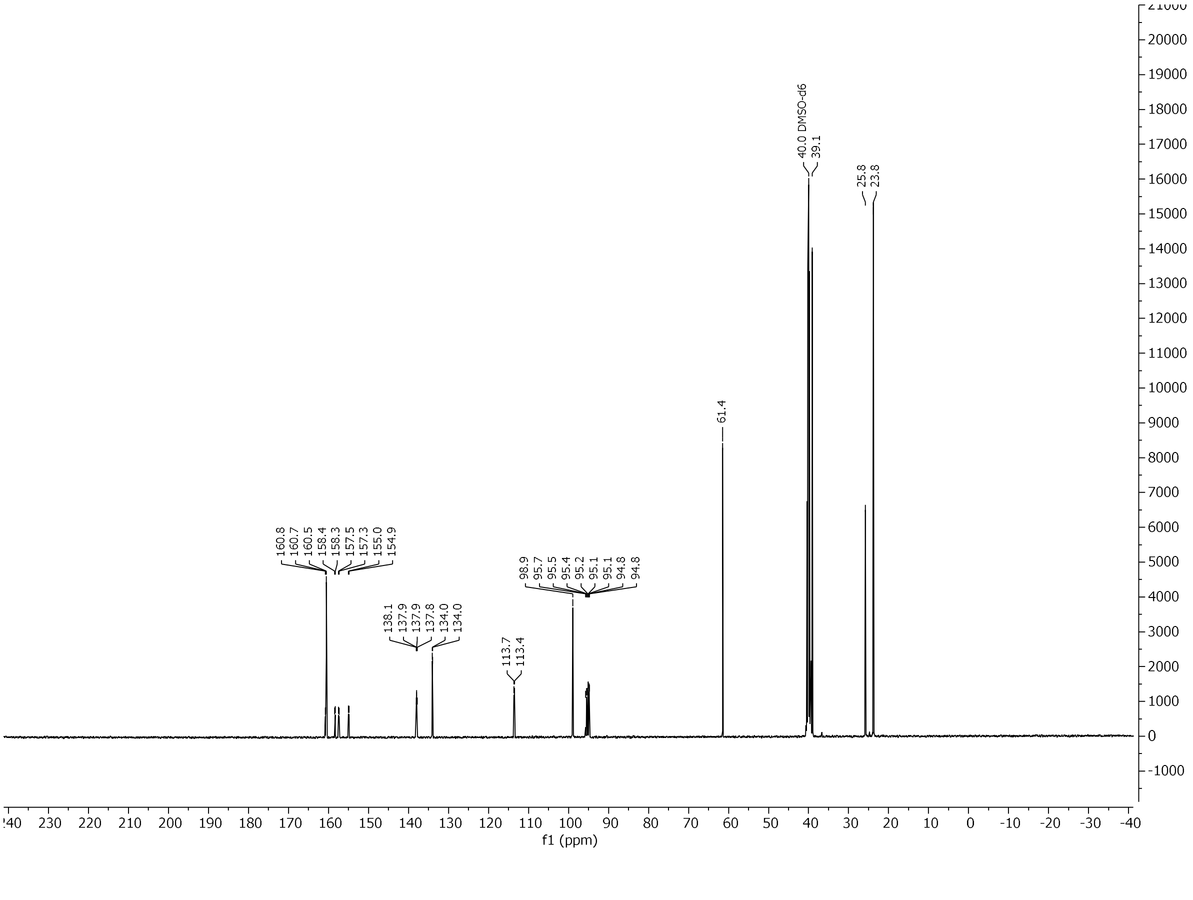

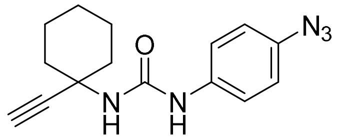

$C_{15}H_{17}N_5O$
283.34 g/mol

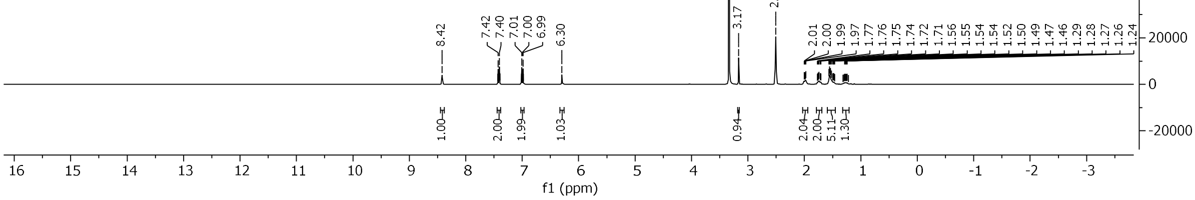

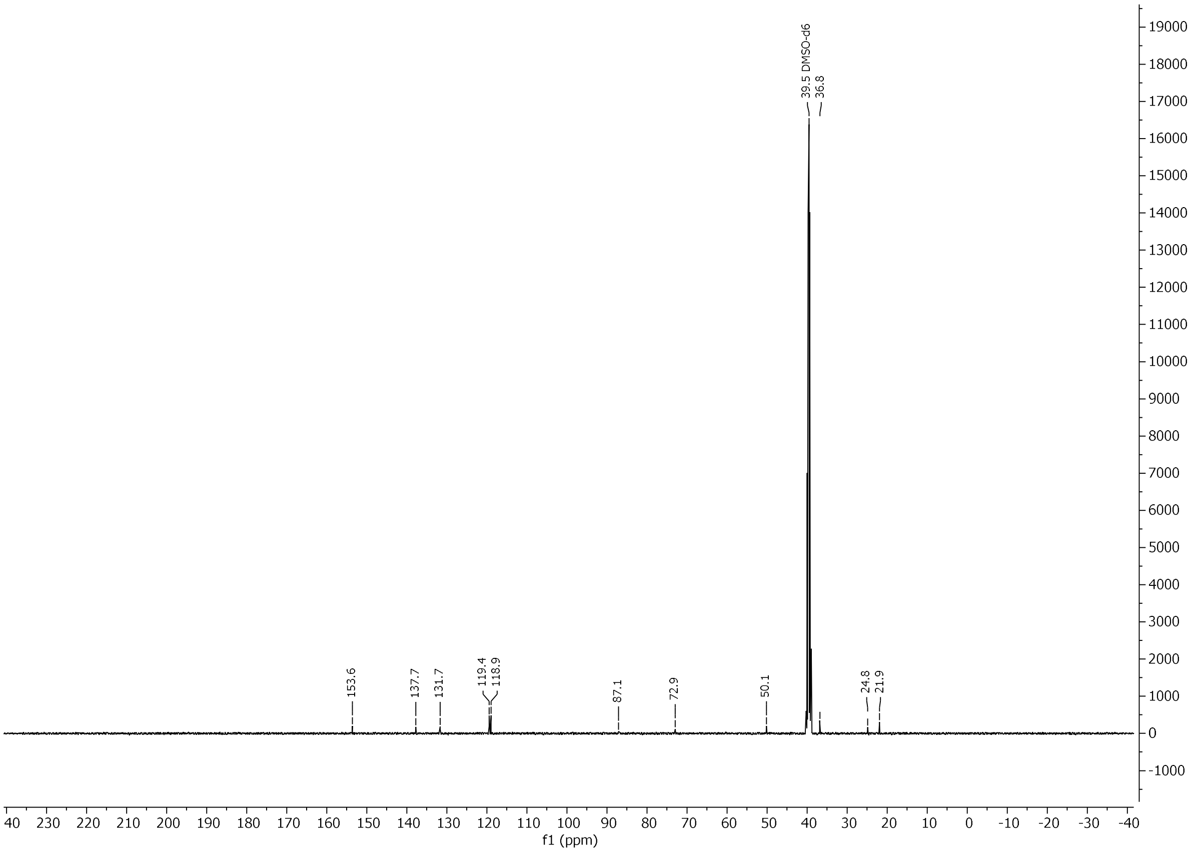

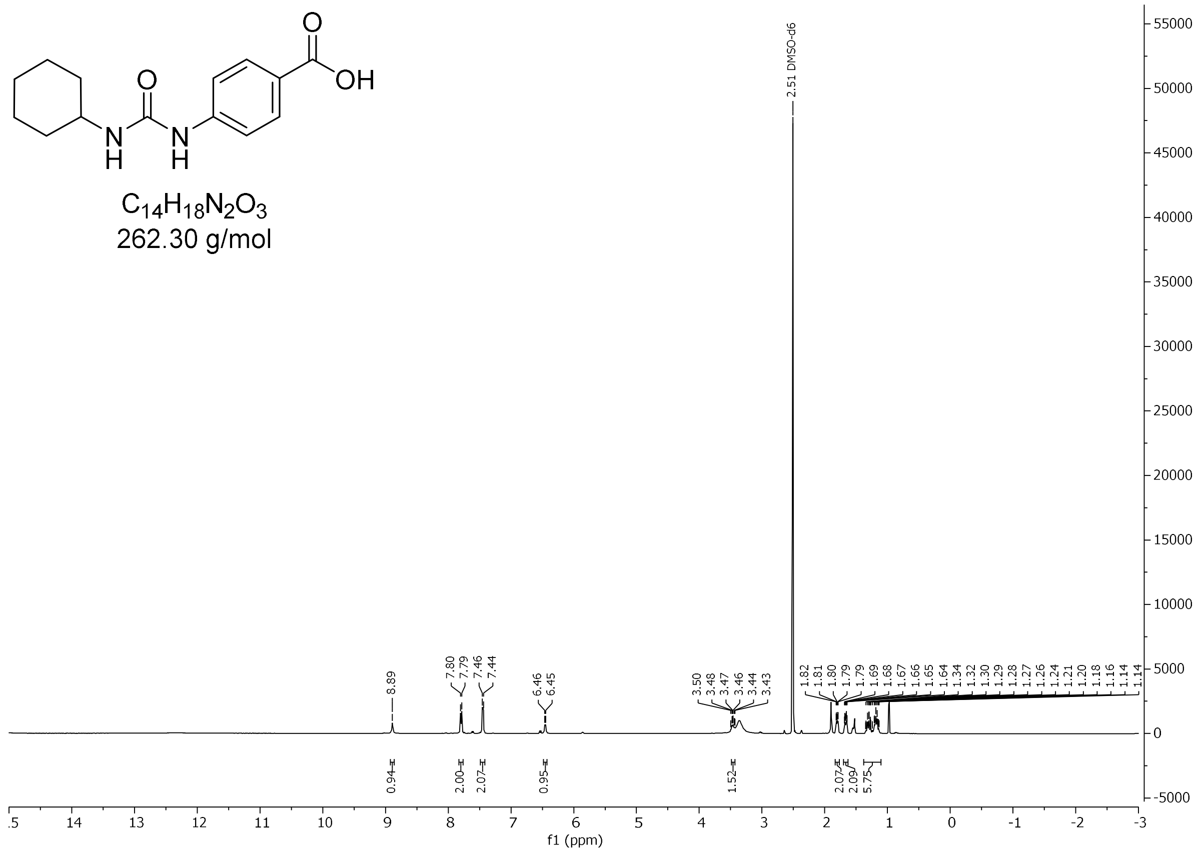

$C_{14}H_{18}N_2O_3$
262.30 g/mol

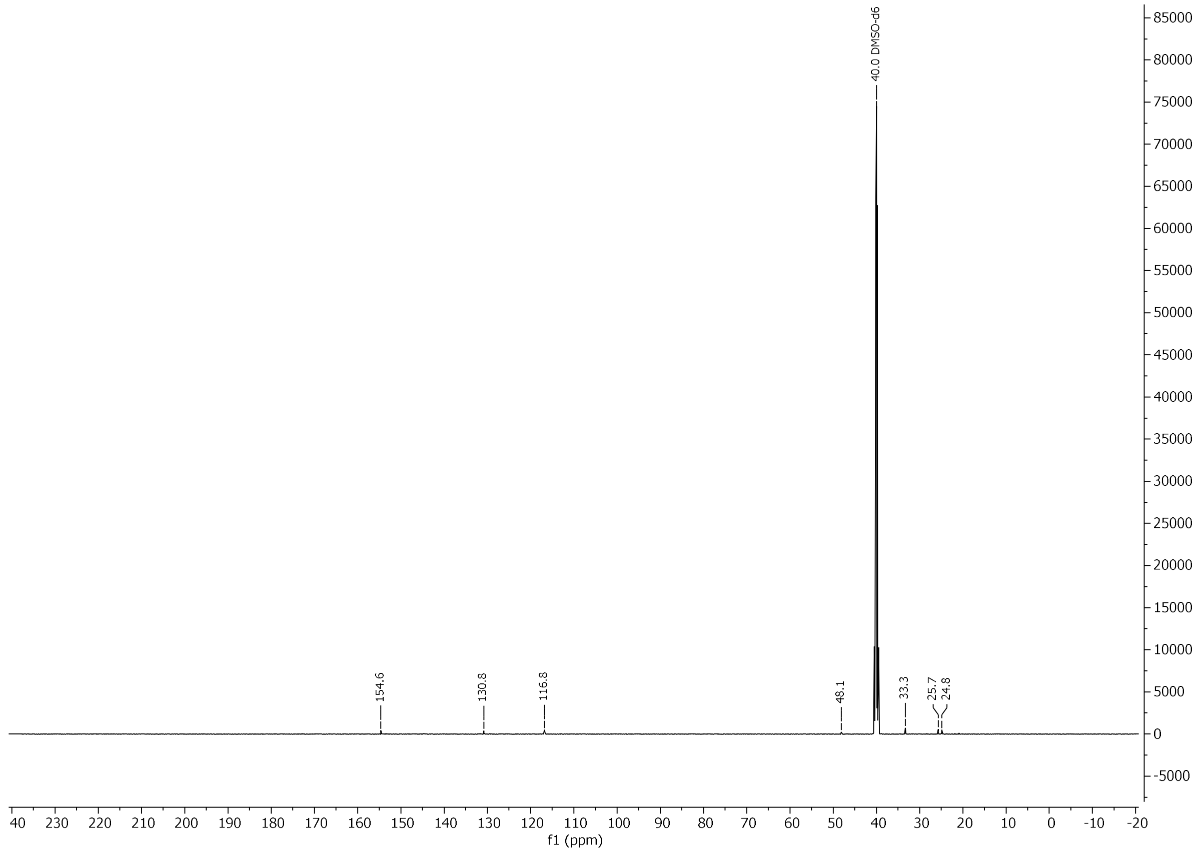

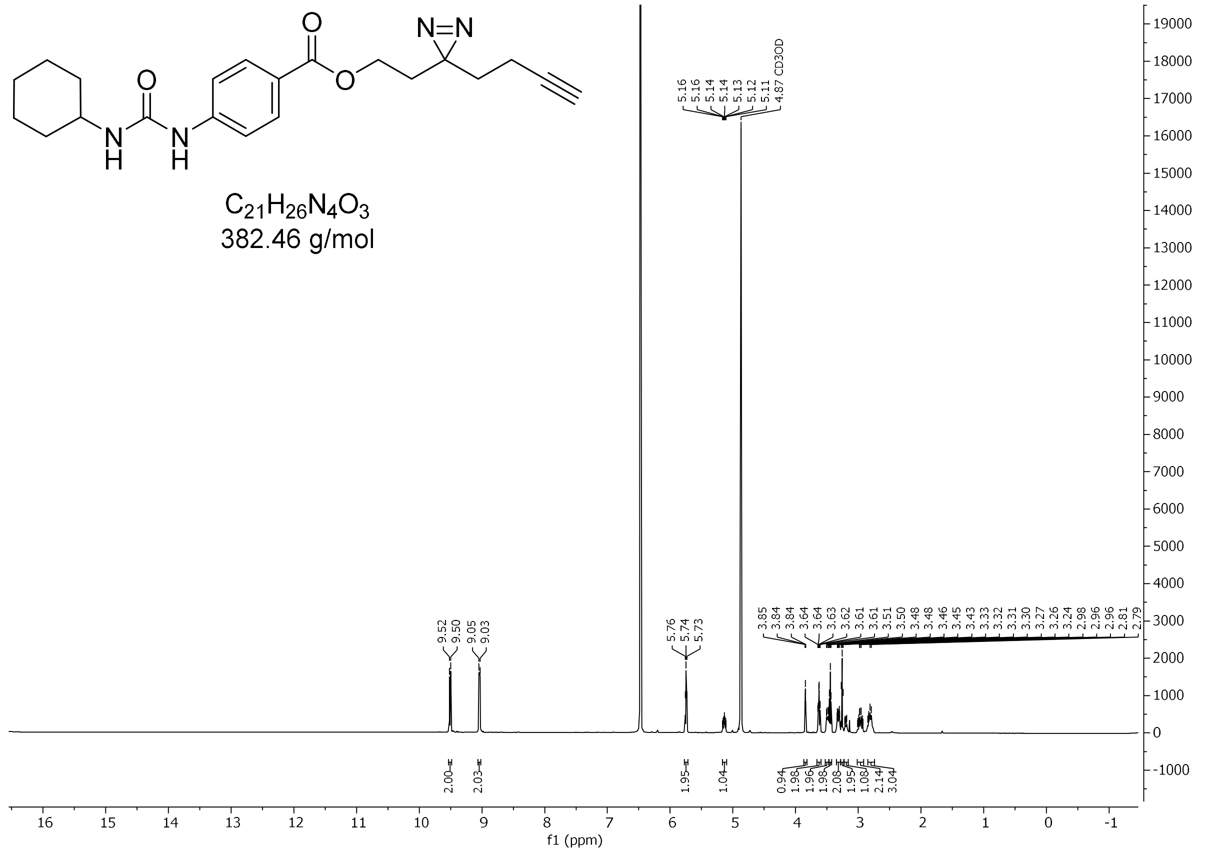
C21H26N4O3
382.46 g/mol
f1 (ppm)
4.87 CD3OD

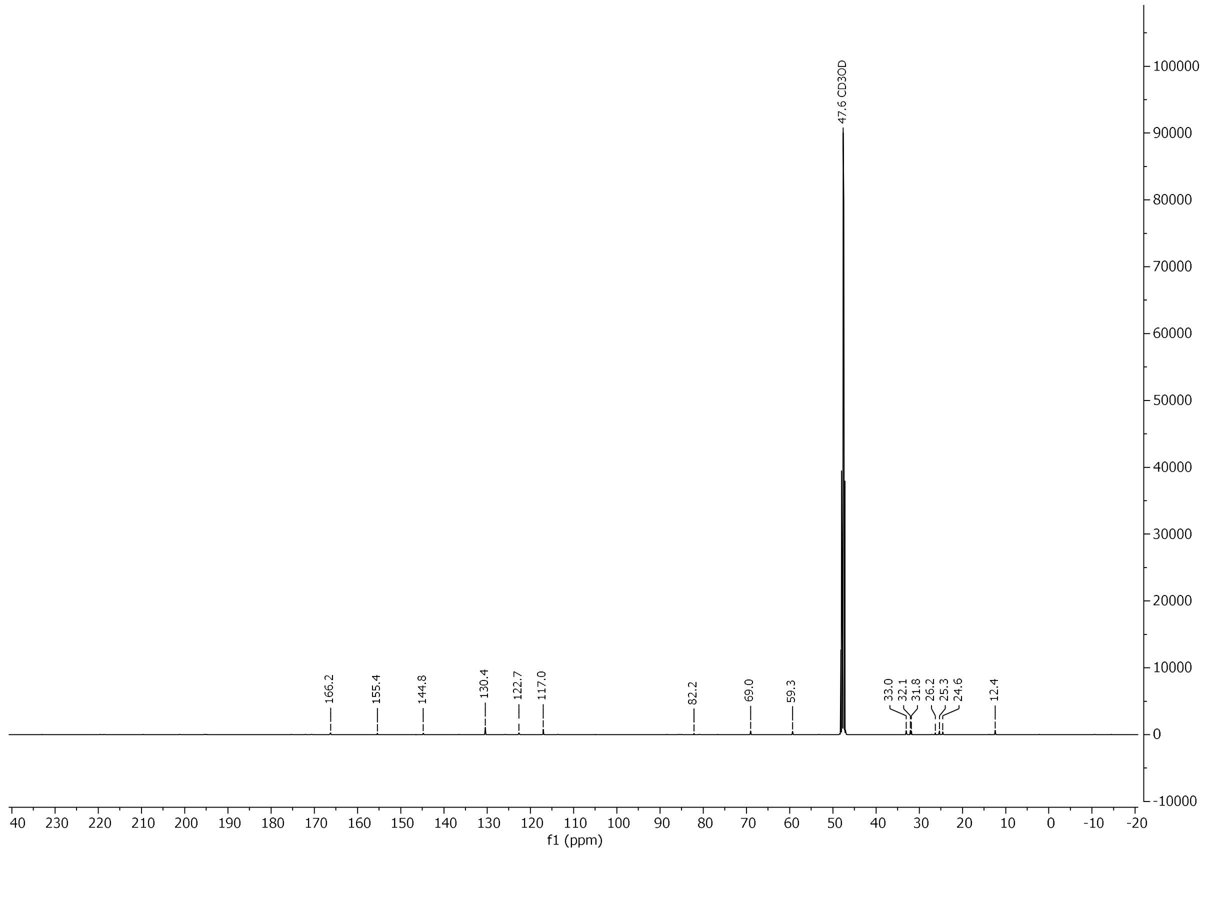
47.6 CD3OD
166.2
155.4
144.8
130.4
122.7
117.0
82.2
69.0
59.3
33.0
32.1
31.8
26.2
25.3
24.6
12.4
f1 (ppm)

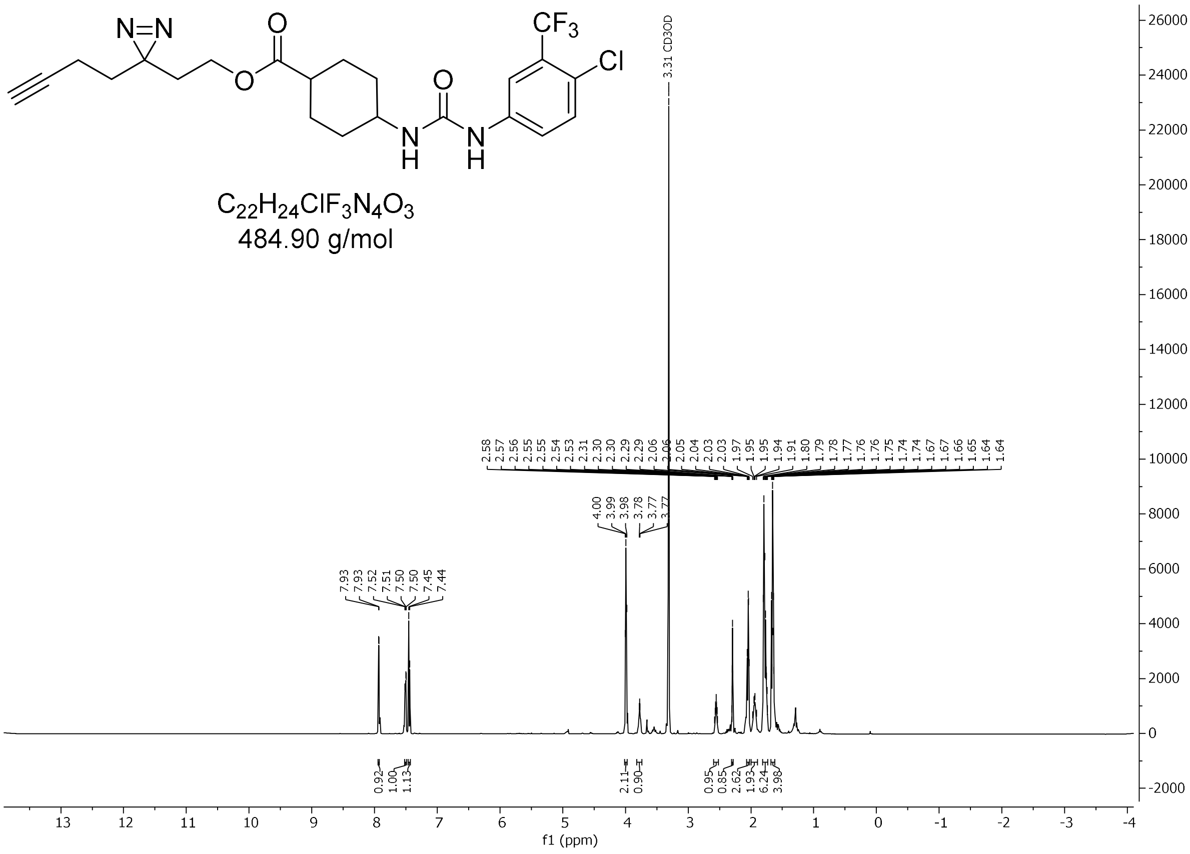
N=N
O
O
CF3
Cl
O
N
H
N
H
C22H24ClF3N4O3
484.90 g/mol
3.31 CD3OD
2.58 2.57 2.56 2.55 2.55 2.54 2.53 2.31 2.30 2.30 2.29 2.29 2.06 2.06 2.05 2.04 2.03 2.03 1.97 1.95 1.95 1.94 1.91 1.80 1.79 1.78 1.77 1.76 1.76 1.75 1.74 1.74 1.67 1.66 1.65 1.64 1.64
4.00 3.99 3.98 3.78 3.77 3.77
7.93 7.93 7.52 7.51 7.50 7.50 7.45 7.44
0.92 1.00 1.13
2.11 0.90
0.95 0.85 2.62 1.93 6.24 3.98
f1 (ppm)

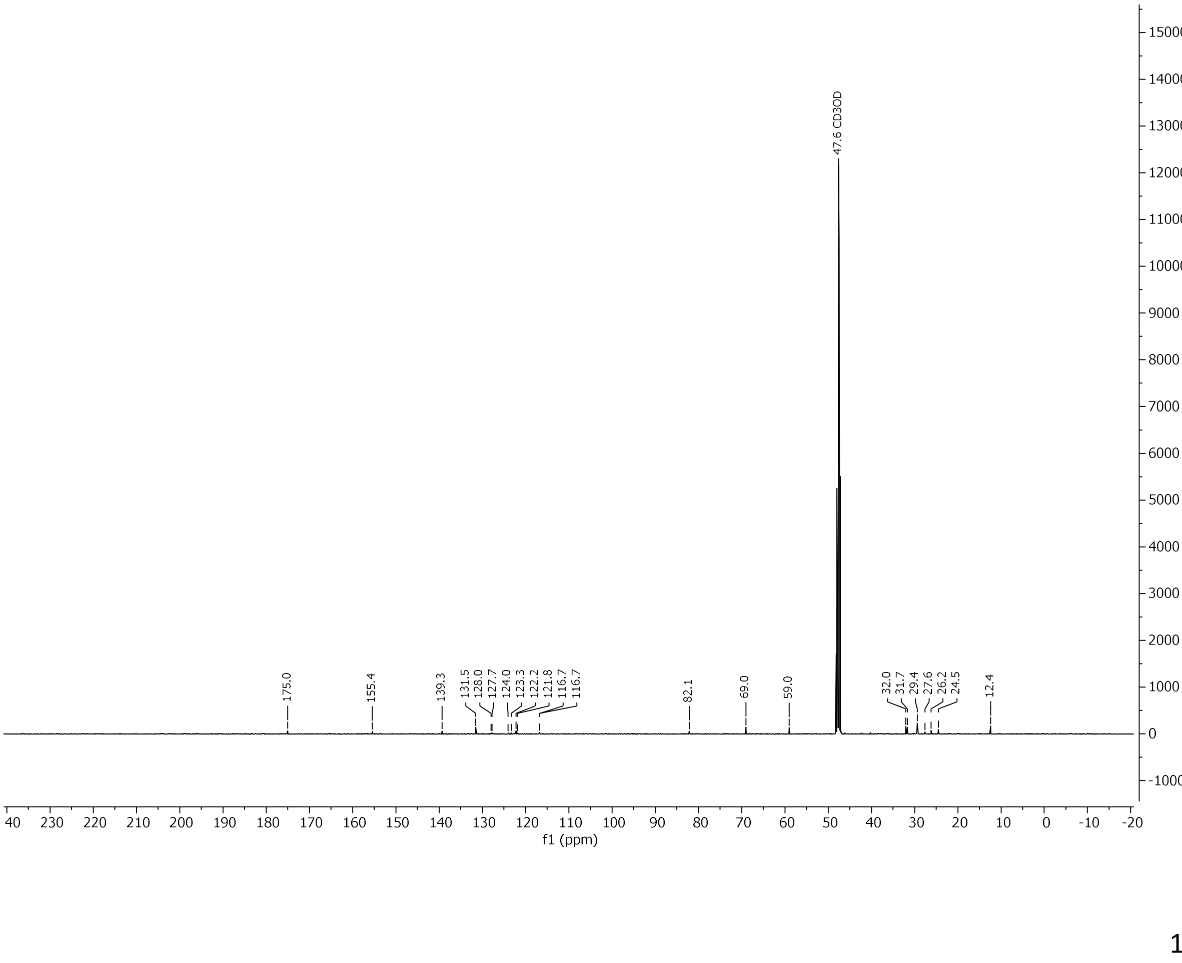
47.6 CD3OD
175.0
155.4
139.3
131.5 128.0 127.7 124.0 123.3 122.2 121.8 116.7 116.7
82.1
69.0
59.0
32.0 31.7 29.4 27.6 26.2 24.5
12.4
f1 (ppm)

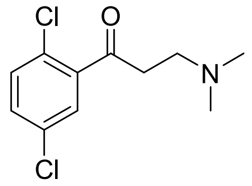

$C_{11}H_{13}Cl_2NO$
246.13 g/mol

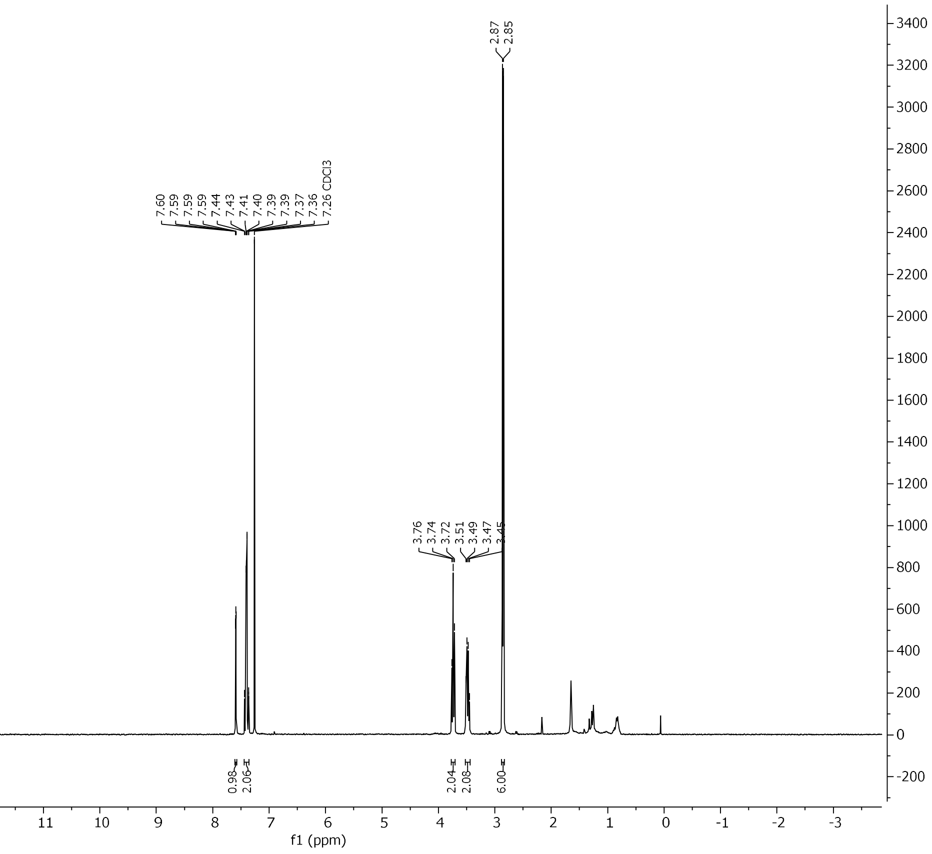

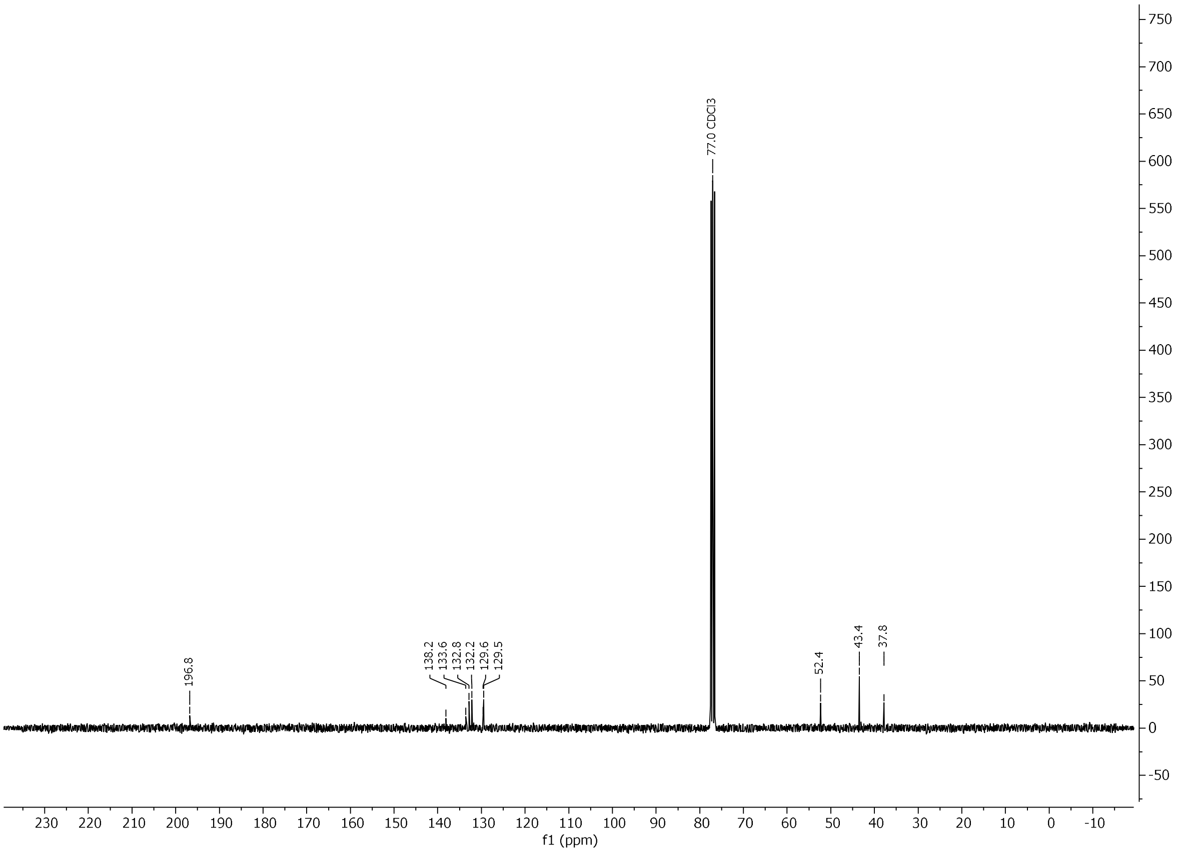

References

1 Y. Minato, D. M. Gohl, J. M. Thiede, J. M. Chacón, W. R. Harcombe, F. Maruyama and A. D. Baughn, *mSystems*, , DOI:10.1128/MSYSTEMS.00070-19.

2 M. A. Dejesus, E. R. Gerrick, W. Xu, S. W. Park, J. E. Long, C. C. Boutte, E. J. Rubin, D. Schnappinger, S. Ehrt, S. M. Fortune, C. M. Sassetti and T. R. Ioerger, *MBio*, , DOI:10.1128/MBIO.02133-16.

3 M. Sassetti, D. H. Boyd, E. J. Rubin, C. M. Sassetti, D. H. Boyd and E. J. Rubin, *Mol. Microbiol.*, 2003, **48**, 77–84.

4 J. E. Griffin, J. D. Gawronski, M. A. DeJesus, T. R. Ioerger, B. J. Akerley and C. M. Sassetti, *PLoS Pathog.*, , DOI:10.1371/JOURNAL.PPAT.1002251.

5 O. H. Vandal, L. M. Pierini, D. Schnappinger, C. F. Nathan and S. Ehrt, *Nat. Med.*, 2008, **14**, 849–854.

6 J. Cox and M. Mann, *Nat. Biotechnol.*, 2008, **26**, 1367–1372.

7 S. Tyanova, T. Temu, P. Sinitcyn, A. Carlson, M. Y. Hein, T. Geiger, M. Mann and J. Cox, *Nat. Methods*, 2016, **13**, 731–740.

8 T. Becker, A. Wiest, A. Telek, D. Bejko, A. Hoffmann-Röder and P. Kielkowski, *JACS Au*, 2022, **2**, 1712–1723.

9 F. Coscia, S. Doll, J. M. Bech, L. Schweizer, A. Mund, E. Lengyel, J. Lindebjerg, G. I. Madsen, J. M. Moreira and M. Mann, *J. Pathol.*, 2020, **251**, 100–112.

10 V. Demichev, C. B. Messner, S. I. Vernardis, K. S. Lilley and M. Ralser, *Nat. Methods*, 2020, **17**, 41–44.

11 Y. Perez-Riverol, J. Bai, C. Bandla, D. García-Seisdedos, S. Hewapathirana, S. Kamatchinathan, D. J. Kundu, A. Prakash, A. Frericks-Zipper, M. Eisenacher, M. Walzer, S. Wang, A. Brazma and J. A. Vizcaíno, *Nucleic Acids Res.*, 2022, **50**, D543–D552.

12 S. Rao, K. Bockstael, S. Nath, Y. Engelborghs, J. Anné and N. Geukens, *FEBS J.*, 2009, **276**, 3222–3234.

13 S. Grover, C. A. Engelhart, E. Pérez-Herrán, W. Li, K. A. Abrahams, K. Papavinasasundaram, J. M. Bean, C. M. Sassetti, A. Mendoza-Losana, G. S. Besra, M. Jackson and D. Schnappinger, *ACS Infect. Dis.*, 2021, **7**, 141–152.

14 R. J. W. Lambert and J. Pearson, *J. Appl. Microbiol.*, 2000, **88**, 784–790.

15 I. V Krieger, S. Yalamanchili, P. Dickson, C. A. Engelhart, M. D. Zimmerman, J. Wood, E. Clary, J. Nguyen, N. Thornton, P. A. Centrella, B. Chan, J. W. Cuozzo, M. Gengenbacher, M.-A. Guié, J. P. Guilinger, C. Bienstock, H. Hartl, C. D. Hupp, R. Jetson, T. Satoh, J. T. S. Yeoman, Y. Zhang, V. Dartois, D. Schnappinger, A. D. Keefe and J. C. Sacchettini, *ACS Infect. Dis.*, 2024, **10**, 1561–1575.

16 B. Zhang, J. Li, X. Yang, L. Wu, J. Zhang, Y. Yang, Y. Zhao, L. Zhang, X. Yang, X. Yang, X. Cheng, Z. Liu, B. Jiang, H. Jiang, L. W. Guddat, H. Yang and Z. Rao, *Cell*, , DOI:10.1016/j.cell.2019.01.003.

17 A. Fiser, R. Kinh, G. Do and A. S. ˇ Ali, *Protein Sci.*, 2000, **9**, 1753–1773.

18 M. A. Martí-Renom, A. C. Stuart, A. Fiser, R. Sánchez, F. Melo and A. Šali, *Annu. Rev. Biophys.*

Biomol. Struct., 2000, **29**, 291–325.

19 G. M. Morris, H. Ruth, W. Lindstrom, M. F. Sanner, R. K. Belew, D. S. Goodsell and A. J. Olson, *J. Comput. Chem.*, 2009, **30**, 2785–2791.

20 M. D. Hanwell, D. E. Curtis, D. C. Lonie, T. Vandermeerschd, E. Zurek and G. R. Hutchison, *J. Cheminform.*, 2012, **4**, 1–17.

21 N. M. O'Boyle, M. Banck, C. A. James, C. Morley, T. Vandermeersch and G. R. Hutchison, *J. Cheminform.*, 2011, **3**, 1–14.

22 J. Eberhardt, D. Santos-Martins, A. F. Tillack and S. Forli, *J. Chem. Inf. Model.*, 2021, **61**, 3891–3898.

23 O. Trott and A. J. Olson, *J. Comput. Chem.*, 2010, **31**, 455–461.
